# Asymmetric Henry Reaction Using Cobalt Complexes with Bisoxazoline Ligands Bearing Two Fluorous Tags

**DOI:** 10.3390/molecules28227632

**Published:** 2023-11-16

**Authors:** Kazuki Ishihara, Yamato Kato, Narisa Takeuchi, Yuka Hayashi, Yuna Hagiwara, Shyota Shibuya, Tohya Natsume, Masato Matsugi

**Affiliations:** Faculty of Agriculture, Meijo University, 1-501, Shiogamaguchi, Tempaku-ku, Nagoya 468-8502, Japan233561503@ccmailg.meijo-u.ac.jp (Y.K.); 233561035@ccmailg.meijo-u.ac.jp (Y.H.);

**Keywords:** fluorous, ligand, oxazoline, Henry reaction, absolute configuration

## Abstract

The effect of the presence of fluorous tags in bisoxazoline ligands on the stereoselectivity of the cobalt-catalyzed asymmetric Henry reaction was investigated. In contrast to the stereoselectivity obtained with conventional nonfluorous ligands, using bisoxazoline bidentate ligands featuring two fluorous tags in adjacent positions on the aromatic ring yielded a reversed stereoselectivity. The stereoselectivity also reversed when the fluorous tags were replaced with alkyl chains of equivalent length, albeit to a considerably lesser degree, highlighting the effect of the fluorous tags.

## 1. Introduction

Optically active *C*_2_-symmetrical bisoxazoline (BOX) ligands are among the most versatile ligands used in organic synthesis [1,2,3,4]. Since Evans et al. reported the asymmetric cyclopropanation reaction [5] using Cu/BOX complexes in 1991, various BOX ligands with structural tunability have been developed [6,7,8,9,10,11]. Using these ligands in reactions such as the Henry reaction [12,13,14], Mukaiyama aldol reaction [15], Michael additions [16,17], and Friedel–Crafts reactions [18] furnishes the target products in high asymmetric yields.

BOX ligands bearing fluorous tags are particularly interesting because they repel water and polar organic compounds while exhibiting a strong mutual affinity [19] among fluorous molecules. These ligands, which have been used in asymmetric reactions including allylic alkylation [20], cyclopropanation [21], and the Henry reaction [22], can be selectively isolated and recovered from reaction mixtures via solid-phase extraction using fluorous silica gel [23] or liquid-phase extraction using fluorous solvents [24]. By incorporating a fluorous tag into the BOX ligand for the first time in asymmetric Henry reactions, Cai et al. achieved high stereoselectivity and recovery [22]. Our study involves the use of BOX ligands incorporating two fluorous tags at different positions on the ligand in asymmetric Henry reactions.

**Figure 1:**
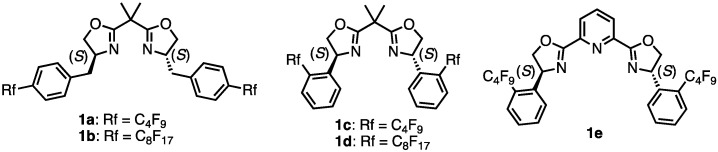
Fluorous bisoxazoline ligands containing two fluorous tags (**1a**–**e**).

Previously, we achieved the first asymmetric epoxidation of an isolated carbon–carbon double bond using an iron salen complex incorporating a fluorous tag at the 3,3′-position of the salen ligand [25]. Apart from its recycling ability, this complex exhibits a unique characteristic stemming from the two fluorous tags; namely, the conformational fixation via intramolecular stacking between neighboring fluorous tags forms a distorted asymmetric structure and creates a unique catalytic reaction field. We postulated that the introduction of two adjacent fluorous tags on freely rotatable substituents in BOX ligands could lock the coordination in place through a stacking between the tags, which would lead to different reactivity and stereoselectivity compared with conventional catalysts. Therefore, we synthesized a series of BOX ligands containing two fluorous tags and investigated their reactivity and stereoselectivity in the asymmetric Henry reaction [26], which is a useful carbon–carbon bond formation reaction. Here, we describe an asymmetric Henry reaction using BOX bidentate ligands **1a**–**1d** with two fluorous tags introduced in spatially adjacent positions and pincer-type BOX tridentate ligand **1e** (Figure 1). For bisoxazoline catalysts, fluorous tag catalysts via flexible benzyl spacers (**1a** and **1b**) and more rigid aryl-type fluorous catalysts (**1c** and **1d**) were investigated. Ligands with freely rotating methylene groups (**1a** and **1b**) and ligands with rigid aromatic planar ring structures (**1c** and **1d**) have different steric environments, potentially resulting in significant differences in stereoselectivity.

## 2. Results and Discussions

**Scheme 1:**
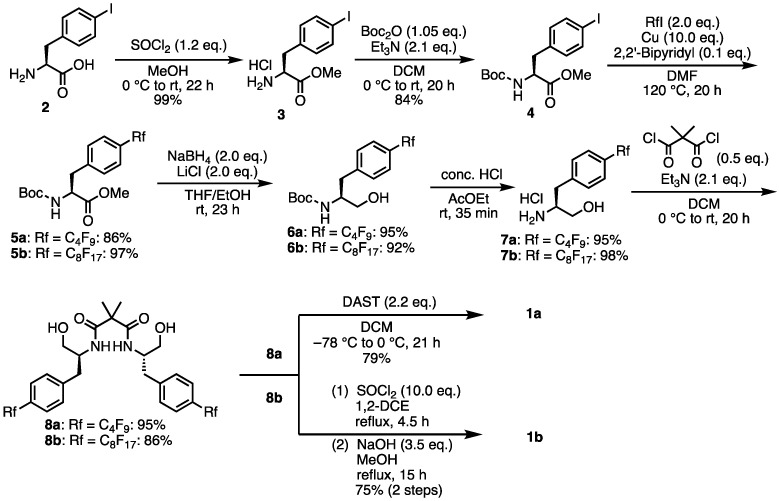
Synthetic route to fluorous bisoxazoline ligands **1a** and **1b**.

Scheme 1 outlines the synthetic pathway for bidentate ligands **1a** and **1b**. First, methylation of 4-iodo-L-phenylalanine **2** afforded **3**, which was Boc-protected to yield compound **4**. Subsequently, the introduction of C_4_F_9_ and C_8_F_17_ fluorous tags was achieved via Ullmann coupling, resulting in intermediates **5a** and **5b**, respectively. After ester reduction and removal of the Boc group, compounds **7a** and **7b** were obtained. The synthesis culminated with the condensation with dimethylmalonyl chloride and subsequent cyclization, furnishing oxazoline ligands **1a** and **1b** bearing the C_4_F_9_ and C_8_F_17_ tags, respectively.

**Scheme 2:**
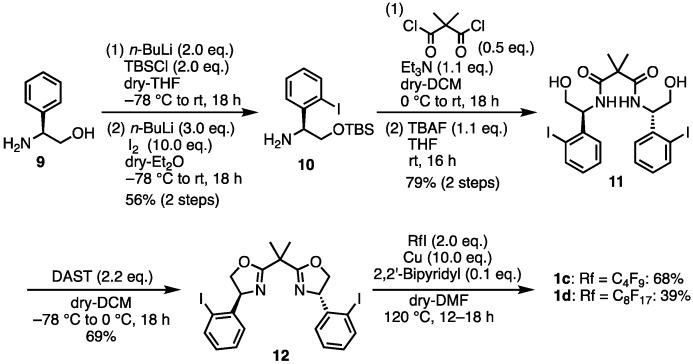
Synthetic route to fluorous bisoxazoline ligands **1c** and **1d**.

Ligands **1c** and **1d** were synthesized following the reaction pathway outlined in Scheme 2. (*S*)-2-Phenylglycinol **9** was iodinated to obtain compound **10**. After condensation with dimethylmalonyl chloride and subsequent deprotection of the TBS group, intermediate **11** was obtained. Then, a cyclization reaction using (diethylamino)sulfur trifluoride (DAST) afforded BOX intermediate **12**. Finally, C_4_F_9_ and C_8_F_17_ fluorous tags were introduced via Ullmann coupling, affording BOX ligands **1c** and **1d**, respectively.

**Scheme 3:**
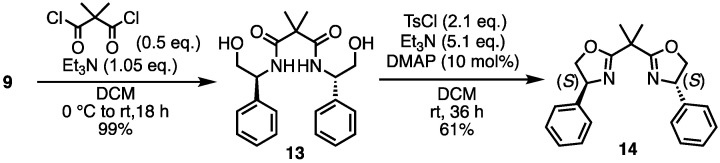
Synthesis of nonfluorous bisoxazoline ligand **14**.

As a control ligand, unsubstituted ligand **14** [27] without fluorous tags was also synthesized from (*S*)-2-phenylglycinol **9** via a condensation reaction with dimethyl malonyl chloride followed by cyclization (Scheme 3).

First, the as-synthesized fluorous BOX ligands were investigated as chiral ligands in the Henry reaction. Using *p*-nitrobenzaldehyde and nitromethane as substrates, various metal sources were employed in the Henry reaction in the presence of ligand **1a** in *^i^*PrOH. When Cu(OAc)_2_·H_2_O was utilized, the reaction proceeded with a 97% conversion and 69% enantiomeric excess (*ee*), predominantly yielding the *S*-aldol addition product (Table 1, entry 1). In contrast, no reaction occurred when using CuCl_2_, CuBr_2_, or Cu(OTf)_2_ (Table 1, entries 2–4). Meanwhile, Co(OAc)_2_ and Zn(OAc)_2_ gave high conversion rates but nearly racemic products (Table 1, entries 5 and 6). Therefore, Cu(OAc)_2_·H_2_O was selected as the metal source for subsequent reactions. 

Next, fluorophobic (*^i^*PrOH and 50% *^i^*PrOH aqueous solution) and fluorophilic solvents (THF and FC-72) were employed as reaction media using ligands **1a**–**d** to investigate the solvent effect in the reaction. Although differences in affinity between the fluorinated ligands and solvents were expected to affect the stereoselectivity, no remarkable impact on stereoselectivity was observed in reactions using **1a** and **1b**. When catalysts (**1a** and **1b**) with fluorous tags via benzyl spacers were used, a moderate level of stereoselectivity was obtained in favor of the formation of *S*-aldol addition products in all cases. (Table 2, entries 1–7).

Interestingly, when fluorous ligands **1c** and **1d** were used as chiral sources, the reverse stereoselectivity was observed even though all ligands **1a**–**1d** exhibited the same absolute stereochemistry (*S*,*S*), favoring the *R*-aldol addition product with moderate stereoselectivity (Table 2, entries 8–13 vs. entries 1–7, 14). This result suggests that the fluorinated aryl BOX ligands at the 2′ position (**1c** and **1d**) create a distinct asymmetric environment compared with the other ligands used. Although there was no substantial difference in stereoselectivity based on differences in reaction solvent and tag length in either case, the combination of ligand **1c** and *^i^*PrOH yielded the highest reverse stereoselectivity (53% *ee*) (Table 2, entry 8).

**Scheme 4:**
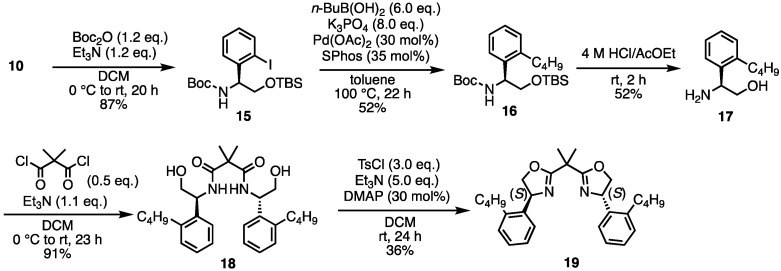
Synthesis of nonfluorous butylated bisoxazoline ligand **19**.

A control experiment was conducted using ligand **19**, which is the nonfluorinated version of **1c**, under the same conditions. Interestingly, as in the case of fluorinated ligand **1c**, a preference for the aldol addition to the *R*-isomer was observed. However, the stereoselectivity drastically decreased to 14% *ee* (Table 2, entry 15). These results indicate that the steric bulkiness at the 2′-position on the aryl-type BOX ligand is a factor that reverses the stereoselectivity. Introducing a fluorous tag at the 2′-position does not substantially affect the reaction result, but it does affect the stereoselectivity more than the corresponding nonfluorous alkyl substituent. Note that ligand **19** was synthesized using the route depicted in Scheme 4.

**Scheme 5:**
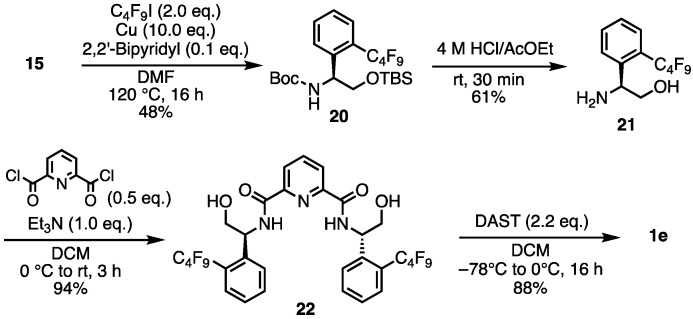
Synthesis of pincer-type fluorous bisoxazoline ligand **1e**.

**Scheme 6:**
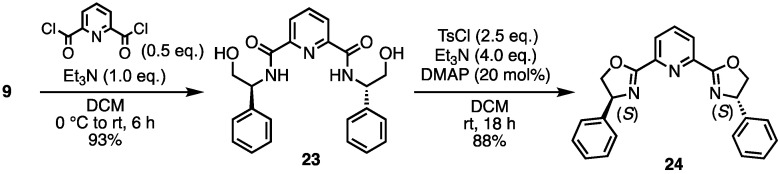
Synthesis of pincer-type nonfluorous bisoxazoline ligand **24**.

Next, pincer-type fluorinated ligand **1e**, which was synthesized using the route depicted in Scheme 5, was applied to the present reaction. Briefly, iodinated compound **15** was first protected with Boc and then the C_4_F_9_ tag was introduced via the Ullmann reaction, forming intermediate **20**. Under acidic conditions, both the TBS and Boc groups were simultaneously removed, and the subsequent condensation reaction with 2,6-pyridinedicarbonyl dichloride yielded **22**. Finally, ligand **1e** was synthesized through a ring-closure reaction using DAST. Furthermore, unsubstituted ligand **24** [28] was synthesized as a control ligand via condensation and cyclization reactions with 2,6-pyridinedicarbonyl dichloride using (*S*)-2-phenylglycinol **9** as the substrate (Scheme 6).

Table 3 shows the results of asymmetric Henry reactions using pincer-type ligands **1e** and **2**. The combination of **1e** and Co(OAc)_2_ afforded the *R*-addition product with slightly higher stereoselectivity than that obtained when using Cu(OAc)_2_·H_2_O or Zn(OAc)_2_ (Table 3, entries 1–3).

Solvent effects were investigated using a 50% *^i^*PrOH aqueous solution, THF, and FC-72 as reaction solvents (Table 3, entries 4–6). The reaction proceeded quantitatively in a 50% *^i^*PrOH aqueous solution but with little stereoselectivity. Using THF resulted in better stereoselectivity, and the target product was obtained with an optical purity of 42% *ee*. The reaction did not proceed when the fluorous solvent FC-72 was used.

The *R*-product was predominantly obtained in experiments conducted in THF with pincer-type ligand **24** lacking fluorous tags. However, the stereoselectivity was lower (8% *ee*) than when using fluorous **1e** (Table 3, entry 5 vs. entry 7). This suggests that introducing the fluorous tags into the pincer-type ligand enhances the stereoselectivity of this reaction.

## 3. Experimental Section

### 3.1. Materials and Reagents

All the laboratory chemicals were purchased from Tokyo Chemical Industry Co., Ltd. (Tokyo, Japan), FUJIFILM Wako Pure Chemical Corporation (Richmond, VA, USA), Sigma-Aldrich Co. LLC (St. Louis, MO, USA), and Kanto Chemical Co., Inc. (Tokyo, Japan) and used without further purification unless otherwise stated. Solvents were removed using rotary evaporation under reduced pressure using a water bath at 40 °C–50 °C. Nonvolatile compounds were dried in vacuo at 0.01 mbar. All reactions were stirred magnetically and monitored using thin-layer chromatography using silica gel plates. Purification by chromatography was performed on silica gel 60 N (spherical, neutral, 63–210 µm, Kanto Chemical Co., Inc.).

### 3.2. Analytical Instruments

Melting points were determined in open-ended capillaries using a Bibby Scientific Ltd. (Stone, UK) Stuart^®^ SMP-30 instrument or ATM-01 of AS ONE Corporation and are uncorrected. Nuclear magnetic resonance (NMR) spectra were recorded using JNM-EX270 (^1^H: 270 MHz) and JNM-ECZ400S (^1^H: 400 MHz, ^13^C: 101 MHz, ^19^F: 376 MHz) spectrometers. Chemical shifts (δ) are given in ppm, and coupling constants (*J*) are given in Hz. Abbreviations for multiplicity are as follows: s (singlet), d (doublet), t (triplet), m (multiplet), dd (double doublet), and br s (broad signal). High-performance liquid chromatography (HPLC) analyses were performed using Daicel Chiralpack IA-3 columns with UV Detector L2400 or SPD-20A. High-resolution mass spectra were obtained using Exactive^TM^ Plus Orbitrap (Thermo Fisher Scientific, Waltham, MA, USA). The spectra were calibrated with Pierce^TM^ LTQ Velos ESI Positive Ion Calibration Solution prior to data acquisition.

### 3.3. General Procedure for the Henry Reaction

A mixture of the ligand (0.012 mmol) and the metal salt (0.011 mmol) was stirred in a solvent (328 μL) at room temperature for 1 h. Aldehyde (0.22 mmol) and MeNO_2_ (2.19 mmol) were added to the reaction mixture, which was then stirred at room temperature for 22 h. The reaction mixture was concentrated. The conversion was determined by ^1^H NMR analysis of the crude product. The *ee* of the product was determined with HPLC of the crude product.

### 3.4. Synthesis

Methyl (*S*)-2-amino-3-(4-iodophenyl)propanoate hydrochloride (**3**) [29]. Under N_2_ atmosphere, a solution of 4-iodo-L-phenylalanine **2** (5.02 g, 17.23 mmol) in dry-MeOH (80 mL) was stirred at 0 °C, then SOCl_2_ (1.5 mL, 20.55 mmol) was added dropwise. The reaction mixture was warmed to room temperature and then stirred for 22 h. The reaction mixture was concentrated. After the addition of Et_2_O, the suspension was purified by filtration to give the desired product **3** (5.84 g, 17.10 mmol, 99%). White solid; mp 191–192 °C; ^1^H NMR (400 MHz, (CD_3_)_2_SO) δ 8.55 (br s, 3H), 7.70 (d, *J* = 8.4 Hz, 2H), 7.06 (d, *J* = 8.4 Hz, 2H), 4.30–4.26 (m, 1H), 3.69 (s, 3H), 3.14–3.04 (m, 2H).
Methyl (*S*)-2-((*tert*-butoxycarbonyl)amino)-3-(4-iodophenyl)propanoate (**4**) [30]. A solution of methyl (*S*)-2-amino-3-(4-iodophenyl)propanoate hydrochloride **3** (252.3 mg, 0.74 mmol) and Et_3_N (213 μL, 1.53 mmol) in dry-DCM (4 mL) was stirred at 0 °C. A solution of Boc_2_O (167.7 mg, 0.77 mmol) in dry-DCM (3 mL) was slowly added to the mixture. Then the reaction temperature rose to room temperature and the mixture was stirred for a further 20 h. The mixture was diluted with brine. The reaction mixture was extracted with DCM. The organic layer was dried over Na_2_SO_4_, filtered, and concentrated. The crude was purified by silica gel chromatography (ethyl acetate:*n*-hexane = 1:1) to give the desired product **4** (250.2 mg, 0.62 mmol, 84%). White solid; mp 74–75 °C; ^1^H NMR (400 MHz, CDCl_3_) δ 7.62 (d, *J* = 8.4 Hz, 2H), 6.88 (d, *J* = 8.0 Hz, 2H), 4.98–4.96 (m, 1H), 4.58–4.56 (m, 1H), 3.72 (s, 3H), 3.10–2.95 (m, 2H), 1.42 (s, 9H).
Methyl (*S*)-2-((*tert*-butoxycarbonyl)amino)-3-(4-(perfluorobutyl)phenyl)propanoate (**5a**). To a mixture of **4** (1.03 g, 2.54 mmol), Cu powder (1.60 g, 25.12 mmol), 2,2′-bipyridyl (38.8 mg, 0.25 mmol), and DMF (5 mL) was added a 1,1,1,2,2,3,3,4,4-nonafluoro-4-iodobutane (0.8 mL, 4.76 mmol). The mixture was stirred at 120 °C for 19 h. The mixture was filtered through Celite, and the solids were washed with MeOH. The filtrate was concentrated. Then 1 M HCl aq. was added to the suspension. The aqueous layer was extracted with ethyl acetate. The organic layer was washed with brine, dried over Na_2_SO_4_, filtered, and concentrated. The crude was purified by silica gel chromatography (ethyl acetate:*n*-hexane = 1:3) to give the desired product **5a** (1.08 g, 2.18 mmol, 86%). Yellow solid; mp 40–41 °C; ^1^H NMR (270 MHz, CDCl_3_) δ 7.52 (d, *J* = 8.1 Hz, 2H), 7.30–7.27 (m, 2H), 5.06–5.03 (m, 1H), 4.65–4.62 (m, 1H), 3.72 (s, 3H), 3.25–3.04 (m, 2H), 1.40 (s, 9H); ^19^F NMR (376 MHz, CDCl_3_) δ −80.95 (3F), −110.79 (2F), −122.71 (2F), −125.51 (2F); ^13^C NMR (101 MHz, CDCl_3_) δ 172.03, 155.04, 140.77, 129.74, 127.64, 127.07,116.14–110.67, 80.26, 54.26, 52.44, 38.49, 28.31; HRMS-DART (*m*/*z*):[M + H]^+^ calcd for C_19_H_21_F_9_NO_4_: 498.1327, found: 498.1320.
*tert*-Butyl (*S*)-(1-hydroxy-3-(4-(perfluorobutyl)phenyl)propan-2-yl)carbamate (**6a**). Lithium chloride (348.0 mg, 8.21 mmol) and sodium borohydride (312.5 mg, 8.26 mmol) were added to a solution of **5a** (2.03 g, 4.07 mmol) in dry-THF (15 mL) and dry-EtOH (30 mL) and the white suspension was stirred for 23 h. Then, 1 M HCl aq. was added to the reaction mixture until pH = 4. The solution was concentrated. The aqueous layer was extracted with DCM. The organic layer was dried over Na_2_SO_4_, filtered, and concentrated. The crude was purified by silica gel chromatography (ethyl acetate:*n*-hexane = 1:1) to give the desired product **6a** (1.82 g, 3.88 mmol, 95%). White solid; mp 89 °C; ^1^H NMR (400 MHz, CDCl_3_) δ 7.52 (d, *J* = 8.4 Hz, 2H), 7.37 (d, *J* = 8.0 Hz, 2H), 4.76 (d, *J* = 7.6 Hz, 1H), 3.90 (s, 1H), 3.71–3.54 (m, 2H), 2.93 (d, *J* = 6.8 Hz, 2H), 2.15 (s, 1H), 1.39 (s, 9H); ^19^F NMR (376 MHz, CDCl_3_) δ −80.95 (3F), −110.68 (2F), −122.69 (2F), −125.54 (2F); ^13^C NMR (101 MHz, CDCl_3_) δ 155.94, 142.58, 129.67, 127.41, 127.07, 118.99–115.88, 79.96, 64.13, 53.43, 39.49, 37.40, 28.33; HRMS-DART (*m*/*z*):[M + H]^+^ calcd for C_18_H_21_F_9_NO_3_: 470.1378, found: 470.1373.
(*S*)-2-Amino-3-(4-(perfluorobutyl)phenyl)propan-1-ol hydrochloride (**7a**). To a solution of *tert*-butyl (*S*)-(1-hydroxy-3-(4-(perfluorobutyl)phenyl)propan-2-yl)carbamate **6a** (0.85 g, 1.82 mmol) in ethyl acetate (6 mL) was added a conc. HCl (6 mL). The mixture was stirred at room temperature for 35 min. Excess water was separated as the toluene azeotrope. After the addition of Et_2_O, the suspension was purified by filtration to give the desired product **7a** (0.71 g, 1.75 mmol, 95%). White solid; mp 166–167 °C; ^1^H NMR (400 MHz, (CD_3_)_2_SO) δ 8.06 (s, 3H), 7.57 (d, *J* = 8.0 Hz, 2H), 7.50 (d, *J* = 8.0 Hz, 2H), 5.28 (t, *J* = 4.4 Hz, 1H), 3.64–3.43 (m, 2H), 3.10–2.95 (m, 2H); ^19^F NMR (376 MHz, (CD_3_)_2_SO) δ −80.45 (3F), −109.43 (2F), −122.26 (2F), −125.11 (2F); ^13^C NMR (101 MHz, (CD_3_)_2_SO) δ 142.54, 130.75, 127.47, 126.10, 116.43–108.50, 60.17, 53.91, 35.02; HRMS-DART (*m*/*z*):[M − Cl]^+^ calcd for C_13_H_13_F_9_NO: 370.0853, found: 370.0844.
*N*^1^,*N*^3^-Bis((*S*)-1-hydroxy-3-(4-(perfluorobutyl)phenyl)propan-2-yl)-2,2-dimethylmalonamide (**8a**). Under N_2_ atmosphere, to a solution of **7a** (1.39 g, 3.44 mmol) and Et_3_N (1 mL, 7.18 mmol) in dry-DCM (30 mL) was added solution of dimethylmalonyl dichloride (227 μL, 1.72 mmol) in dry-DCM (11 mL) at 0 °C. Then, the reaction temperature rose to room temperature and the mixture was stirred for 20 h. The mixture was concentrated. The crude was purified by silica gel chromatography (MeOH:CHCl_3_ = 1:20) to give the desired product **8a** (1.37 g, 1.64 mmol, 95%). White solid; mp 116–117 °C; ^1^H NMR (270 MHz, CDCl_3_) δ 7.49 (d, *J* = 8.1 Hz, 4H), 7.31 (d, *J* = 8.4 Hz, 4H), 6.49 (d, *J* = 8.4 Hz, 2H), 4.28 (br s, 2H), 3.74 (dd, *J* = 11.3, 3.2 Hz, 2H), 3.47 (dd, *J* = 11.3, 6.5 Hz, 2H), 2.95–2.73 (m, 4H), 1.16 (s, 6H); ^19^F NMR (376 MHz, CDCl_3_) δ −81.02 (6F), −110.81 (4F), −122.77 (4F), −125.54 (4F); ^13^C NMR (101 MHz, CDCl_3_) δ 173.98, 141.95, 129.42, 127.35, 127.01, 121.74–111.20, 64.16, 52.50, 49.78, 36.71, 23.26; HRMS-DART (*m*/*z*):[M + H]^+^ calcd for C_31_H_29_F_18_N_2_O_4_: 835.1840, found: 835.1824.
(4*S*,4′*S*)-2,2′-(Propane-2,2-diyl)bis(4-(4-(perfluorobutyl)benzyl)-4,5-dihydrooxazole) (**1a**). Under N_2_ atmosphere, to a solution of **8a** (444.2 mg, 0.53 mmol) in dry-DCM (30 mL) was added a solution of diethylaminosulfur trifluoride (155 μL, 1.17 mmol) in dry-DCM (15 mL) at −78 °C. After stirring at 0 °C for 11 h, the mixture was washed with saturated aqueous NaHCO_3_ and the aqueous layer was extracted with DCM. The combined organic layers were dried over Na_2_SO_4_ and concentrated. The crude was purified by silica gel chromatography (MeOH:CHCl_3_ = 1:20) to give the desired product **1a** (337.3 mg, 0.42 mmol, 79%). Yellow oil; ^1^H NMR (400 MHz, CDCl_3_) δ 7.50 (d, *J* = 8.4 Hz, 4H), 7.34 (d, *J* = 8.0 Hz, 4H), 4.45–4.39 (m, 2H), 4.22 (t, *J* = 8.8 Hz, 2H), 3.97–3.94 (m, 2H), 3.05–3.00 (m, 2H), 2.85–2.80 (m, 2H), 1.41 (s, 6H); ^19^F NMR (376 MHz, CDCl_3_) δ −81.99 (6F), −110.76 (4F), −122.67 (4F), −125.54 (4F); ^13^C NMR (101 MHz, CDCl_3_) δ 169.70, 141.98, 129.96, 127.13, 126.88, 118.97–108.83, 71.79, 66.43, 41.06, 38.61, 24.08; HRMS-DART (*m*/*z*):[M + H]^+^ calcd for C_31_H_25_F_18_N_2_O: 799.1629, found: 799.1627.
Methyl (*S*)-2-((*tert*-butoxycarbonyl)amino)-3-(4-(perfluorooctyll)phenyl)propanoate (**5b**). To a mixture of **4** (103.3 mg, 0.25 mmol), Cu powder (165.6 mg, 2.61 mmol), 2,2′-bipyridyl (4.0 mg, 0.03 mmol), and DMF (1 mL) was added a 1,1,1,2,2,3,3,4,4,5,5,6,6,7,7,8,8-heptadecafluoro-8-iodooctane (130 μL, 0.49 mmol). The mixture was stirred at 120 °C for 21 h. The mixture was filtered through Celite, and the solids were washed with MeOH. The filtrate was concentrated. Then, 1 M HCl aq. was added to the suspension. The aqueous layer was extracted with ethyl acetate. The organic layer was washed with brine, dried over Na_2_SO_4_, filtered, and concentrated. The crude was purified by silica gel chromatography (ethyl acetate:*n*-hexane = 1:3) to give the desired product **5b** (0.17 g, 0.25 mmol, 97%). White solid; mp 70–71 °C; ^1^H NMR (400 MHz, CDCl_3_) δ 7.52 (d, *J* = 8.4 Hz, 2H), 7.29 (d, *J* = 8.0 Hz, 2H), 5.02 (d, *J* = 7.2 Hz, 1H), 4.66–4.61 (m, 1H), 3.72 (s, 3H), 3.24–3.05 (m, 2H), 1.40 (s, 9H); ^19^F NMR (376 MHz, CDCl_3_) δ −80.83 (3F), −110.51 (2F), −121.17 (2F), −121.77 (6F), −122.63 (2F), −126.03 (2F); ^13^C NMR (101 MHz, CDCl_3_) δ 171.99, 154.99, 140.71, 129.68, 127.71, 127.04, 120.07–100.39, 80.21, 54.21,52.38, 38.46, 28.25; HRMS-DART (*m*/*z*):[M + H]^+^ calcd for C_23_H_21_F_17_NO_4_: 698.1199, found: 698.1188.
*tert*-Butyl (*S*)-(1-hydroxy-3-(4-(perfluorooctyl)phenyl)propan-2-yl)carbamate (**6b**). Lithium chloride (211.3 mg, 4.98 mmol) and sodium borohydride (188.8 mg, 4.99 mmol) were added to a solution of **5b** (1.70 g, 2.43 mmol) in dry-THF (15 mL) and dry-EtOH (30 mL) and the white suspension was stirred for 13 h. Then, 1 M HCl aq. was added to the reaction mixture until pH = 4. The solution was concentrated. The aqueous layer was extracted with DCM. The organic layer was dried over Na_2_SO_4_, filtered, and concentrated. The crude was purified by silica gel chromatography (ethyl acetate:*n*-hexane = 1:2) to give the desired product **6b** (1.50 g, 2.24 mmol, 92%). White solid; mp 186–187 °C; ^1^H NMR (400 MHz, CDCl_3_) δ 7.52 (d, *J* = 8.4 Hz, 2H), 7.37 (d, *J* = 8.0 Hz, 2H), 4.74 (d, *J* = 7.2 Hz, 1H), 3.91 (s, 1H), 3.71–3.55 (m, 2H), 2.93 (d, *J* = 6.8 Hz, 2H), 2.10 (s, 1H), 1.39 (s, 9H); ^19^F NMR (376 MHz, CDCl_3_) δ −80.62 (3F), −110.32 (2F), −121.14 (2F), −121.71 (6F), −122.58 (2F), −125.97 (2F); ^13^C NMR (101 MHz, CDCl_3_) δ 155.95, 142.83, 129.67, 127.27, 127.14, 118.64–108.61, 79.94, 64.04, 53.59, 37.52, 28.57; HRMS-DART (*m*/*z*):[M + H]^+^ calcd for C_22_H_21_F_17_NO_3_: 670.1250, found: 670.1227.
(*S*)-2-Amino-3-(4-(perfluorooctyl)phenyl)propan-1-ol hydrochloride (**7b**). To a solution of *tert*-butyl (*S*)-(1-hydroxy-3-(4-(perfluorooctyl)phenyl)propan-2-yl)carbamate **6b** (1.44 g, 2.15 mmol) in ethyl acetate (8 mL) was added a conc. HCl (8 mL). The mixture was stirred at room temperature for 35 min. Excess water was separated as the toluene azeotrope. After the addition of Et_2_O, the suspension was purified by filtration to give the desired product **7b** (1.28 g, 2.12 mmol, 98%). White solid; mp 98–99 °C; ^1^H NMR (400 MHz, (CD_3_)_2_SO) δ 8.10 (s, 3H), 7.65 (d, *J* = 8.0 Hz, 2H), 7.56 (d, *J* = 8.0 Hz, 2H), 5.41–5.40 (m, 1H), 3.54–3.37 (m, 2H), 3.05–2.92 (m, 2H); ^19^F NMR (376 MHz, (CD_3_)_2_SO) δ −80.13 (3F), −109.22 (2F), −120.95 (2F), −121.33 to −121.58 (m, 6F), −122.37 (2F), −125.63 (2F); ^13^C NMR (101 MHz, (CD_3_)_2_SO) δ 142.47, 130.74, 127.45, 126.47, 122.63–110.16, 60.22, 53.84, 35.08; HRMS-DART (*m*/*z*):[M − Cl]^+^ calcd for C_17_H_13_F_17_NO: 570.0726, found: 570.0722.
*N^1^,N^3^*-bis((*S*)-1-hydroxy-3-(4-(perfluorooctyl)phenyl)propan-2-yl)-2,2-dimethylmalonamide (**8b**). Under N_2_ atmosphere, to a solution of **7b** (1.23 g, 2.04 mmol) and Et_3_N (595 μL, 4.27 mmol) in dry-DCM (12 mL) was added a solution of dimethylmalonyl dichloride (135 μL, 1.02 mmol) in dry-DCM (12 mL) at 0 °C. Then, the reaction temperature rose to room temperature and the mixture was stirred for 20 h. The mixture was concentrated. The crude was purified by silica gel chromatography (MeOH:CHCl_3_ = 1:20) to give the desired product **8b** (1.09 g, 0.88 mmol, 86%). White solid; mp 142 °C; ^1^H NMR (400 MHz, CDCl_3_) δ 7.50 (d, *J* = 8.0 Hz, 4H), 7.32 (d, *J* = 8.4 Hz, 4H), 6.44 (d, *J* = 8.4 Hz, 2H), 4.27–4.24 (m, 2H), 3.76–3.72 (m, 2H), 3.52–3.48 (m, 2H), 2.95–2.90 (m, 2H), 2.84–2.79 (m, 2H), 1.18 (s, 6H); ^19^F NMR (376 MHz, CDCl_3_) δ −80.69 (6F), −110.57 (4F), −121.17 (4F), −121.80 (12F), −122.63 (4F), −126.02 (4F); ^13^C NMR (101 MHz, CD_3_OD) δ 174.38, 143.64, 129.69, 126.46, 126.40, 118.58, 115.99, 115.71, 113.51, 110.92, 110.62, 110.29, 108.27, 63.14, 52.59, 49.79, 36.28, 22.72; HRMS-DART (*m*/*z*):[M + H]^+^ calcd for C_39_H_29_F_34_N_2_O_4_: 1235.1584, found: 1235.1556.
(4*S*,4′*S*)-2,2′-(Propane-2,2-diyl)bis(4-(4-(perfluorohexyl)benzyl)-4,5-dihydrooxazole) (**1b**). The **8b** (673.1 mg, 0.545 mmol) was dissolved in dry-1,2-DCE (110 mL) and heated to 80 °C. Thionyl chloride (4 mL, 5.48 mmol) was added and stirred at 80 °C for 5 h. The reaction mixture was concentrated and quenched with a saturated NaHCO_3_ solution. The mixture was extracted with DCM and the combined organic layers were dried over Na_2_SO_4_ filtered and the solvent was removed under reduced pressure. The residue was dissolved in a solution of NaOH (84.1 mg, 2.10 mmol) in MeOH (153 mL) and heated to 60 °C for 15 h. The solvent was removed under reduced pressure and the resulting residue was partitioned between DCM and H_2_O. The aqueous layer was extracted with DCM. The combined organic layers were dried over Na_2_SO_4_, filtered, and the solvent was removed under reduced pressure. The crude was purified by silica gel chromatography (MeOH:CHCl_3_ = 1:100) to give the desired product **1b** (0.49 g, 0.41 mmol, 75%). White solid; mp 78–79 °C; ^1^H NMR (400 MHz, CDCl_3_) δ 7.50 (d, *J* = 8.4 Hz, 4H), 7.34 (d, *J* = 7.6 Hz, 4H), 4.46–4.39 (m, 2H), 4.24–4.19 (m, 2H), 3.97–3.93 (m, 2H), 3.02 (dd, *J* = 14.0, 5.2 Hz, 2H), 2.83 (dd, *J* = 13.6, 6.8 Hz, 2H), 1.41 (s, 6H); ^19^F NMR (376 MHz, CDCl_3_) δ −80.65 (6F), −110.36 (4F), −121.19 (4F), −121.80 (12F), −122.63 (4F), −126.02 (4F); ^13^C NMR (101 MHz, CDCl_3_) δ 169.62, 141.93, 129.93, 127.18, 126.91, 126.85, 126.79, 121.86–110.25, 71.75, 66.40, 40.95, 38.57, 24.02; HRMS-DART (*m*/*z*):[M + H]^+^ calcd for C_39_H_25_F_34_N_2_O_2_: 1199.1373, found: 1199.1360.
(*S*)-2-((*tert*-Butyldimethylsilyl)oxy)-1-(2-iodophenyl)ethan-1-amine (**10**) [31]. To a suspension of (*S*)-2-amino-2-phenylethan-1-ol **9** (137.2 mg, 1.00 mmol) in THF (4 mL) at −78 °C was added *n*-BuLi (1.6 M solution in hexane, 1.25 mL) dropwise. The resulting purple solution was stirred at −78 °C for 30 min before a solution of TBSCl (*tert*-butyldimethylsilyl chloride) (317 mg, 2.10 mmol) in THF (2 mL) was added at the same temperature. The reaction mixture was allowed to warm to room temperature naturally and was stirred for 18 h. After removing the THF solvent under reduced pressure, the residue was redissolved in ether (5 mL). To this solution at −78 °C was added *n*-BuLi (1.6 M solution in hexane, 1.88 mL) dropwise. The reaction mixture was allowed to slowly warm to room temperature for 3 h and stirred at room temperature for 1 h. I_2_ (508 mg, 2.00 mmol) was added at −78 °C and the reaction mixture was allowed to warm to room temperature and stirred at room temperature for 18 h. Then, 10% Na_2_S_2_O_3_ solution (2 mL) was added and the resulting mixture was stirred vigorously for 10 min. To a suspension was added H_2_O (20 mL). The aqueous layer was extracted with ethyl acetate. The combined organic layers were dried over Na_2_SO_4_, filtered, and the solvent was removed under reduced pressure. The crude was purified by silica gel chromatography (ethyl acetate:*n*-hexane = 1:5) to give the desired product **10** (212 mg, 0.56 mmol, 56%). Brown oil; ^1^H NMR (400 MHz, CDCl_3_) δ 7.80 (dd, *J* = 7.6, 1.2 Hz, 1H), 7.56 (dd, *J* = 7.6, 1.2 Hz, 1H), 7.33 (dt, *J* = 8.0, 1.6 Hz, 1H), 6.94 (dt, *J* = 7.2, 1.2 Hz, 1H), 4.32 (dd, *J* = 7.6, 3.6 Hz, 1H), 3.79 (dd, *J* = 9.6, 3.2 Hz, 1H), 3.41 (dd, *J* = 10.0, 7.6 Hz, 1H), 1.79 (br s, 2H), 0.90 (s, 9H), 0.06 (s, 3H), 0.02 (s, 3H); ^13^C NMR (101 MHz, CDCl_3_) δ 144.40, 139.47, 129.11, 128.37, 128.30, 99.83, 67.53, 60.86, 26.04, 18.39, −5.14, −5.23; HRMS-DART (*m*/*z*):[M + H]^+^ calcd for C_14_H_25_INOSi: 378.0745, found: 378.0742.
*N*^1^,*N*^3^-Bis((*S*)-2-Hydroxy-1-(2-iodophenyl)ethyl)-2,2-dimethylmalonamide (**11**). Under N_2_ atmosphere, to a solution of **10** (201 mg, 0.533 mmol) and Et_3_N (77 μL, 0.558 mmol) in dry-THF (2 mL) was added dimethylmalonyl dichloride (34 μL, 0.254 mmol) at 0 °C. Then, the reaction temperature rose to room temperature and the mixture was stirred for 18 h. To the mixture was added TBAF (1 M solution in THF, 553 μL). The mixture was stirred at room temperature for 16 h. To a suspension was added saturated aqueous NH_4_Cl (10 mL). The aqueous layer was extracted with ethyl acetate. The combined organic layers were dried over Na_2_SO_4_, filtered, and the solvent was removed under reduced pressure. The crude was purified by silica gel chromatography (MeOH:CHCl_3_ = 1:20) to give the desired product **11** (125 mg, 0.201 mmol, 79%). White solid; mp 75–76 °C; ^1^H NMR (400 MHz, CDCl_3_) δ 7.86 (dd, *J* = 8.4, 1.2 Hz, 2H), 7.27–7.23 (m, 8H), 7.15 (dd, *J* = 7.6, 1.2 Hz, 2H), 6.97 (dt, *J* = 7.6, 1.6 Hz, 2H), 5.33–5.28 (m, 2H), 3.94 (dd, *J* = 12.0, 4.4 Hz, 2H), 3.79 (dd, *J* = 11.6, 6.4 Hz, 2H); ^13^C NMR (101 MHz, CDCl_3_) δ 173.79, 140.77, 140.21, 129.62, 128.69, 127.78, 99.20, 64.59, 59.70, 50.00, 23.86; HRMS-DART (*m*/*z*):[M + H]^+^ calcd for C_21_H_25_I_2_N_2_O_4_: 622.9904, found: 622.9904.
(4*S*,4′*S*)-2,2′-(Propane-2,2-diyl)bis(4-(2-iodophenyl)-4,5-dihydrooxazole) (**12**). Under N_2_ atmosphere, to a solution of **11** (1.92 g, 3.082 mmol) in dry-DCM (20 mL) was added a solution of diethylaminosulfur trifluoride (888 μL, 6.78 mmol) in dry-DCM (10 mL) at −78 °C. After stirring at 0 °C for 18 h, the mixture was washed with saturated aqueous NaHCO_3_ and the aqueous layer was extracted with DCM. The combined organic layers were dried over Na_2_SO_4_ and concentrated. The crude was purified by silica gel chromatography (ethyl acetate:*n*-hexane = 1:5) to give the desired product **12** (1.25 g, 2.217 mmol, 69%). Yellow solid; mp 135–136 °C; ^1^H NMR (400 MHz, CDCl_3_) δ 7.82 (d, *J* = 8.4 Hz, 2H), 7.32 (d, *J* = 4.0 Hz, 4H), 7.00–6.96 (m, 2H), 5.49 (dd, *J* = 10.4, 7.6 Hz, 2H), 4.87 (dd, *J* = 10.0, 8.4 Hz, 2H), 3.98 (t, *J* = 8.0 Hz, 2H), 1.72 (s, 6H); ^13^C NMR (101 MHz, CDCl_3_) δ 171.31, 145.54, 139.13, 129.28, 128.70, 127.68, 98.14, 74.95, 72.86, 39.41, 24.53; HRMS-DART (*m*/*z*):[M + H]^+^ calcd for C_21_H_21_I_2_N_2_O_2_: 586.9692, found: 586.9687.
(4*S*,4′*S*)-2,2′-(Propane-2,2-diyl)bis(4-(2-(perfluorobutyl)phenyl)-4,5-dihydrooxazole) (**1c**). To a mixture of **12** (50 mg, 0.0853 mmol), Cu powder (54.2 mg, 0.853 mmol), 2,2′-bipyridyl (1.3 mg, 0.00853 mmoml), and DMF (1 mL) was added a 1,1,1,2,2,3,3,4,4-nonafluoro-4-iodobutane (29 μL, 0.171 mmol). The mixture was stirred at 120 °C for 12 h. The mixture was filtered through Celite, and the solids were washed with MeOH. The filtrate was concentrated. Then, 1 M HCl aq. was added to the suspension. The aqueous layer was extracted with ethyl acetate. The organic layer was washed with brine, dried over Na_2_SO_4_, filtered, and concentrated. The crude was purified by silica gel chromatography (ethyl acetate:*n*-hexane = 1:5) to give the desired product **1c** (44.7 mg, 0.0580 mmol, 68%). White solid; mp 89–90 °C; ^1^H NMR (400 MHz, CDCl_3_) δ 7.58–7.50 (m, 6H), 7.43–7.39 (m, 2H), 5.64–5.58 (m, 2H), 4.70 (t, *J* = 8.8 Hz, 2H), 4.02 (t, *J* = 8.4 Hz, 2H), 1.74 (s, 6H); ^19^F NMR (376 MHz, CDCl_3_) δ −80.81 (6F), −102.04 to −105.02 (m, 4F), −121.34 (4F), −125.54 (4F); ^13^C NMR (101 MHz, CDCl_3_) δ 171.45, 142.55, 132.86, 128.37, 128.28, 127.62, 125.49, 117.35, 116.00, 112.30, 111.79, 76.16, 66.09, 39.27, 24.47; HRMS-DART (*m*/*z*):[M + H]^+^ calcd for C_29_H_21_F_18_N_2_O_2_: 771.1316, found: 771.1308.
(4*S*,4′*S*)-2,2′-(Propane-2,2-diyl)bis(4-(2-(perfluorooctyl)phenyl)-4,5-dihydrooxazole) (**1d**). To a mixture of **12** (80 mg, 0.136 mmol), Cu powder (86.7 mg, 1.365 mmol), 2,2′-bipyridyl (2.1 mg, 0.0136 mmol), and DMF (1 mL) was added a 1,1,1,2,2,3,3,4,4,5,5,6,6,7,7,8,8-heptadecafluoro-8-iodooctane (72 μL, 0.273 mmol). The mixture was stirred at 120 °C for 18 h. The mixture was filtered through Celite, and the solids were washed with MeOH. The filtrate was concentrated. Then, 1 M HCl aq. was added to the suspension. The aqueous layer was extracted with ethyl acetate. The organic layer was washed with brine, dried over Na_2_SO_4_, filtered, and concentrated. The crude was purified by silica gel chromatography (ethyl acetate:*n*-hexane = 1:5) to give the desired product **1d** (62.9 mg, 0.0537 mmol, 39%). White solid; mp 108–109 °C; ^1^H NMR (400 MHz, CDCl_3_) δ 7.57–7.50 (m, 6H), 7.43–7.39 (m, 2H), 5.65–5.59 (m, 2H), 4.70 (t, *J* = 8.4 Hz, 2H), 4.02 (t, *J* = 8.4 Hz, 2H), 1.74 (s, 6H); ^19^F NMR (376 MHz, CDCl_3_) δ −80.59 (6F), −101.81 to −104.80 (m, 4F), −120.35 (4F), −121.15 (4F), −121.61 to −121.76 (m, 8F), −122.58 (4F), −125.97 (4F); ^13^C NMR (101 MHz, CDCl_3_) δ 171.45, 142.53, 132.85, 128.39, 128.28, 127.61, 125.60, 121.25–107.94, 76.16, 66.08, 39.28, 24.46; HRMS-DART (*m*/*z*):[M + H]^+^ calcd for C_37_H_21_F_34_N_2_O_2_: 1171.1060, found: 1171.1079.
*N*^1^,*N*^3^-Bis((*S*)-2-Hydroxy-1-phenylethyl)-2,2-dimethylmalonamide (**13**) [27]. Under N_2_ atmosphere, to a solution of **9** (600 mg, 4.374 mmol) and Et_3_N (635 μL, 4.582 mmol) in dry-DCM (6 mL) was added dimethylmalonyl dichloride (275 μL, 2.083 mmol) at 0 °C. Then, the reaction temperature rose to room temperature and the mixture was stirred for 18 h. The reaction mixture was concentrated. The crude was purified by silica gel chromatography (MeOH:CHCl_3_ = 1:5) to give the desired product **13** (765 mg, 2.064 mmol, 99%). Yellow solid; mp 128–130 °C; ^1^H NMR (400 MHz, CDCl_3_) δ 7.35–7.22 (m, 10H), 7.10 (d, *J* = 7.6 Hz, 2H), 5.14–5.10 (m, 2H), 3.96–3.90 (m, 2H), 3.83–3.77 (m, 2H), 2.79–2.76 (m, 2H), 1.61 (s, 6H).
(4*S*,*4*′S)-2,2′-(Propane-2,2-diyl)bis(4-phenyl-4,5-dihydrooxazole) (**14**) [32]. To a solution of **13** (765 mg, 2.065 mmol), DMAP (25.2 mg, 0.207 mmol) and Et_3_N (1.46 mL, 10.532 mmol) in DCM (8 mL) was added tosyl chloride (826.7 mg, 4.337 mmol). After stirring at room temperature for 36 h, the mixture was washed with saturated aqueous NH_4_Cl and the aqueous layer was extracted with DCM. The combined organic layers were washed with saturated aqueous NaHCO_3_; the aqueous layer was extracted with DCM. The combined organic layers were dried over Na_2_SO_4_ and concentrated. The crude was purified by silica gel chromatography (ethyl acetate:*n*-hexane = 1:1) to give the desired product **14** (424.3 mg, 1.269 mmol, 61%). Colorless oil; ^1^H NMR (400 MHz, CDCl_3_) δ 7.34–7.26 (m, 10H), 5.26–5.21 (m, 2H), 4.70–4.65 (m, 2H), 4.19–4.15 (m, 2H), 1.68 (s, 6H).
*tert*-Butyl (*S*)-(2-((*tert*-butyldimethylsilyl)oxy)-1-(2-iodophenyl)ethyl)carbamate (**15**). A solution of **10** (639.7 mg, 1.695 mmol) and Et_3_N (285 μL, 2.048 mmol) in dry-DCM (8 mL) was stirred at 0 °C. A solution of Boc_2_O (449.2 mg, 2.058 mmol) in dry-DCM (4 mL) was slowly added to the mixture. Then the reaction temperature rose to room temperature and the mixture was stirred for a further 20 h. The mixture was diluted with brine. The reaction mixture was extracted with DCM. The organic layer was dried over Na_2_SO_4_, filtered, and concentrated. The crude was purified by silica gel chromatography (ethyl acetate:*n*-hexane = 1:5) to give the desired product **15** (637 mg, 1.424 mmol, 84%). Yellow oil; ^1^H NMR (400 MHz, CDCl_3_) δ 7.81 (d, *J* = 7.6 Hz, 1H), 7.32–7.29 (m, 2H), 6.96–6.92 (m, 1H), 5.51 (br s, 1H), 4.94 (br s, 1H), 3.88–3.71 (m, 2H), 1.43 (br s, 9H), 0.83 (s, 9H), −0.08 (s, 3H), −0.14 (s, 3H); ^13^C NMR (101 MHz, CDCl_3_) δ 155.32, 142.31, 139.61, 129.02, 128.12, 127.97, 98.49, 79.73, 64.68, 59.64, 28.42, 25.90, 18.33, −5.55; HRMS-DART (*m*/*z*):[M + H]^+^ calcd for C_19_H_33_INO_3_Si: 478.1274, found: 478.1271.
*tert*-Butyl (*S*)-(2-((*tert*-butyldimethylsilyl)oxy)-1-(2-butylphenyl)ethyl)carbamate (**16**). To a mixture of K_3_PO_4_ (216.4 mg, 1.019 mmol), toluene (15 mL), **15** (60.8 mg, 0.127 mmol), and butylboronic acid (68.2 mg, 0.669 mmol) was added Pd(OAc)_2_ (9.2 mg, 0.0410 mmol) and SPhos (19.7 mg, 0.0480 mmol). The mixture was stirred at 100 °C for 22 h. The mixture was filtered through Celite, and the solids were washed with MeOH. The filtrate was concentrated. The saturated aqueous NH_4_Cl was added to the suspension. The aqueous layer was extracted with ethyl acetate. The organic layer was washed with brine, dried over Na_2_SO_4_, filtered, and concentrated. The crude was purified by silica gel chromatography (ethyl acetate:*n*-hexane = 1:10) to give the desired product **16** (26.8 mg, 0.0657 mmol, 52%). Yellow oil; ^1^H NMR (400 MHz, CDCl_3_) δ 7.31–7.27 (m, 1H), 7.18–7.13 (m, 3H), 5.14–4.99 (m, 2H), 3.85–3.81 (m, 1H), 3.69–3.67 (m, 1H), 2.70–2.66 (m, 2H), 1.68–1.37 (m, 13H), 0.95 (t, *J* = 7.6 Hz, 3H), 0.85 (s, 9H), −0.055 to −0.058 (m, 6H); ^13^C NMR (101 MHz, CDCl_3_) δ 155.39, 140.31, 138.28, 129.43, 127.14, 126.41, 125.84, 79.23, 66.15, 60.40, 33.69, 32.35, 28.46, 25.77, 22.94, 18.26, 13.92, −5.45, −5.56; HRMS-DART (*m*/*z*):[M + H]^+^ calcd for C_23_H_42_NO_3_Si: 408.2934, found: 408.2933.
(*S*)-2-Amino-2-(2-butylphenyl)ethan-1-ol (**17**). To **16** (59.3 mg, 0.145 mmol) was added 4 M HCl in AcOEt (2 mL). The mixture was stirred at room temperature for 2 h. The reaction mixture was concentrated. Then, 4 M NaOH aq. was added to the residue. The aqueous layer was extracted with ethyl acetate. The organic layer was dried over Na_2_SO_4_, filtered, and concentrated. The crude was purified by silica gel chromatography (MeOH:CHCl_3_ = 1:5) to give the desired product **17** (14.6 mg, 0.0755 mmol, 52%). Yellow oil; ^1^H NMR (400 MHz, CDCl_3_) δ 7.38–7.36 (m, 1H), 7.24–7.16 (m, 3H), 4.34–4.30 (m, 1H), 3.69 (dd, *J* = 10.8, 4.4 Hz, 1H), 3.56 (dd, *J* = 10.8, 8.8 Hz, 1H), 2.74–2.59 (m, 2H), 1.60–1.53 (m, 2H), 1.45–1.36 (m, 2H), 0.95 (t, *J* = 7.2 Hz, 3H); ^13^C NMR (101 MHz, CDCl_3_) δ 140.30, 140.07, 129.82, 127.37, 126.47, 125.59, 67.52, 52.43, 34.16, 32.54, 22.85, 14.07; HRMS-DART (*m*/*z*):[M + H]^+^ calcd for C_12_H_20_NO: 194.1545, found: 194.1540.
*N*^1^,*N*^3^-Bis((*S*)-1-(2-butylphenyl)-2-hydroxyethyl)-2,2-dimethylmalonamide (**18**). Under N_2_ atmosphere, to a solution of **17** (17 mg, 0.088 mmol) and Et_3_N (13 μL, 0.097 mmol) in dry-DCM (2 mL) was added dimethylmalonyl dichloride (5.8 μL, 0.044 mmol) at 0 °C. Then, the reaction temperature rose to room temperature and the mixture was stirred for 3 h. The reaction mixture was concentrated. The crude was purified by silica gel chromatography (MeOH:CHCl_3_ = 1:20) to give the desired product **18** (19.4 mg, 0.040 mmol, 91%). Yellow oil; ^1^H NMR (400 MHz, CDCl_3_) δ 7.22–7.17 (m, 10H), 6.96 (d, *J* = 7.6 Hz, 2H), 5.41–5.36 (m, 2H), 3.89–3.76 (m, 4H), 2.70–2.66 (m, 4H), 1.66–1.37 (m, 14H), 0.94 (t, *J* = 7.2 Hz, 6H); ^13^C NMR (101 MHz, CDCl_3_) δ 174.08, 140.75, 136.18, 130.11, 127.96, 126.53, 125.66, 66.54, 52.01, 49.89, 33.59, 32.50, 23.67, 22.79, 14.06; HRMS-DART (*m*/*z*):[M + H]^+^ calcd for C_29_H_43_N_2_O_4_: 483.3223, found: 483.3221.
(4*S*,4′*S*)-2,2′-(Propane-2,2-diyl)bis(4-(2-butylphenyl)-4,5-dihydrooxazole) (**19**). To a solution of **18** (18.8 mg, 0.039 mmol), DMAP (1.4 mg, 0.0117 mmol) and Et_3_N (27 μL, 0.195 mmol) in DCM (0.5 mL) was added tosyl chloride (22.3 mg, 0.117 mmol). After stirring at room temperature for 24 h, the mixture was diluted with DCM. The mixture was washed with saturated aqueous NH_4_Cl and saturated aqueous NaHCO_3_. The organic layer was dried over Na_2_SO_4_ and concentrated. The crude was purified by silica gel chromatography (ethyl acetate:*n*-hexane = 1:5) to give the desired product **19** (6.2 mg, 0.0139 mmol, 36%). Colorless oil; ^1^H NMR (400 MHz, CDCl_3_) δ 7.29–7.13 (m, 8H), 6.96 (d, *J* = 7.6 Hz, 2H), 5.53–5.48 (m, 2H), 4.75–4.70 (m, 2H), 4.04–4.00 (m, 2H), 2.66–2.53 (m, 4H), 1.72 (s, 6H), 1.59–1.34 (m, 8H), 0.94 (t, *J* = 7.2 Hz, 6H); ^13^C NMR (101 MHz, CDCl_3_) δ 170.47, 140.39, 139.41, 129.24, 127.30, 126.66, 126.32, 75.55, 65.65, 39.14, 33.68, 32.78, 24.64, 22.83, 14.05; HRMS-DART (*m*/*z*):[M + H]^+^ calcd for C_29_H_39_N_2_O_2_: 447.3012, found: 447.3008.
*tert*-Butyl (*S*)-(2-((*tert*-butyldimethylsilyl)oxy)-1-(2-(perfluorobutyl)phenyl)ethyl)carbamate (**20**). To a mixture of **15** (3.5 g, 7.33 mmol), Cu powder (4.66 g, 73.31 mmol), 2,2′-bipyridyl (144.5 mg, 0.733 mmol), and DMF (10 mL) was added a 1,1,1,2,2,3,3,4,4-nonafluoro-4-iodobutane (2.46 mL, 14.661 mmol). The mixture was stirred at 120 °C for 16 h. The mixture was filtered through Celite, and the solids were washed with MeOH. The filtrate was concentrated. Then, 1 M HCl aq. was added to the suspension. The aqueous layer was extracted with ethyl acetate. The organic layer was washed with brine, dried over Na_2_SO_4_, filtered, and concentrated. The crude was purified by silica gel chromatography (ethyl acetate:*n*-hexane = 1:10) to give the desired product **20** (2.0 g, 3.51 mmol, 48%). Yellow oil; ^1^H NMR (400 MHz, CDCl_3_) δ 7.62–7.51 (m, 3H), 7.39 (t, *J* = 7.6 Hz,1H), 5.34 (br s, 1H), 5.06 (br s, 1H), 3.94–3.90 (m, 1H), 3.64–3.62 (m, 1H), 1.37 (br s, 9H), 0.83 (s, 9H), −0.09 (s, 3H), −0.12 (s, 3H); ^19^F NMR (376 MHz, CDCl_3_) δ −80.75 (3F), −101.78 to −104.10 (m, 2F), −120.90 (2F), −125.42 (2F); ^13^C NMR (101 MHz, CDCl_3_) δ 159.41, 141.39, 136.25, 131.69, 131.69, 128.86, 128.72, 127.32, 119.91, 119.06, 117.73, 116.18, 77.29, 76.34, 66.67, 28.32, 25.85, 18.31, −5.65, −5.76; HRMS-DART (*m*/*z*):[M + H]^+^ calcd for C_23_H_33_F_9_NO_3_Si: 570.2086, found: 570.2088.
(*S*)-2-Amino-2-(2-(perfluorobutyl)phenyl)ethan-1-ol (**21**). To **20** (2 g, 3.51 mmol) was added 4 M HCl in AcOEt (17.5 mL). The mixture was stirred at room temperature for 30 min. The reaction mixture was concentrated. Then, 4 M NaOH aq. was added to the residue. The aqueous layer was extracted with ethyl acetate. The organic layer was dried over Na_2_SO_4_, filtered, and concentrated. The crude was purified by silica gel chromatography (MeOH:CHCl_3_ = 1:10) to give the desired product **21** (760.7 mg, 2.14 mmol, 61%). Yellow solid; mp 70–72 °C; ^1^H NMR (400 MHz, CDCl_3_) δ 7.72 (d, *J* = 8.0 Hz, 1H), 7.61–7.56 (m, 2H), 7.42 (t, *J* = 7.4 Hz, 1H), 4.42–4.38 (m, 1H), 3.75–3.71 (m, 1H), 36.0–3.55 (m, 1H), 1.78 (br s, 3H); ^19^F NMR (376 MHz, CDCl_3_) δ −80.88 (3F), −102.09 to −104.25 (m, 2F), −120.61 to −122.47 (m, 2F), −125.54 (2F); ^13^C NMR (101 MHz, CDCl_3_) δ 143.70, 132.47, 128.54, 128.15, 127.57, 126.01, 119.97, 118.97, 117.39, 116.11, 67.66, 52.80; HRMS-DART (*m*/*z*):[M + H]^+^ calcd for C_12_H_11_F_9_NO: 356.0697, found: 356.0695.
*N*^2^,*N*^6^-Bis((*S*)-2-hydroxy-1-(2-(perfluorobutyl)phenyl)ethyl)pyridine-2,6-dicarboxamide (**22**). Under N_2_ atmosphere, to a solution of **21** (400 mg, 1.126 mmol) and Et_3_N (157 μL, 1.126 mmol) in dry-DCM (5mL) was added pyridine-2,6-dicarbonyl dichloride (114.9 mg, 0.563 mmol) at 0 °C. Then, the reaction temperature rose to room temperature, stirring the mixture for 3 h. The reaction mixture was concentrated. The crude was purified by silica gel chromatography (MeOH:CHCl_3_ = 1:10) to give the desired product **22** (445.3 mg, 0.529 mmol, 94%). White solid; mp 95–96 °C; ^1^H NMR (400 MHz, CDCl_3_) δ 8.77 (d, *J* = 6.4 Hz, 2H), 8.33–8.29 (m, 2H), 8.04–8.00 (m, 1H), 7.69–7.44 (m, 8H), 5.54 (br s, 2H), 4.10–4.06 (m, 2H), 3.98–3.94 (m, 2H); ^19^F NMR (376 MHz, CDCl_3_) δ −80.67 (6F), −101.70 to −104.28 (m, 4F), −121.01 (4F), −125.34 (4F); ^13^C NMR (101 MHz, CDCl_3_) δ 163.13, 148.50, 139.60, 139.54, 132.50, 129.46, 128.13, 128.06, 126.26, 125.21, 119.78, 118.98, 117.90, 116.40, 66.75, 52.48; HRMS-DART (*m*/*z*):[M + H]^+^ calcd for C_31_H_22_F_18_N_3_O_4_: 842.1323, found: 842.1326.
2,6-Bis((*S*)-4-(2-(perfluorobutyl)phenyl)-4,5-dihydrooxazol-2-yl)pyridine (**1e**). Under N_2_ atmosphere, to a solution of **22** (416.7 mg, 0.495 mmol) in dry-DCM (5 mL) was added a solution of diethylaminosulfur trifluoride (195 μL, 1.486 mmol) in dry-DCM (2 mL) at −78 °C. After stirring at 0 °C for 16 h, the mixture was washed with saturated aqueous NaHCO_3_ and the aqueous layer was extracted with DCM. The combined organic layers were Na_2_SO_4_ and concentrate. The crude was purified by silica gel chromatography (ethyl acetate:*n*-hexane = 1:3) to give the desired product **1e** (350.9 mg, 0.436 mmol, 88%). Yellow solid; mp 63–64 °C; ^1^H NMR (400 MHz, CDCl_3_) δ 8.42 (d, *J* = 7.6 Hz, 2H), 7.99 (t, *J* = 8.0 Hz, 1H), 7.61–743 (m, 8H), 5.85–5.79 (m, 2H), 4.94–4.88 (m, 2H), 4.29–4.25 (m, 2H); ^19^F NMR (376 MHz, CDCl_3_) δ −80.80 (6F), −102.22 to −104.85 (m, 4F), −121.34 (4F), −125.54 (4F); ^13^C NMR (101 MHz, CDCl_3_) δ 164.60, 146.72, 141.96, 141.92, 137.75, 132.90, 128.52, 127.90, 126.66, 125.65, 119.88–116.11, 76.44, 66.88; HRMS-DART (*m*/*z*):[M + H]^+^ calcd for C_31_H_18_F_18_N_3_O_2_: 806.1112, found: 806.1113.
*N*^2^,*N*^6^-Bis((*S*)-2-hydroxy-1-phenylethyl)pyridine-2,6-dicarboxamide (**23**) [33]. Under N_2_ atmosphere, to a solution of **9** (823 mg, 6.0 mmol) and Et_3_N (836 μL, 6.0 mmol) in dry-DCM (5 mL) was added pyridine-2,6-dicarbonyl dichloride (612 mg, 3.0 mmol) at 0 °C. Then, the reaction temperature rose to room temperature, stirring the mixture for 6 h. The reaction mixture was concentrated. The crude was purified by silica gel chromatography (MeOH:CHCl_3_ = 1:5) to give the desired product **23** (1.13 g, 2.79 mmol, 93%). White solid; mp 79–80 °C; ^1^H NMR (400 MHz, CDCl_3_) δ 8.71 (d, *J* = 7.2 Hz, 2H), 8.16 (d, *J* = 8.0 Hz, 2H), 7.83 (t, *J* = 7.6 Hz, 1H), 7.35–7.24 (m, 10H), 5.21–5.17 (m, 2H), 3.92–3.91 (m, 2H), 3.52 (br s, 2H).
2,6-Bis((*S*)-4-phenyl-4,5-dihydrooxazol-2-yl)pyridine (**24**) [33]. To a solution of **23** (1 g, 2.466 mmol), DMAP (60 mg, 0.493 mmol) and Et_3_N (1.37 mL, 9.866 mmol) in DCM (0.5 mL) was added tosyl chloride (1.18 g, 6.166 mmol). After stirring at room temperature for 18 h, water was added to the mixture. The aqueous layer was extracted with DCM. The combined organic layers were dried over Na_2_SO_4_ and concentrated. The crude was purified by silica gel chromatography (ethyl acetate:*n*-hexane = 1:1) to give the desired product **24** (801.8 mg, 2.170 mmol, 88%). White solid; mp 169 °C; ^1^H NMR (400 MHz, CDCl_3_) δ 8.35 (d, *J* = 8.4 Hz, 2H), 7.92 (t, *J* = 7.6 Hz, 1H), 7.39–7.29 (m, 10H), 5.49–5.44 (m, 2H), 4.96–4.91 (m, 2H), 4.45–4.41 (m, 2H).

## 4. Conclusions

In asymmetric Henry reactions, using a ligand featuring dual fluorous tags strategically positioned adjacent to the chiral ligand effectively reverses the stereoselectivity of the resulting product. While the degree of stereoselectivity in the aldol product does not surpass that of other catalytic systems reported in the literature [34,35], the introduction of fluorous tags at the 2′-aryl position of the BOX ligand can dynamically modify the asymmetric environment in the transition state of this reaction. Future work will involve the application of this approach to asymmetric synthesis reactions using other asymmetric transition metal complexes.

## Figures and Tables

**Table 1:**
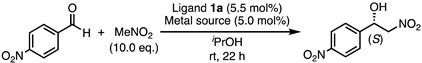
Asymmetric Henry reaction using fluorous bisoxazoline **1a**.

Entry	Metal Source	Conv. (%) ^1^	*ee* (%) ^2,3^
1	Cu(OAc)_2_·H_2_O	97	69
2	CuCl_2_	no reaction	-
3	CuBr_2_	no reaction	-
4	Cu(OTf)_2_	no reaction	-
5	Co(OAc)_2_	95	9
6	Zn(OAc)_2_	96	3

^1^ Determined by ^1^H Nuclear magnetic resonance (NMR) of the crude product. ^2^ Determined by chiral high-performance liquid chromatography (HPLC) (Chiralpak IA-3 column, hex:*^i^*PrOH = 80:20, 1.0 mL/min). ^3^ The absolute configuration of the aldol adduct was determined by comparison with previously published data [13].

**Table 2:**
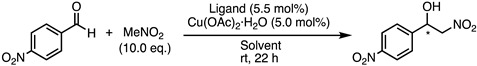
Asymmetric Henry reaction using fluorous ligands **1a**–**d** and control ligand **14**.

Entry	Ligand	Solvent	Conv. (%) ^1^	*ee* (%) ^2^	Config. ^3^
1	**1a**	*^i^*PrOH	97	69	*S*
2	**1a**	*^i^*PrOH/H_2_O (1/1)	94	41	*S*
3	**1a**	THF	79	41	*S*
4	**1a**	FC-72	93	45	*S*
5	**1b**	*^i^*PrOH	91	58	*S*
6	**1b**	*^i^*PrOH/H_2_O (1/1)	87	44	*S*
7	**1b**	FC-72	14	33	*S*
8	**1c**	*^i^*PrOH	94	53	*R*
9	**1c**	*^i^*PrOH/H_2_O (1/1)	95	36	*R*
10	**1c**	THF	45	42	*R*
11	**1c**	FC-72	95	32	*R*
12	**1d**	*^i^*PrOH	98	47	*R*
13	**1d**	THF	37	35	*R*
14	**14**	*^i^*PrOH	98	27	*S*
15	**19**	*^i^*PrOH	98	14	*R*

^1^ Determined by ^1^H Nuclear magnetic resonance (NMR) of the crude product. ^2^ Determined by chiral high-performance liquid chromatography (HPLC) (Chiralpak IA-3 column, hex:*^i^*PrOH = 80:20, 1.0 mL/min). ^3^ The absolute configuration of the aldol adduct was determined through comparison with previously published data [13]. * Represents the asymmetric carbon.

**Table 3:**
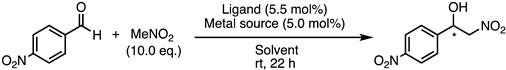
Asymmetric Henry reaction using pincer-type fluorous ligand **1e** and control ligand **24**.

Entry	Ligand	Metal Source	Solvent	Conv. (%) ^1^	*ee* (%) ^2^	Config. ^3^
1	**1e**	Co(OAc)_2_	*^i^*PrOH	97	25	*R*
2	**1e**	Cu(OAc)_2_·H_2_O	*^i^*PrOH	98	10	*R*
3	**1e**	Zn(OAc)_2_	*^i^*PrOH	94	13	*R*
4	**1e**	Co(OAc)_2_	*^i^*PrOH/H_2_O (1/1)	96	2	*R*
5	**1e**	Co(OAc)_2_	THF	72	42	*R*
6	**1e**	Co(OAc)_2_	FC-72	no reaction	-	-
7	**24**	Co(OAc)_2_	THF	99	8	*R*

^1^ Determined by ^1^H Nuclear magnetic resonance (NMR) of the crude product. ^2^ Determined by chiral high-performance liquid chromatography (HPLC) (Chiralpak IA-3 column, hex:*^i^*PrOH = 80:20, 1.0 mL/min). ^3^ The absolute configuration of the aldol adduct was determined through comparison with previously published data [13]. * Represents the asymmetric carbon.

## Data Availability

All data from this study are reported in the text or in Supplementary Materials.

## Supplementary Materials

The following supporting information can be downloaded at: https://www.mdpi.com/article/10.3390/molecules28227632/s1, Copies of ^1^H NMR spectra and HPLC chromatograms of asymmetric Henry reaction; Copies of ^1^H NMR, ^19^F NMR, ^13^C NMR spectra of compounds (**1**, **3**–**8**, **10**–**24)** can be found in Supplementary Materials.
