# Supplementary material for: Asymmetric Henry Reaction Using Cobalt Complexes with Bisoxazoline Ligands Bearing Two Fluorous Tags
Source: Molecules. 2023 Nov 16;28(22):7632. doi: 10.3390/molecules28227632 (PMC10675312; doi:10.3390/molecules28227632)

Asymmetric Henry Reaction Using Cobalt Complexes with Bisoxazoline Ligands Bearing Two Fluorous Tags

Kazuki Ishihara, Yamato Kato, Narisa Takeuci, Yuka Hayashi, Yuna Hagiwara, Shyota Shibuya, Tohya Natsume, and Masato Matsugi*

Faculty of Agriculture, Meijo University, 1-501 Shiogamaguchi, Tempaku-ku, Nagoya 468-8502, Japan

* Correspondence: matsugi@meijo-u.ac.jp

Table of Contents

1. ^{1}H NMR spectra and HPLC chromatograms of Asymmetric Henry Reaction……S2–S25

2. ^{1}H NMR, ^{19}F NMR, ^{13}C NMR spectra of compounds (1, 3–8, 10–24) …………S26–S96

1. ^{1}H NMR spectra and HPLC chromatograms of Asymmetric Henry Reaction

Table 1, entry 1

^{1}H NMR, 400MHz, $CDCl_3$

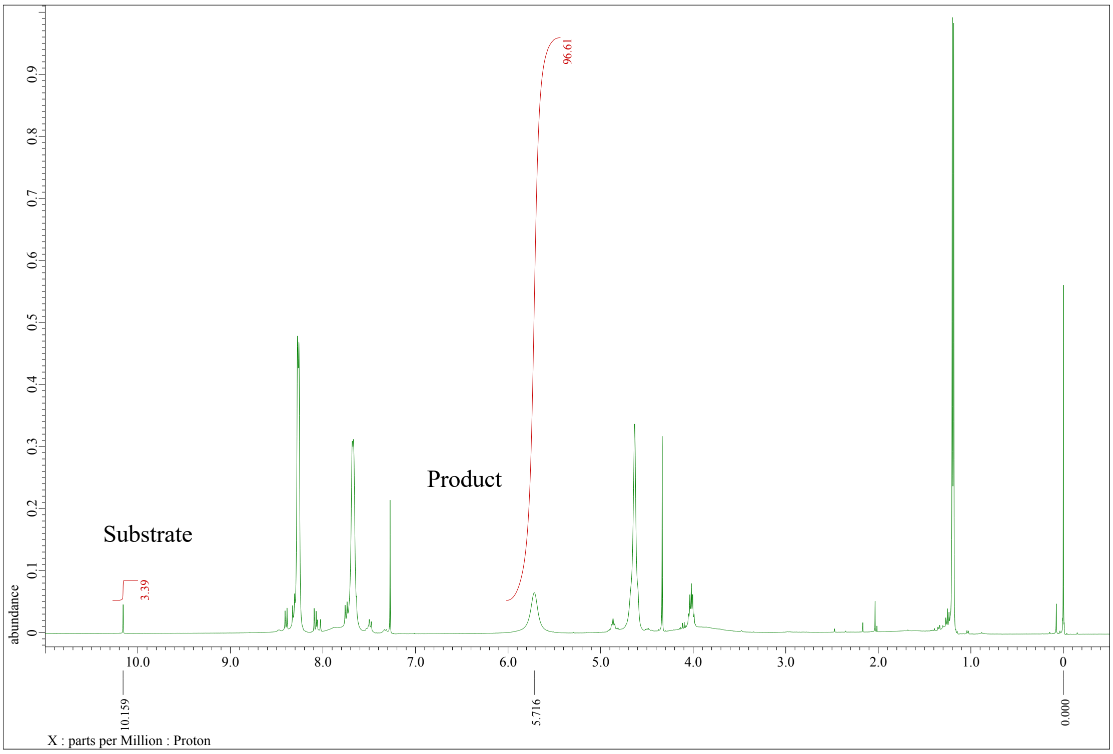

HPLC analysis

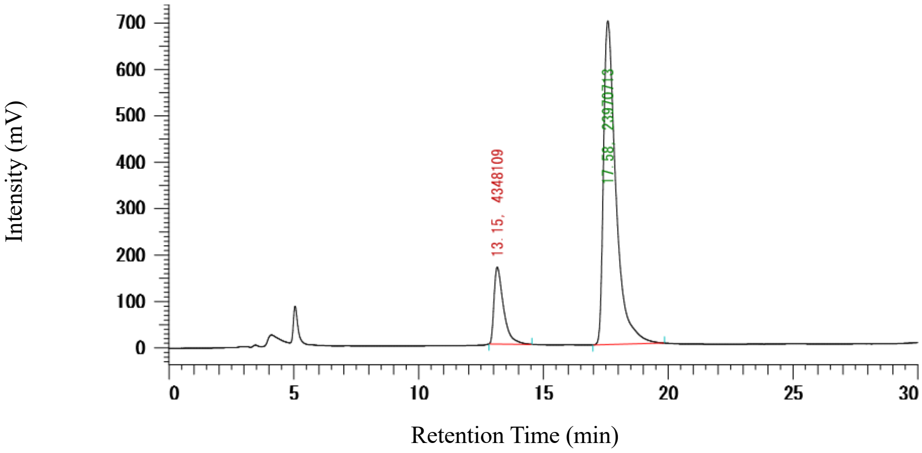

NO	RT	Area	Concentration	BC
1	13.15	4348109	15.354	MC
2	17.58	23970713	84.646	BB
		28318822	100.000	

Conv.: 97%, 69% *ee*, The *ee* of product was determined by HPLC. [205 nm, Daicel chiralpack IA-3 column, hex:^{i}PrOH = 80:20, flow rate 1.0 mL/min, t_R = 17.499 min (major), t_R = 13.305 min (minor)]

HPLC analysis of racemic compound

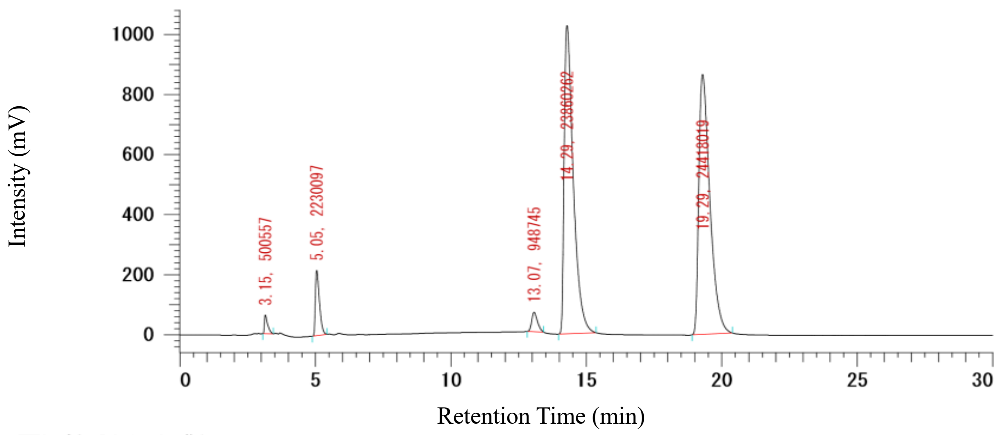

NO	RT	Area	Concentration	BC
1	3. 15	500557	0. 963	MC
2	5. 05	2230097	4. 292	MC
3	13. 07	948745	1. 826	MC
4	14. 29	23860262	45. 922	MC
5	19. 29	24418019	46. 996	MC
		51957680	100. 000	

Table 1, entry 5

^{1}H NMR, 400MHz, $CDCl_3$

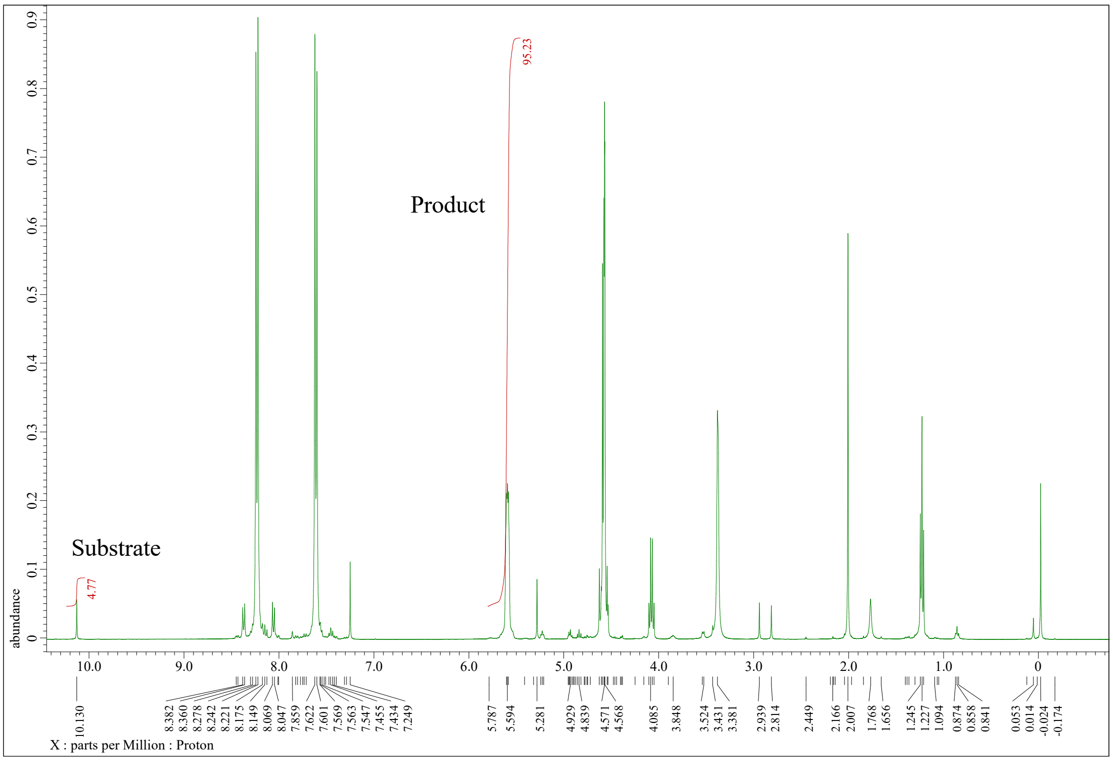

HPLC analysis

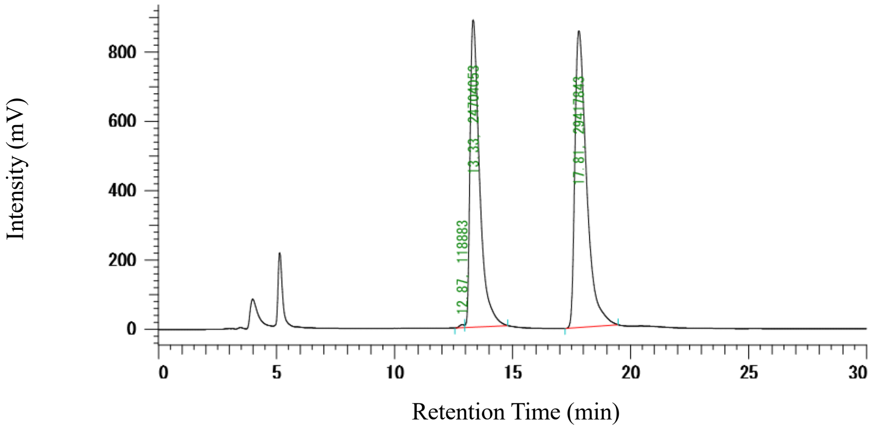

NO	RT	Area	Concentration	BC
1	12.87	118883	0.219	BV
2	13.33	24704053	45.545	VB
3	17.81	29417843	54.236	BB
		54240779	100.000	

Conv.: 95%, 9% *ee*, The *ee* of product was determined by HPLC. [205 nm, Daicel chiralpack IA-3 column, hex:^{i}PrOH = 80:20, flow rate 1.0 mL/min, t_R = 17.81 min (major), t_R = 13.33 min (minor)]

Table 1, entry 6

^{1}H NMR, 400MHz, $CDCl_3$

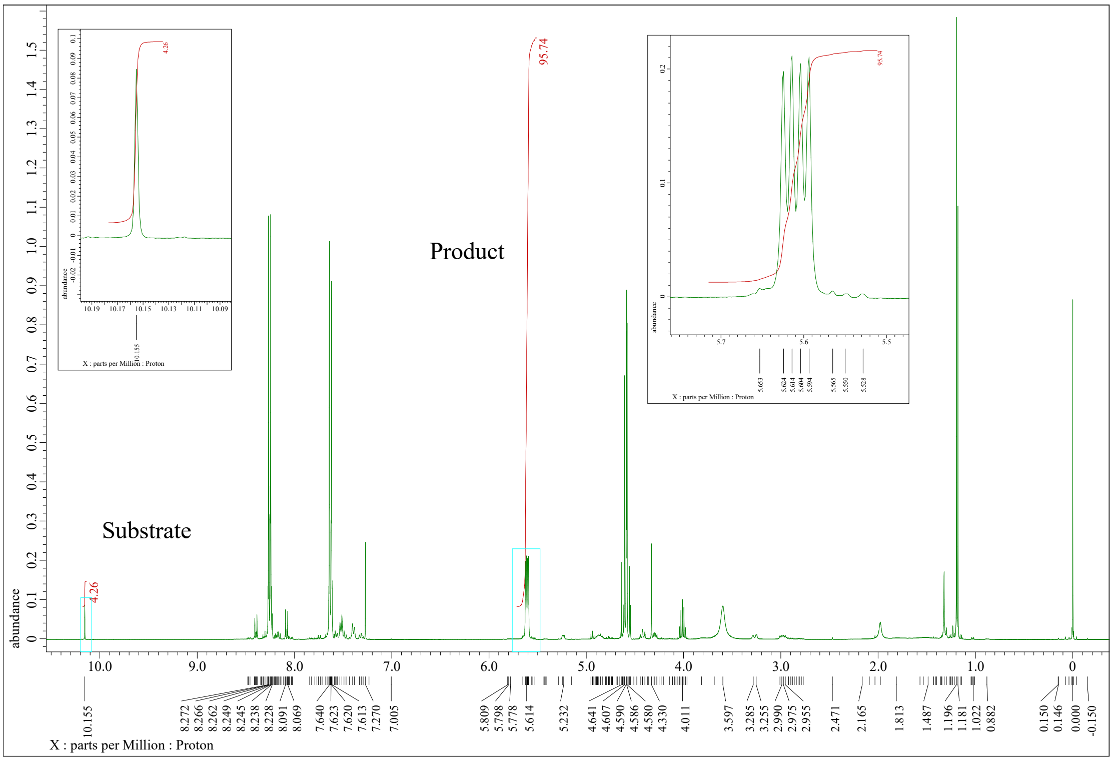

HPLC analysis

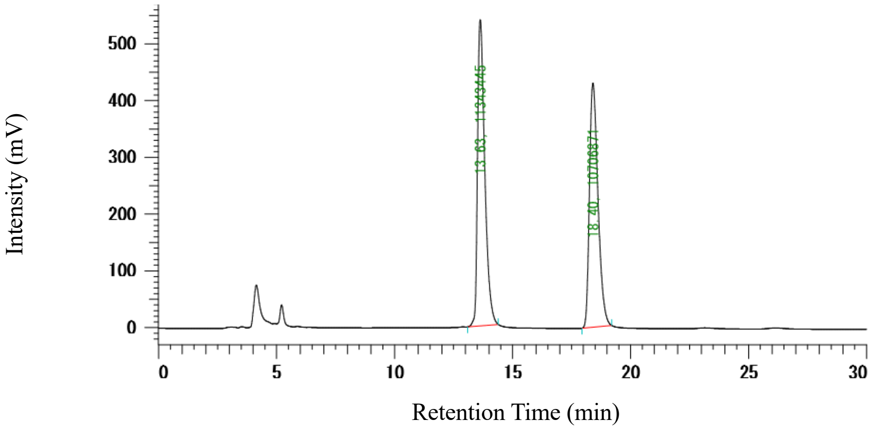

NO	RT	Area	Concentration	BC
1	13.63	11343445	51.443	BB
2	18.40	10706871	48.557	BB
		22050316	100.000	

Conv.: 96%, 3% *ee*, The *ee* of product was determined by HPLC. [205 nm, Daicel chiralpack IA-3 column, hex:^{i}PrOH = 80:20, flow rate 1.0 mL/min, t_R = 18.40 min (minor), t_R = 13.63 min (major)]

Table 2, entry 2

^{1}H NMR, 400MHz, $CDCl_3$

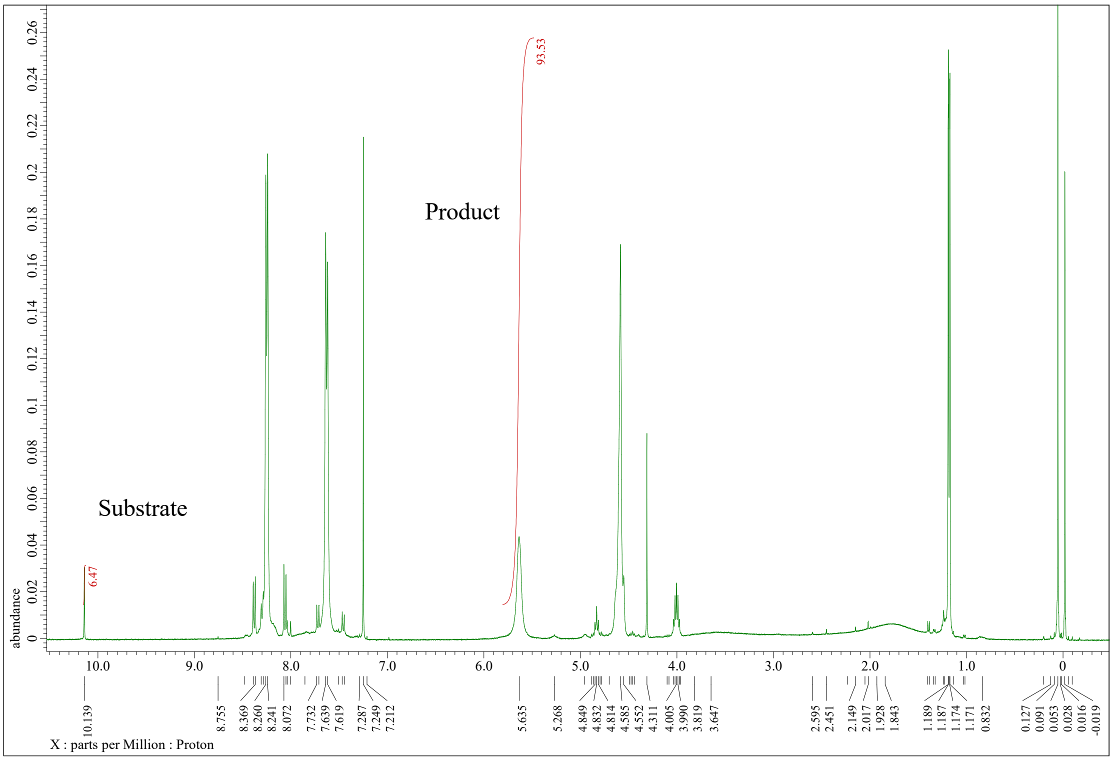

HPLC analysis

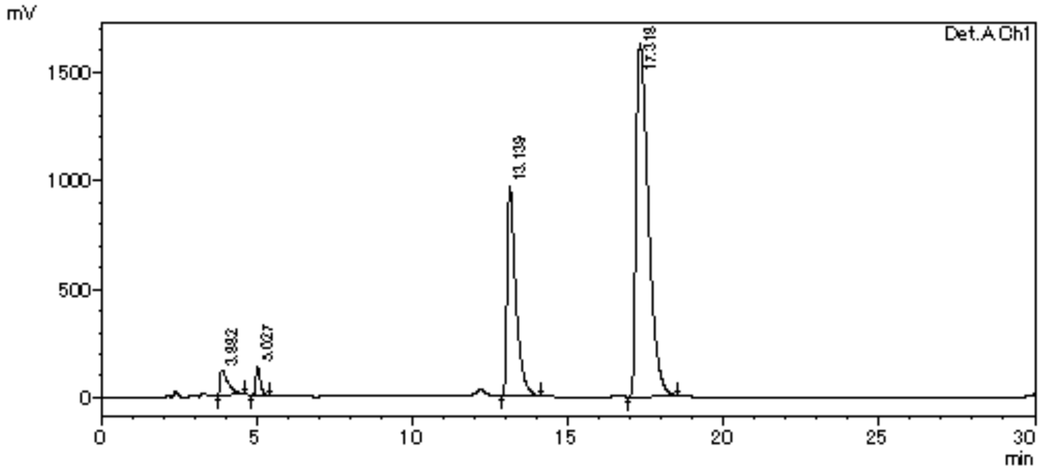

Detecter A 205 nm

Peak	Retemtion Time	Area	Height	Area%	Height%
1	3.882	2300500	120326	3.418	4.219
2	5.027	1314179	136492	1.953	4.786
3	13.139	18769031	968943	27.886	33.977
4	17.318	44921509	1626009	66.743	57.018
Sum		67305220	2851770	100.000	100.000

Conv.: 94%, 41% *ee*, The *ee* of product was determined by HPLC. [205 nm, Daicel chiralpack IA-3 column, hex:^{i}PrOH = 80:20, flow rate 1.0 mL/min, t_R = 17.318 min (major), t_R = 13.139 min (minor)]

Table 2, entry 3

^{1}H NMR, 400MHz, $CDCl_3$

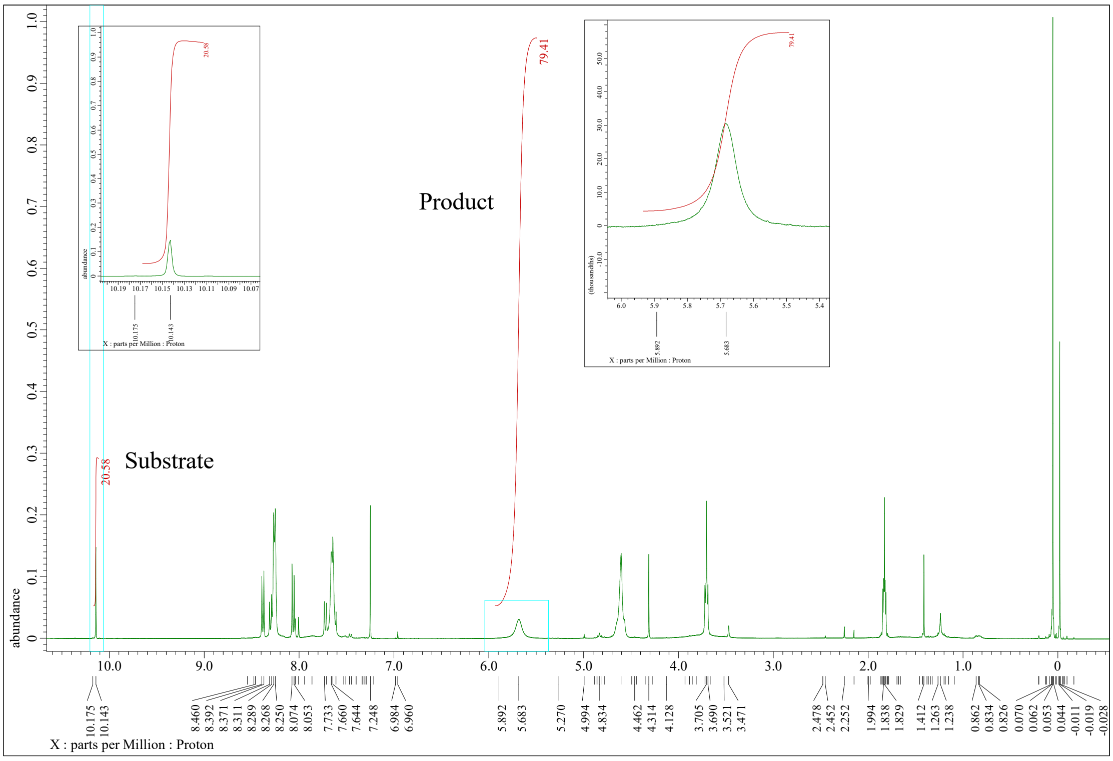

HPLC analysis

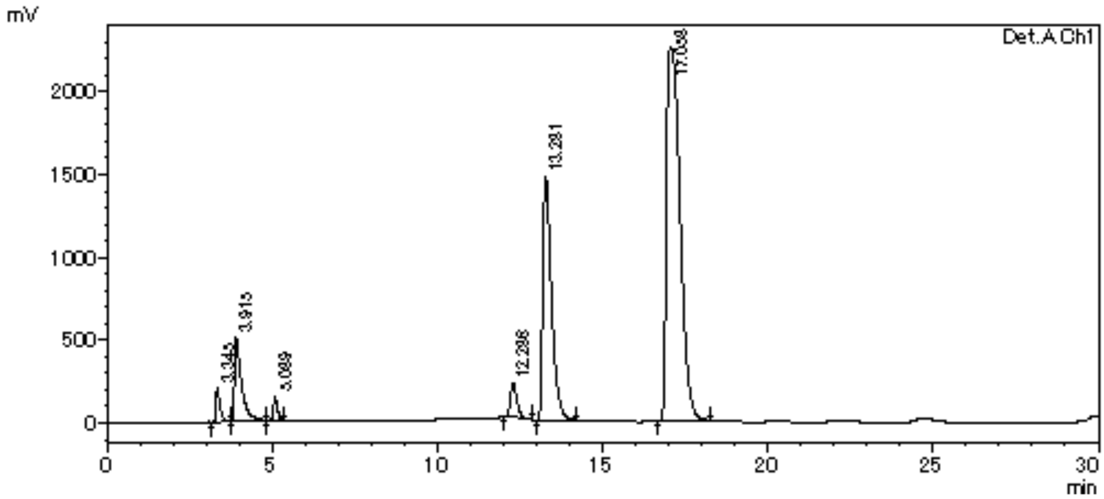

Detecter A 205 nm

Peak	Retemtion Time	Area	Height	Area%	Height%
1	3.345	1758784	209170	1.670	4.371
2	3.915	7544950	501243	7.163	10.474
3	5.089	1292917	139634	1.227	2.918
4	12.286	2959011	208342	2.809	4.353
5	13.281	27007829	1468761	25.640	30.691
6	17.058	64773241	2258553	61.492	47.194
Sum		105336733	4785703	100.000	100.000

Conv.: 79%, 41% *ee*, The *ee* of product was determined by HPLC. [205 nm, Daicel chiralpack IA-3 column, hex:^{i}PrOH = 80:20, flow rate 1.0 mL/min, t_R = 17.058 min (major), t_R = 13.281 min (minor)]

Table 2, entry 4

^{1}H NMR, 400MHz, $CDCl_3$

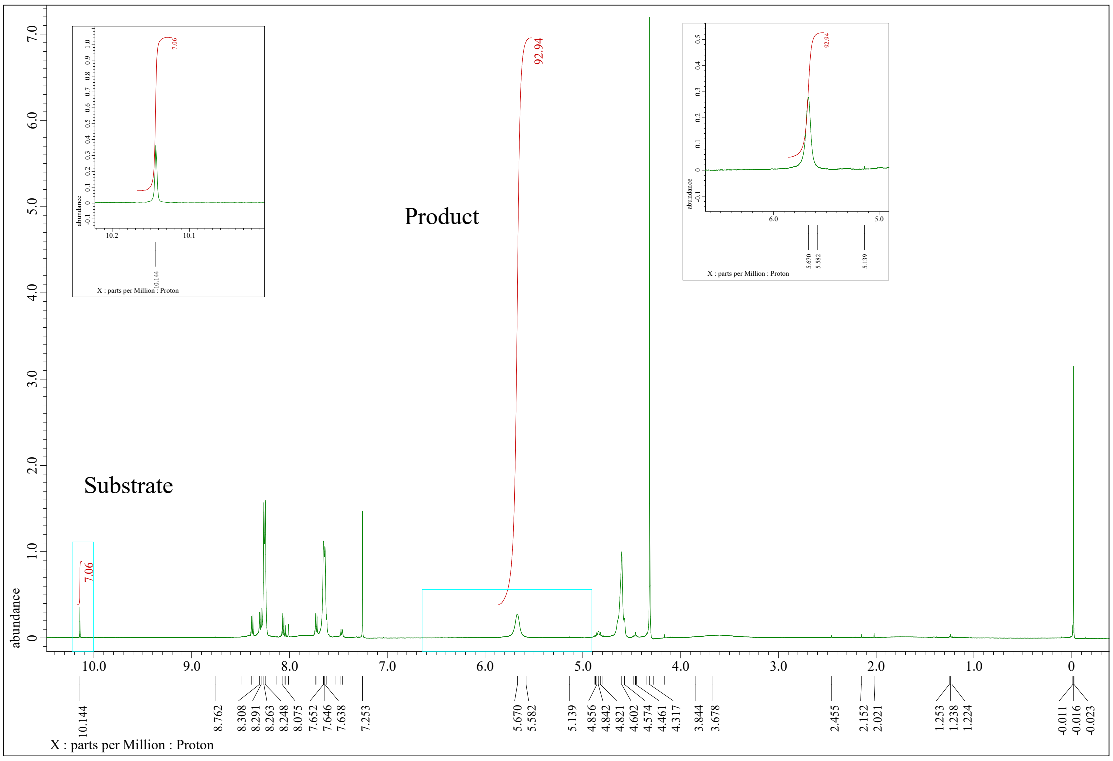

HPLC analysis

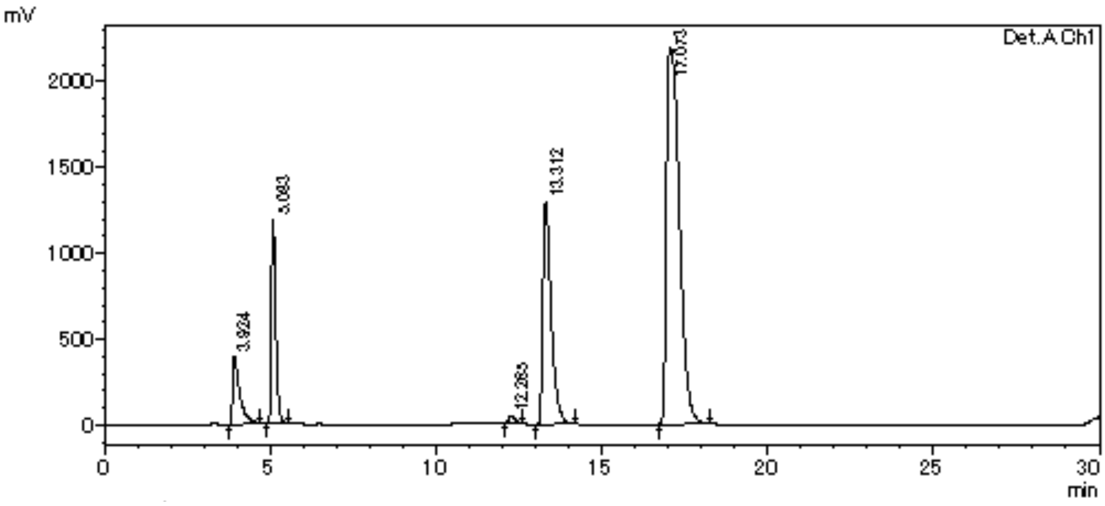

Detecter A 205 nm

Peak	Retemtion Time	Area	Height	Area%	Height%
1	3.924	5781268	393334	5.797	7.702
2	5.083	10832039	1185178	10.862	23.207
3	12.265	579823	44173	0.581	0.865
4	13.312	22625238	1291790	22.688	25.294
5	17.073	59905456	2192561	60.071	42.932
Sum		99723824	5107036	100.000	100.000

Conv.: 93%, 45% *ee*, The *ee* of product was determined by HPLC. [205 nm, Daicel chiralpack IA-3 column, hex:^{i}PrOH = 80:20, flow rate 1.0 mL/min, t_R = 17.073 min (major), t_R = 13.312 min (minor)]

Table 2, entry 5

^{1}H NMR, 400MHz, $CDCl_3$

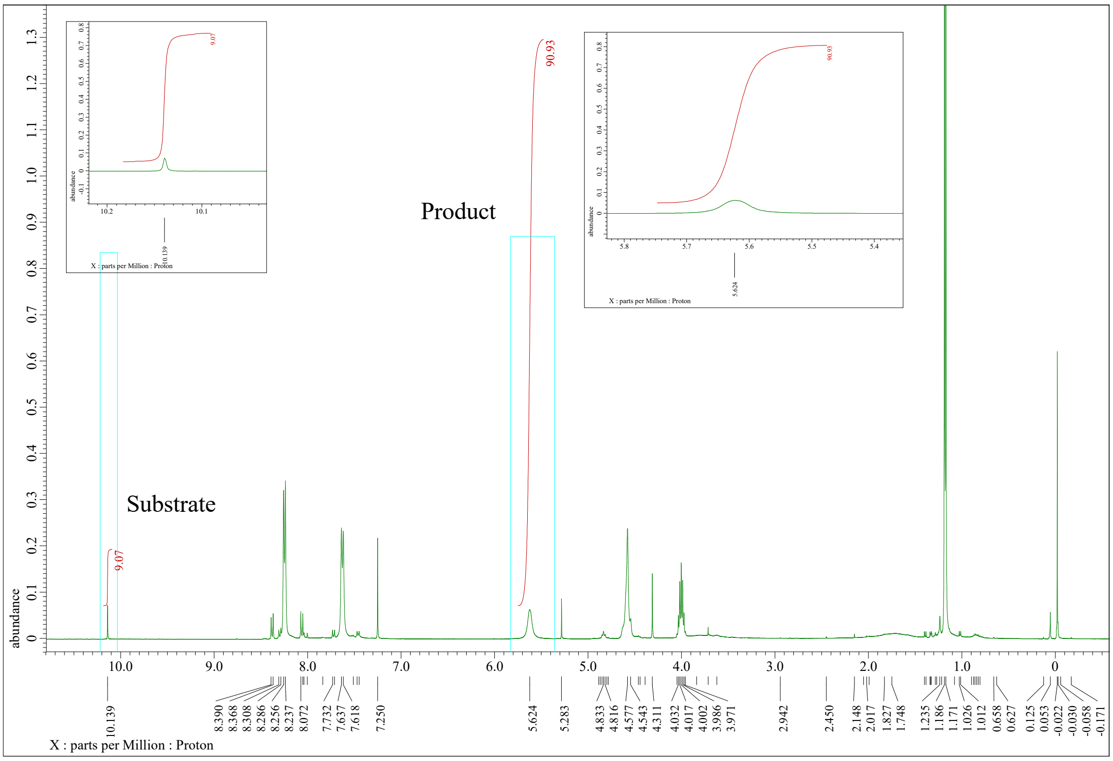

HPLC analysis

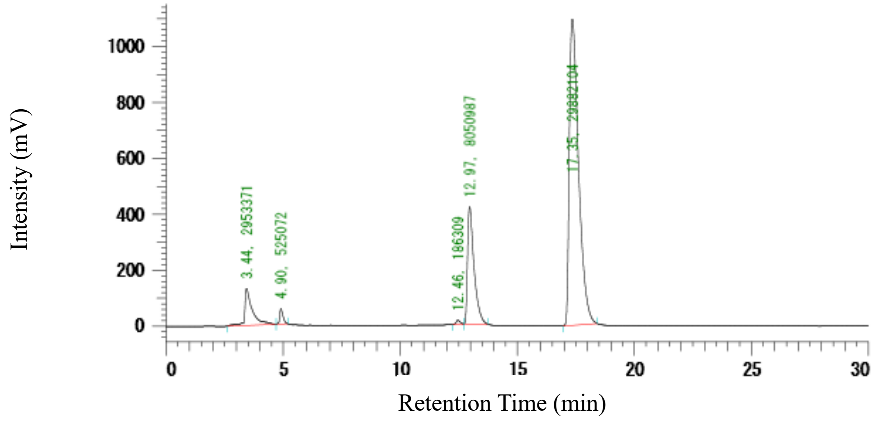

NO	RT	Area	Concentration	BC
1	3. 44	2953371	7. 100	BB
2	4. 90	525072	1. 262	BB
3	12. 46	186309	0. 448	BB
4	12. 97	8050987	19. 354	BB
5	17. 35	29882104	71. 836	BB
		41597843	100. 000	

Conv.: 91%, 58% *ee*, The *ee* of product was determined by HPLC. [205 nm, Daicel chiralpack IA-3 column, hex:^{i}PrOH = 80:20, flow rate 1.0 mL/min, t_R = 17.35 min (major), t_R = 12.97 min (minor)]

Table 2, entry 6

^{1}H NMR, 400MHz, $CDCl_3$

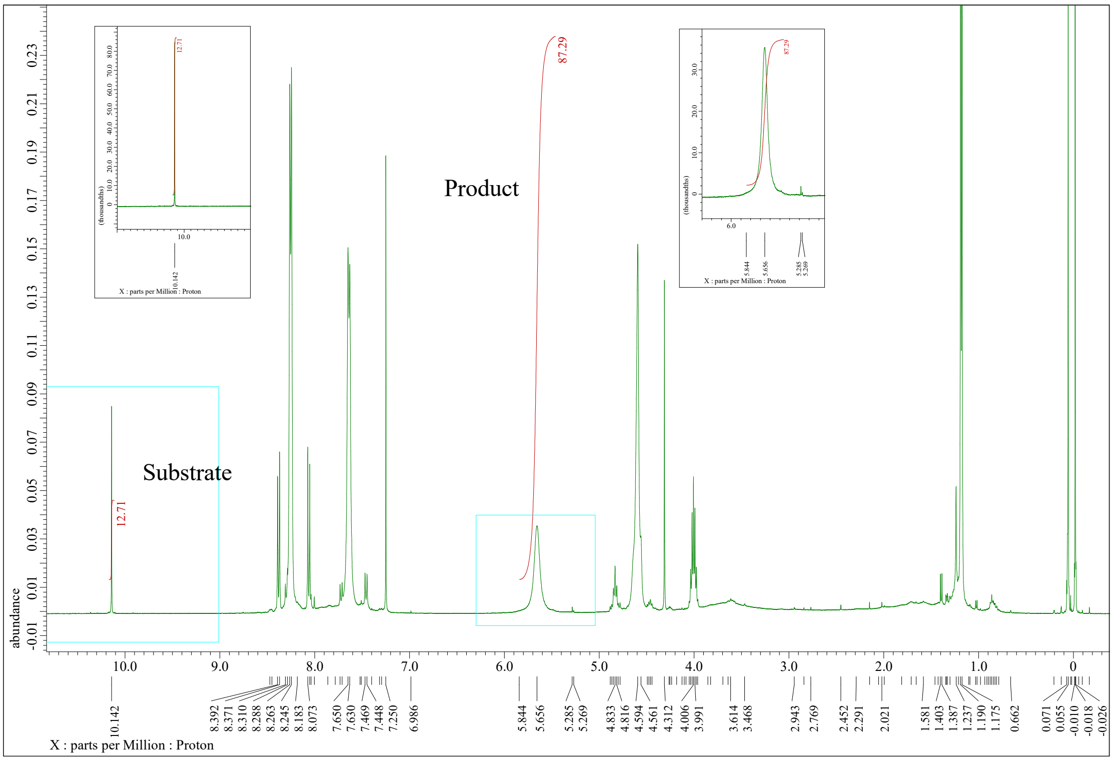

HPLC analysis

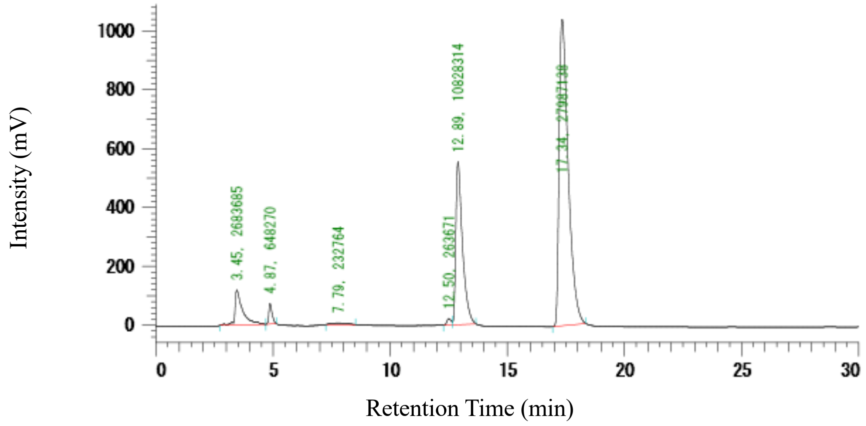

NO	RT	Area	Concentration	BC
1	3.45	2683685	6.293	BB
2	4.87	648270	1.520	BB
3	7.79	232764	0.546	BB
4	12.50	263671	0.618	BV
5	12.89	10828314	25.392	VB
6	17.34	27987138	65.630	BB
		42643842	100.000	

Conv.: 87%, 44% *ee*, The *ee* of product was determined by HPLC. [205 nm, Daicel chiralpack IA-3 column, hex:^{i}PrOH = 80:20, flow rate 1.0 mL/min, t_R = 17.34 min (major), t_R = 12.89 min (minor)]

Table 2, entry 7

^{1}H NMR, 400MHz, $CDCl_3$

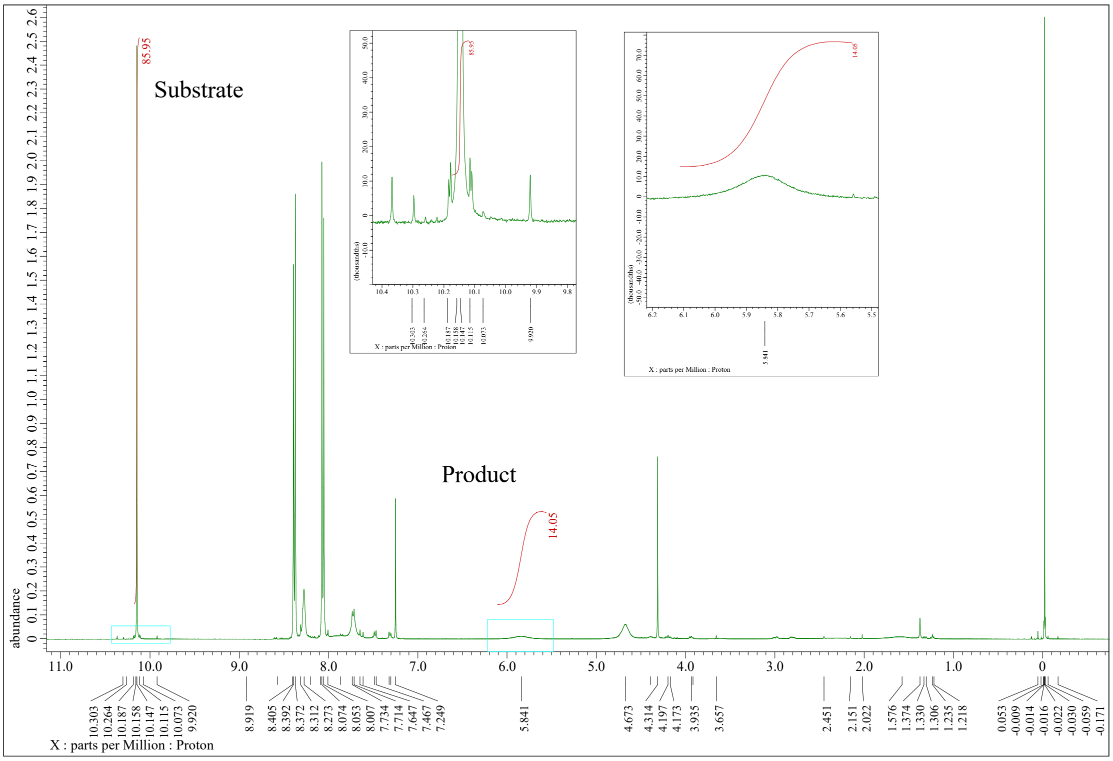

HPLC analysis

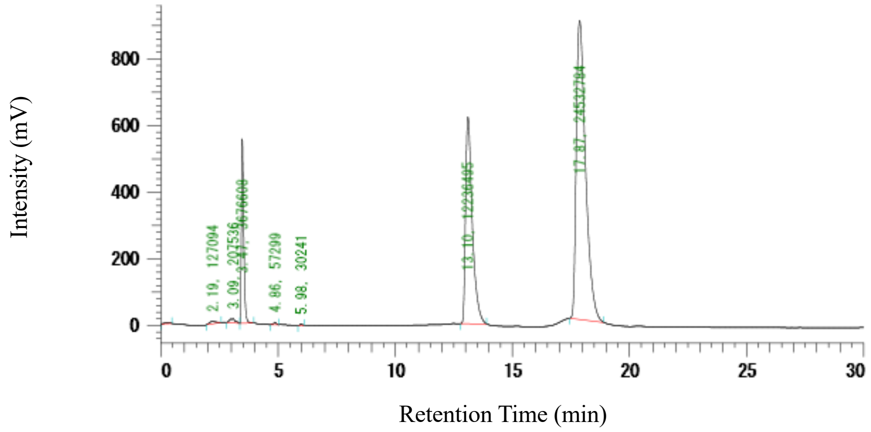

NO	RT	Area	Concentration	BC
1	0.31	37478	0.092	BB
2	2.19	127094	0.311	BB
3	3.09	207536	0.507	BB
4	3.47	3676608	8.988	BB
5	4.86	57299	0.140	BB
6	5.98	30241	0.074	BB
7	13.10	12236495	29.914	BB
8	17.87	24532784	59.974	BB
		40905535	100.000	

Conv.: 14%, 33% *ee*, The *ee* of product was determined by HPLC. [205 nm, Daicel chiralpack IA-3 column, hex:^{i}PrOH = 80:20, flow rate 1.0 mL/min, t_R = 17.87 min (major), t_R = 13.10 min (minor)]

Table 2, entry 8

^{1}H NMR, 400MHz, $CDCl_3$

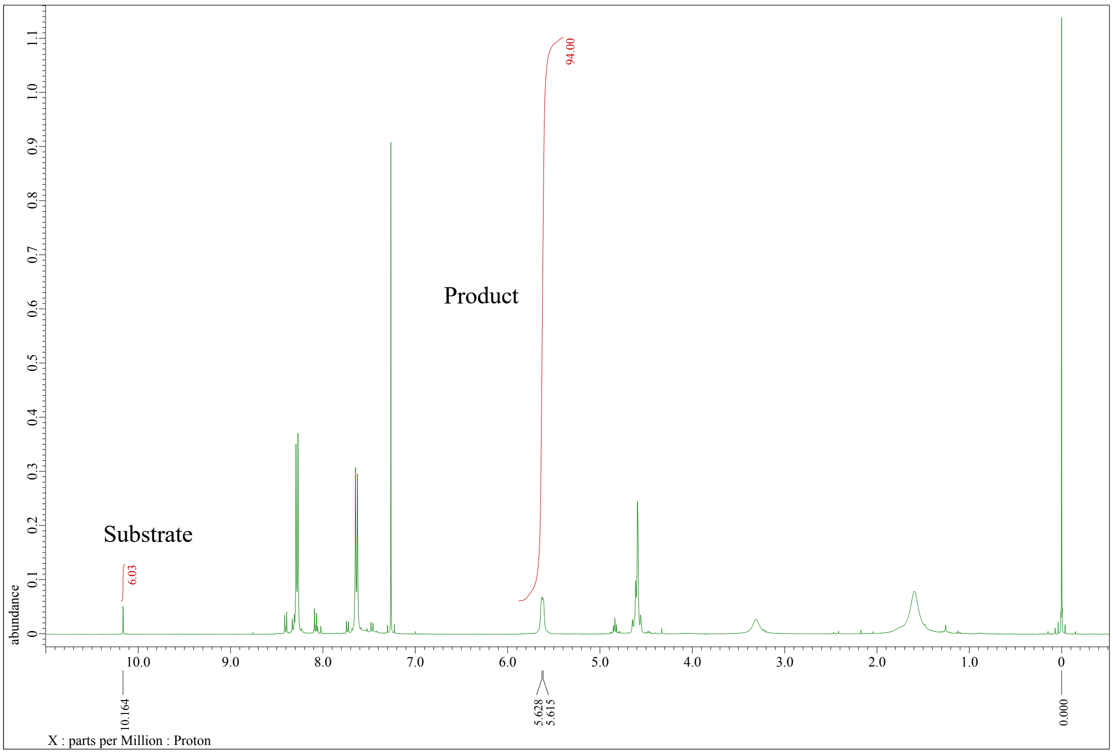

HPLC analysis

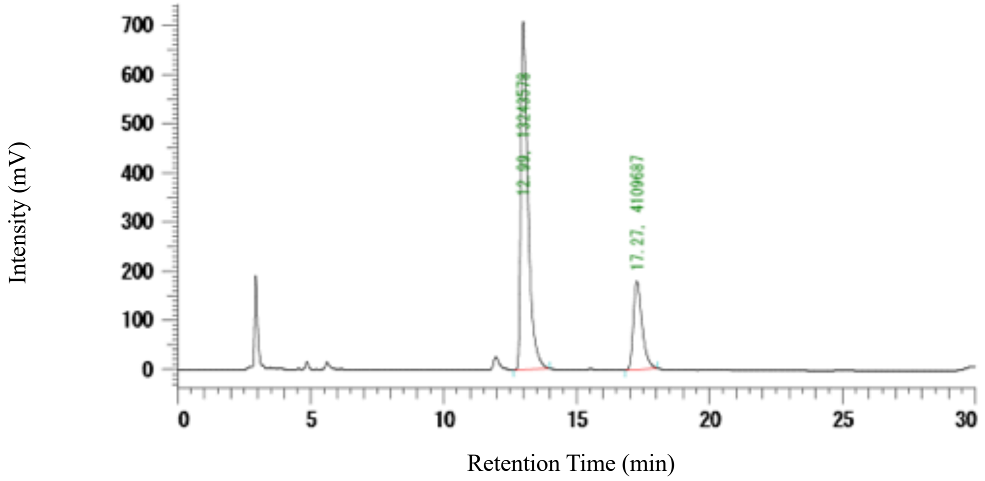

NO	RT	Area	Concentration	BC
1	12.99	13243578	76.317	BB
2	17.27	4109687	23.682	BB
		17353265	100.000	

Conv.: 94%, 53% *ee*, The *ee* of product was determined by HPLC. [205 nm, Daicel chiralpack IA-3 column, hex:^{i}PrOH = 80:20, flow rate 1.0 mL/min, t_R = 17.27 min (minor), t_R = 12.99 min (major)]

Table 2, entry 9

^{1}H NMR, 400MHz, $CDCl_3$

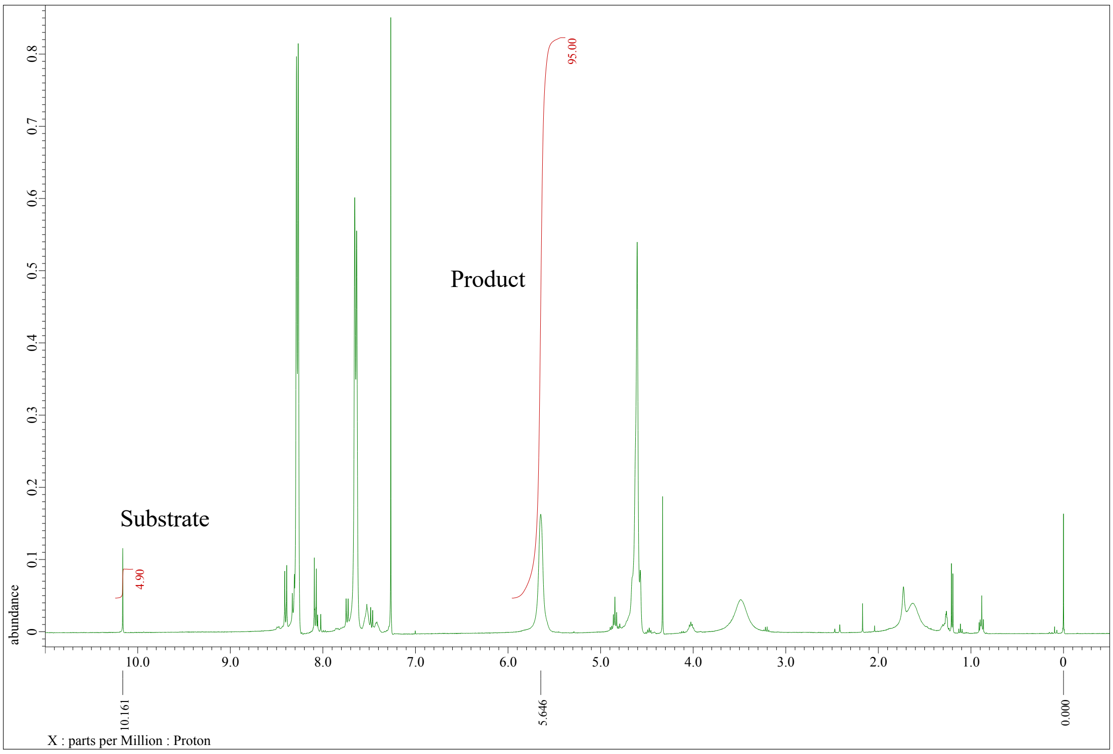

HPLC analysis

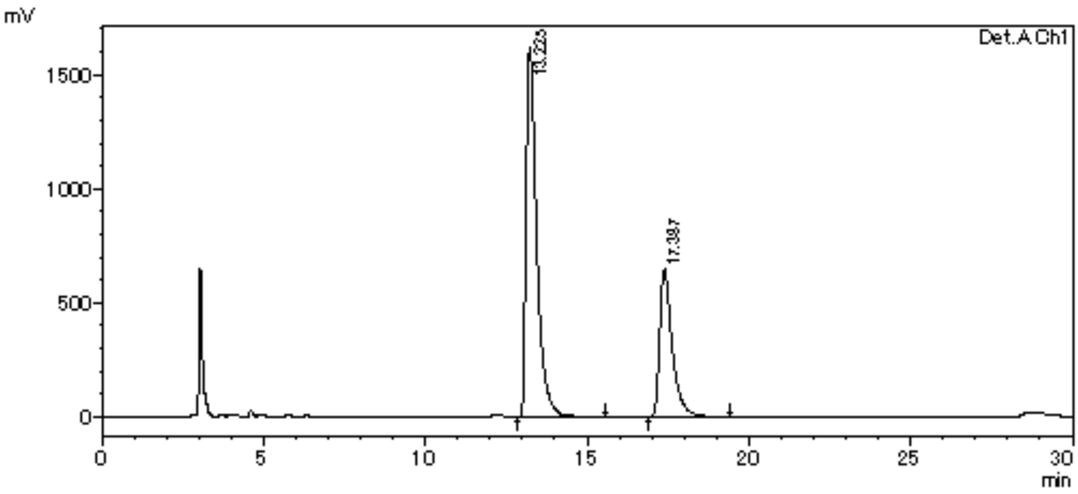

Detecter A 205 nm

Peak	Retemtion Time	Area	Height	Area%	Height%
1	13.225	36683829	1618282	67.816	71.520
2	17.387	17409236	644412	32.184	28.480
Sum		54093065	2262694	100.000	100.000

Conv.: 95%, 36% *ee*, The *ee* of product was determined by HPLC. [205 nm, Daicel chiralpack IA-3 column, hex:^{i}PrOH = 80:20, flow rate 1.0 mL/min, t_R = 17.39 min (minor), t_R = 13.23 min (major)]

Table 2, entry 10

^{1}H NMR, 400MHz, $CDCl_3$

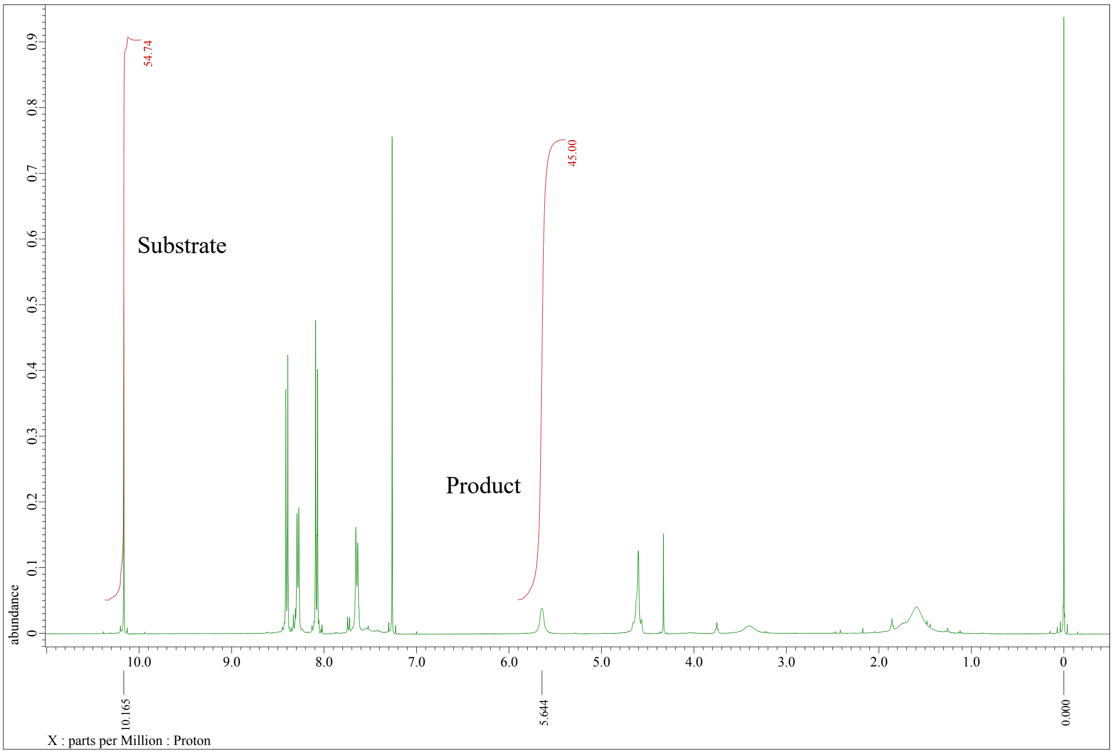

HPLC analysis

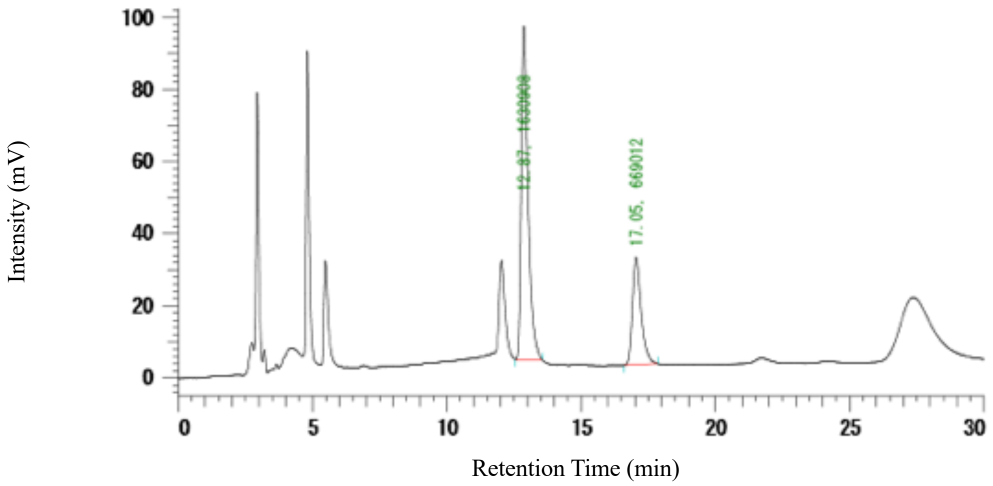

NO	RT	Area	Concentration	BC
1	12.87	1630908	70.912	BB
2	17.05	669012	29.088	BB
		2299920	100.000	

Conv.: 45%, 42% *ee*, The *ee* of product was determined by HPLC. [205 nm, Daicel chiralpack IA-3 column, hex:^{i}PrOH = 80:20, flow rate 1.0 mL/min, t_R = 17.05 min (minor), t_R = 12.87 min (major)]

Table 2, entry 11

^{1}H NMR, 400MHz, $CDCl_3$

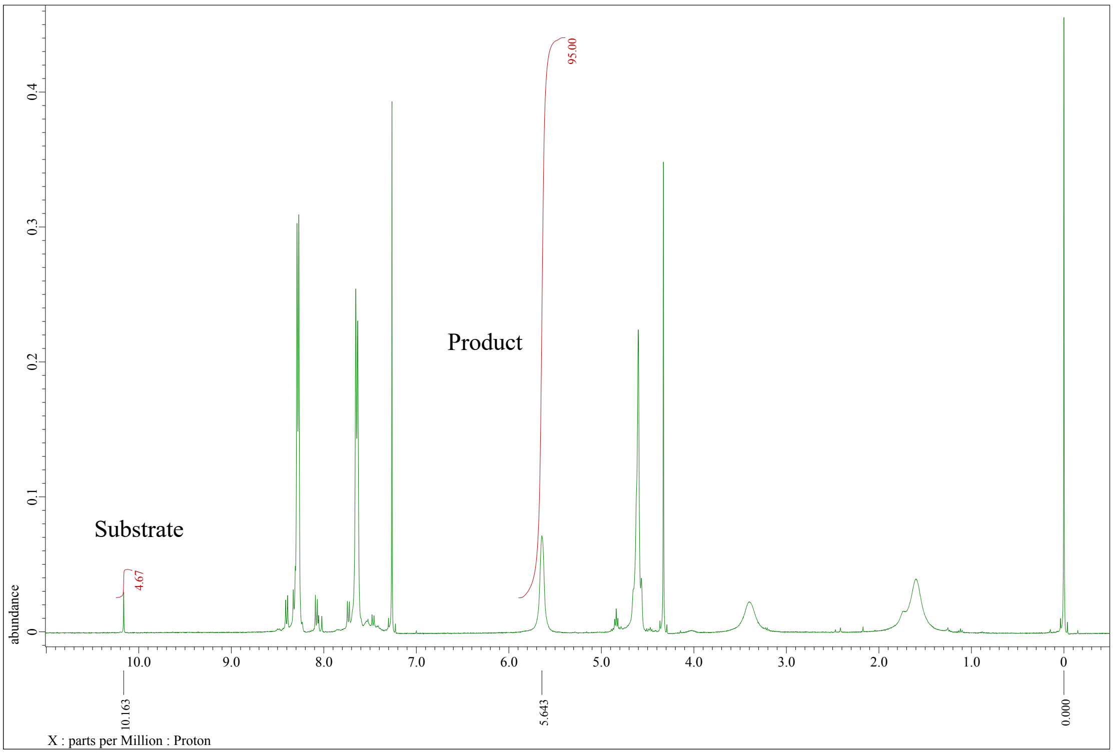

HPLC analysis

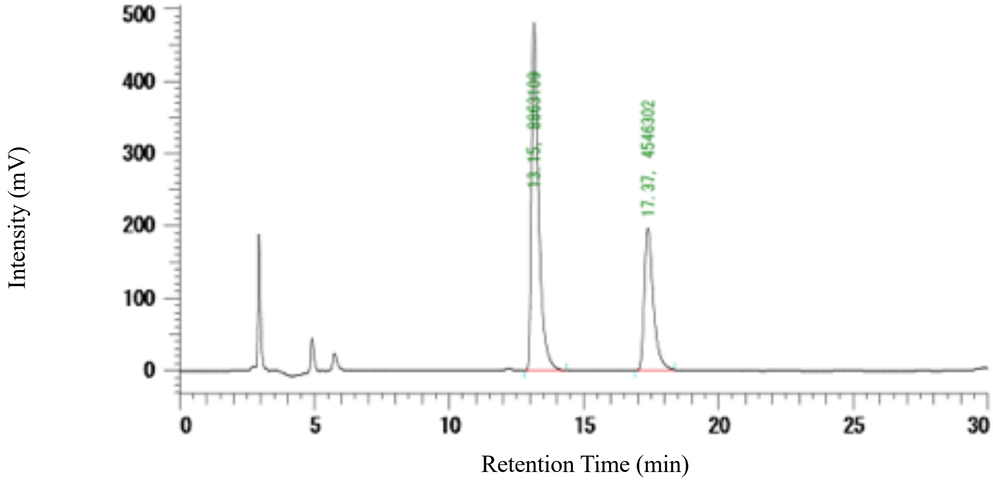

NO	RT	Area	Concentration	BC
1	13.15	8863109	66.096	BB
2	17.37	4546302	33.904	BB
		13409411	100.000	

Conv.: 95%, 32% *ee*, The *ee* of product was determined by HPLC. [205 nm, Daicel chiralpack IA-3 column, hex:^{i}PrOH = 80:20, flow rate 1.0 mL/min, t_R = 17.37 min (minor), t_R = 13.15 min (major)]

Table 2, entry 12

^{1}H NMR, 400MHz, $CDCl_3$

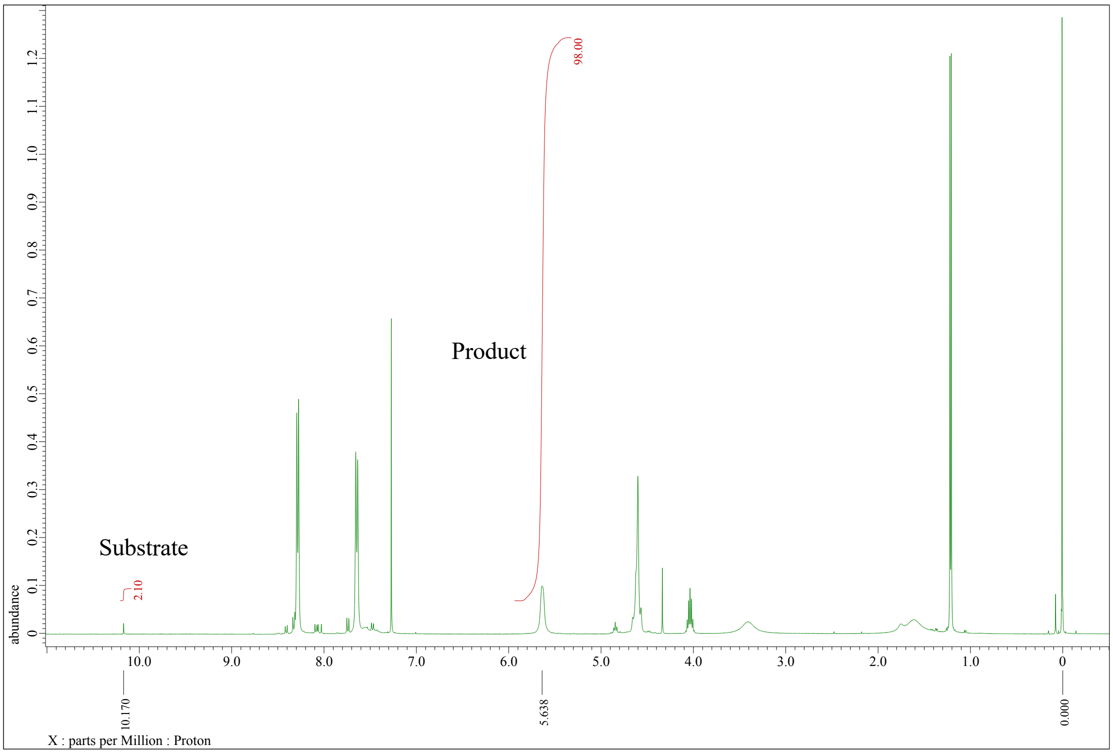

HPLC analysis

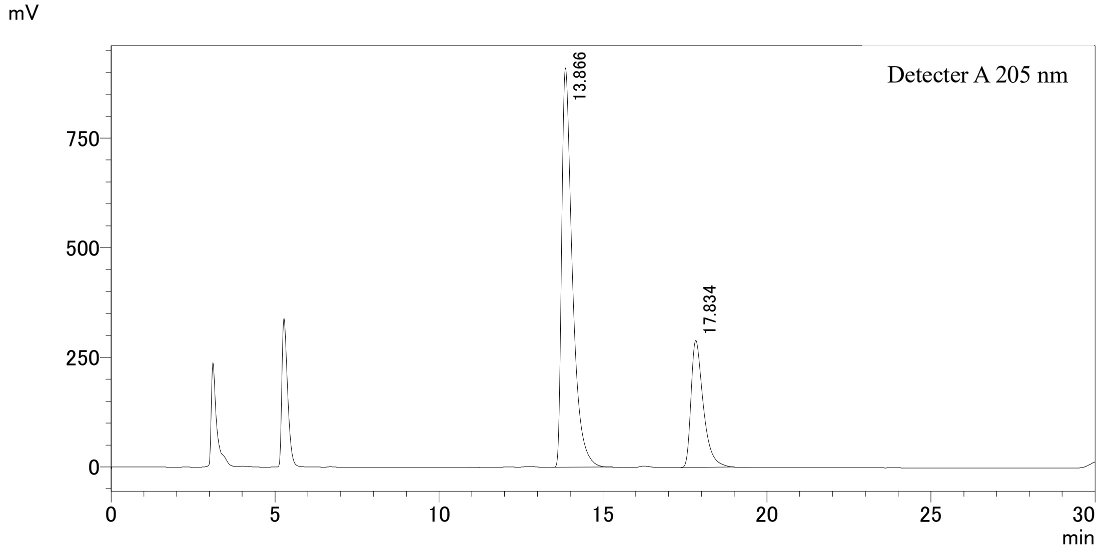

Detecter A 205 nm

Peak	Retemtion Time	Area	Height	Concentration
1	13.866	20699126	860545	73.328
2	17.834	7529084	276425	26.672
Sum		28228210	1136970	

Conv.: 98%, 47% *ee*, The *ee* of product was determined by HPLC. [205 nm, Daicel chiralpack IA-3 column, hex:^{i}PrOH = 80:20, flow rate 1.0 mL/min, t_R = 17.83 min (minor), t_R = 13.87 min (major)]

Table 2, entry 13

^{1}H NMR, 400MHz, $CDCl_3$

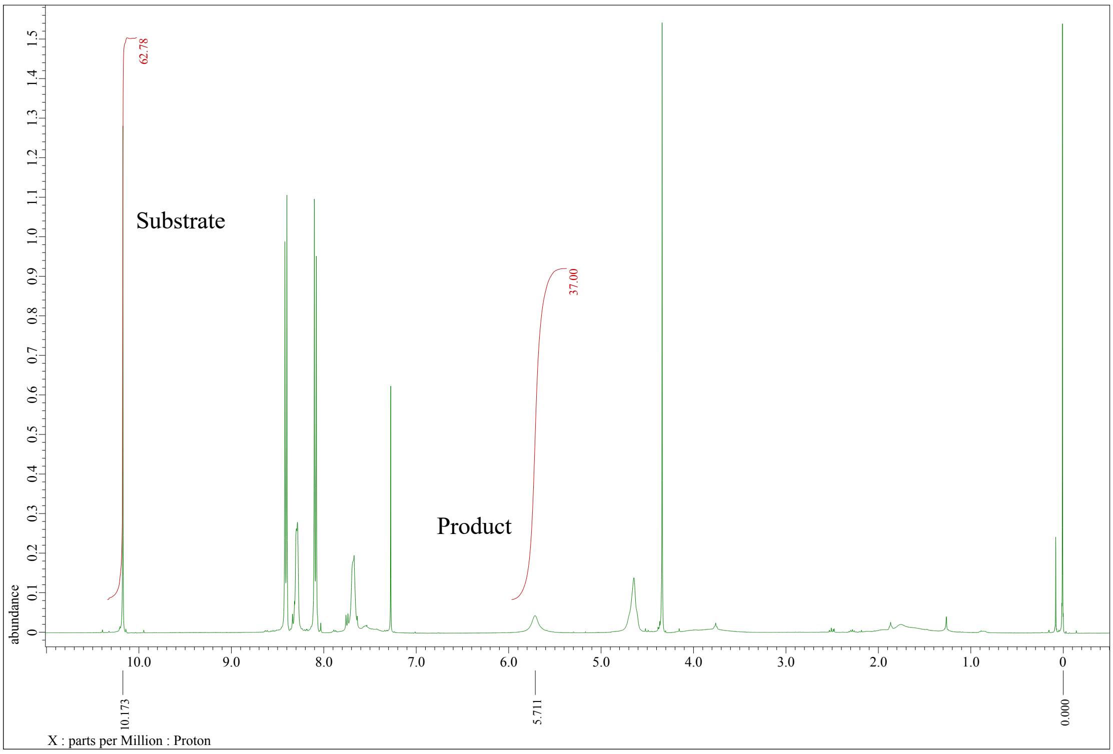

HPLC analysis

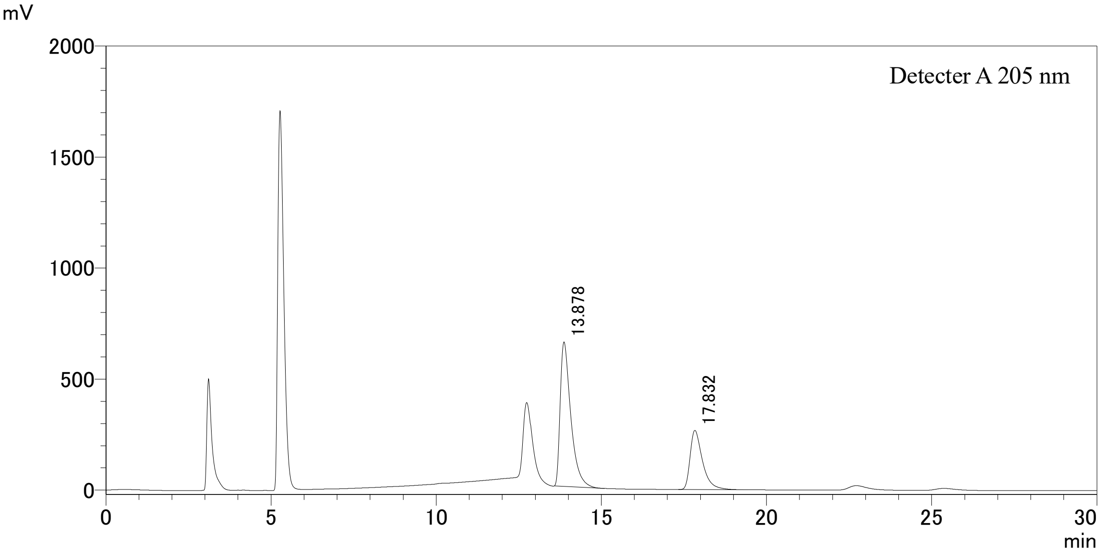

Detecter A 205 nm

Peak	Retemtion Time	Area	Height	Concentration
1	13.878	14595018	638954	67.661
2	17.832	6975919	256871	32.339
Sum		21570938	895825	

Conv.: 37%, 35% *ee*, The *ee* of product was determined by HPLC. [205 nm, Daicel chiralpack IA-3 column, hex:^{i}PrOH = 80:20, flow rate 1.0 mL/min, t_R = 17.83 min (minor), t_R = 13.89 min (major)]

Table 2, entry 14

^{1}H NMR, 400MHz, $CDCl_3$

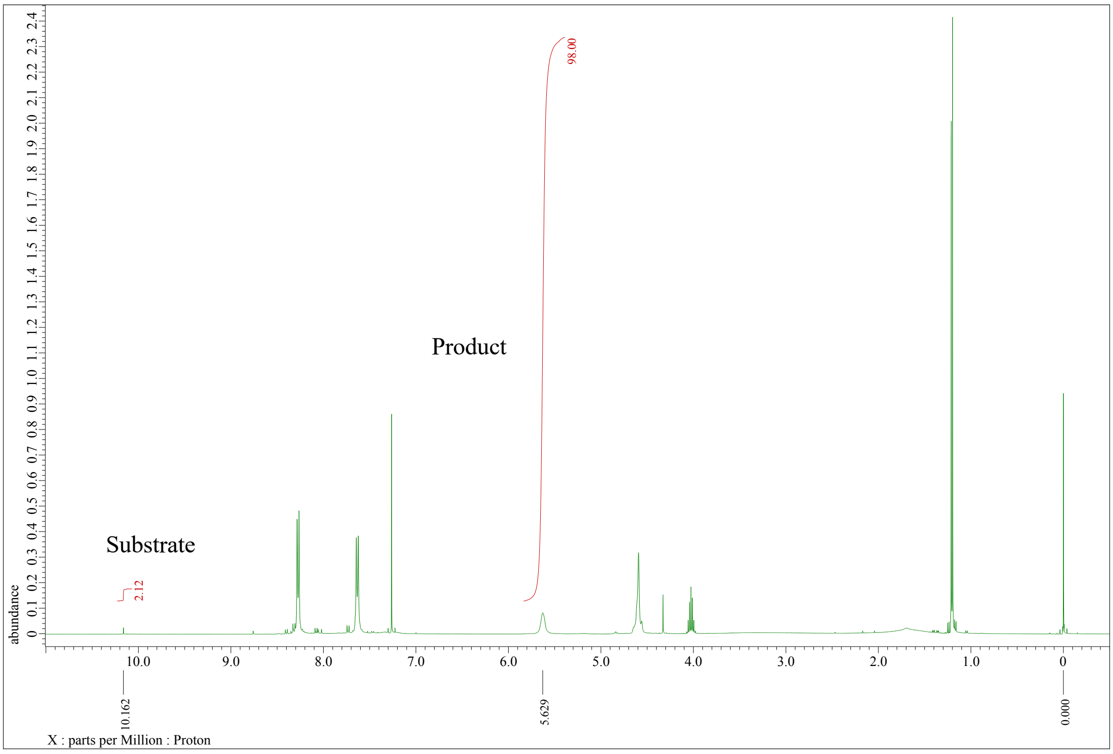

HPLC analysis

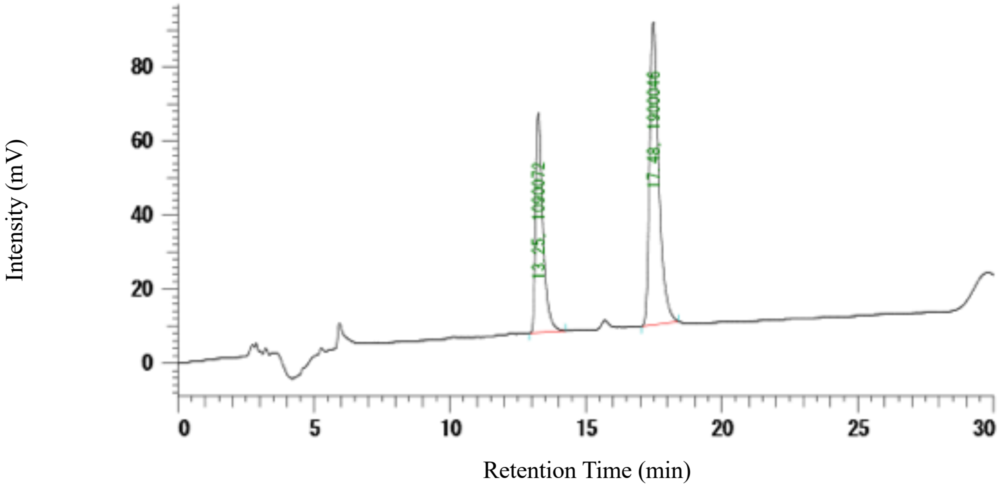

NO	RT	Area	Concentration	BC
1	13.25	1090072	36.456	BB
2	17.48	1900046	63.544	BB
		2990118	100.000	

Conv.: 98%, 27% *ee*, The *ee* of product was determined by HPLC. [205 nm, Daicel chiralpack IA-3 column, hex:^{i}PrOH = 80:20, flow rate 1.0 mL/min, t_R = 17.48 min (major), t_R = 13.28 min (minor)]

Table 2, entry 15

^{1}H NMR, 400MHz, $CDCl_3$

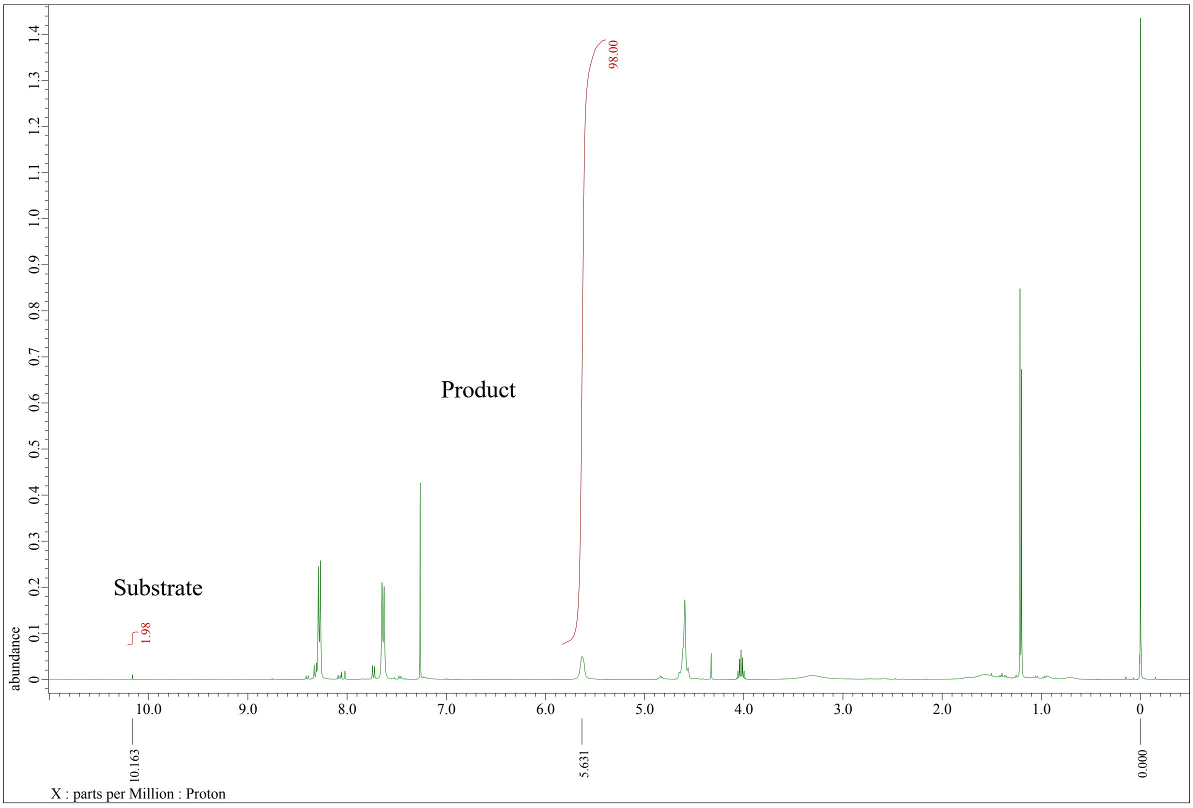

HPLC analysis

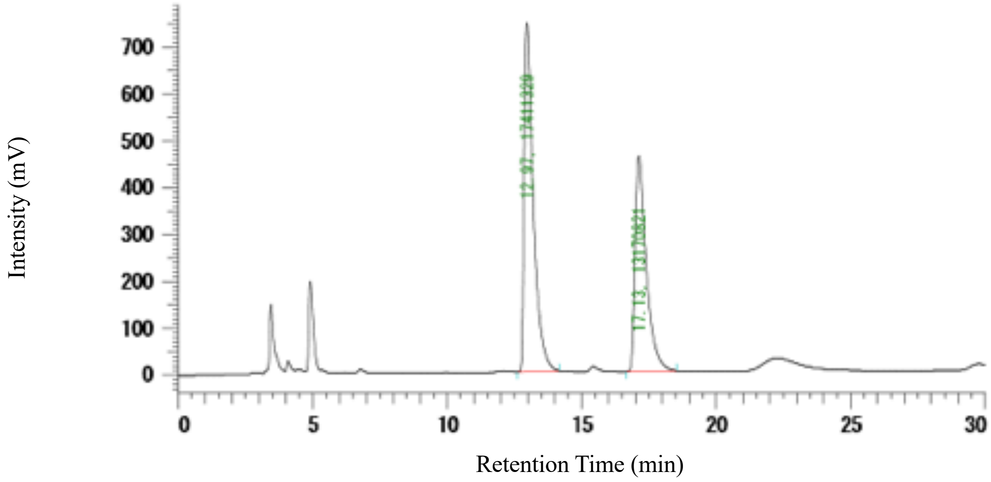

NO	RT	Area	Concentration	BC
1	12.97	17411329	56.933	BB
2	17.13	13170821	43.067	BB
		30582150	100.000	

Conv.: 98%, 14% *ee*, The *ee* of product was determined by HPLC. [205 nm, Daicel chiralpack IA-3 column, hex:^{i}PrOH = 80:20, flow rate 1.0 mL/min, t_R = 17.13 min (minor), t_R = 12.97 min (major)]

Table 3, entry 1

^{1}H NMR, 400MHz, $CDCl_3$

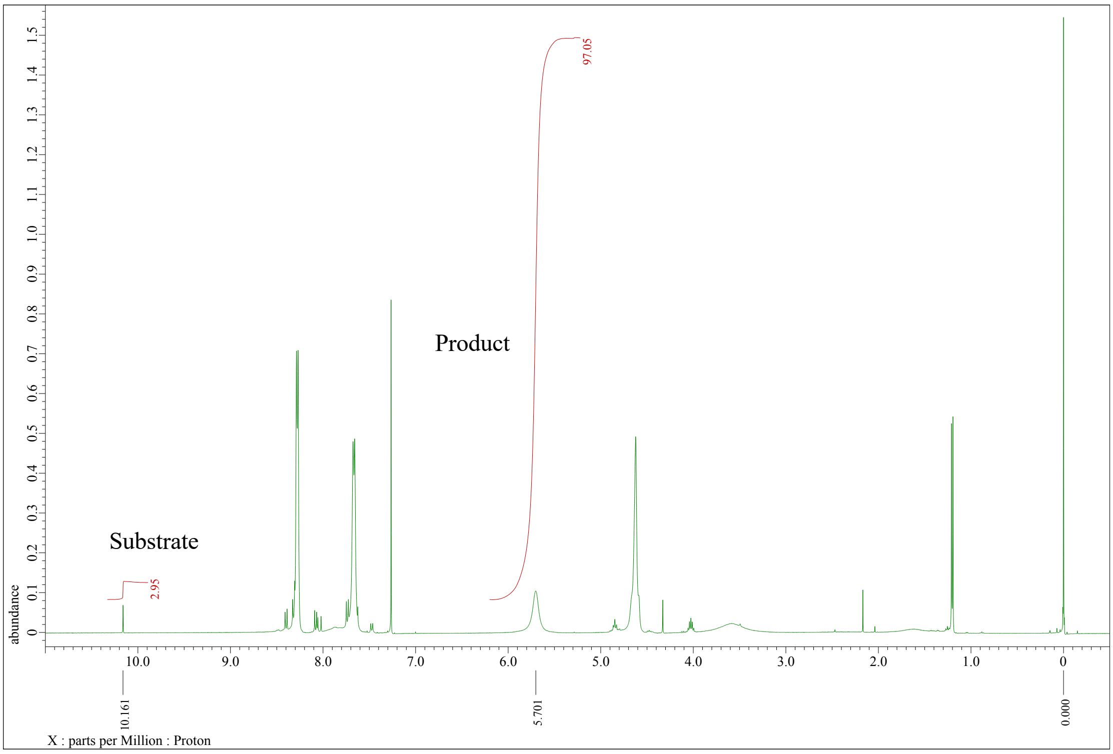

HPLC analysis

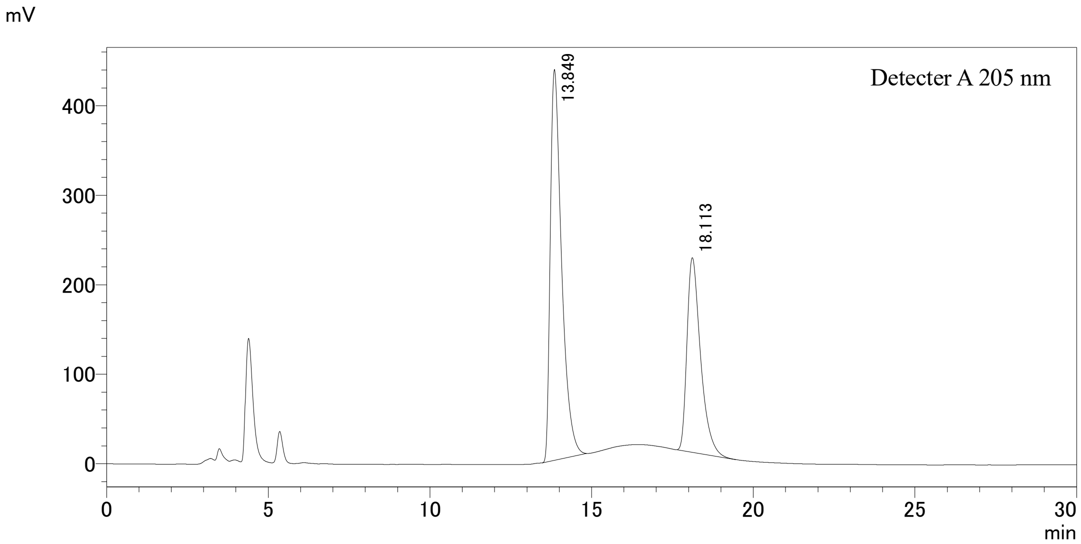

Detecter A 205 nm

Peak	Retemtion Time	Area	Height	Concentration
1	13.849	10638425	436606	62.557
2	18.113	6367589	217604	37.443
Sum		17006014	654210	

Conv.: 97%, 25% *ee*, The *ee* of product was determined by HPLC. [205 nm, Daicel chiralpack IA-3 column, hex:^{i}PrOH = 80:20, flow rate 1.0 mL/min, t_R = 18.11 min (minor), t_R = 13.85 min (major)]

Table 3, entry 2

^{1}H NMR, 400MHz, $CDCl_3$

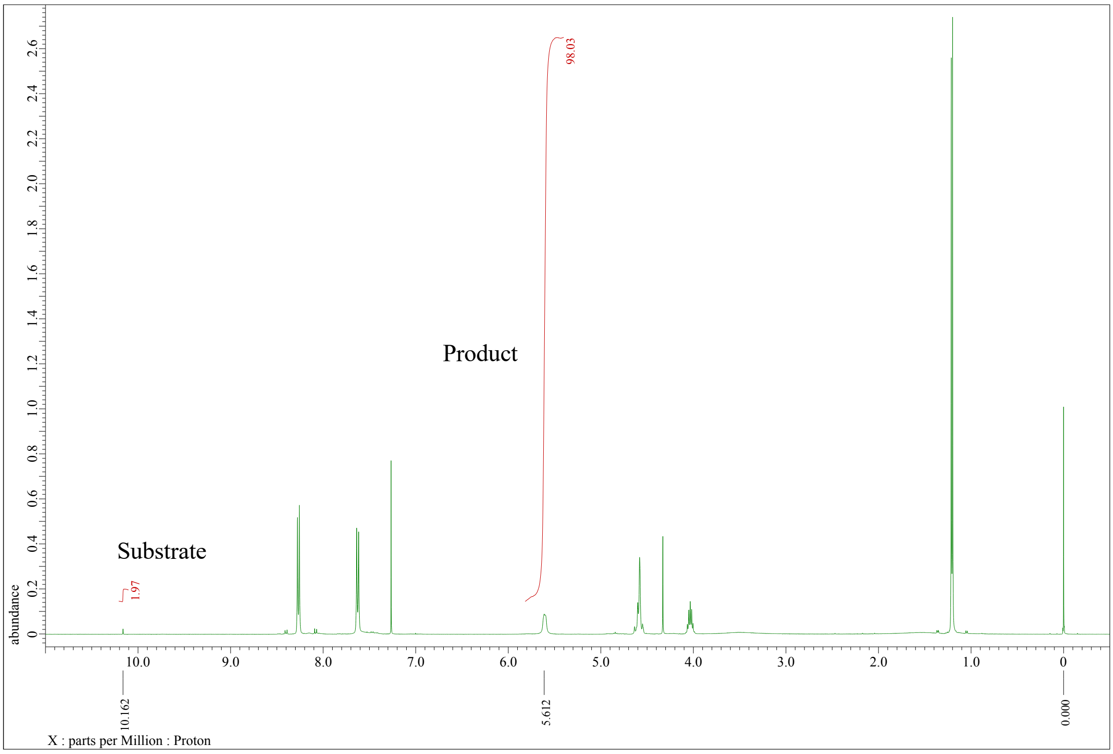

HPLC analysis

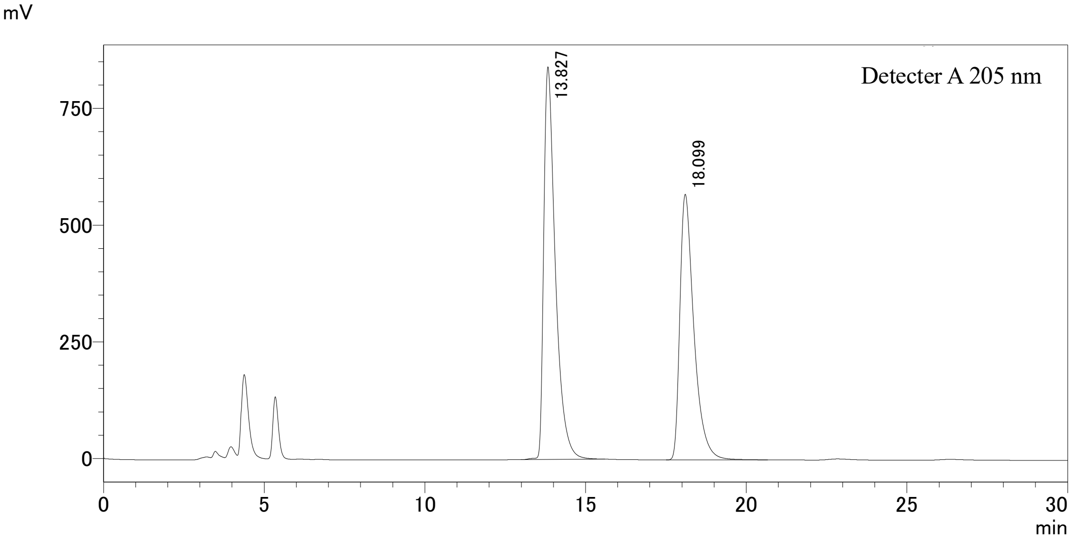

Detecter A 205 nm

Peak	Retemtion Time	Area	Height	Concentration
1	13.827	21138419	840791	55.054
2	18.099	17257289	569096	44.946
Sum		38395708	1409888	

Conv.: 98%, 10% *ee*, The *ee* of product was determined by HPLC. [205 nm, Daicel chiralpack IA-3 column, hex:^{i}PrOH = 80:20, flow rate 1.0 mL/min, t_R = 18.10 min (minor), t_R = 13.83 min (major)]

Table 3, entry 3

^{1}H NMR, 400MHz, $CDCl_3$

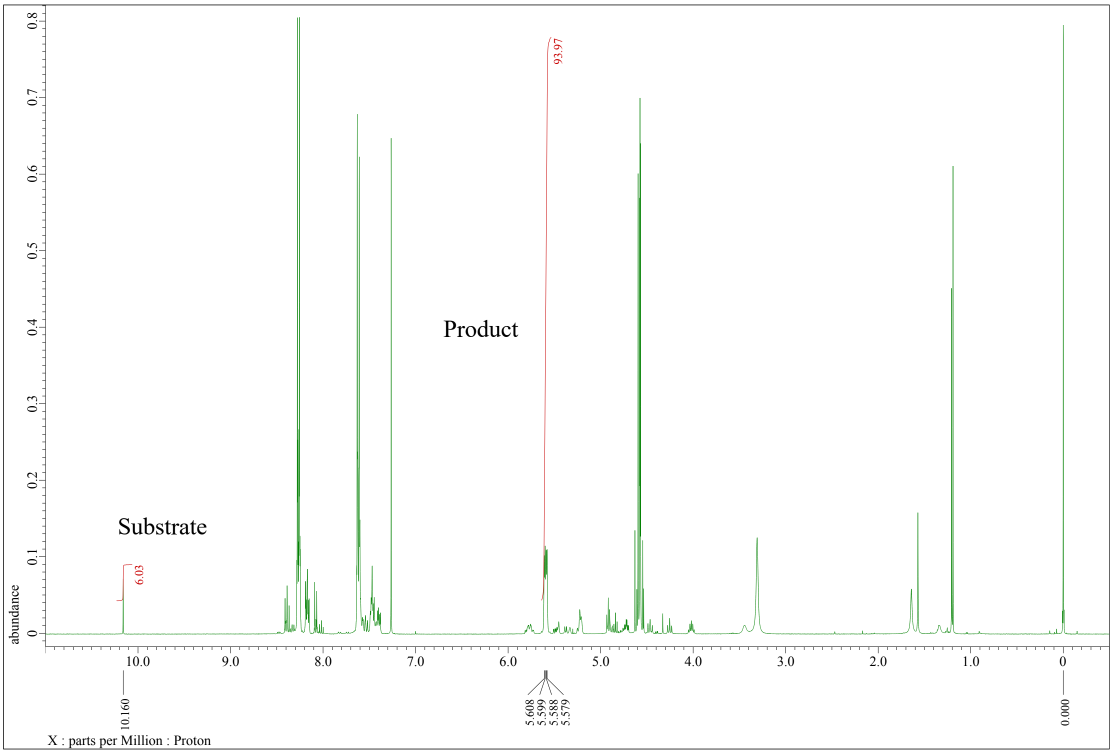

HPLC analysis

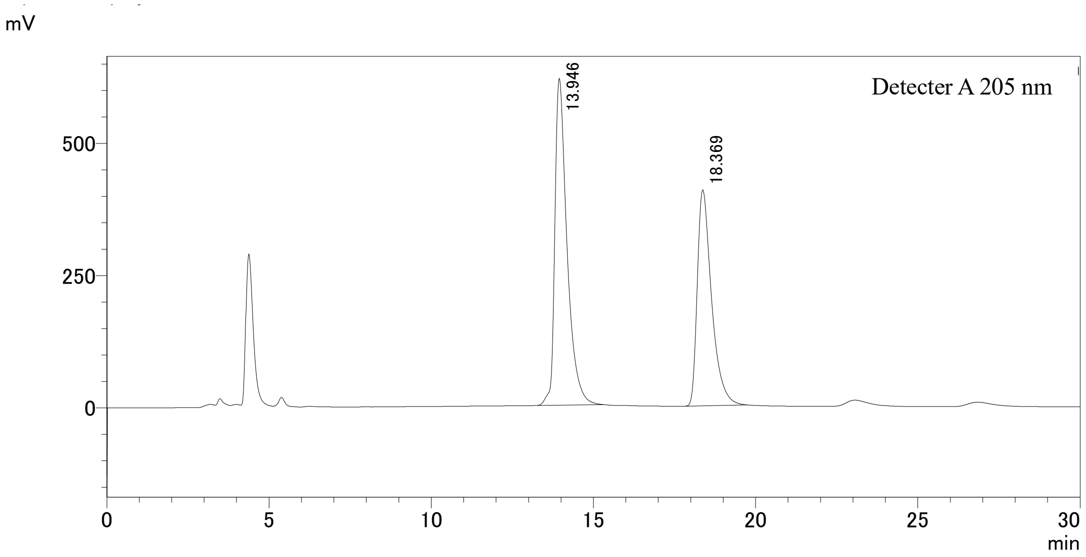

Detecter A 205 nm

Peak	Retemtion Time	Area	Height	Concentration
1	13.946	16187973	617759	56.361
2	18.369	12534036	408444	43.639
Sum		28722009	1026203	

Conv.: 94%, 13% *ee*, The *ee* of product was determined by HPLC. [205 nm, Daicel chiralpack IA-3 column, hex:^{i}PrOH = 80:20, flow rate 1.0 mL/min, t_R = 18.37 min (minor), t_R = 13.95 min (major)]

Table 3, entry 4

^{1}H NMR, 400MHz, $CDCl_3$

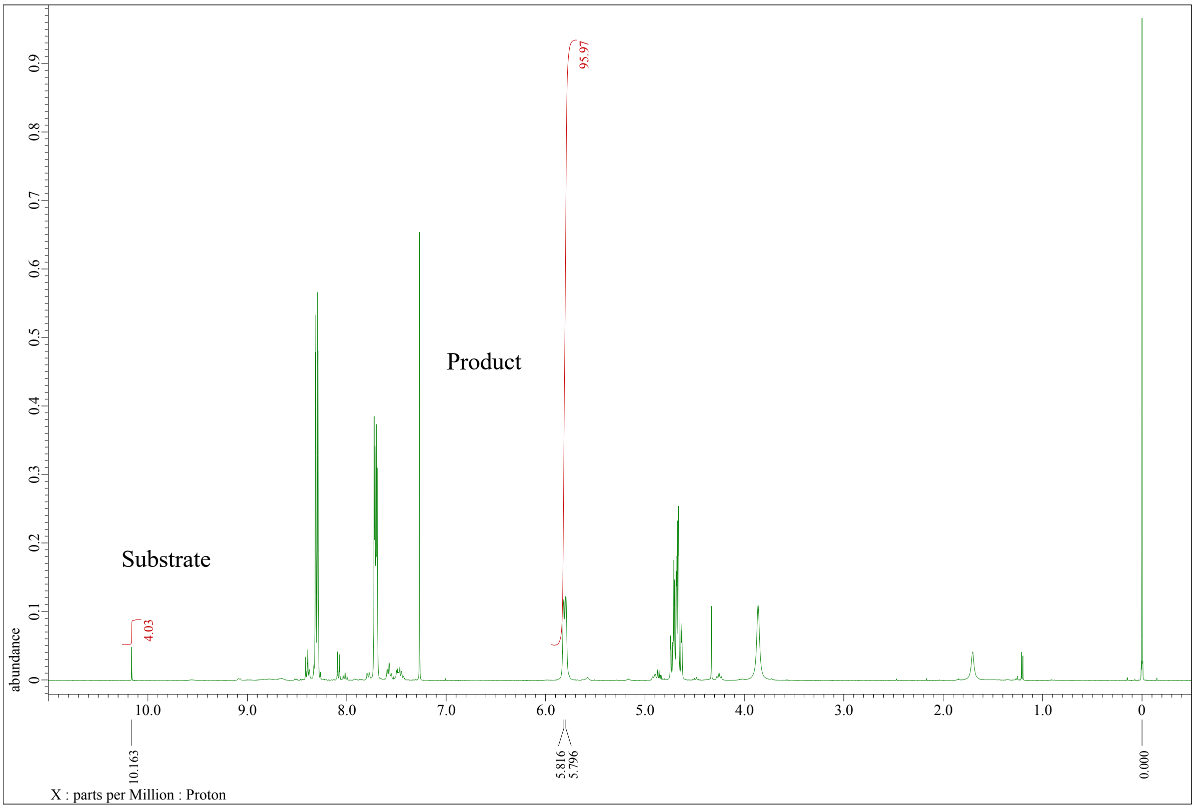

HPLC analysis

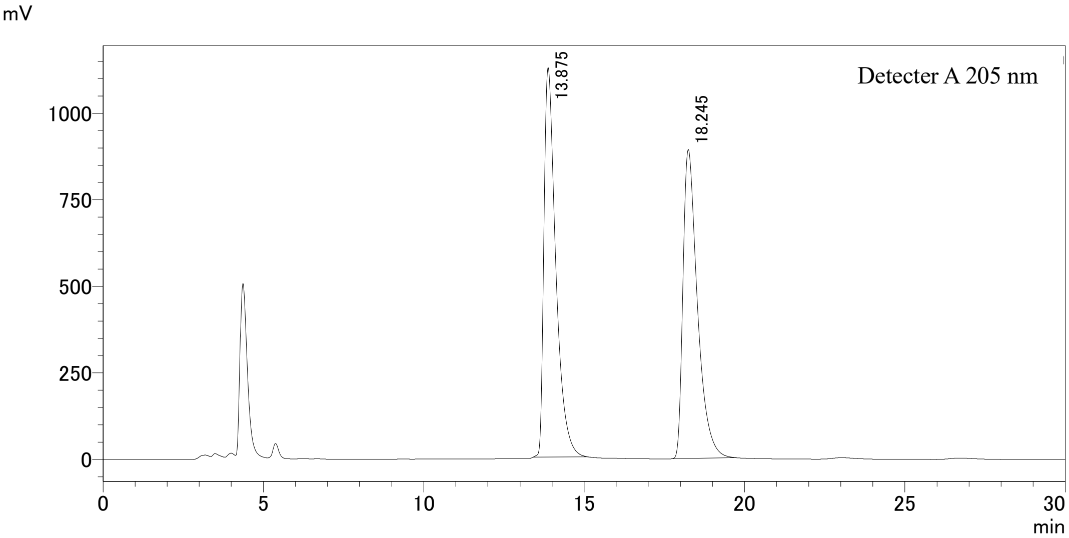

Detecter A 205 nm

Peak	Retemtion Time	Area	Height	Concentration
1	13.875	28950268	1124828	50.805
2	18.245	28032713	892691	49.195
Sum		56982981	2017519	

Conv.: 96%, 2% *ee*, The *ee* of product was determined by HPLC. [205 nm, Daicel chiralpack IA-3 column, hex:^{i}PrOH = 80:20, flow rate 1.0 mL/min, t_R = 18.25 min (minor), t_R = 13.88 min (major)]

Table 3, entry 5

^{1}H NMR, 400MHz, $CDCl_3$

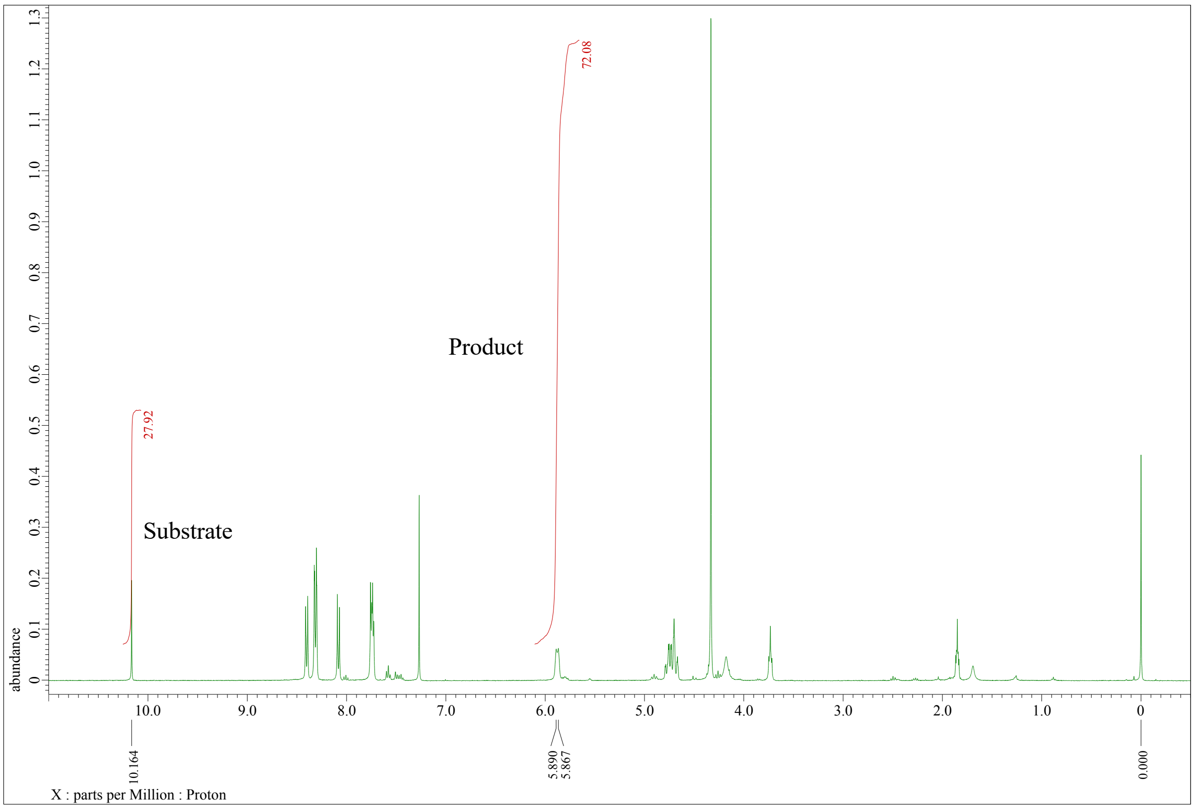

HPLC analysis

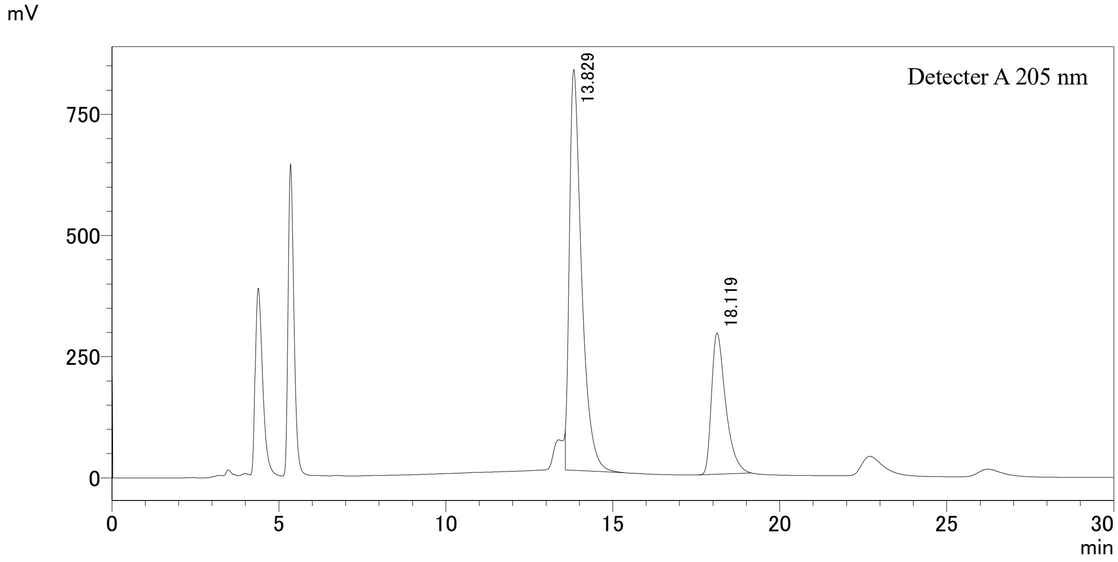

Detecter A 205 nm

Peak	Retemtion Time	Area	Height	Concentration
1	13.829	20987831	826920	70.964
2	18.119	8587481	291554	29.036
Sum		29575312	1118475	

Conv.: 72%, 42% *ee*, The *ee* of product was determined by HPLC. [205 nm, Daicel chiralpack IA-3 column, hex:^{i}PrOH = 80:20, flow rate 1.0 mL/min, t_R = 18.12 min (minor), t_R = 13.83 min (major)]

Table 3, entry 7

^{1}H NMR, 400MHz, $CDCl_3$

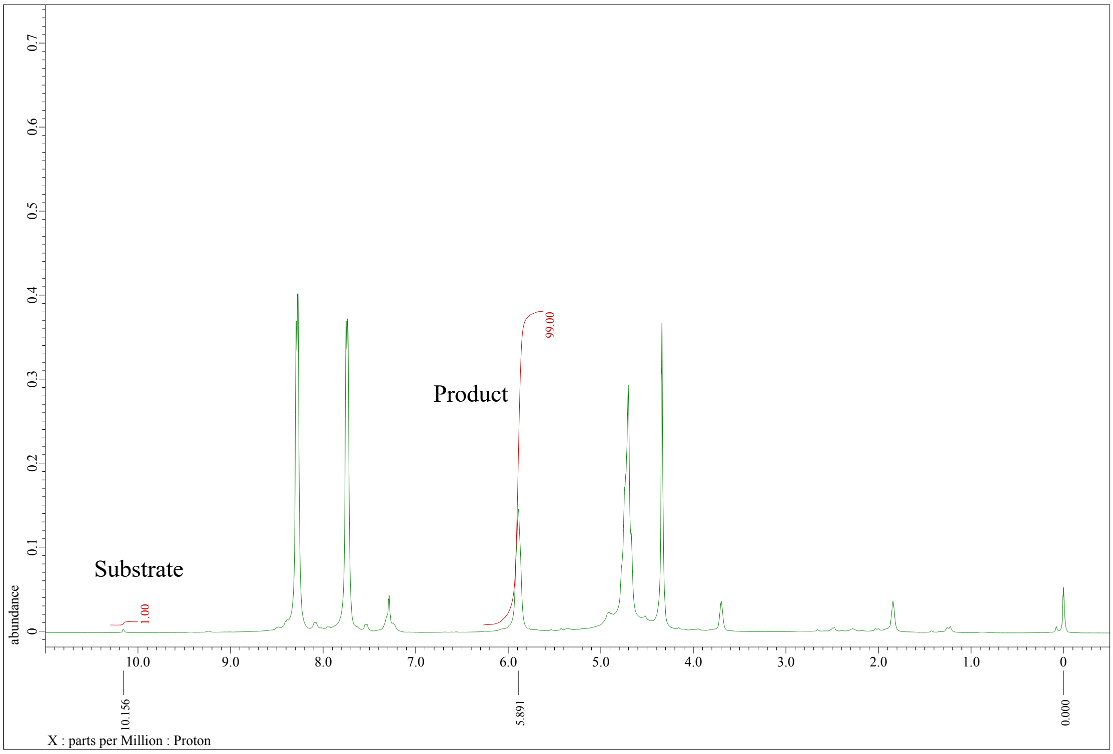

HPLC analysis

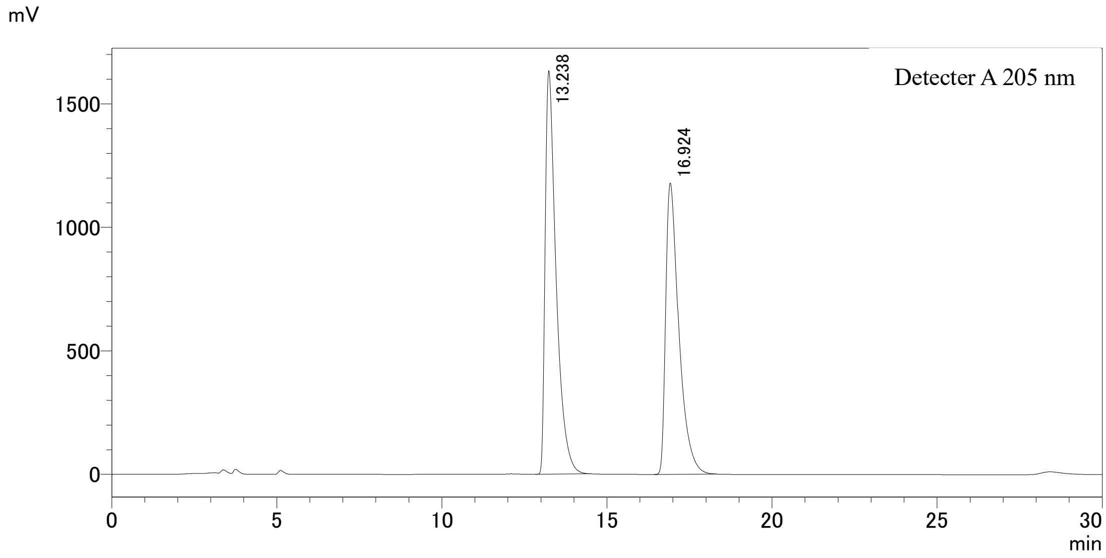

Detecter A 205 nm

Peak	Retemtion Time	Area	Height	Concentration
1	13.238	37709034	1617845	53.743
2	16.924	32456169	1177910	46.257
Sum		70165203	2795755	

Conv.: 99%, 8% *ee*, The *ee* of product was determined by HPLC. [205 nm, Daicel chiralpack IA-3 column, hex:^{i}PrOH = 80:20, flow rate 1.0 mL/min, t_R = 16.92 min (minor), t_R = 13.24 min (major)]

2. ^{1}H NMR, ^{19}F NMR, ^{13}C NMR spectra of compounds

^{1}H NMR, 400 MHz, $CDCl_3$

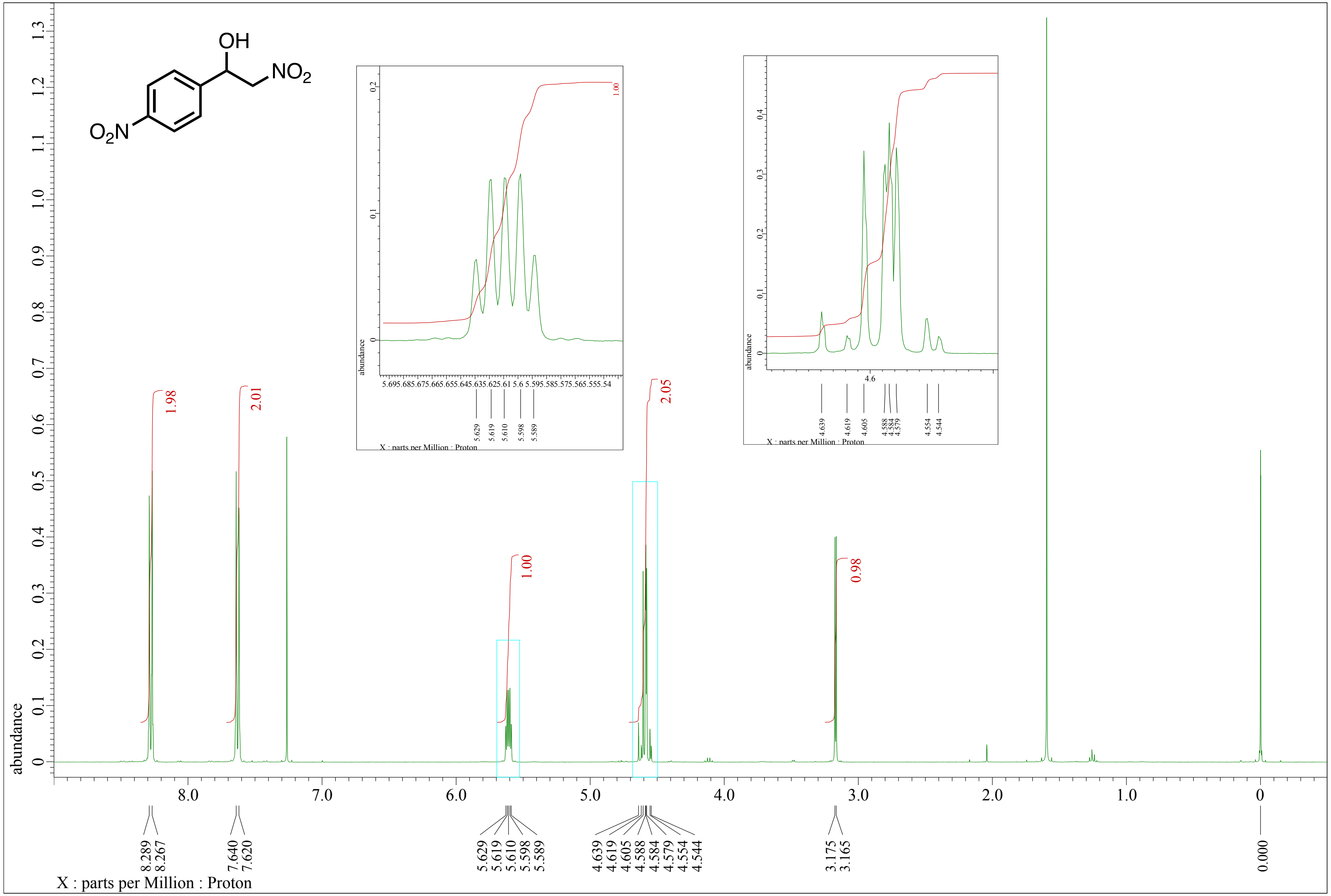

^{1}H NMR, 400 MHz $(CD_3)_2SO$

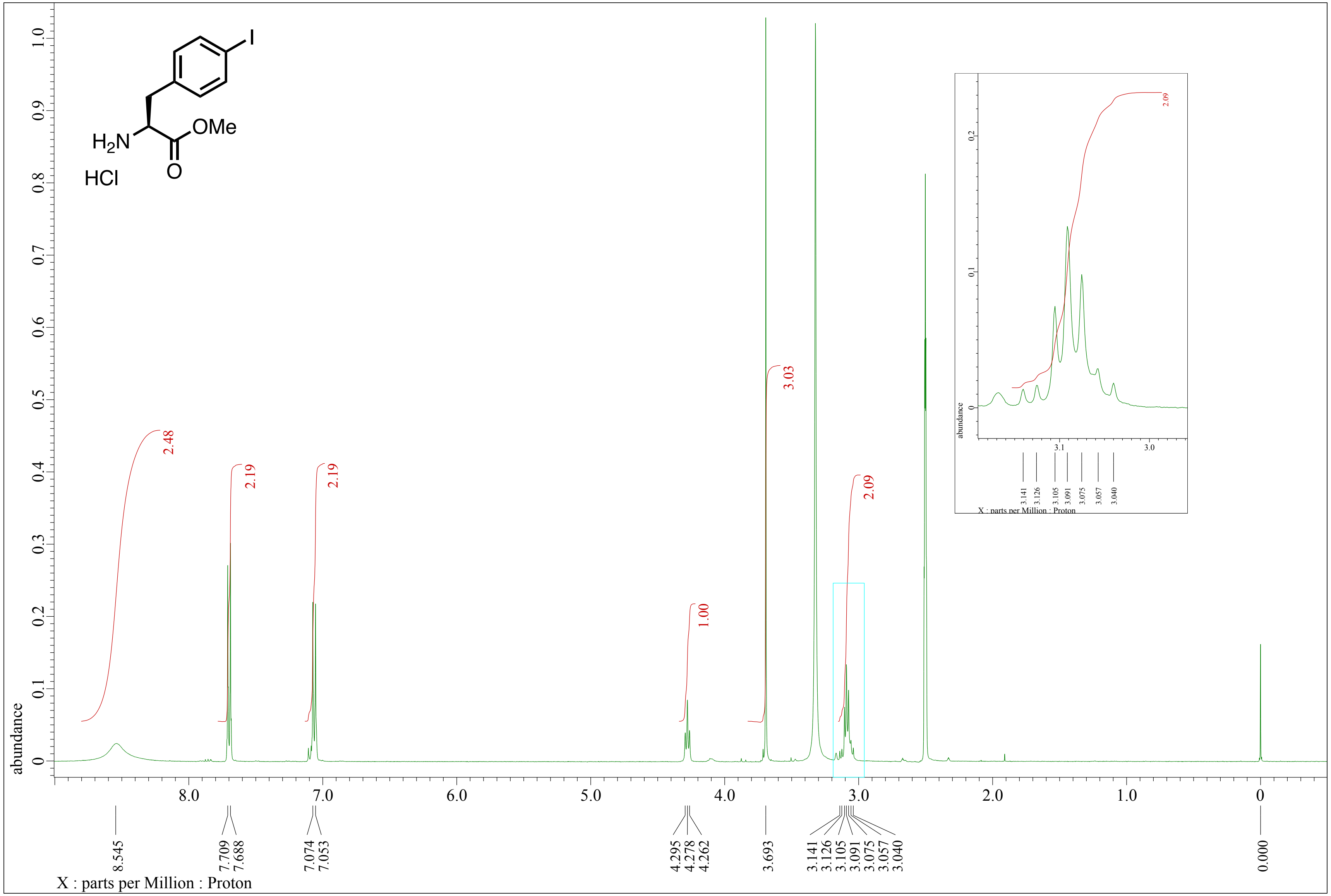

^{1}H NMR, 400 MHz, $CDCl_3$

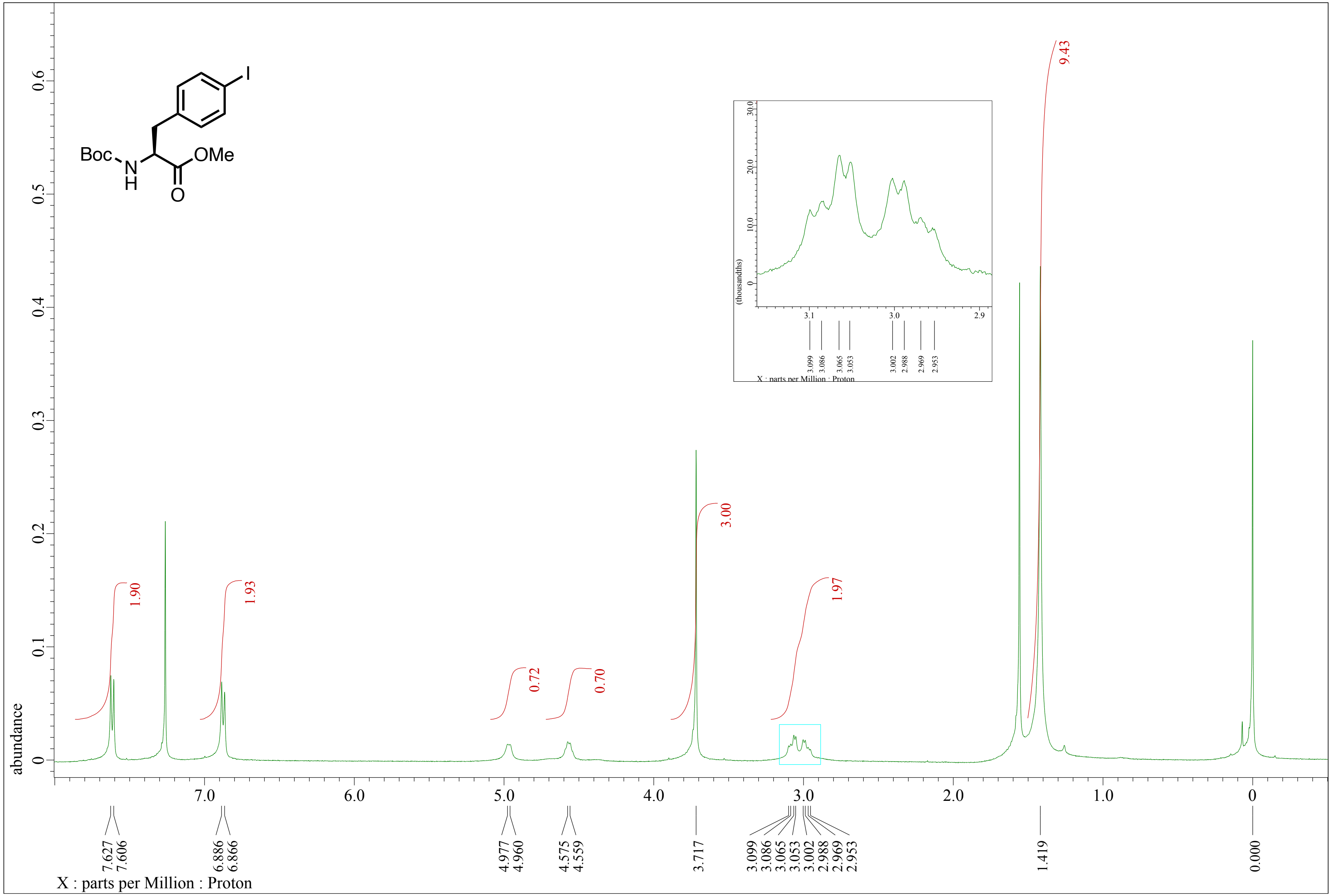

^1H NMR, 270 MHz, $CDCl_3$

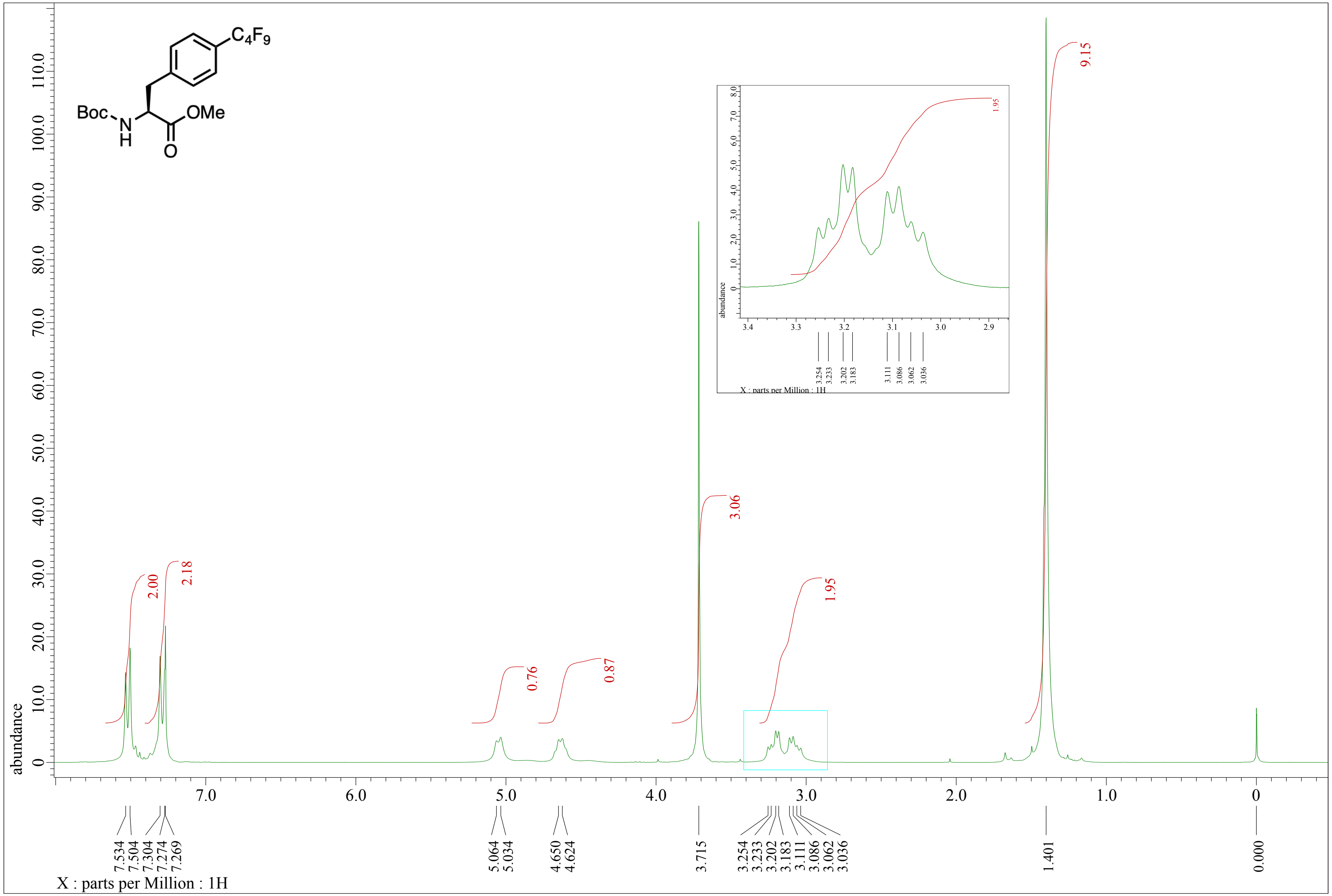

^{19}F NMR, 376 MHz, $CDCl_3$

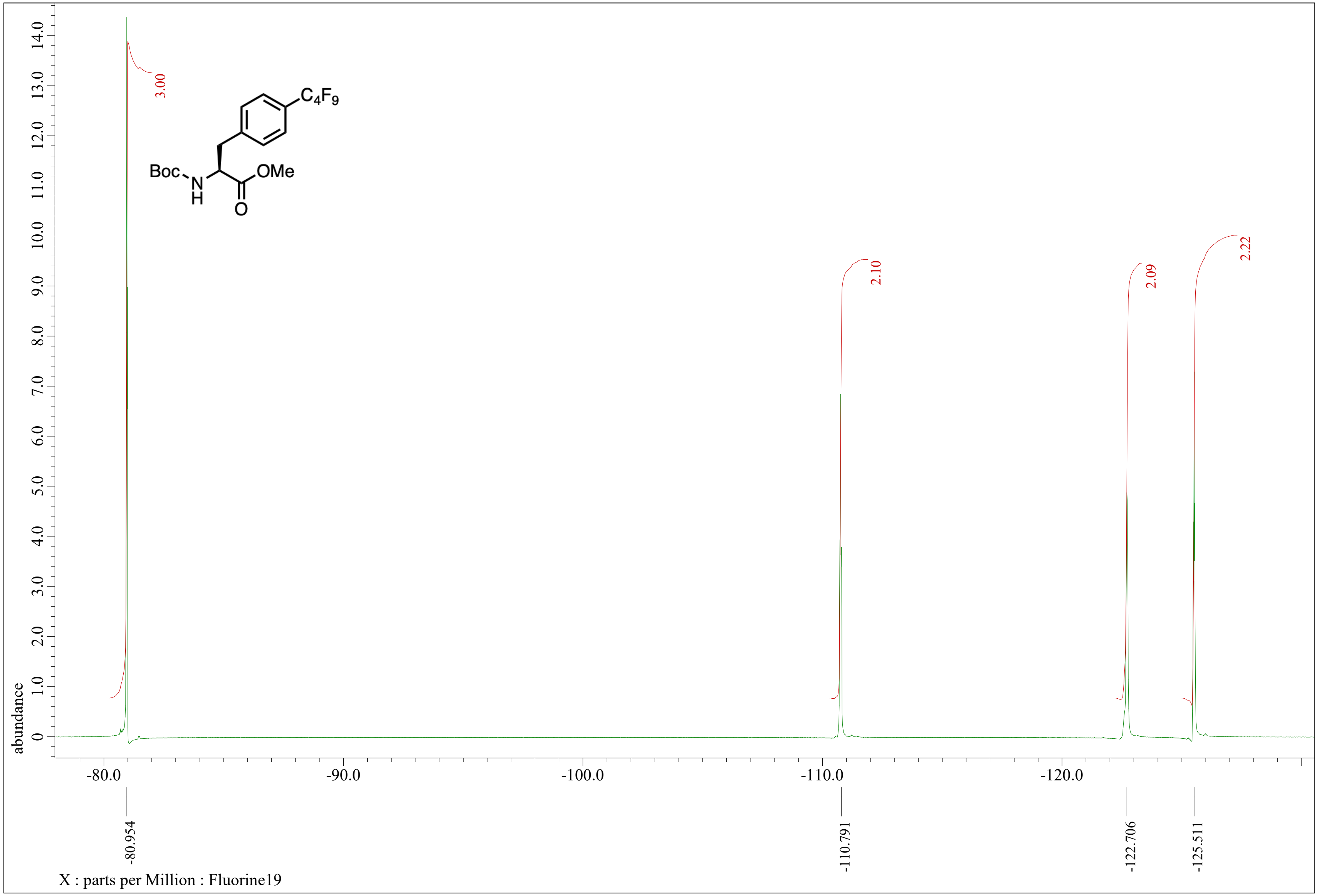

^{13}C NMR, 101 MHz, $CDCl_3$

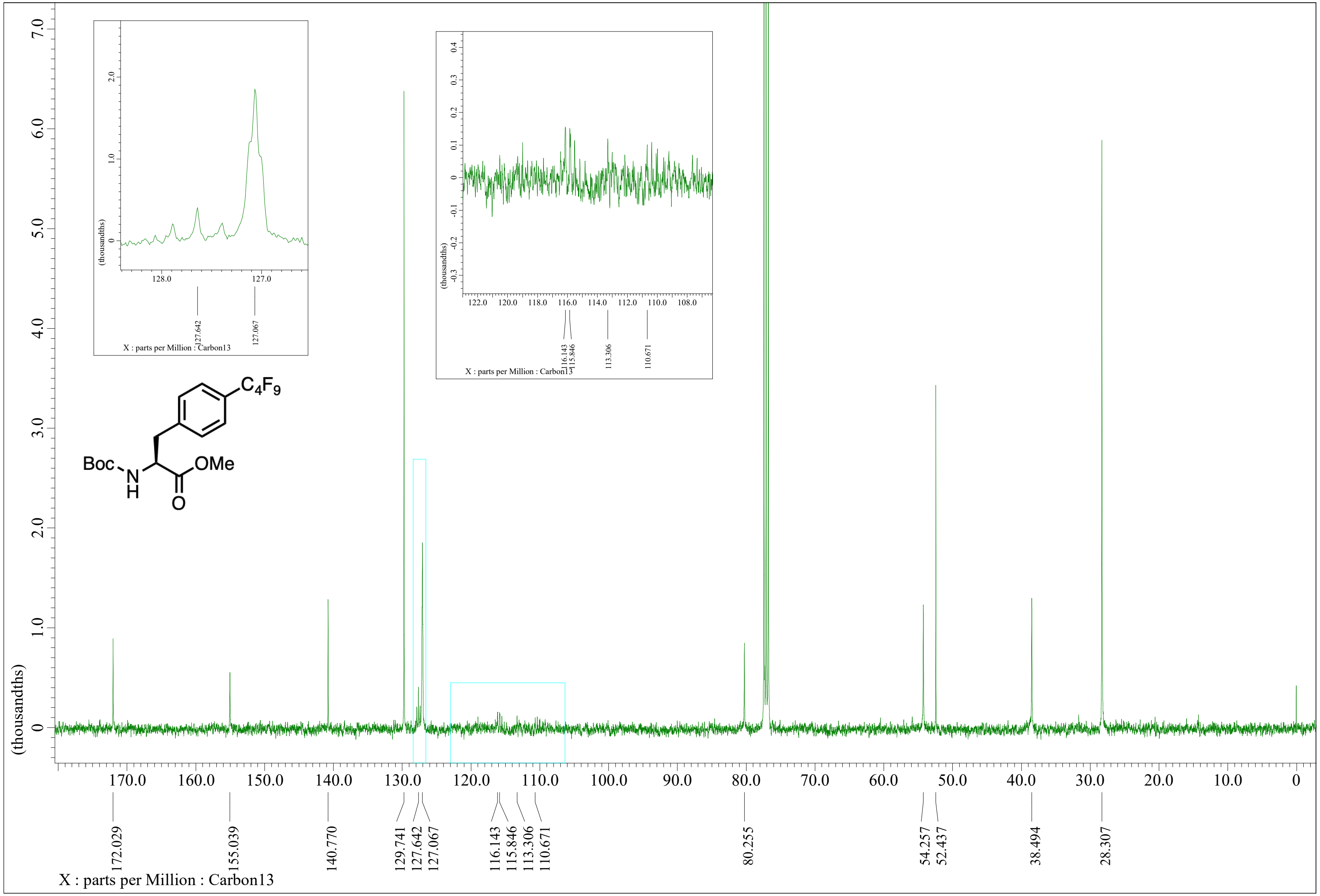

^{1}H NMR, 400 MHz, $CDCl_3$

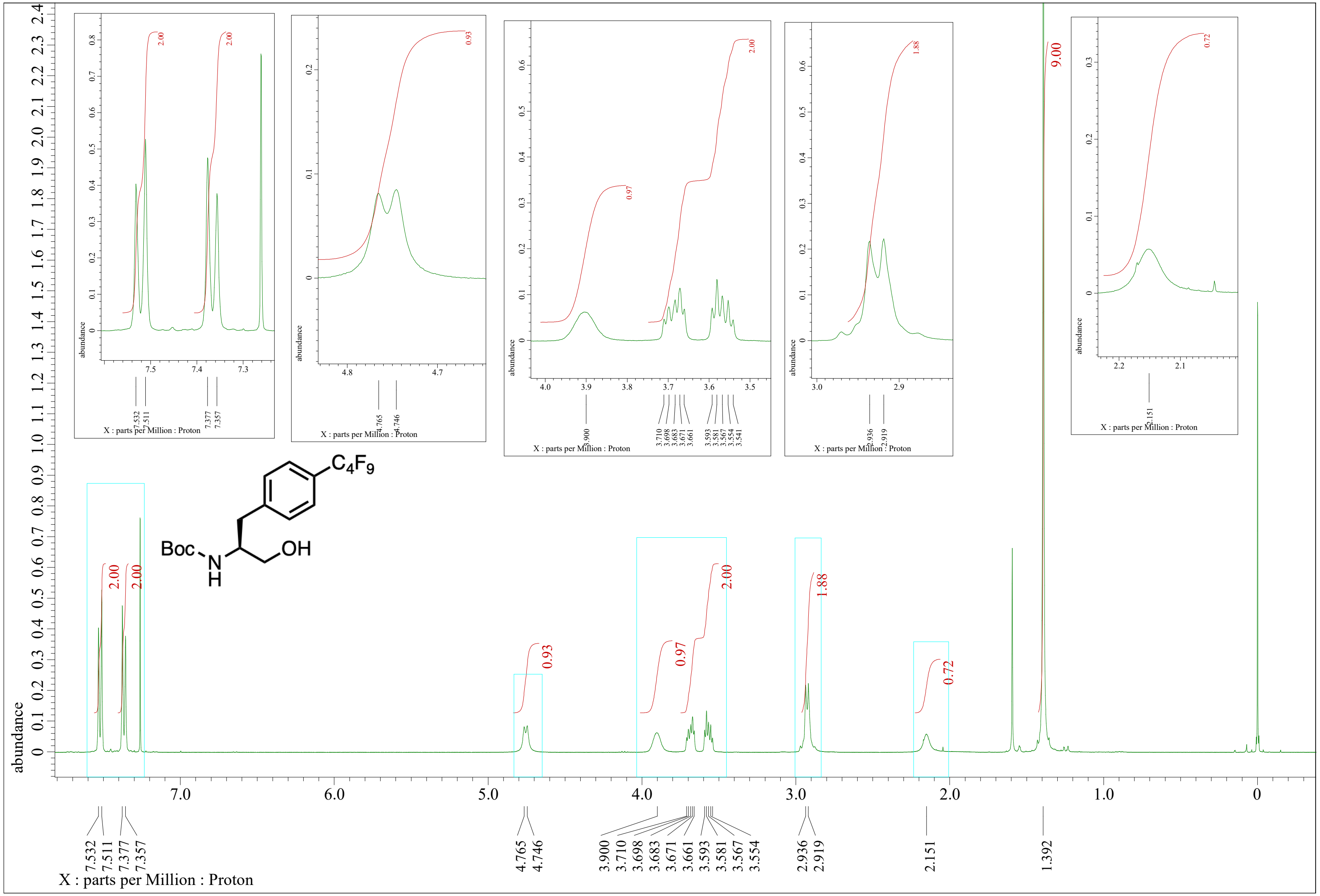

^{19}F NMR, 376 MHz, $CDCl_3$

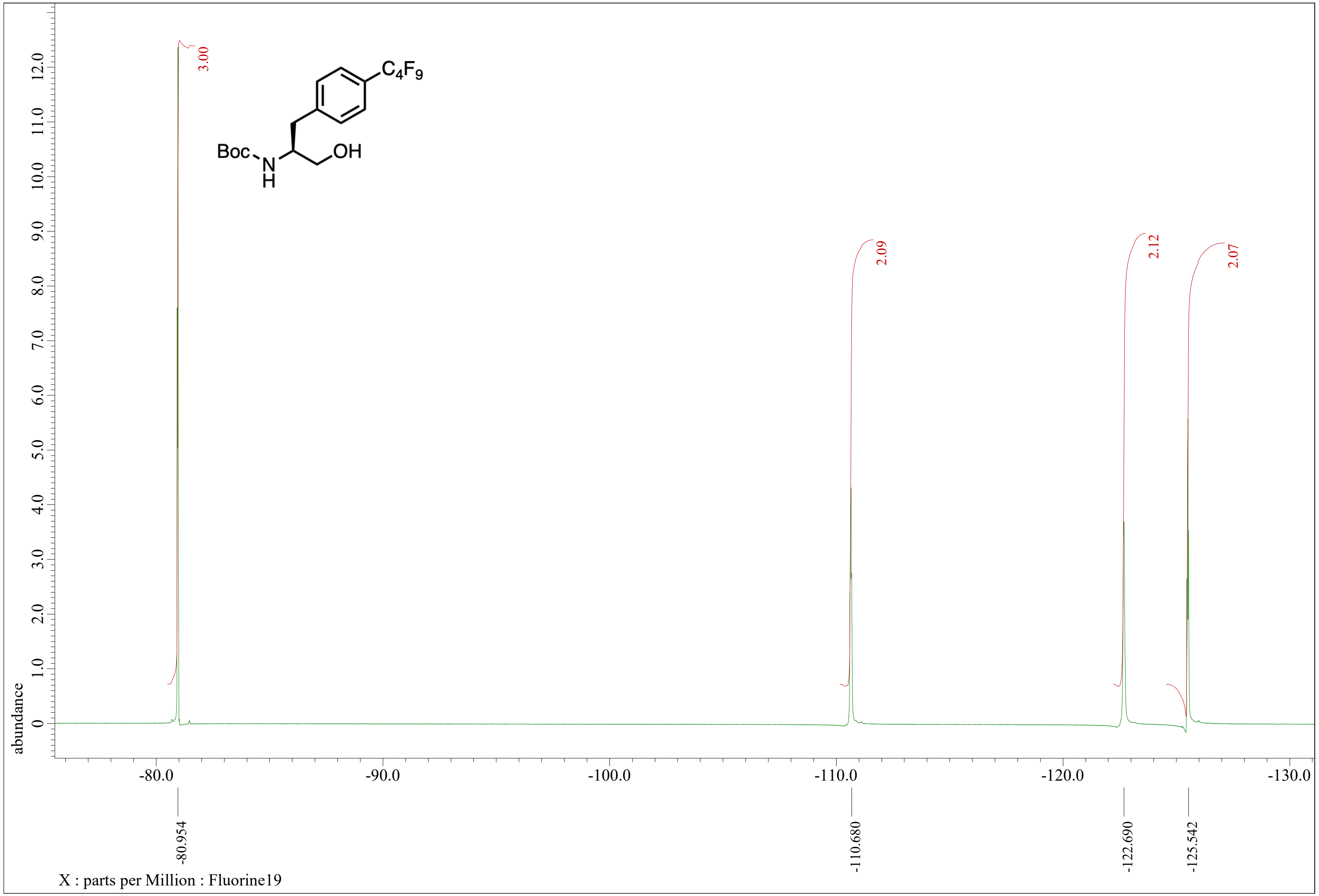

^{13}C NMR, 101 MHz, $CDCl_3$

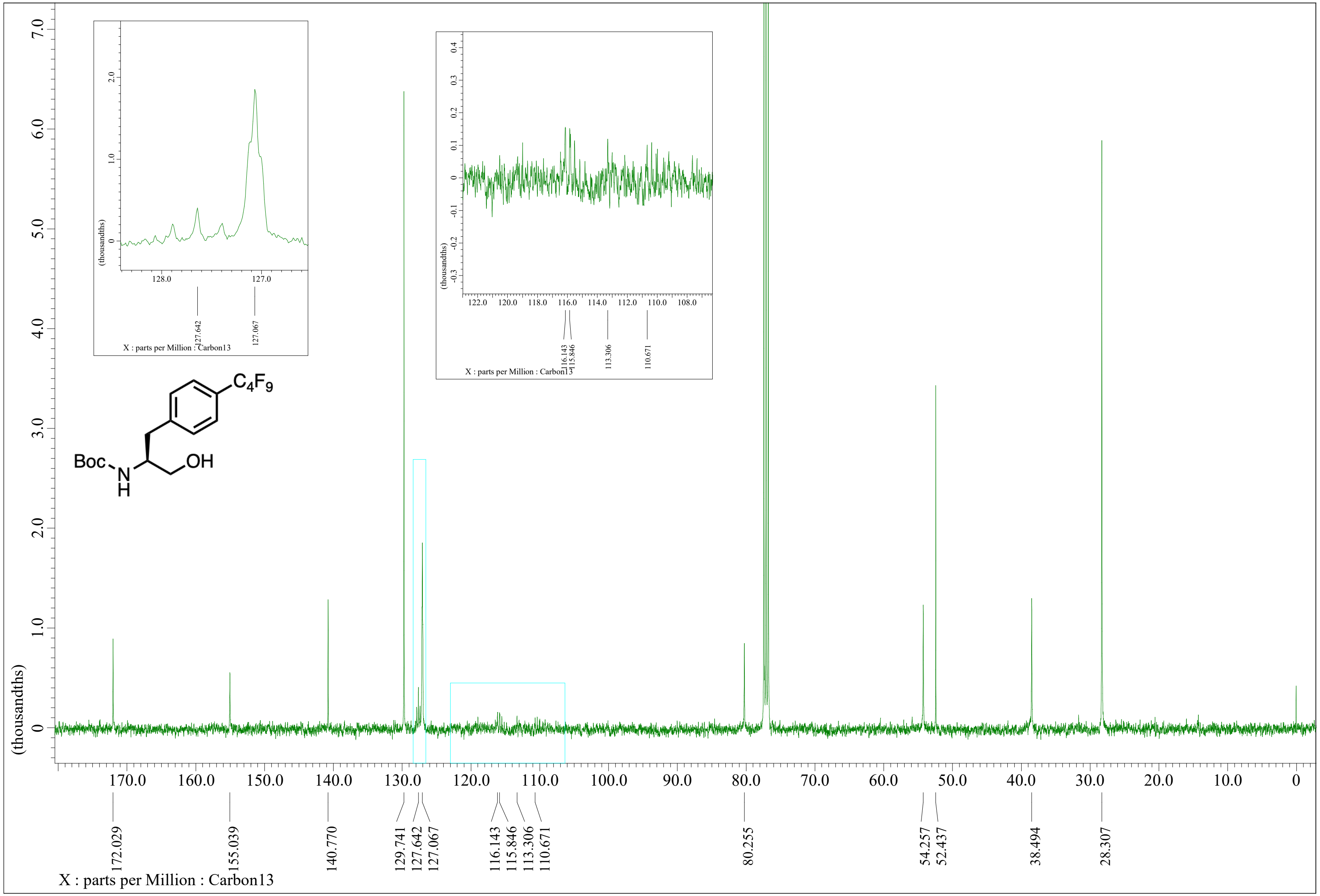

^{1}H NMR, 400 MHz, $(CD_3)_2SO$

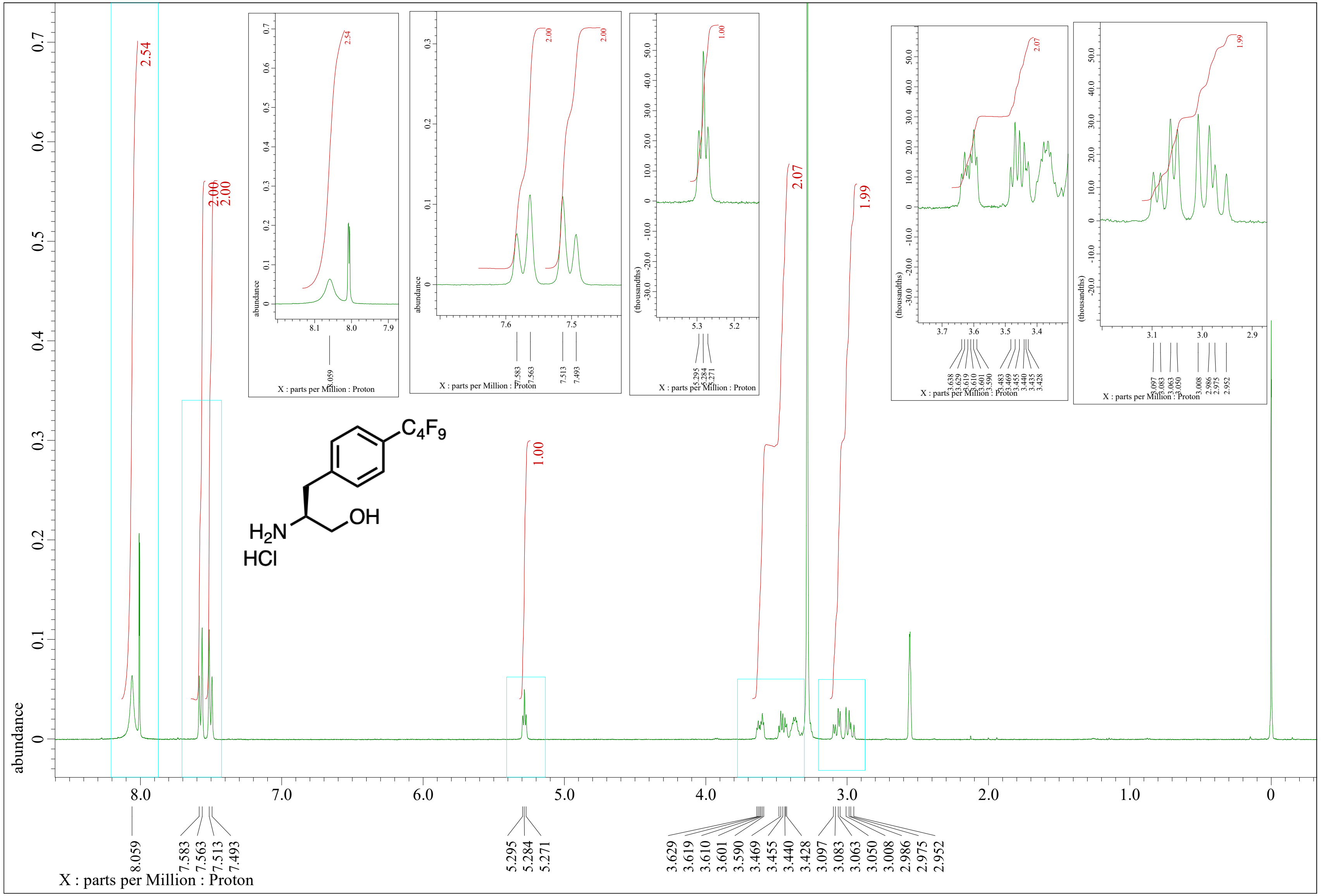

^{19}F NMR, 376 MHz, $(CD_3)_2SO$

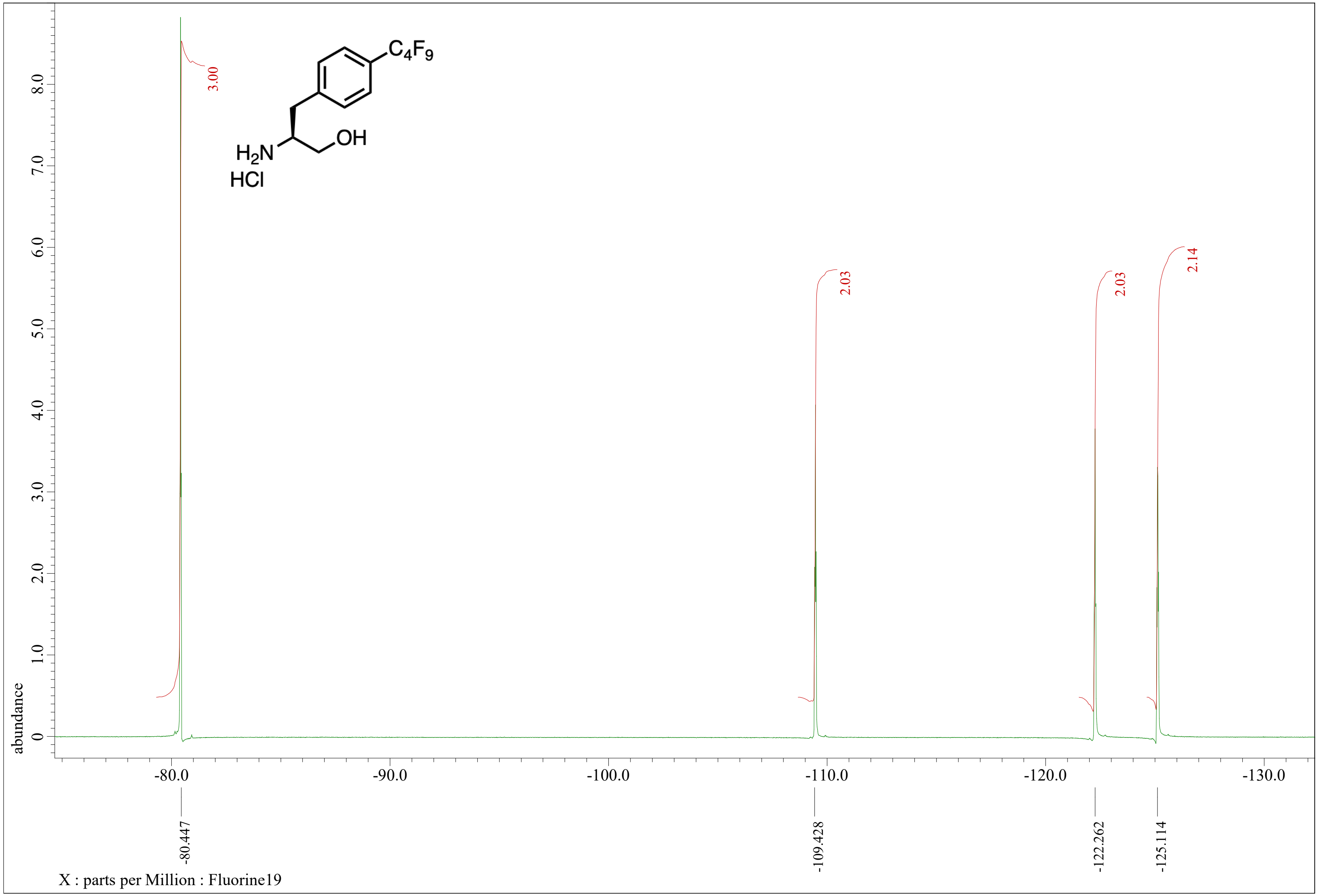

^{13}C NMR, 101 MHz, $(CD_3)_2SO$

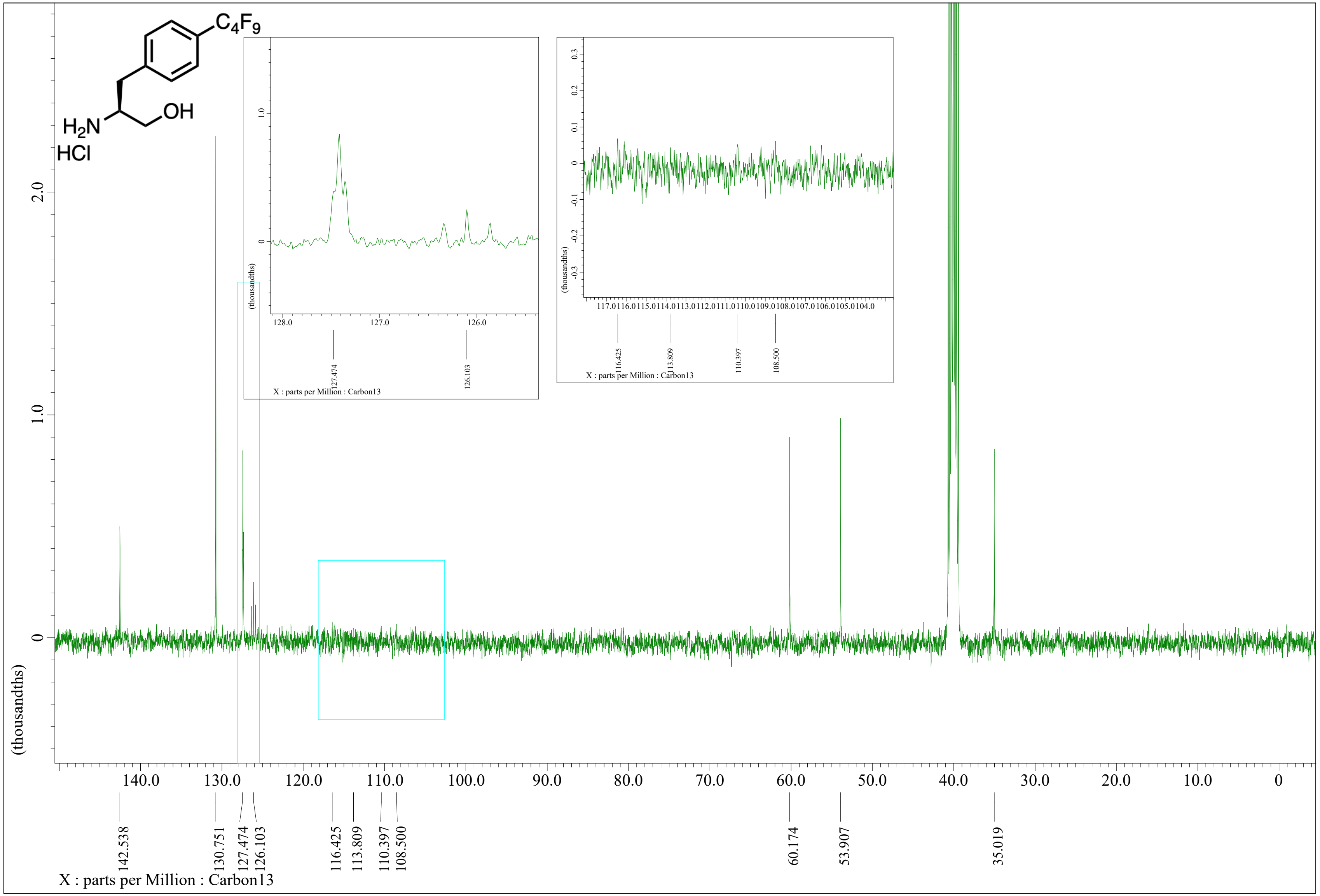

^{1}H NMR, 270 MHz, $CDCl_3$

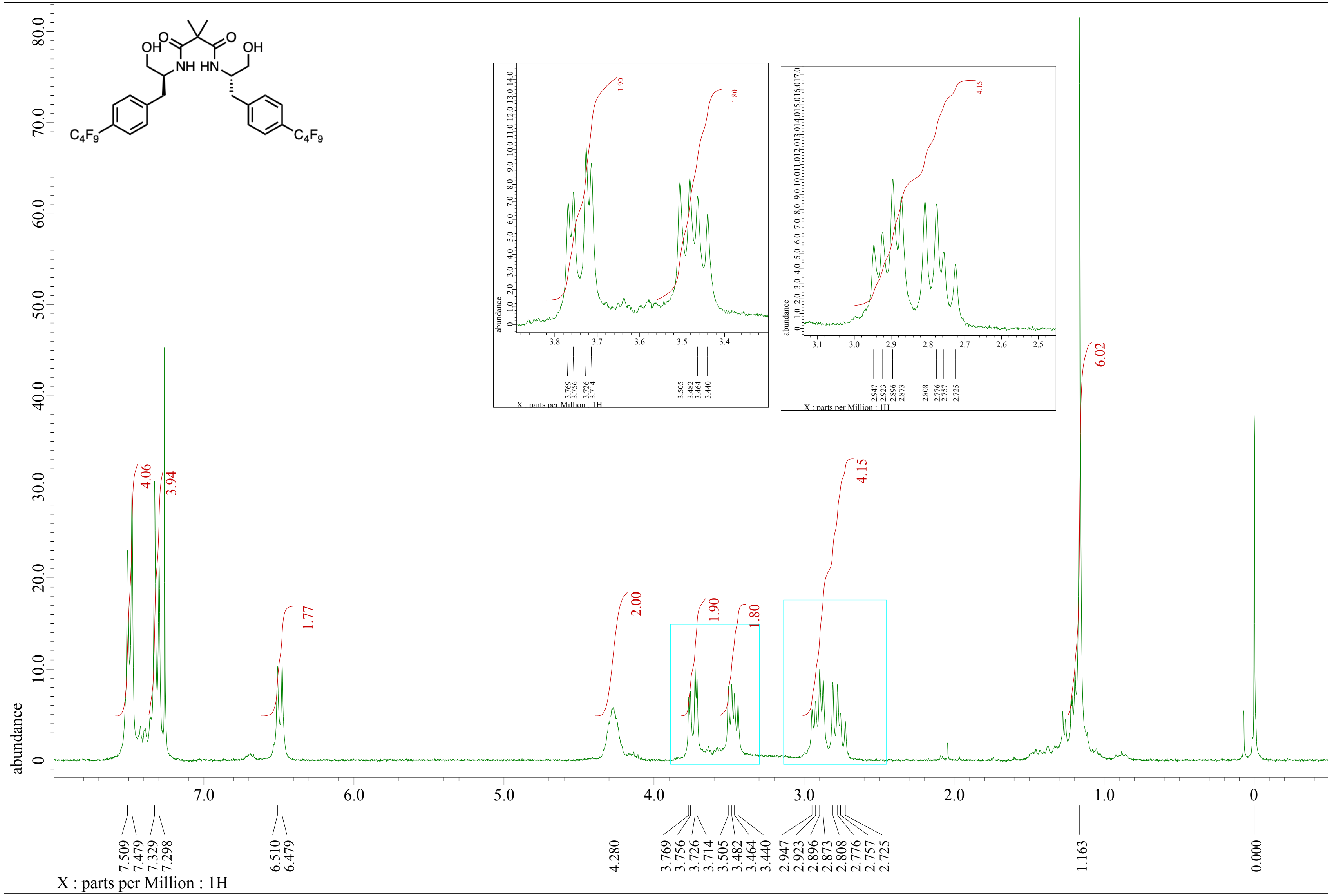

^{19}F NMR, 376 MHz, $CDCl_3$

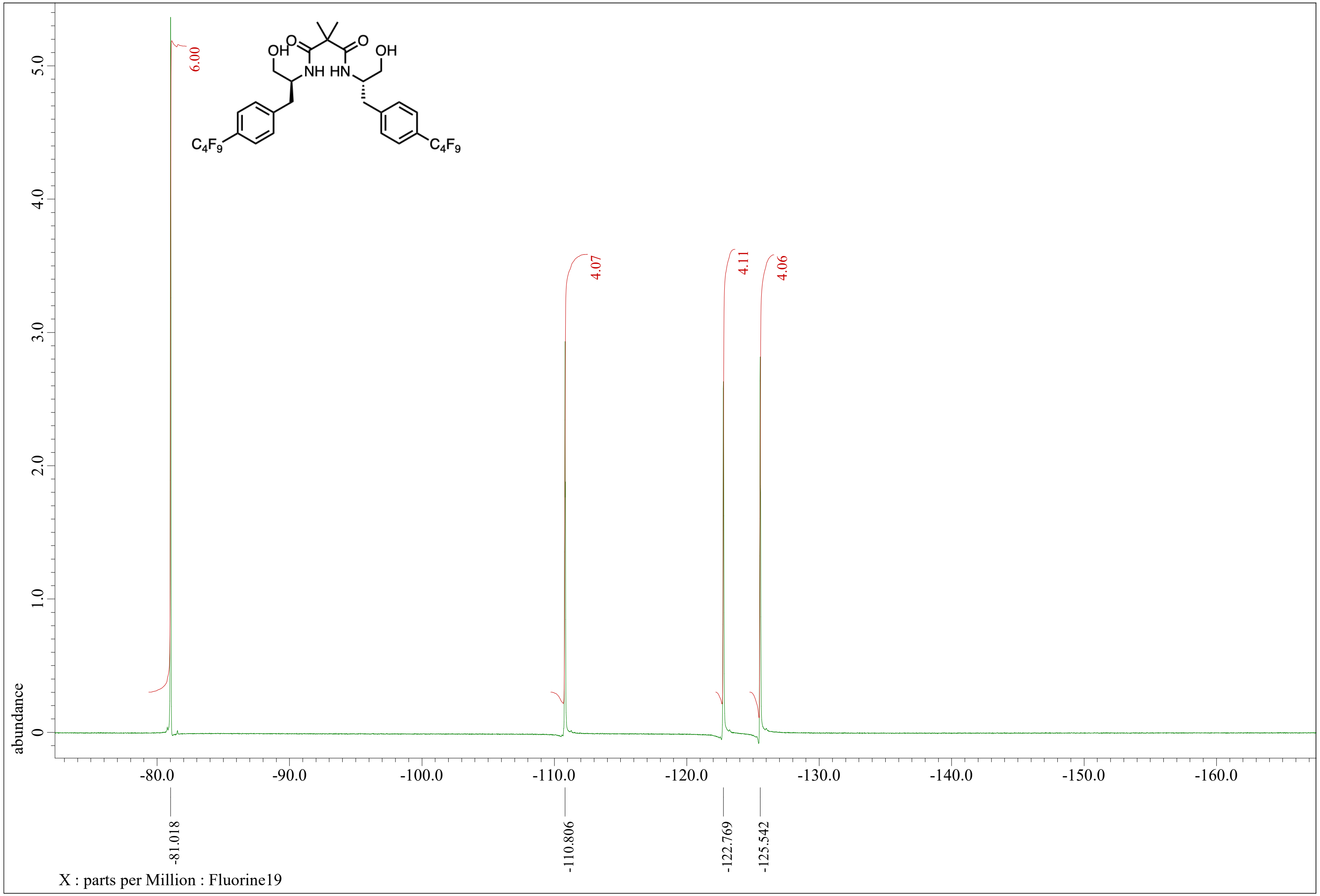

^{13}C NMR, 101 MHz, $CDCl_3$

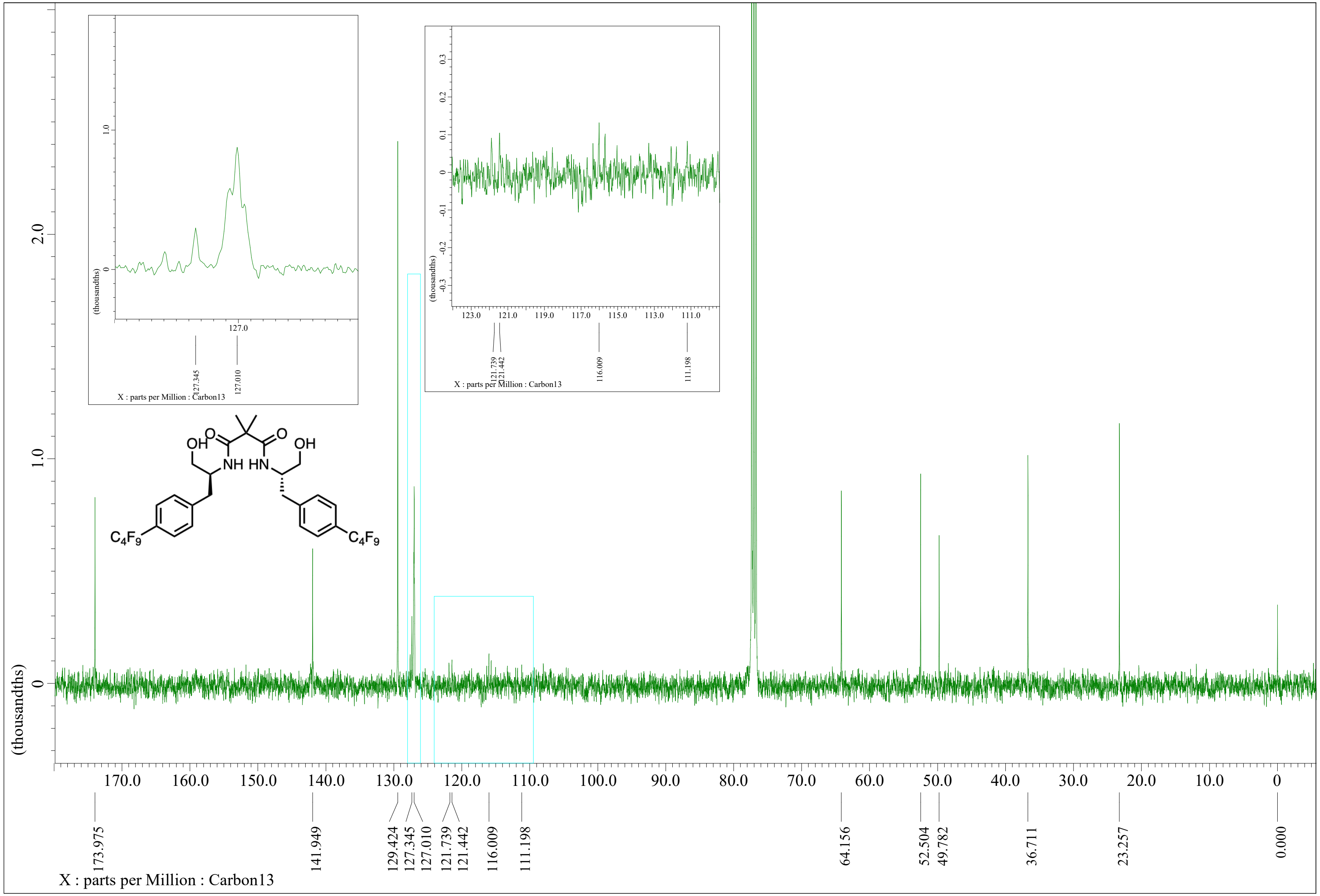

^{1}H NMR, 400 MHz, $CDCl_3$

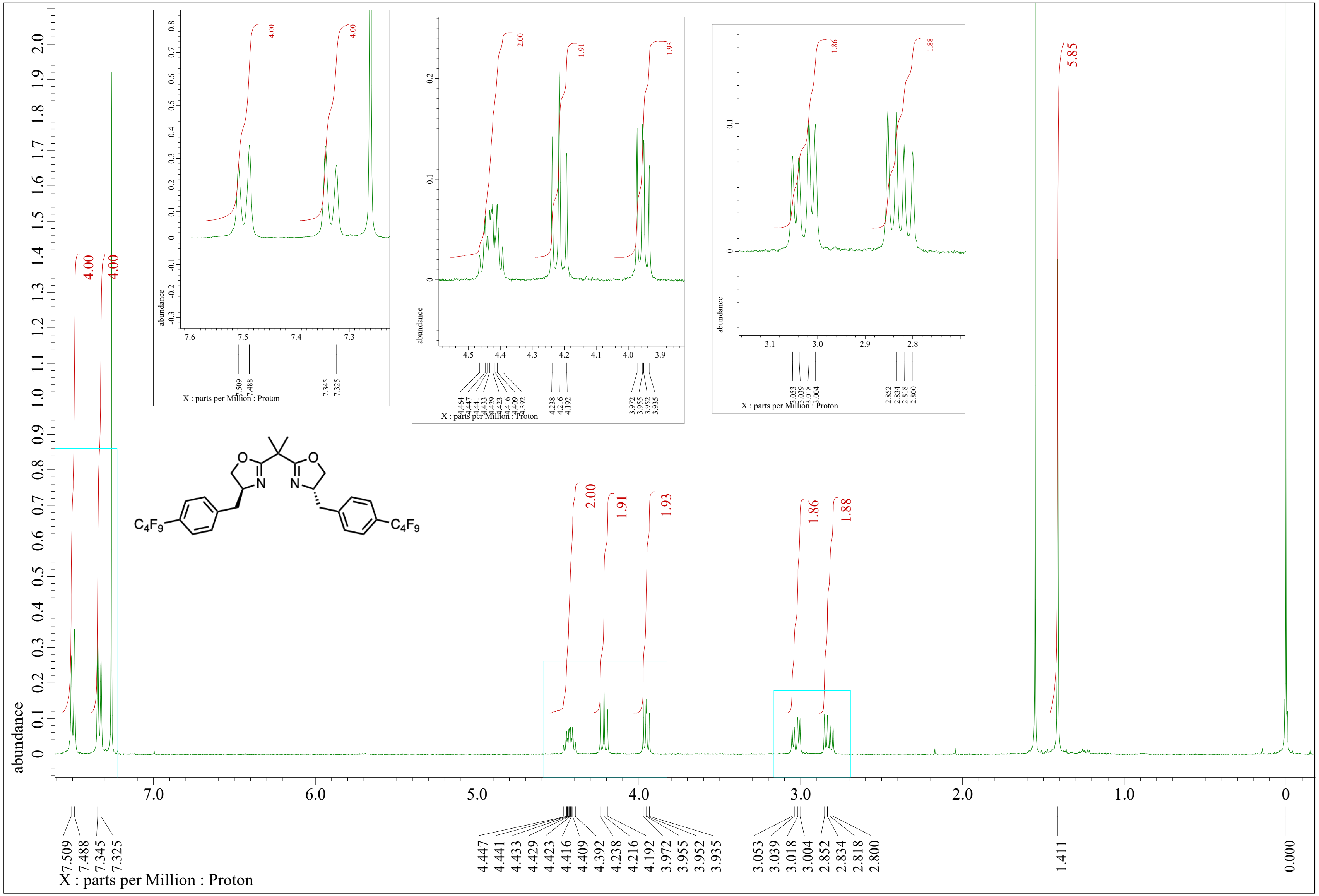

^{19}F NMR, 376 MHz, CDCl$_3$

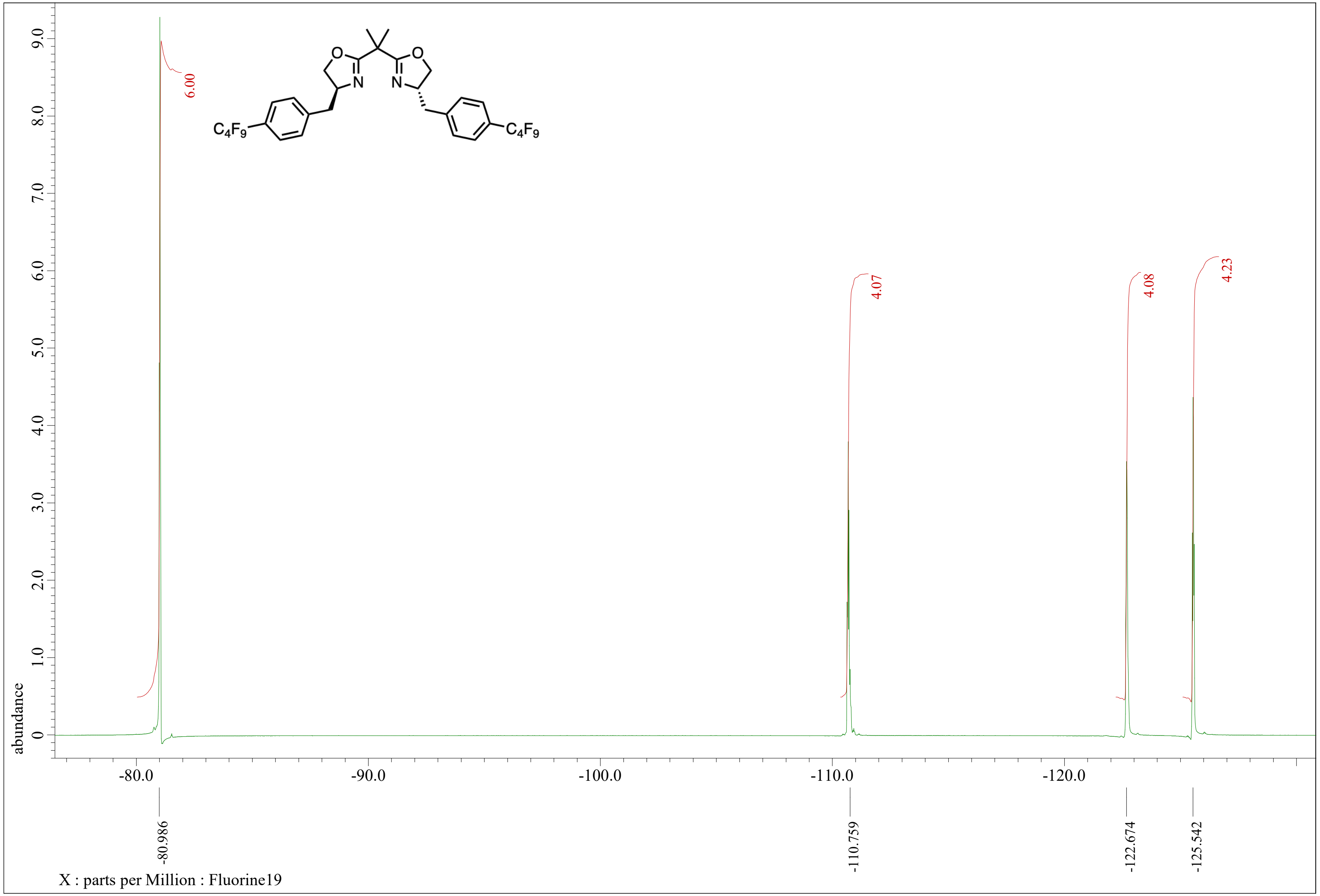

^{13}C NMR, 101 MHz, $CDCl_3$

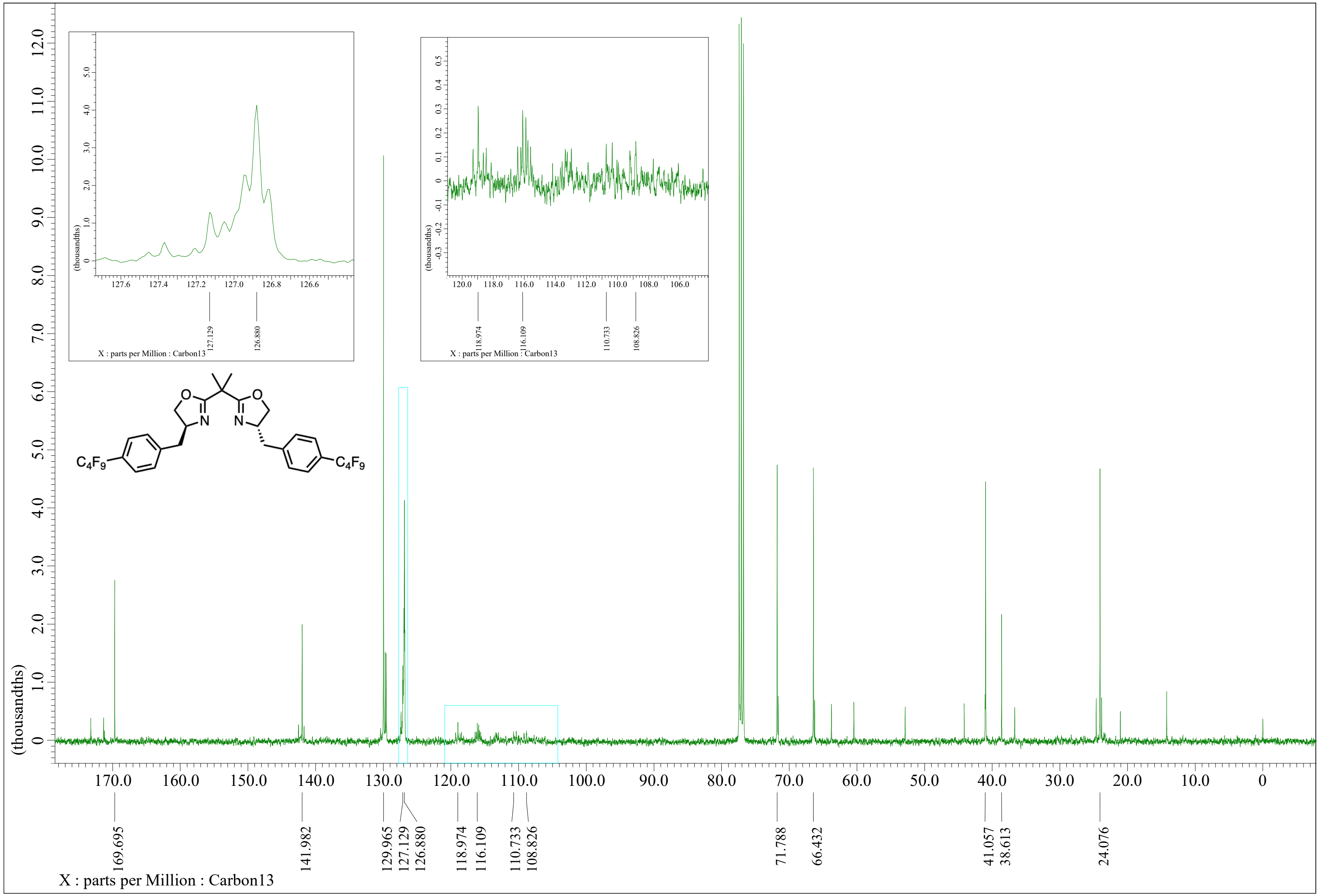

^{1}H NMR, 400 MHz, $CDCl_3$

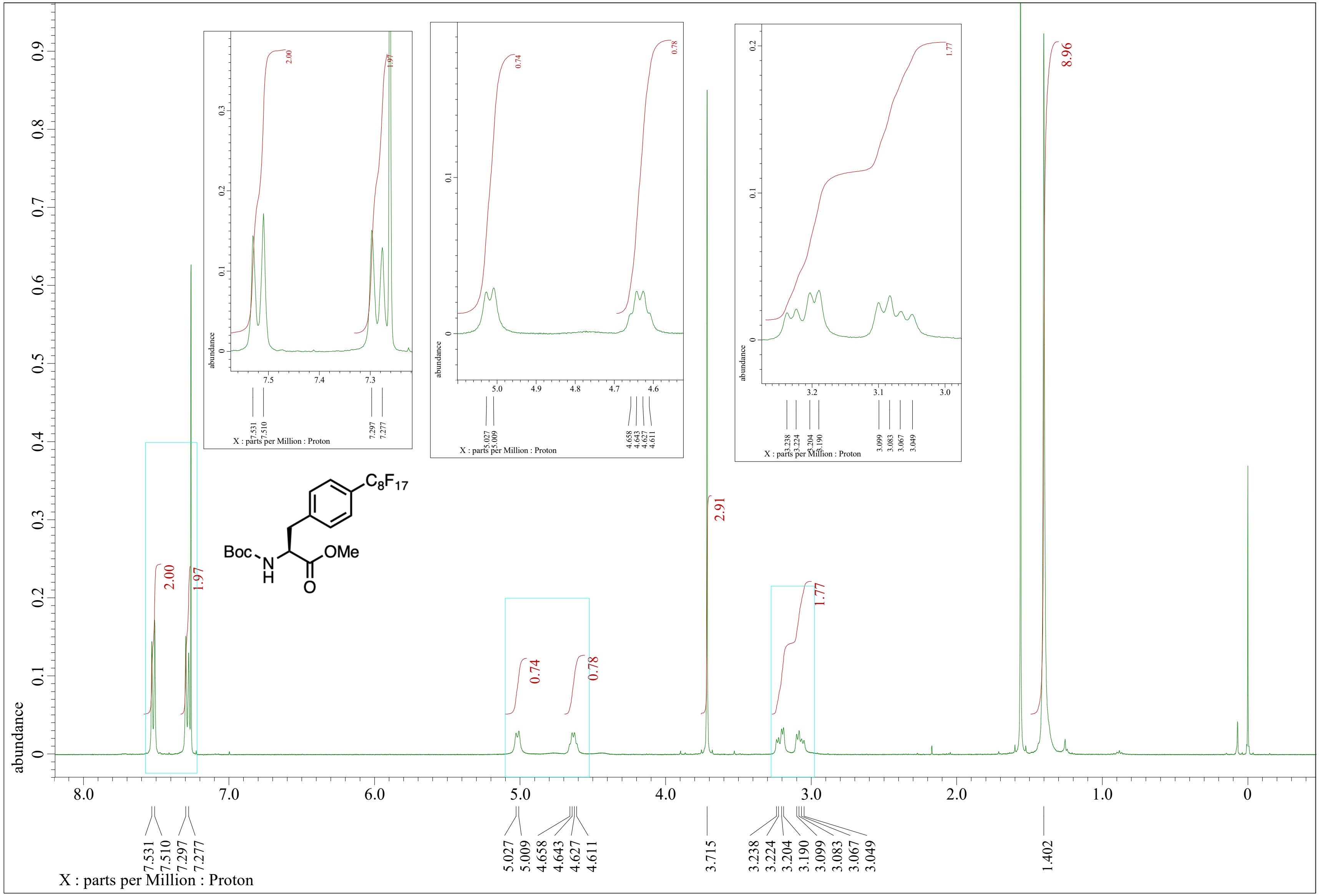

^{19}F NMR, 376 MHz, $CDCl_3$

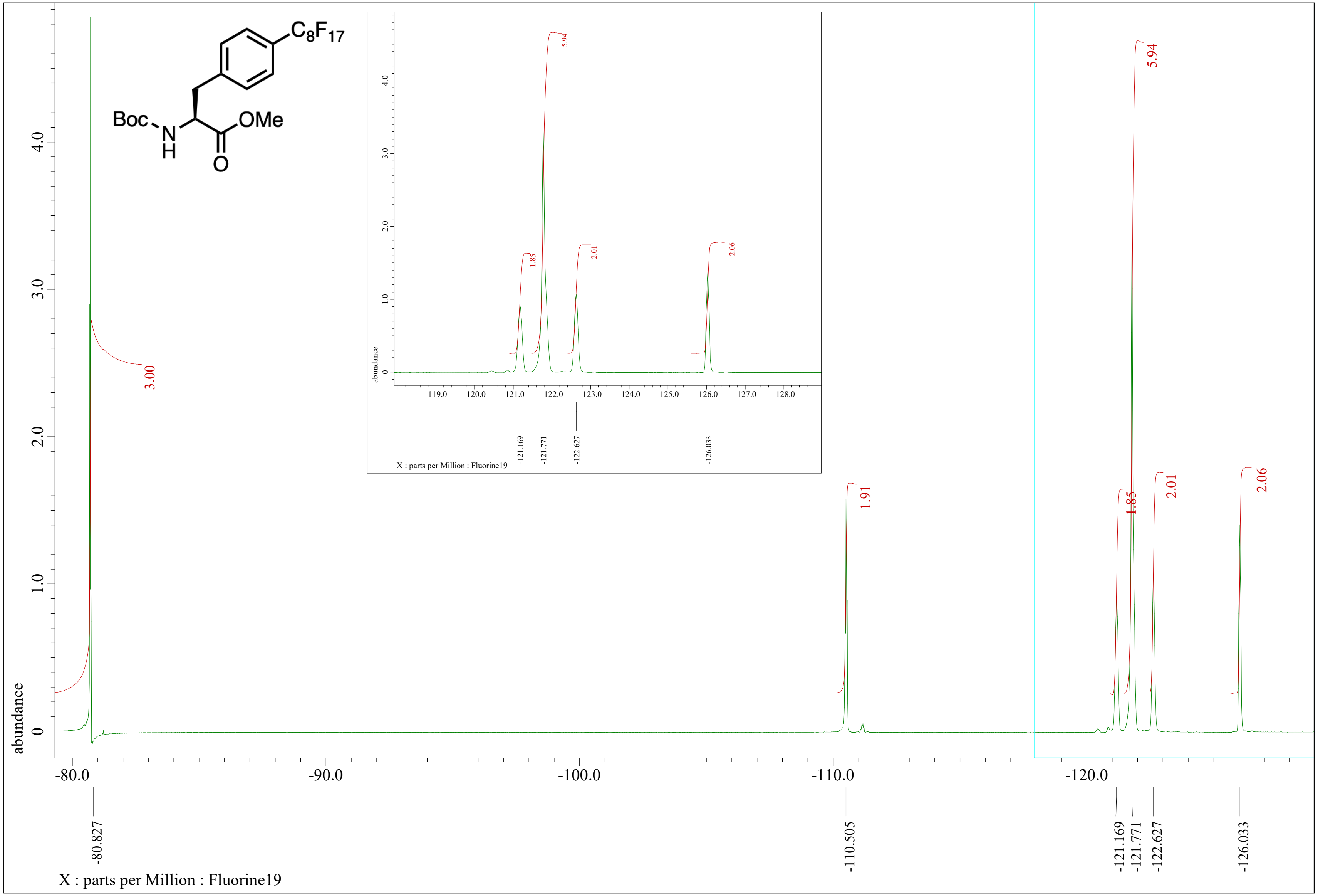

^{13}C NMR, 101 MHz, $CDCl_3$

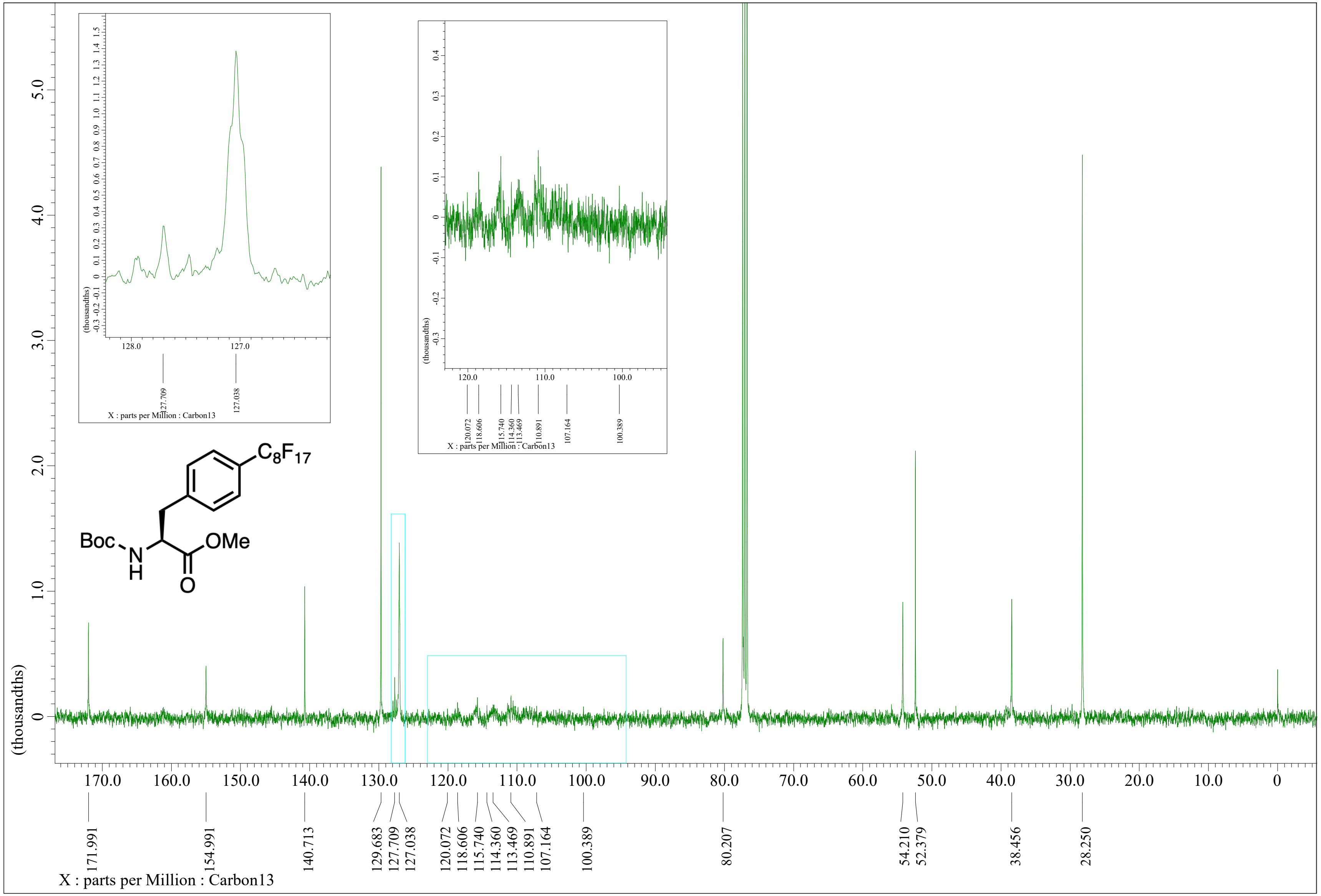

^{1}H NMR, 400 MHz, $CDCl_3$

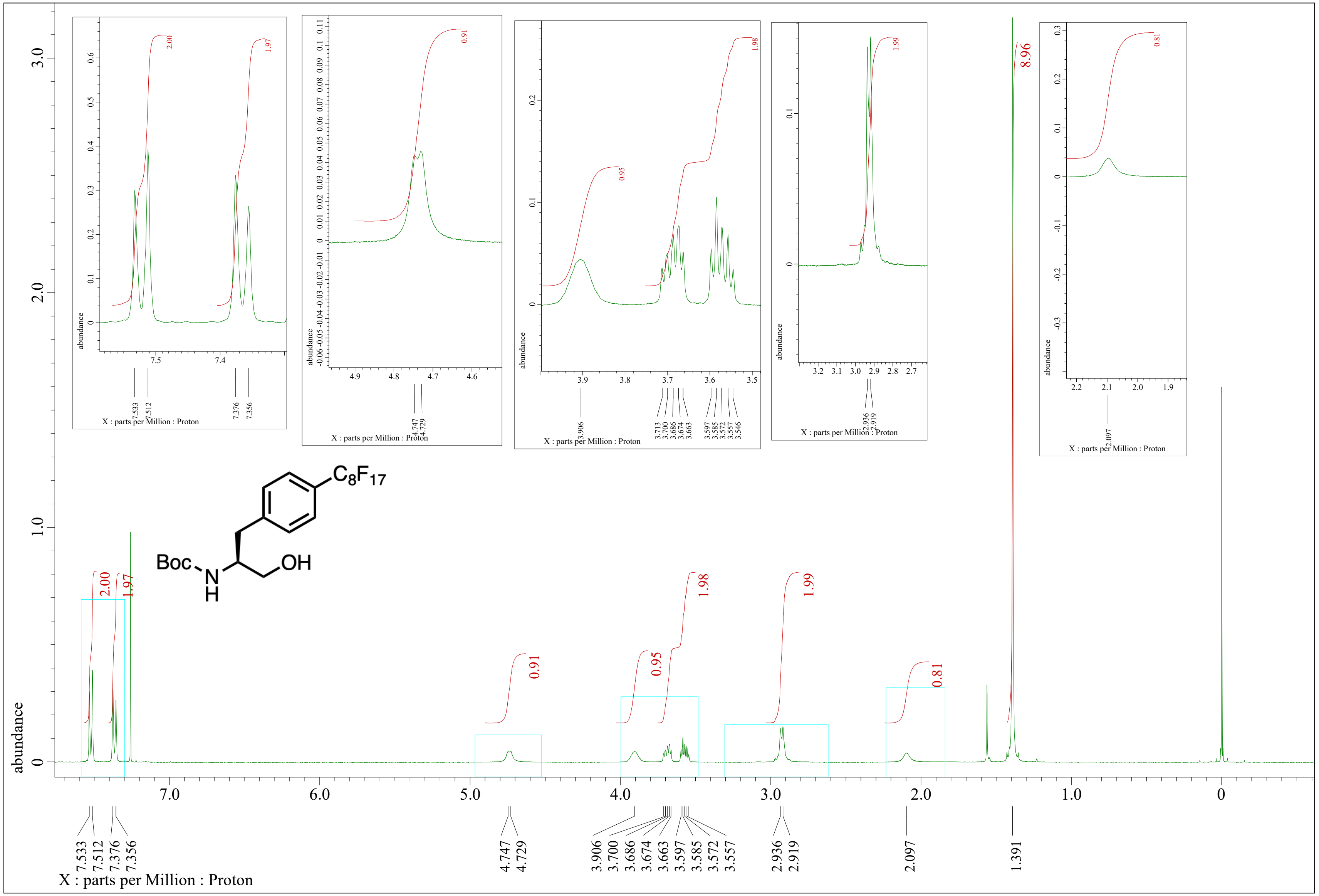

^{19}F NMR, 376 MHz, $CDCl_3$

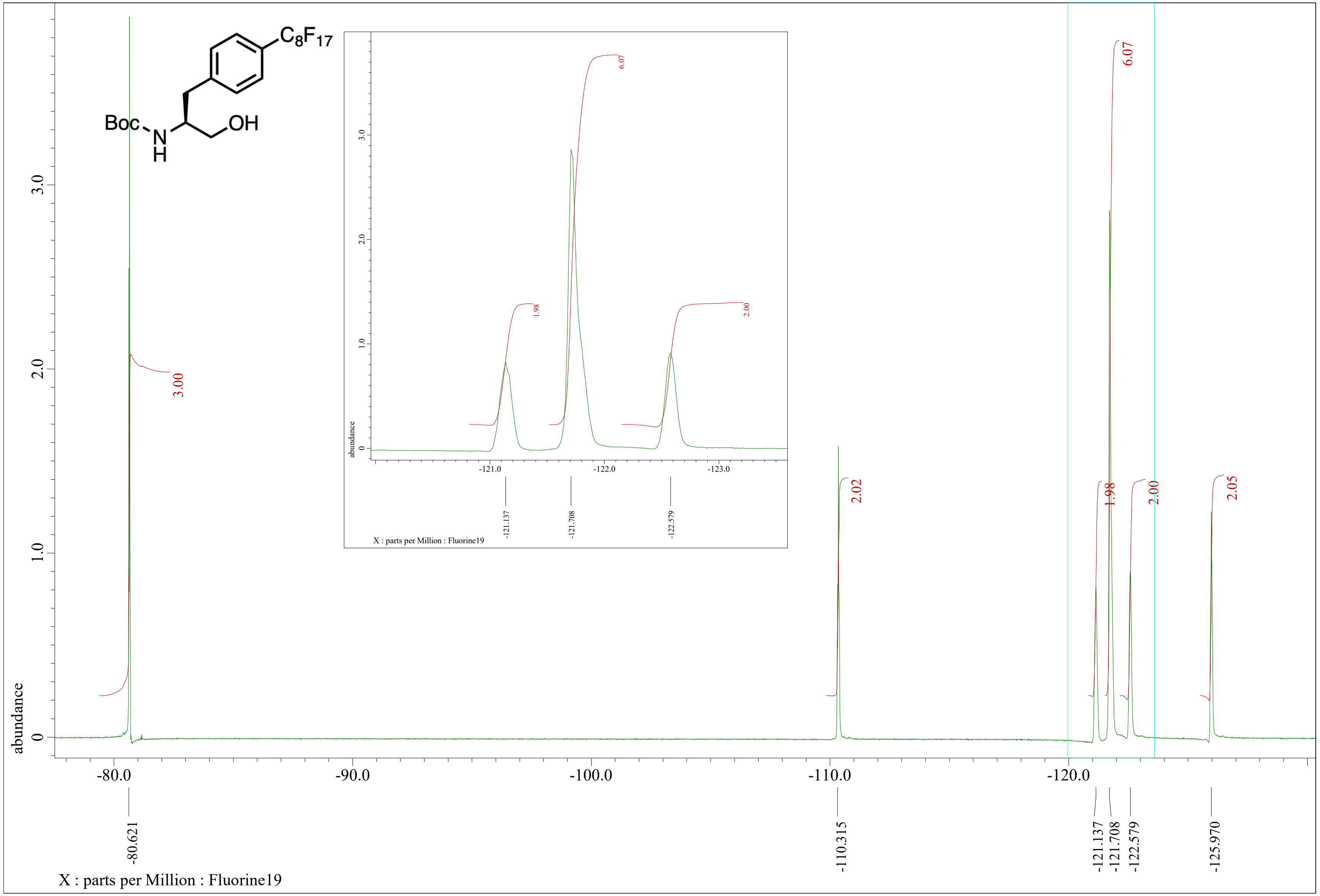

^{13}C NMR, 101 MHz, $CDCl_3$

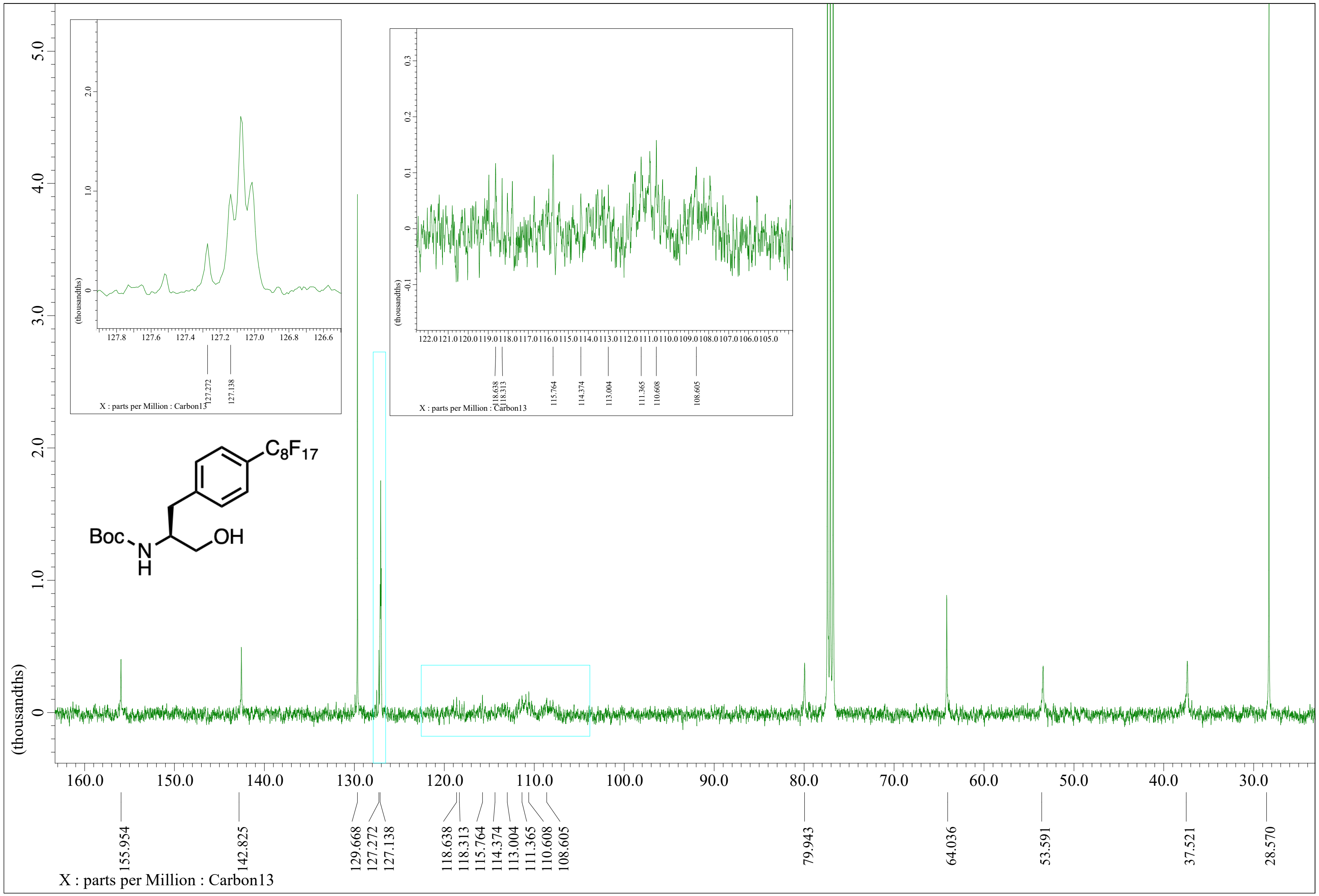

^{1}H NMR, 400 MHz, $(CD_3)_2SO$

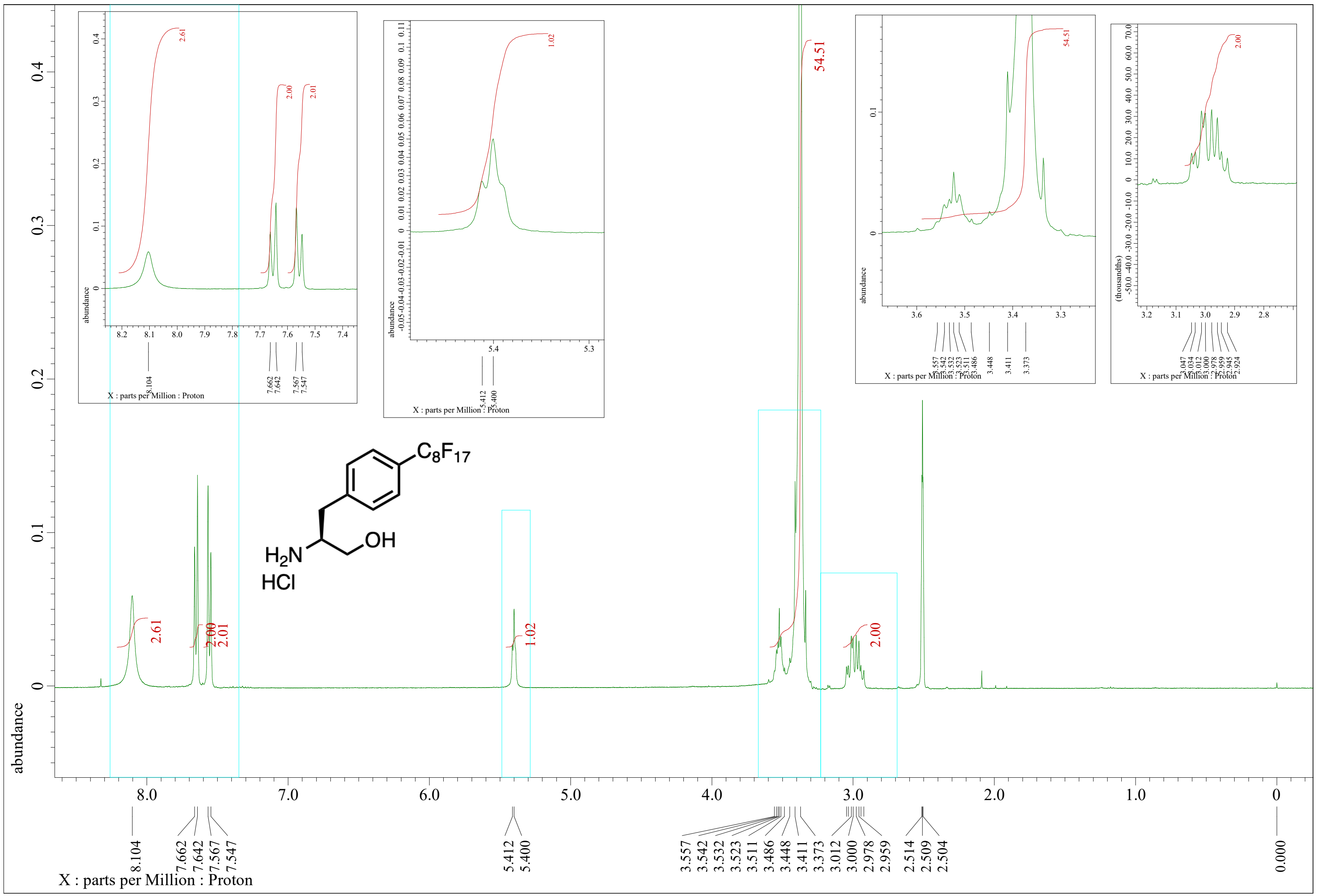

^{19}F NMR, 376 MHz, $(CD_3)_2SO$

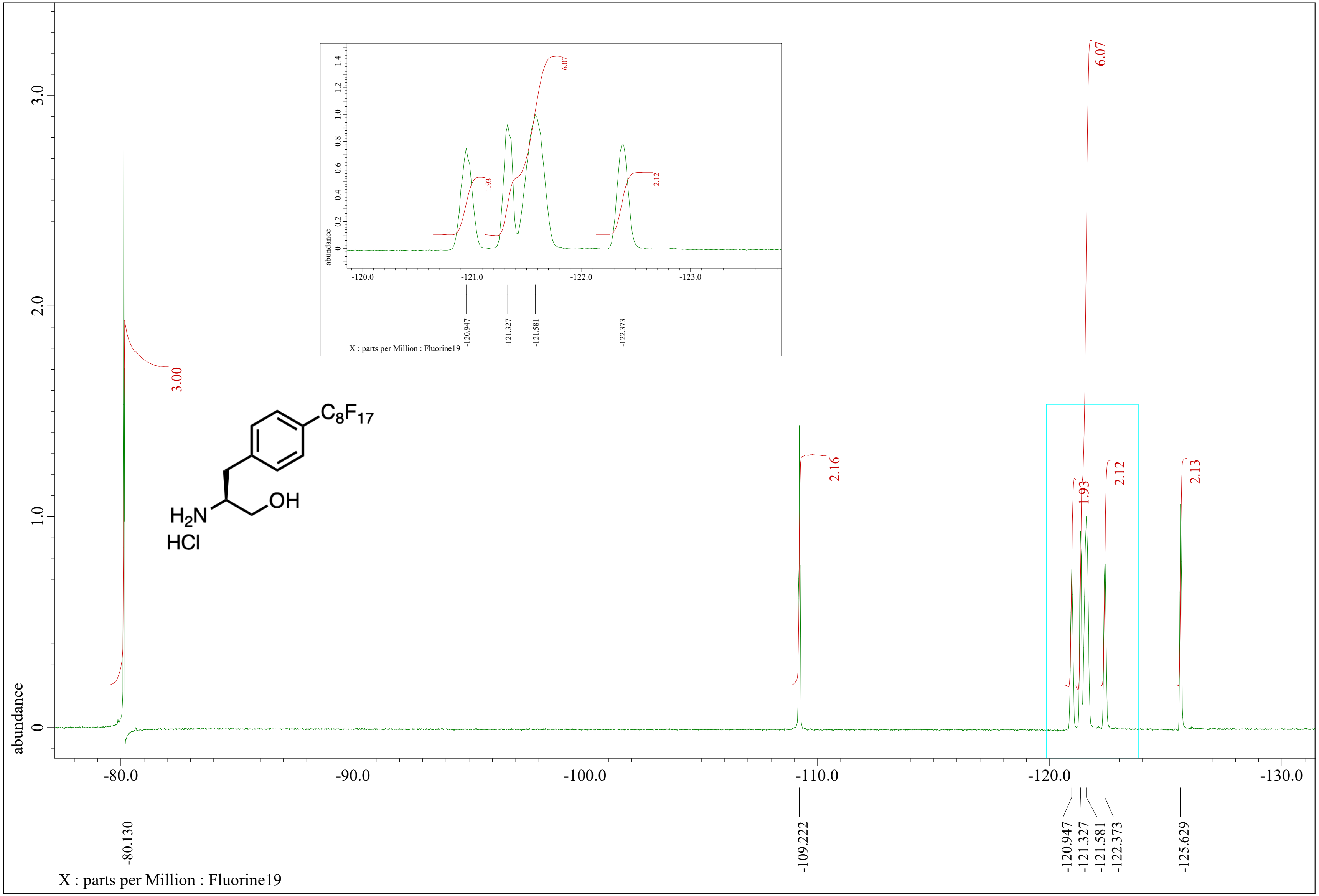

^{13}C NMR, 101 MHz, $(CD_3)_2SO$

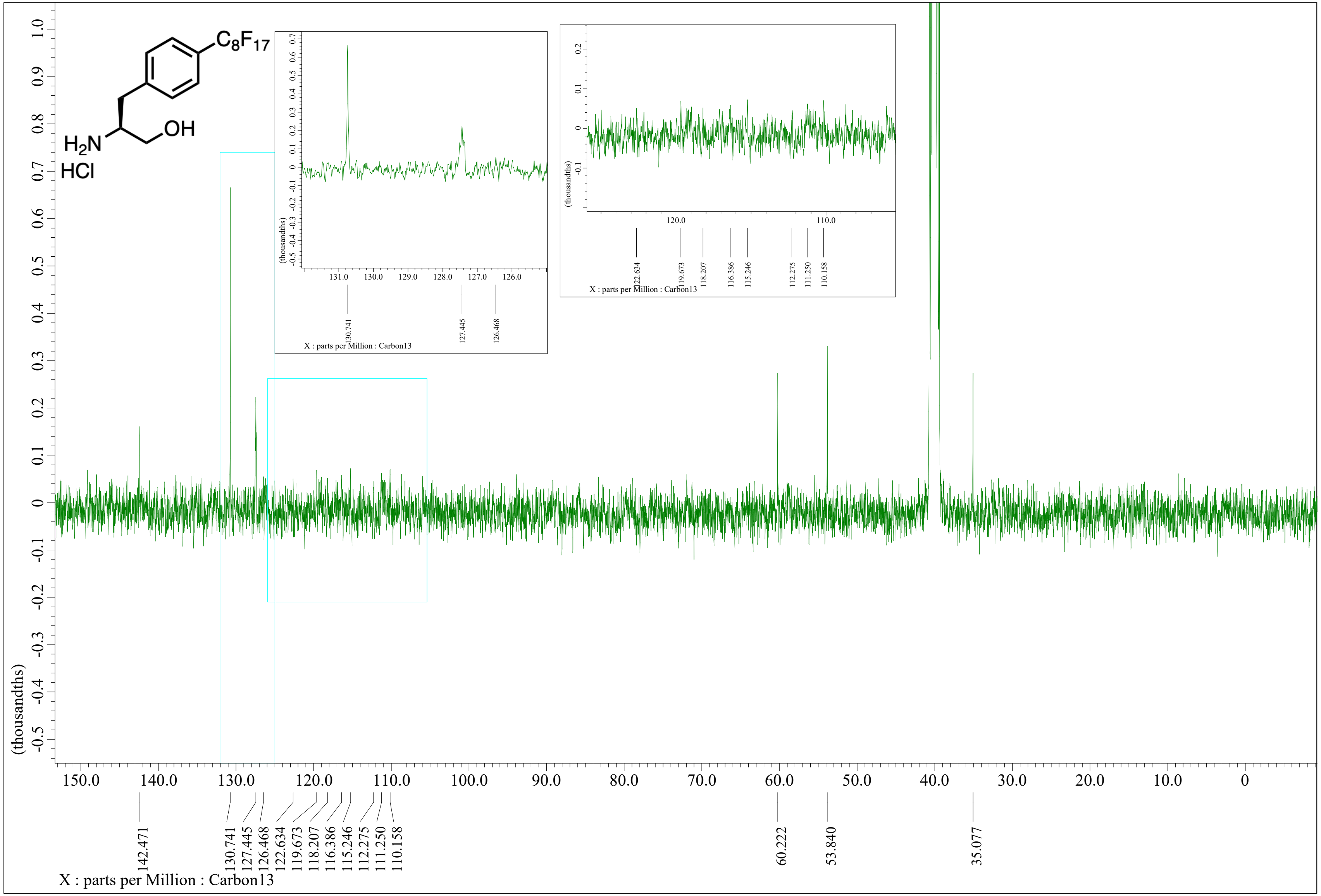

^{1}H NMR, 400 MHz, $CDCl_3$

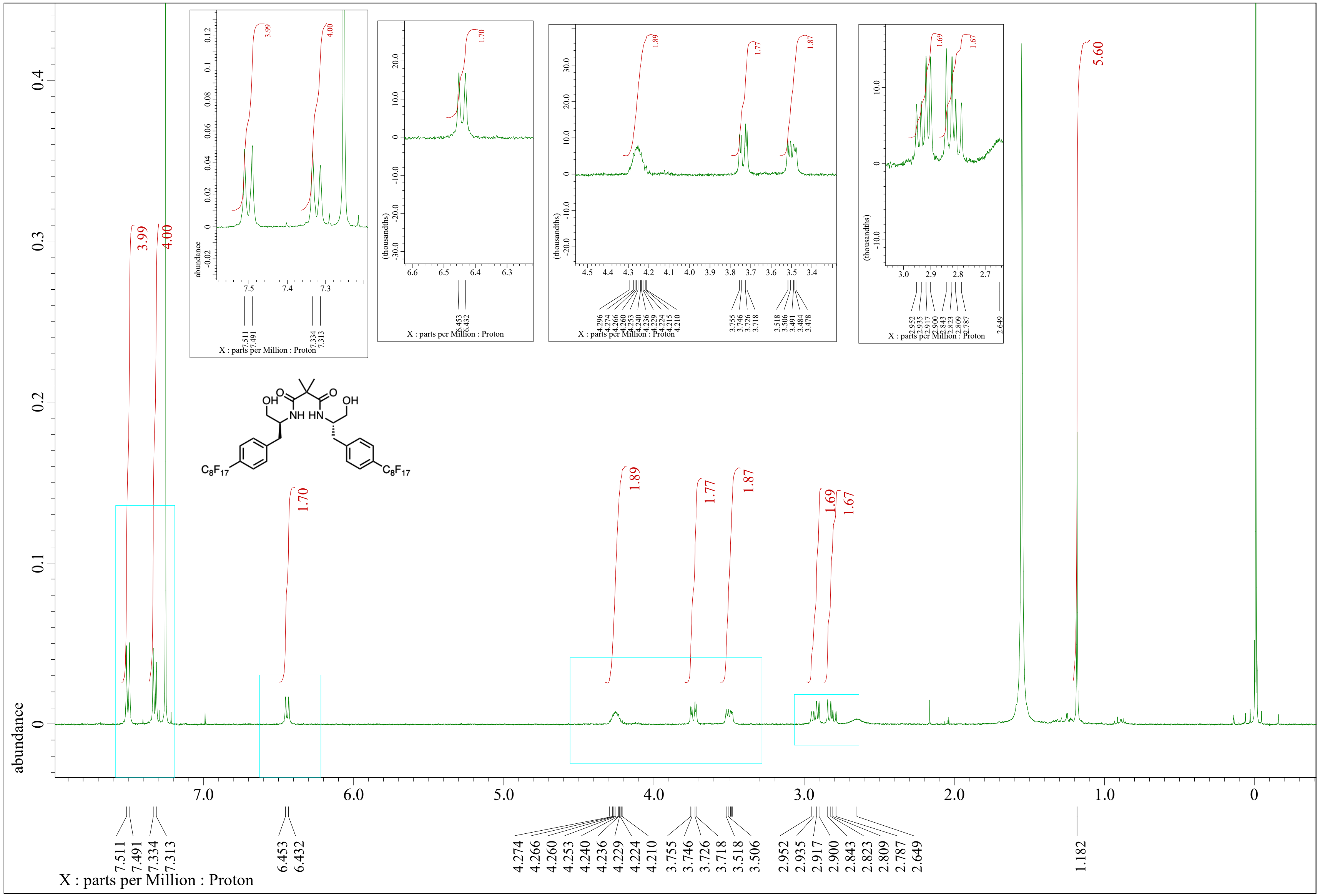

^{19}F NMR, 376 MHz, $CDCl_3$

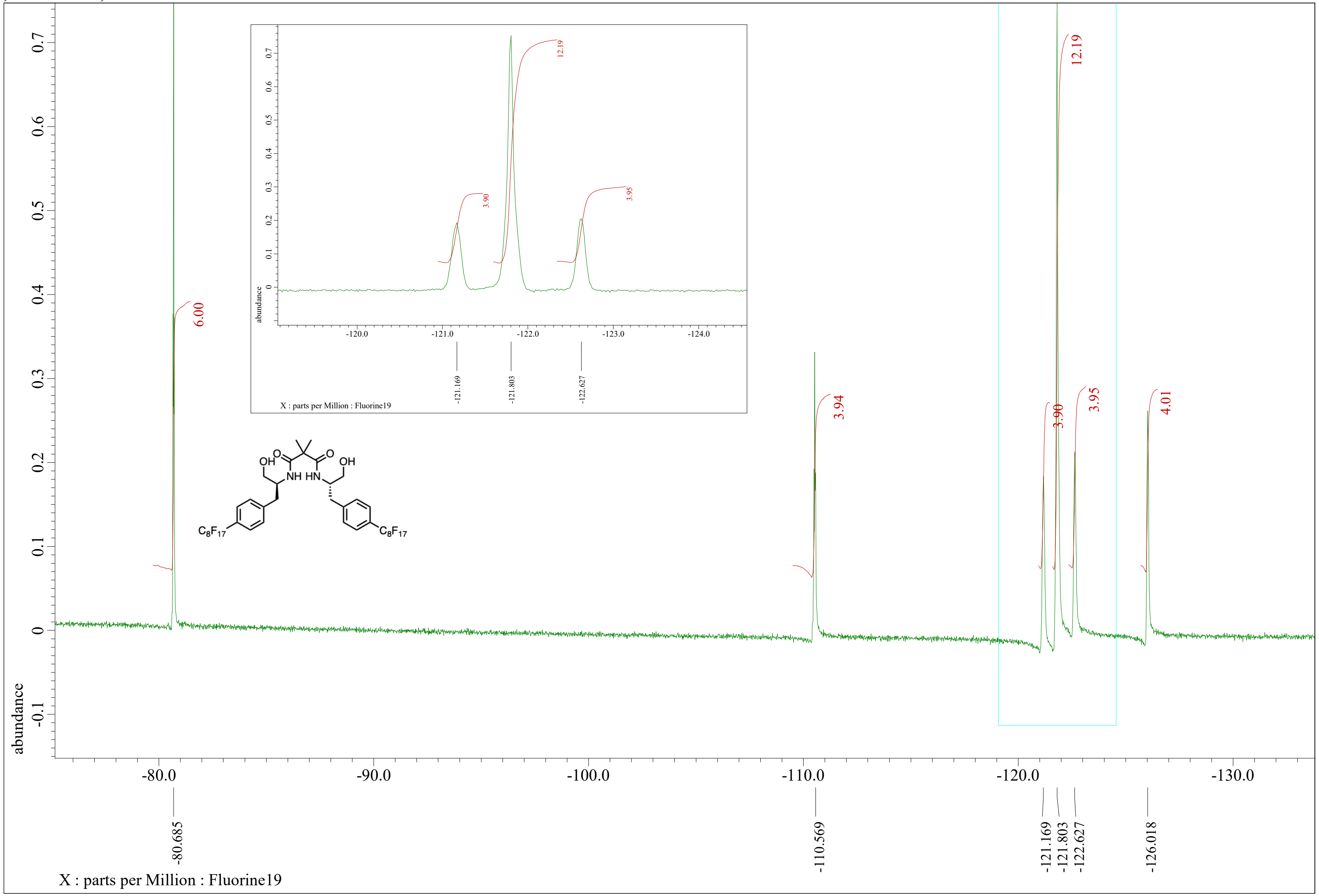

^{13}C NMR, 101 MHz, CD_3OD

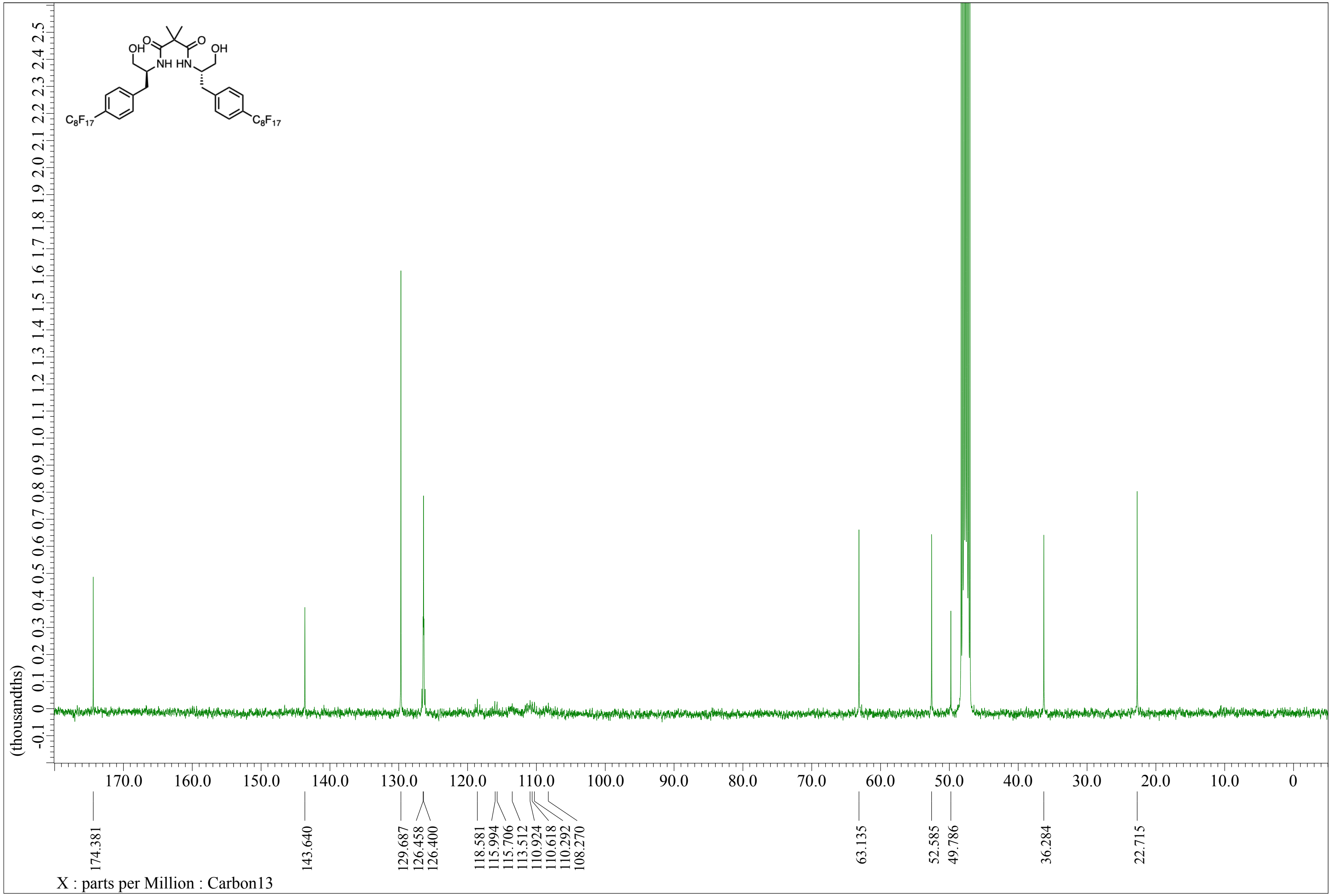

^{1}H NMR, 400 MHz, $CDCl_3$

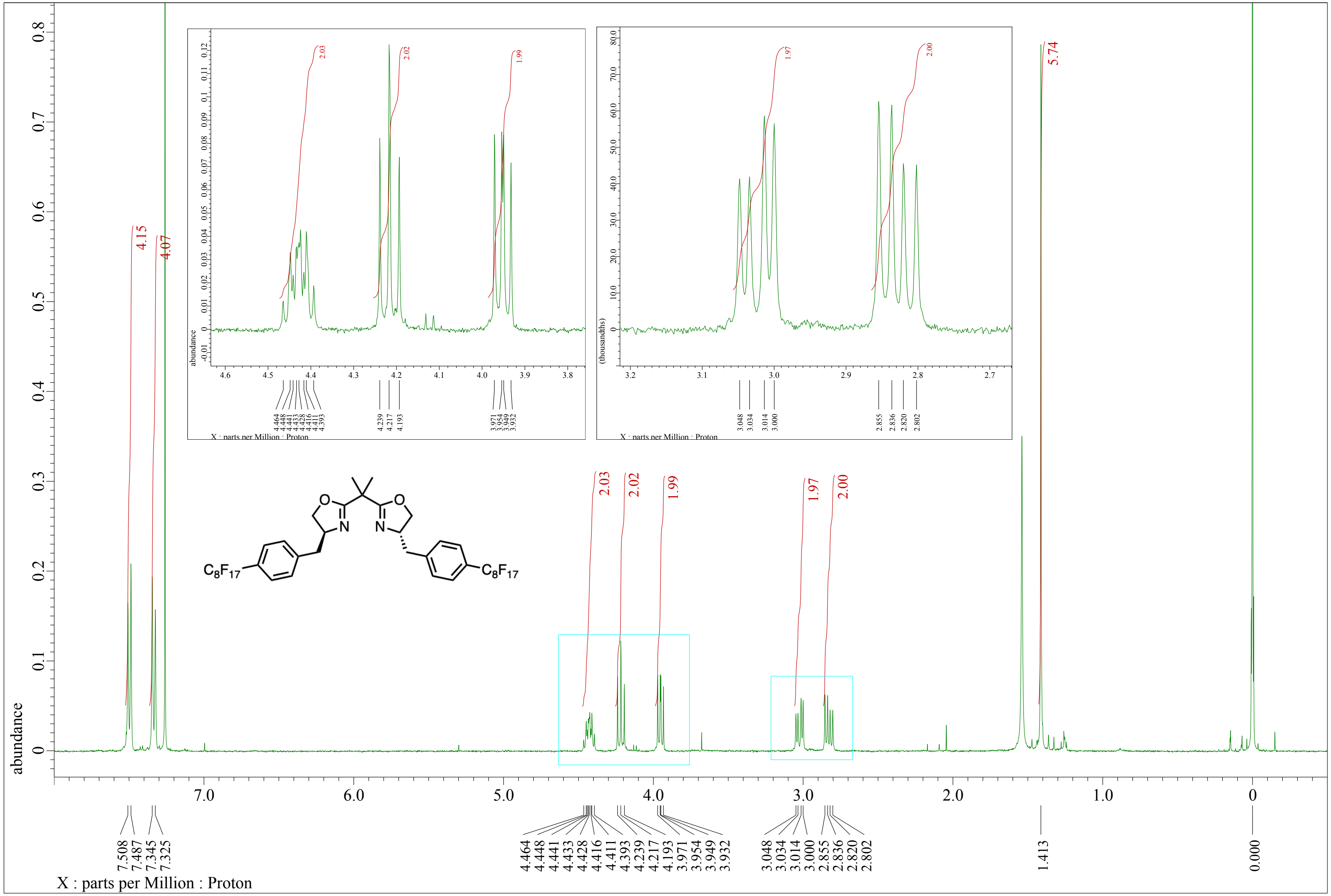

^{19}F NMR, 376 MHz, $CDCl_3$

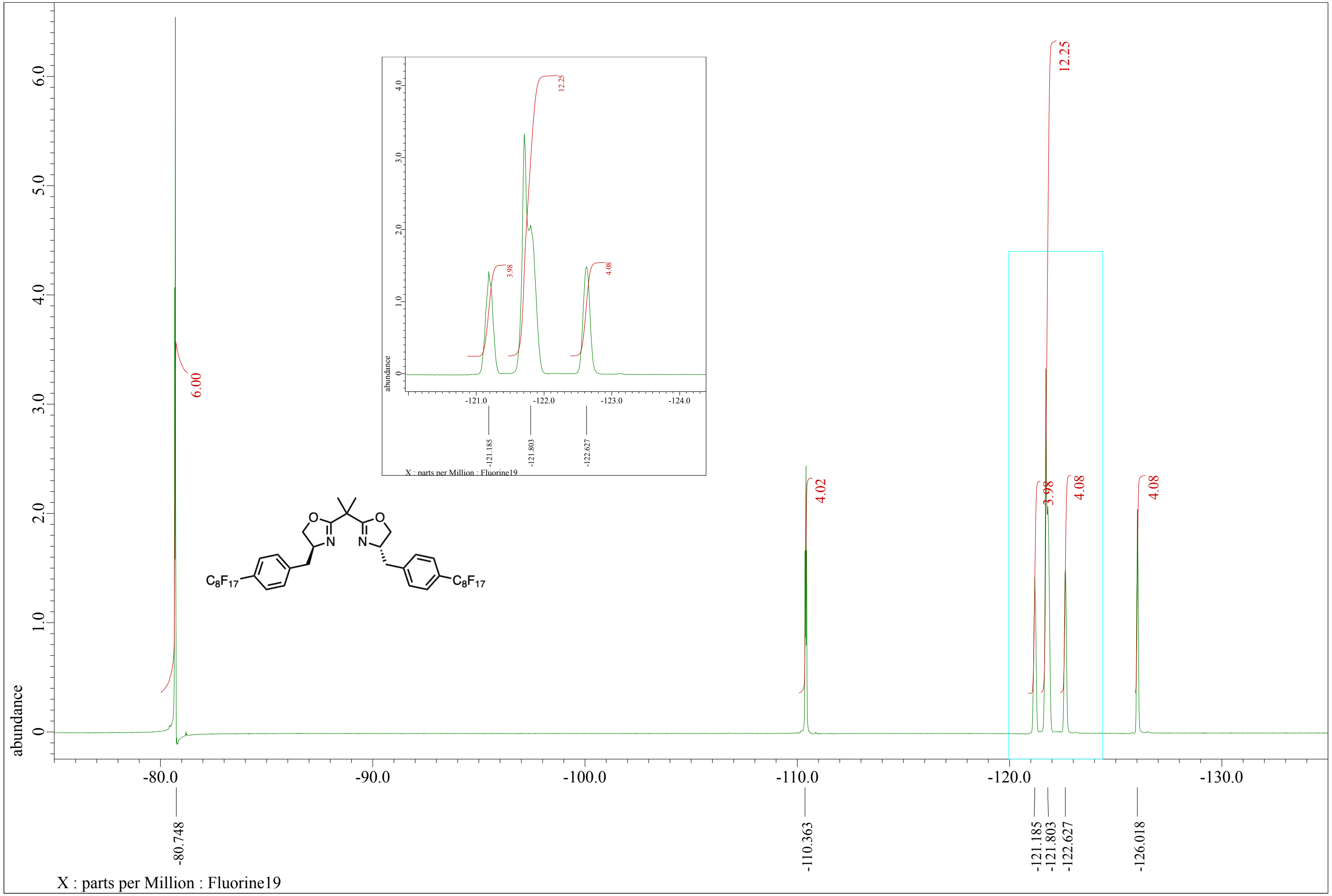

^{13}C NMR, 101 MHz, $CDCl_3$

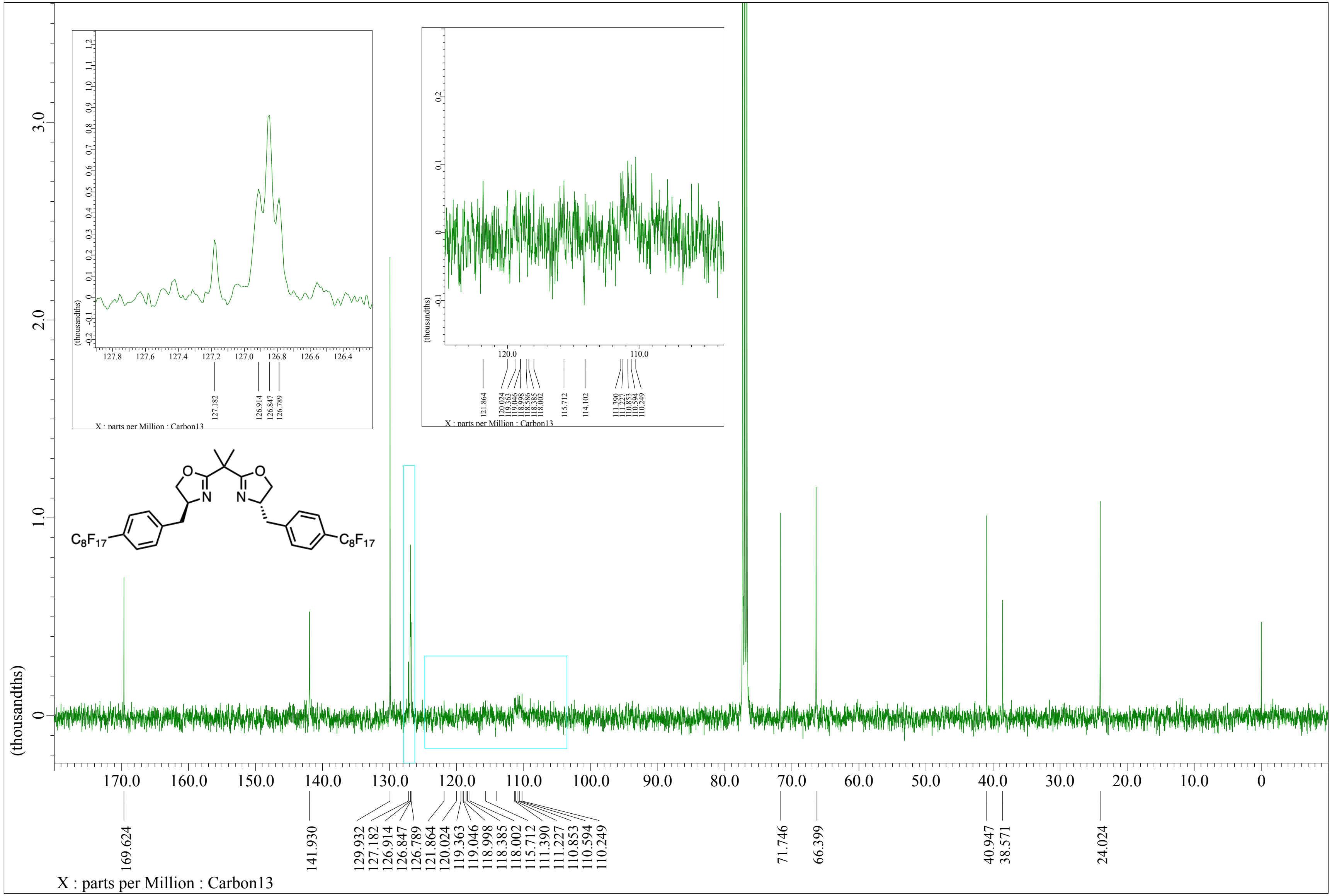

^{1}H NMR, 400 MHz, $CDCl_3$

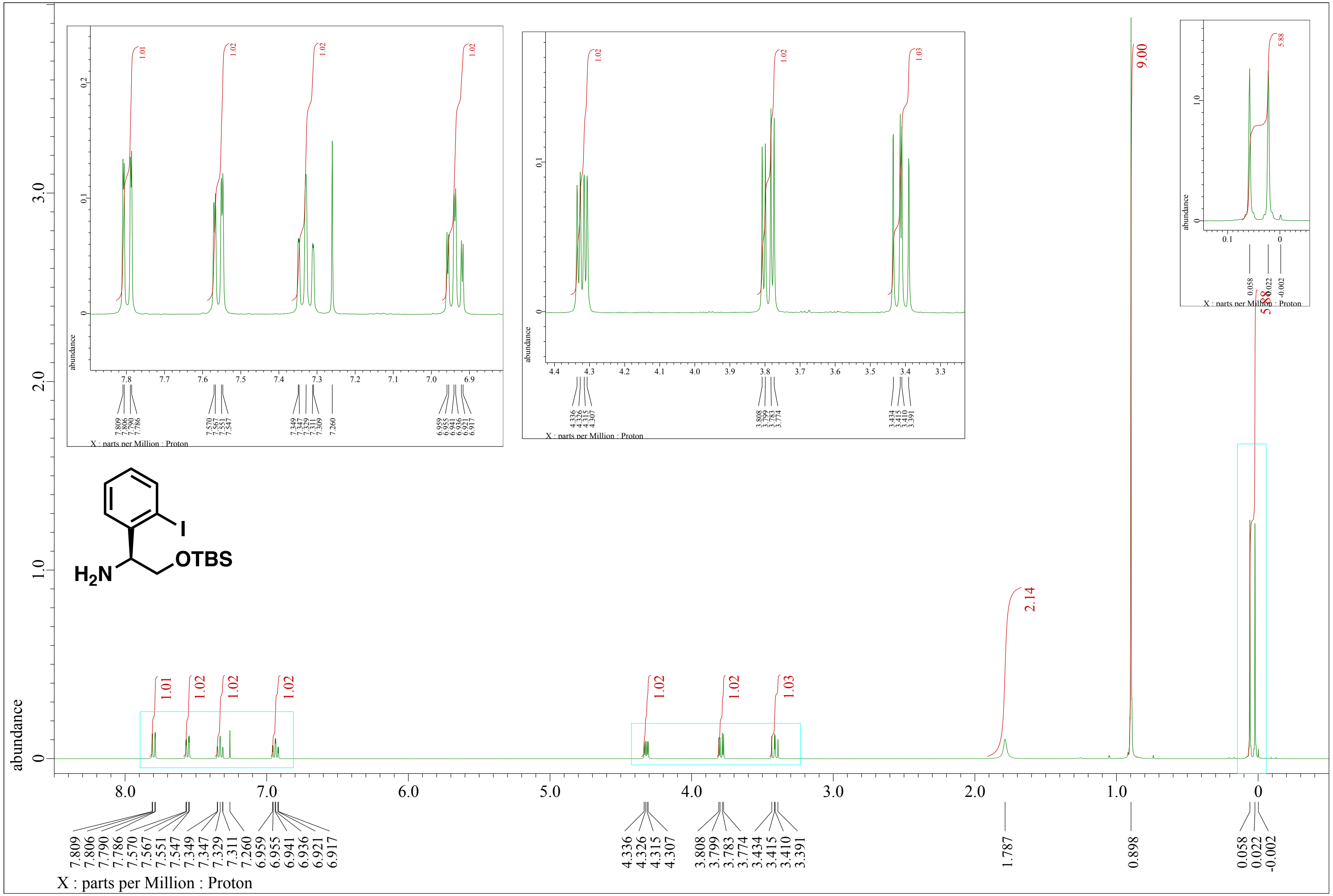

^{13}C NMR, 101 MHz, $CDCl_3$

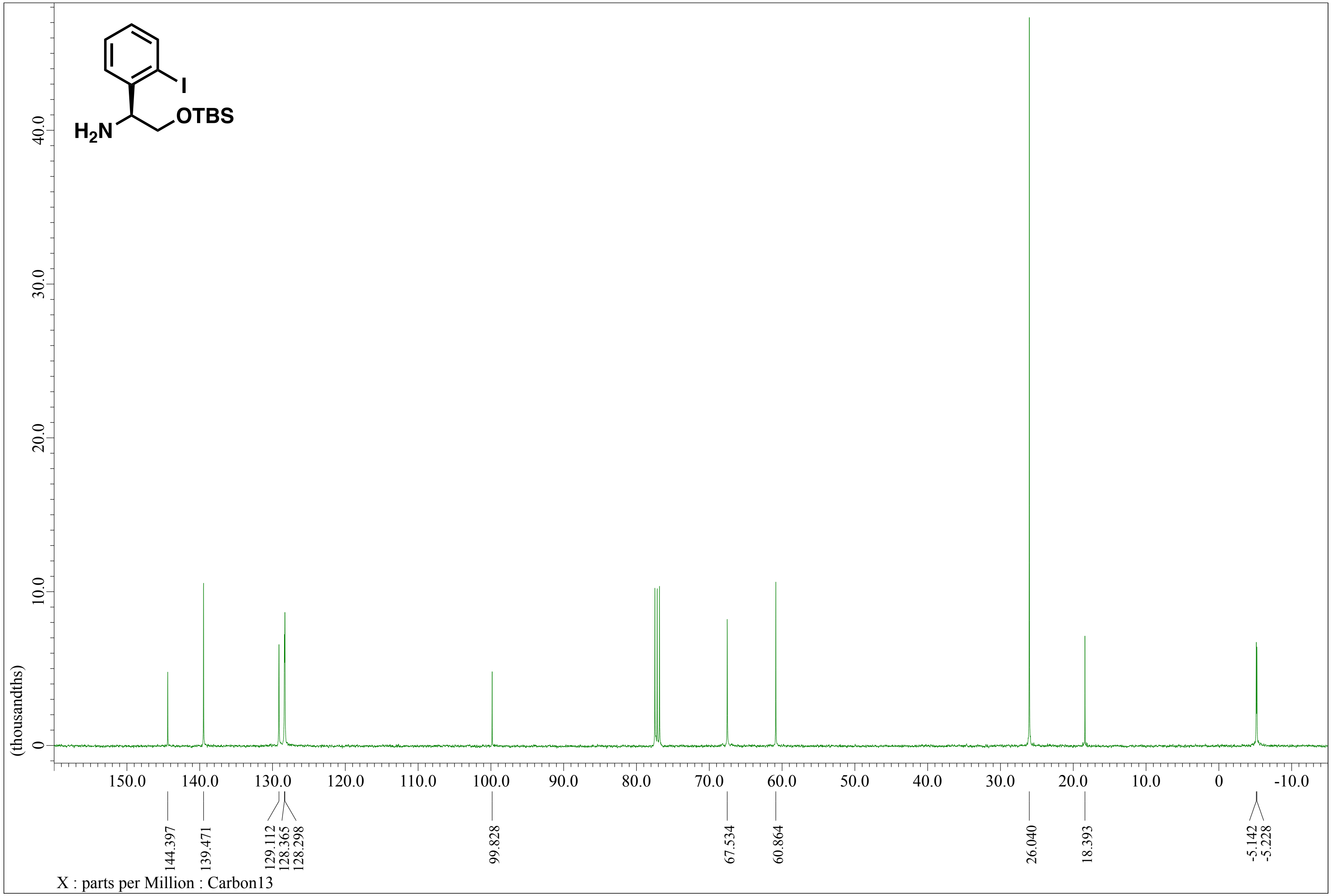

^{1}H NMR, 400 MHz, $CDCl_3$

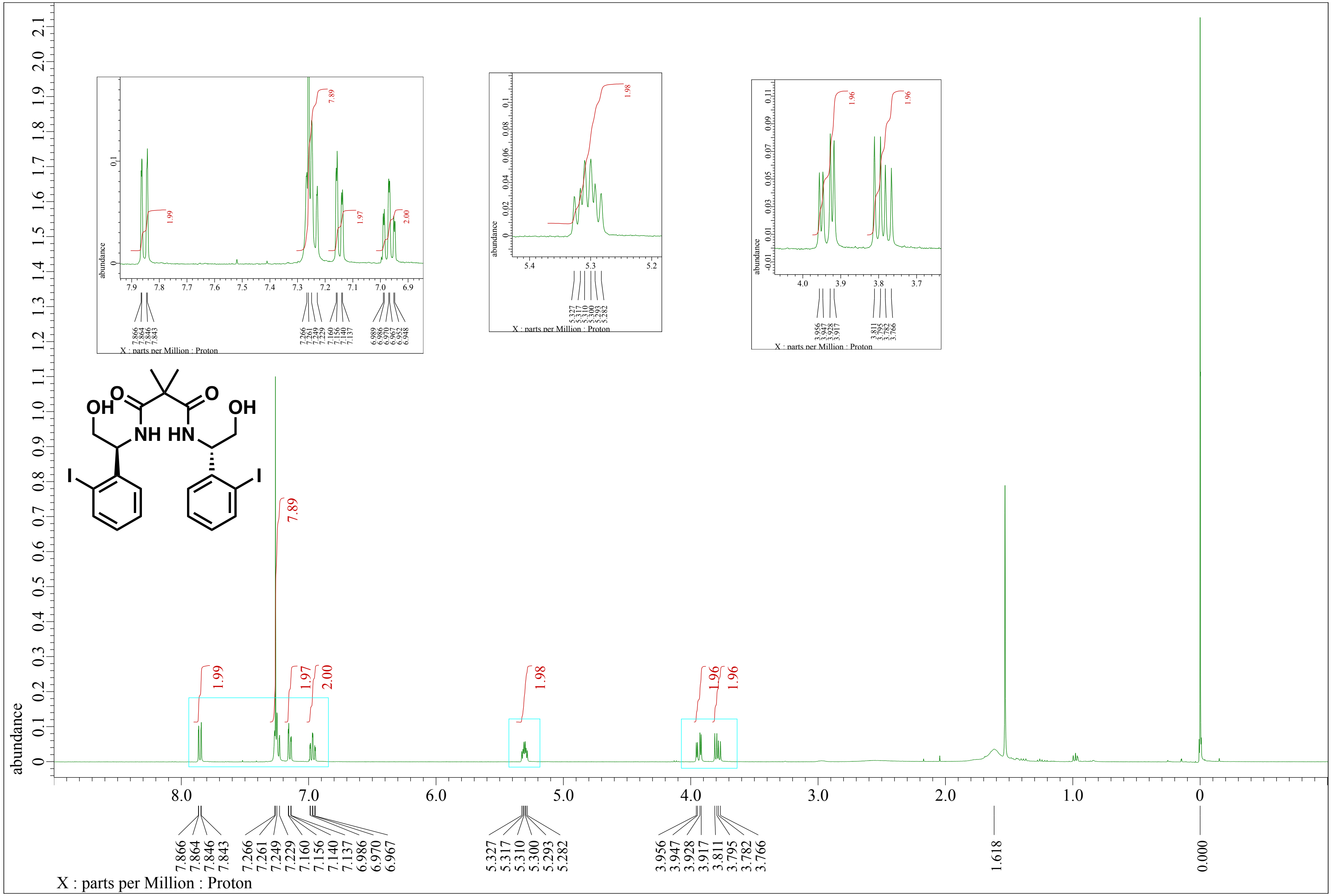

^{13}C NMR, 101 MHz, $CDCl_3$

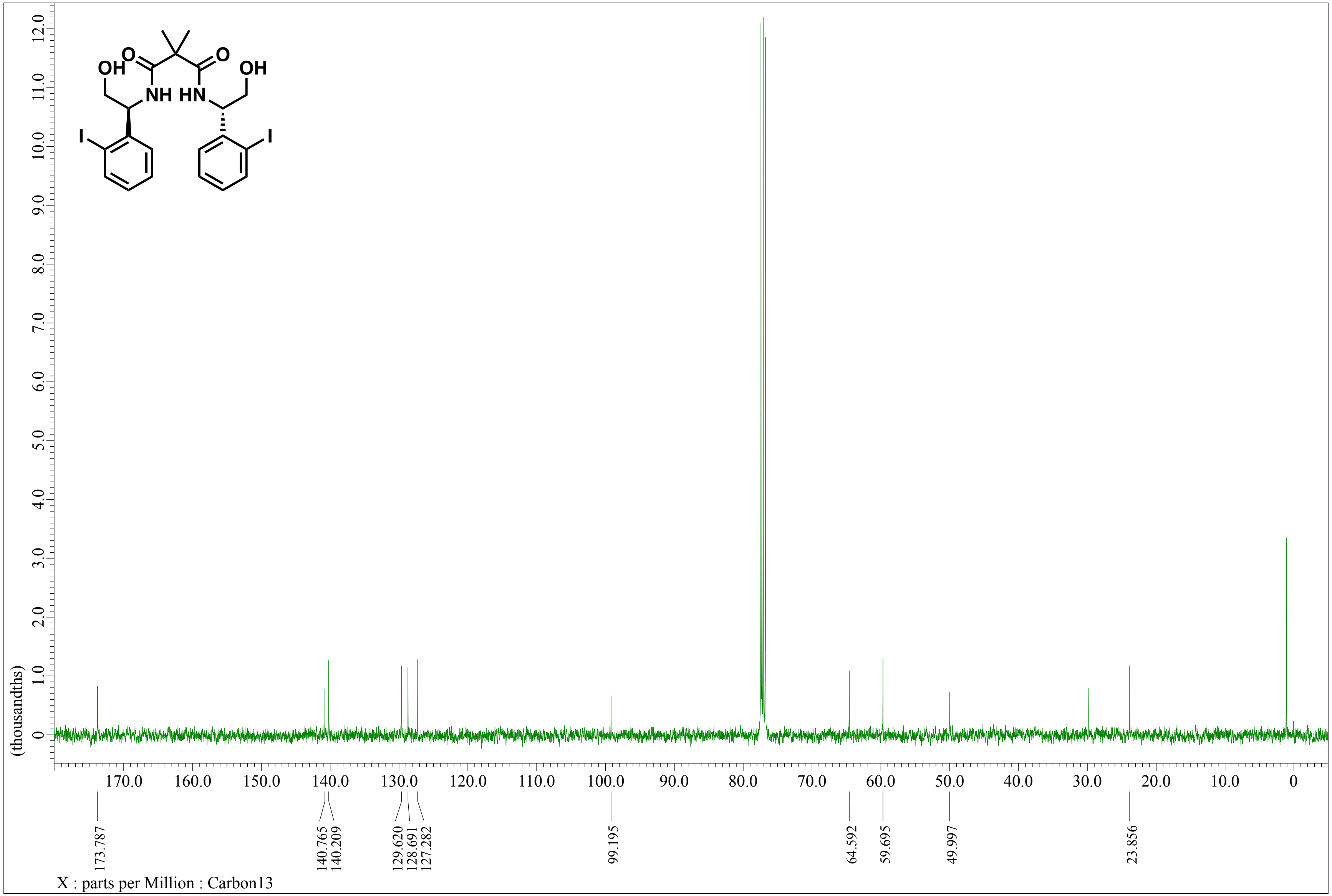

^{1}H NMR, 400 MHz, CDCl$_3$

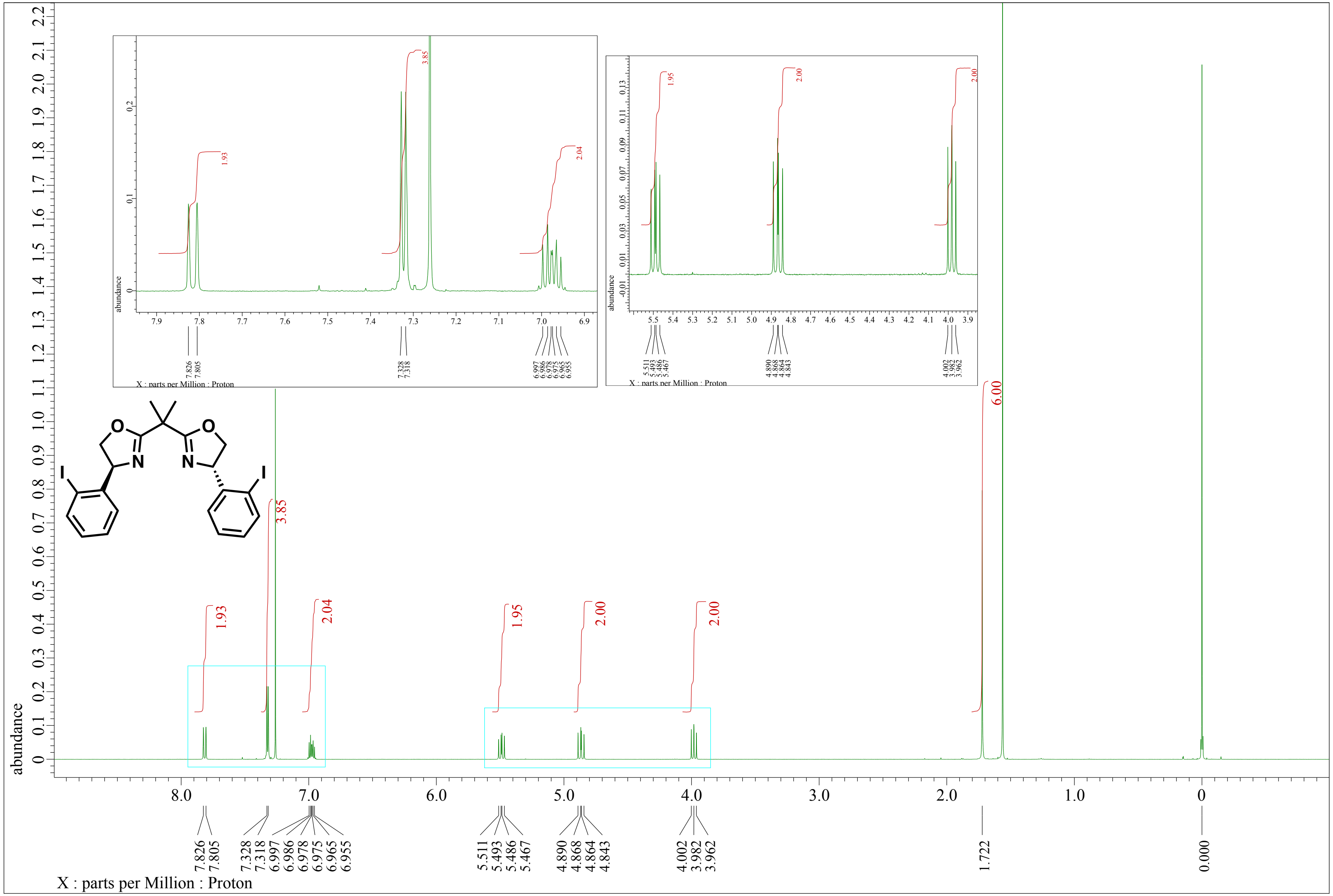

^{13}C NMR, 101 MHz, $CDCl_3$

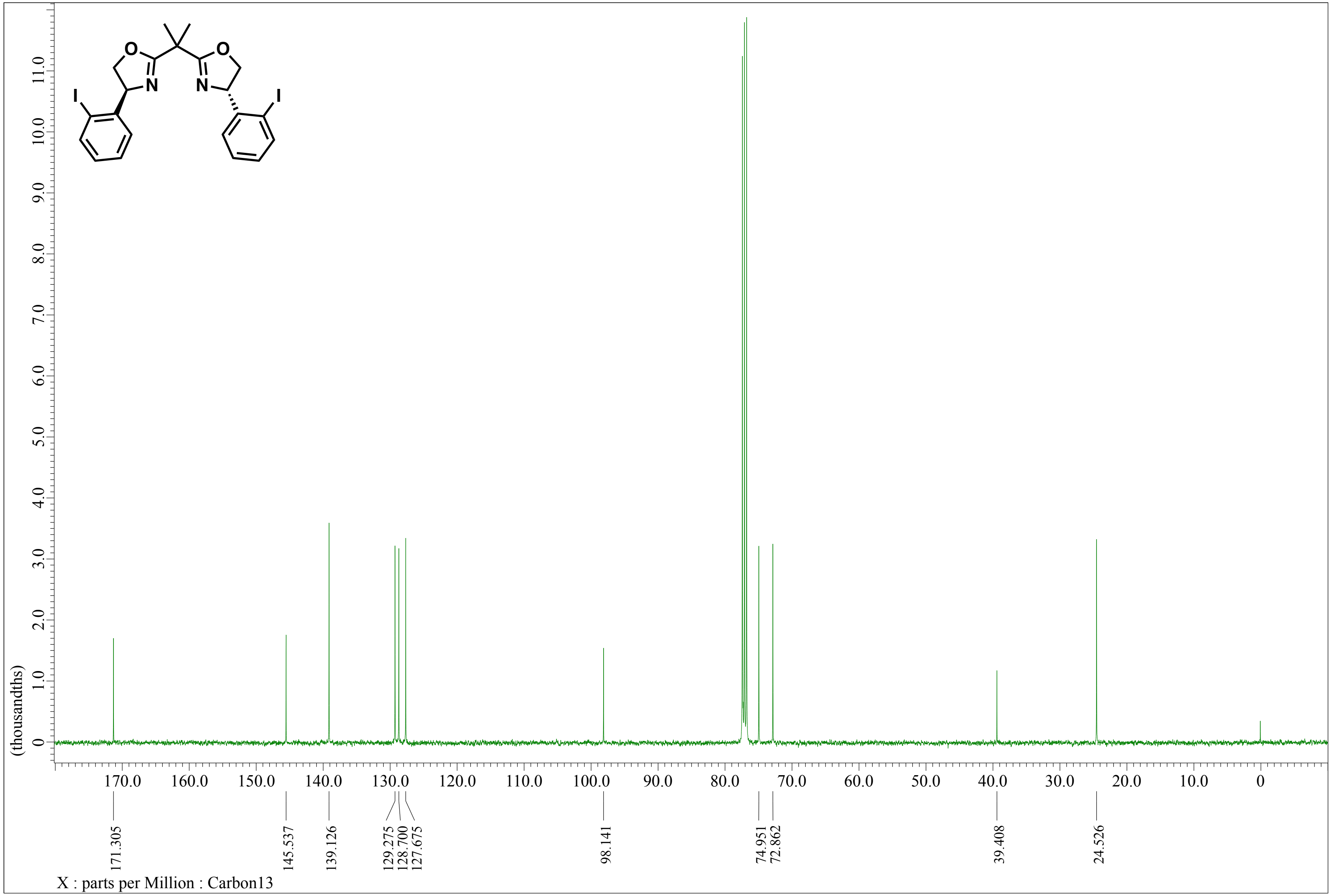

^{1}H NMR, 400 MHz, $CDCl_3$

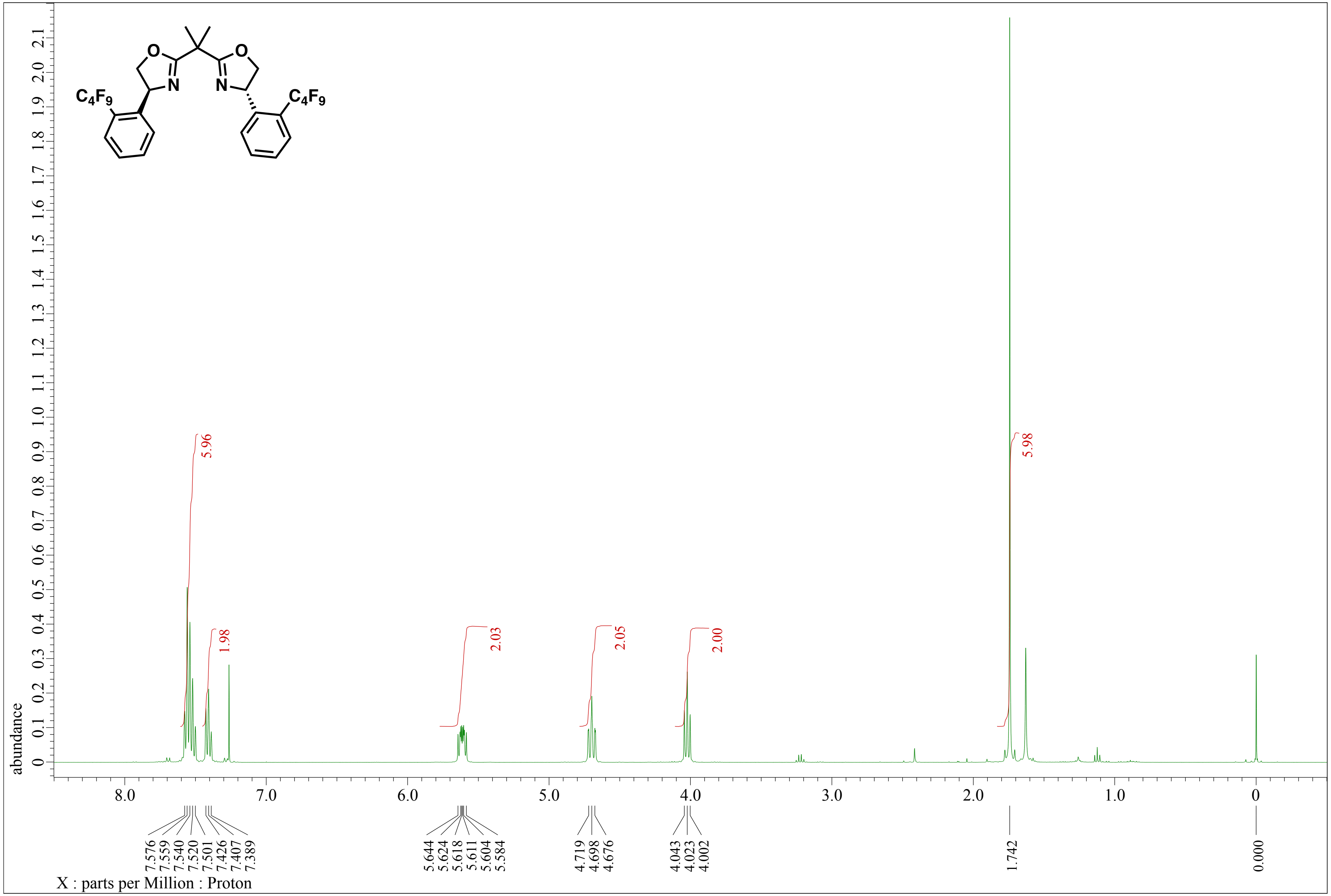

^{19}F NMR, 376 MHz, $CDCl_3$

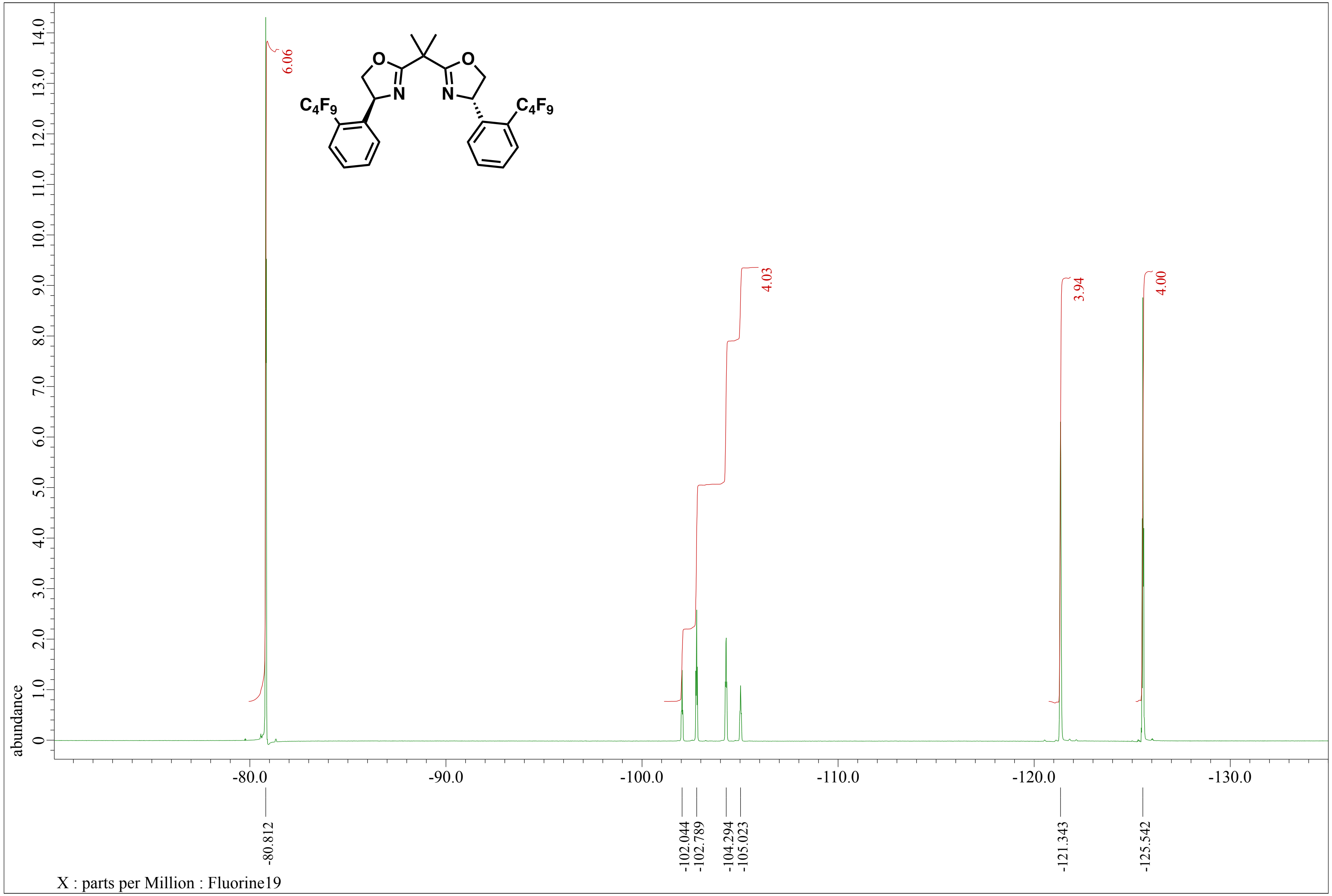

^{13}C NMR, 101 MHz, $CDCl_3$

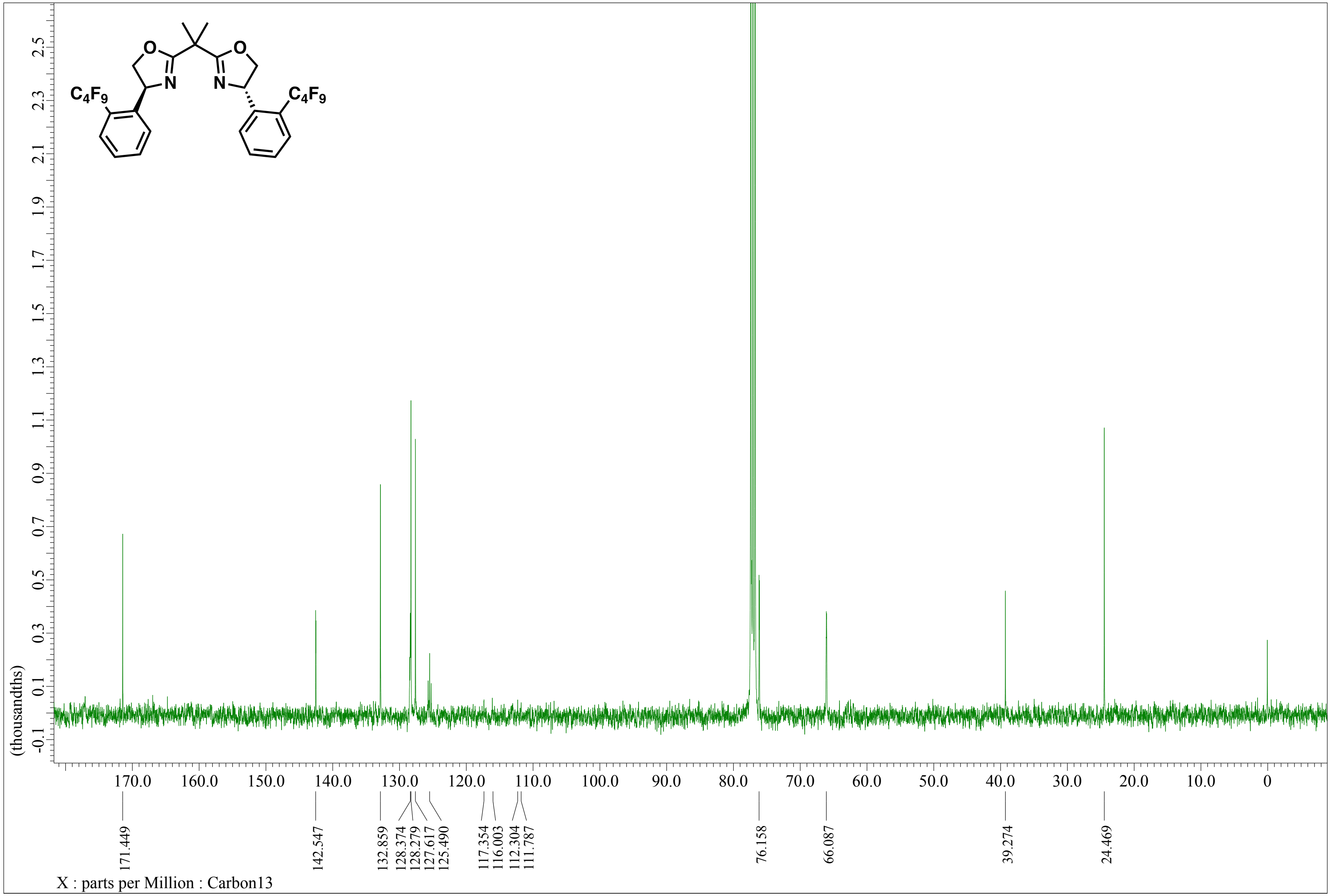

^{1}H NMR, 400 MHz, $CDCl_3$

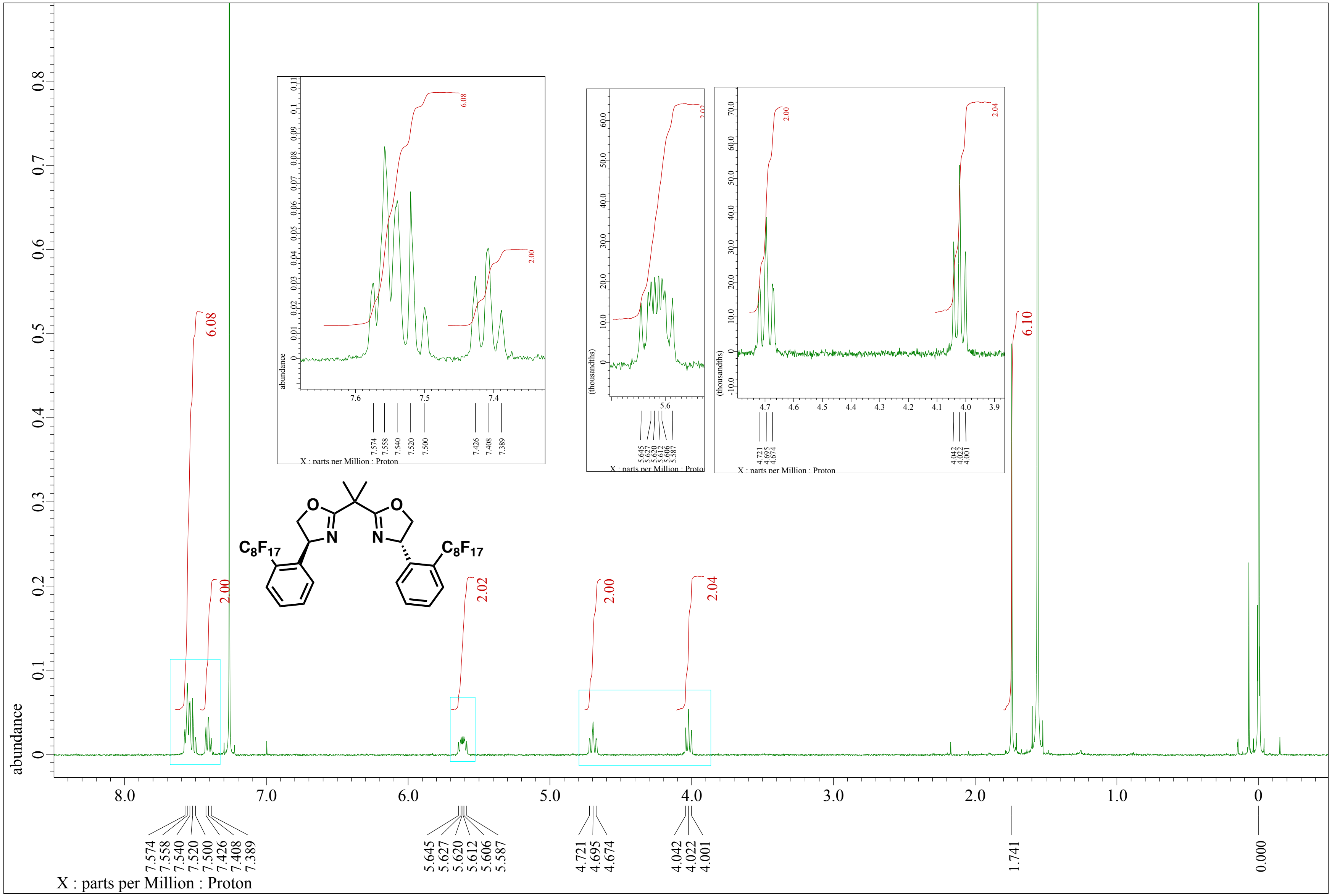

^{19}F NMR, 376 MHz, $CDCl_3$

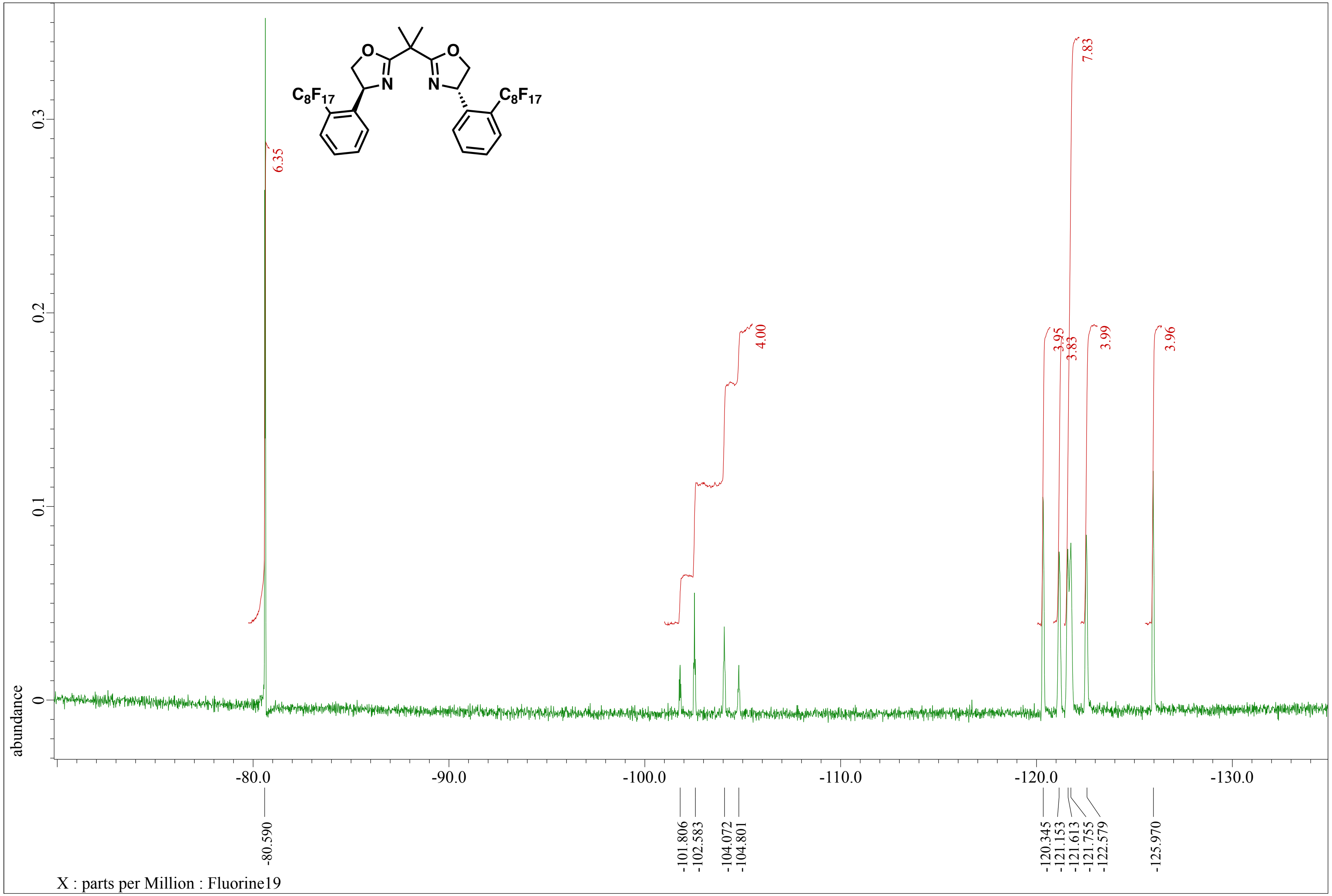

^{13}C NMR, 101 MHz, $CDCl_3$

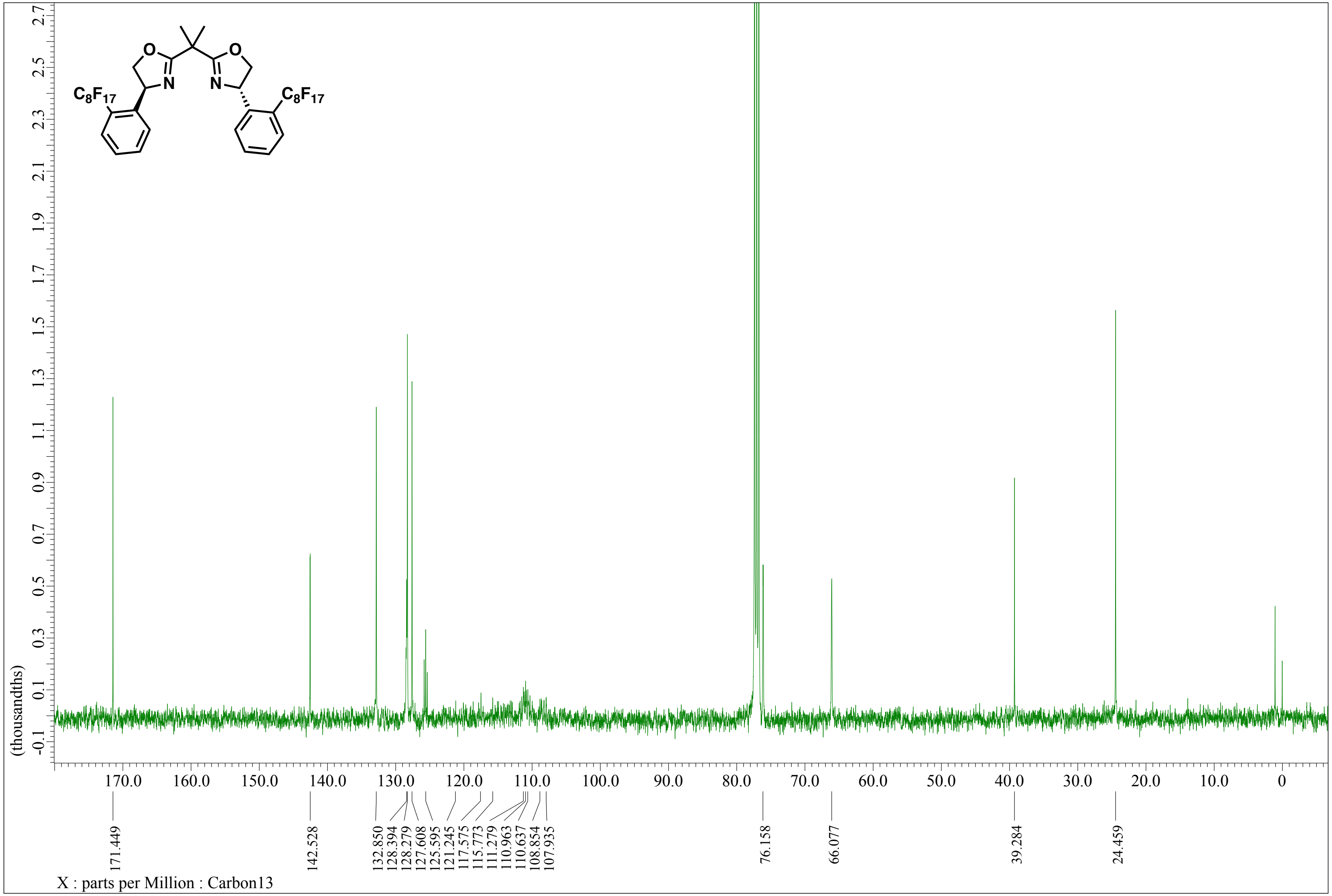

^{1}H NMR, 400 MHz, $CDCl_3$

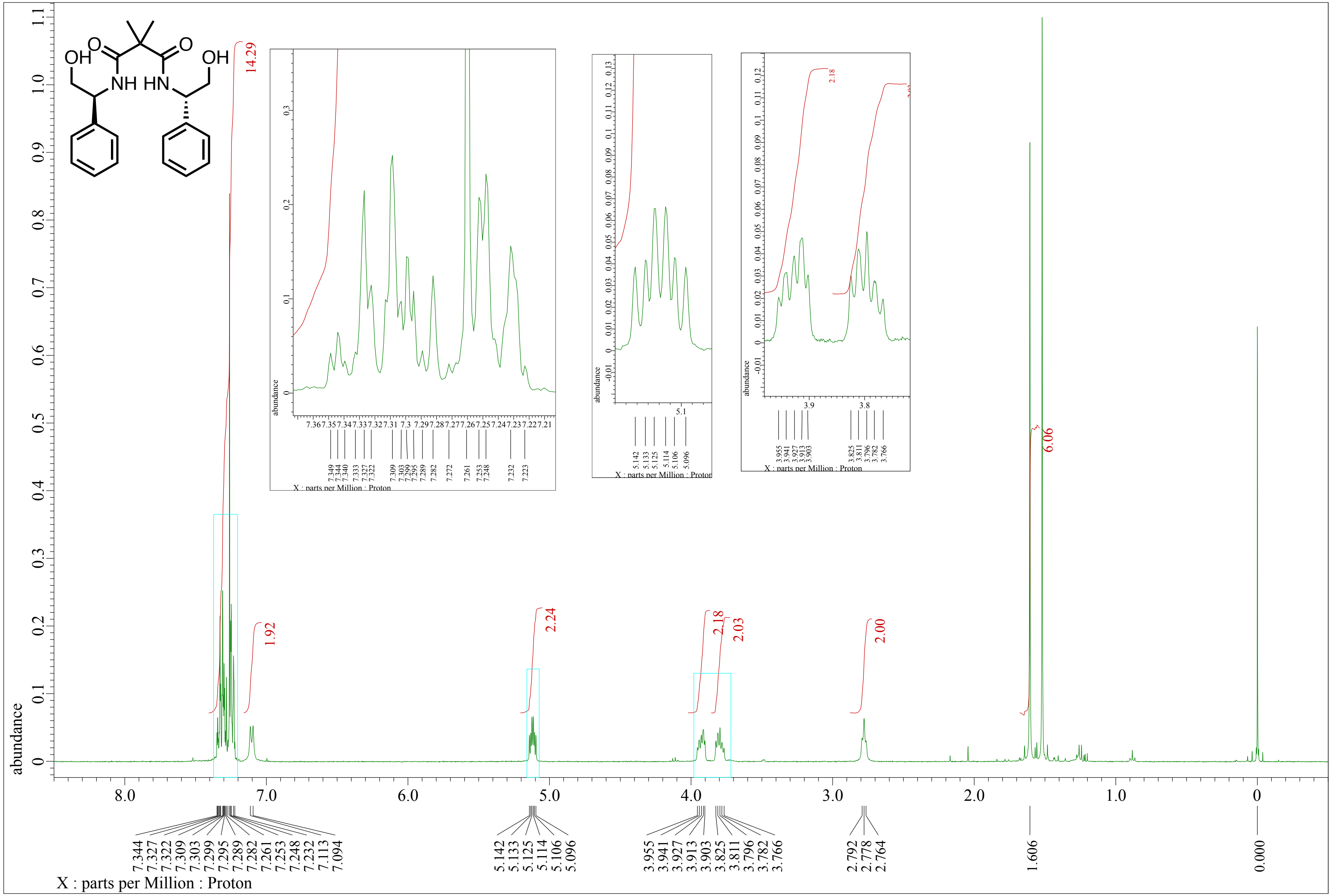

^{1}H NMR, 400 MHz, $CDCl_3$

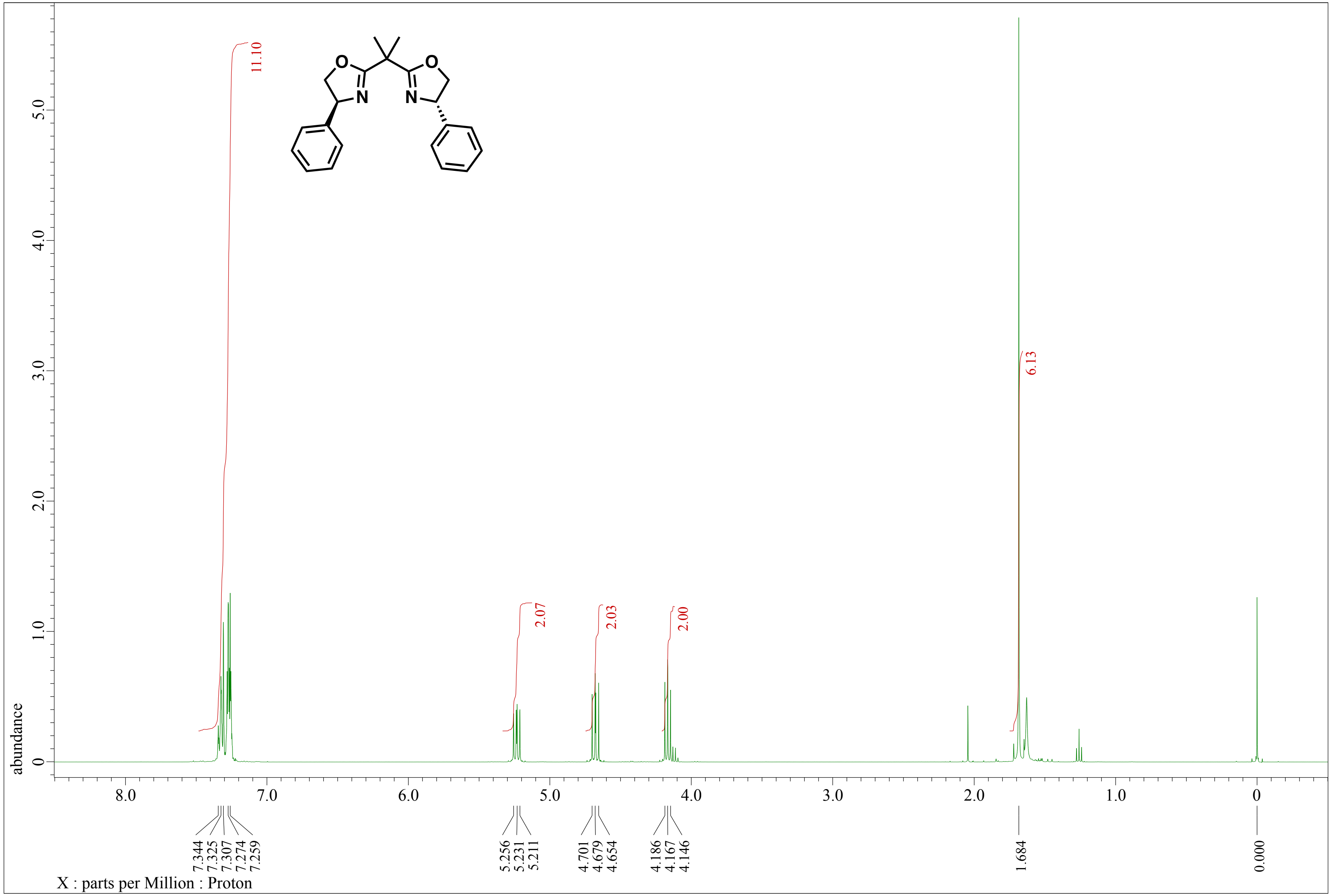

^{1}H NMR, 400 MHz, $CDCl_3$

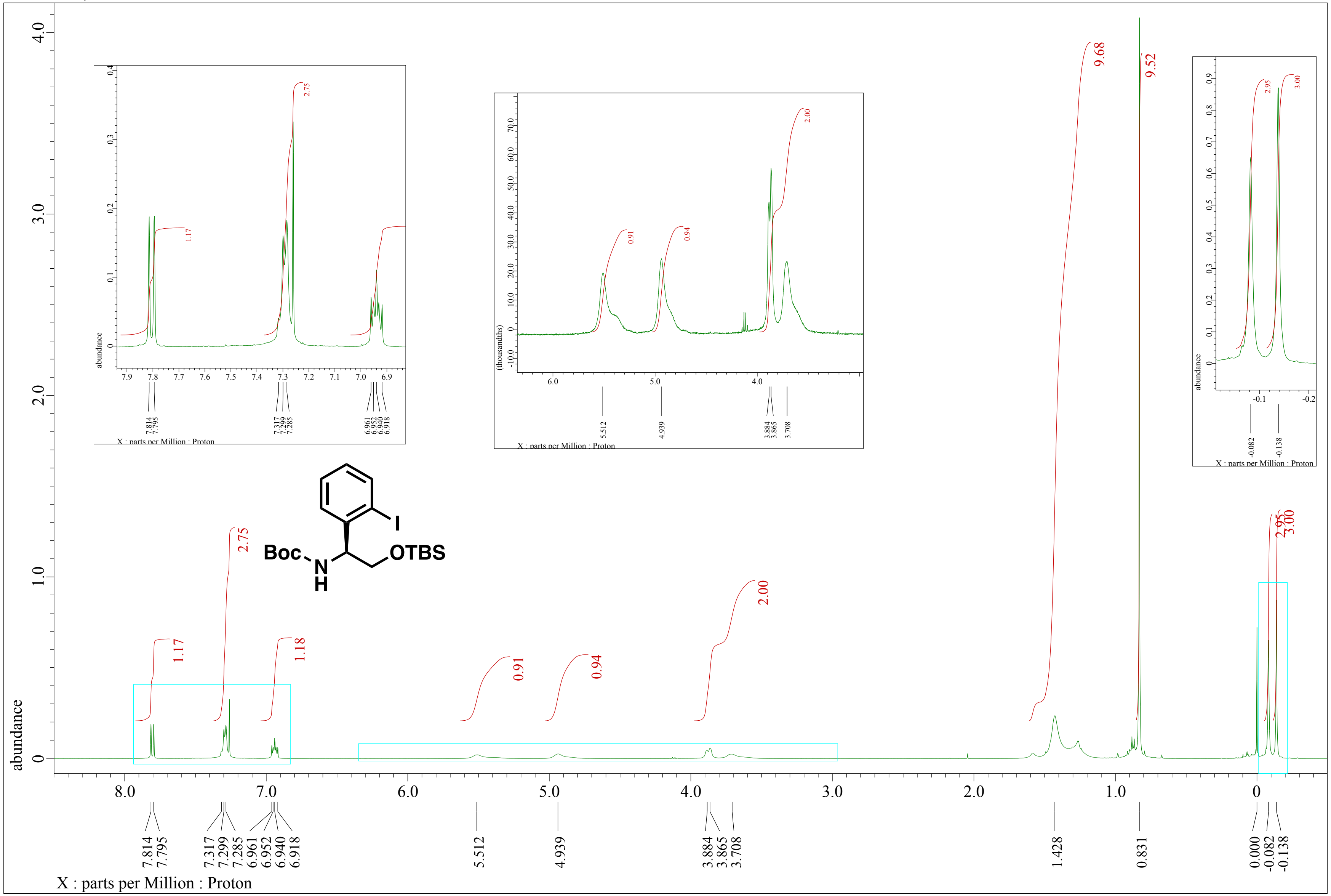

^{13}C NMR, 101 MHz, $CDCl_3$

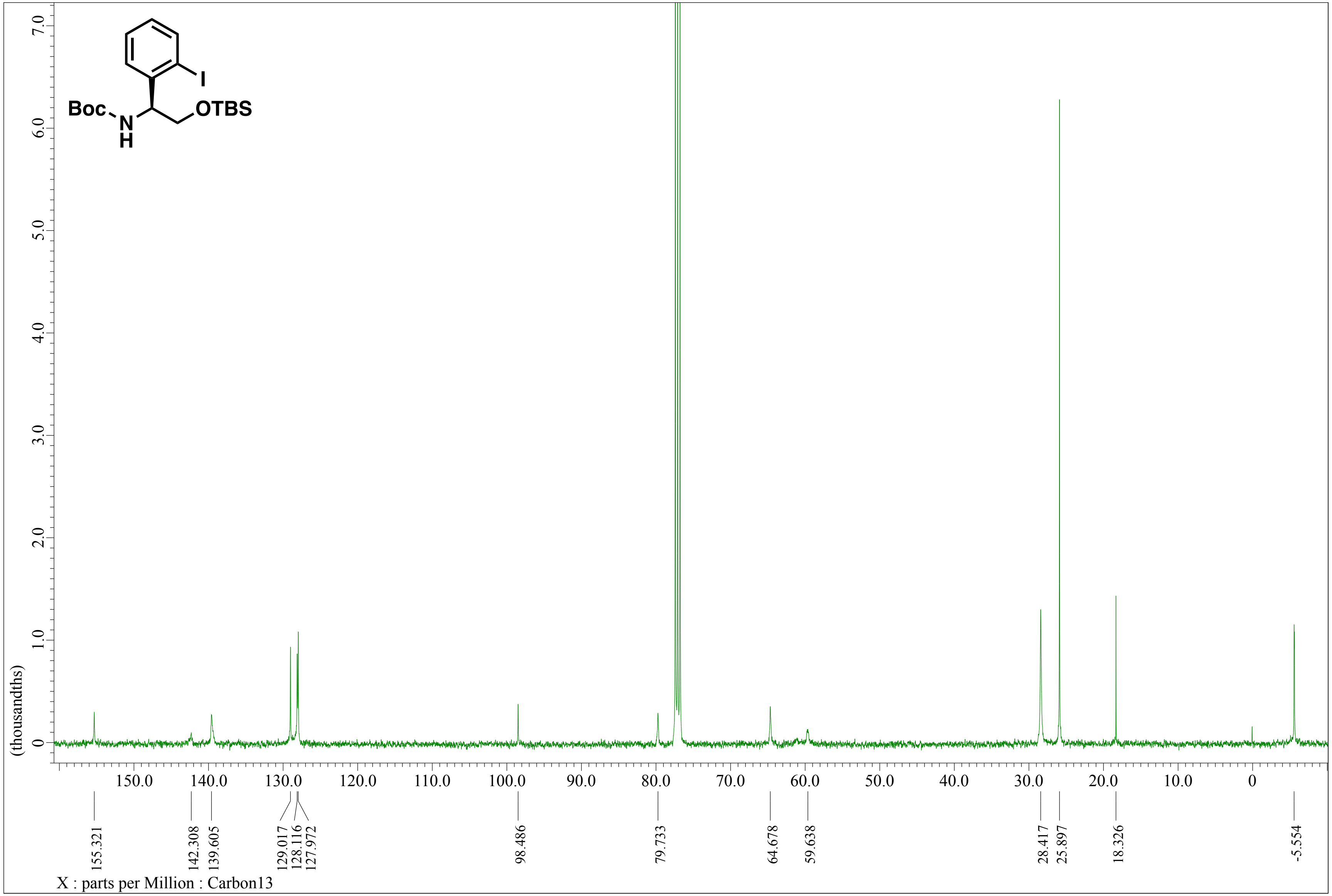

^{1}H NMR, 400 MHz, $CDCl_3$

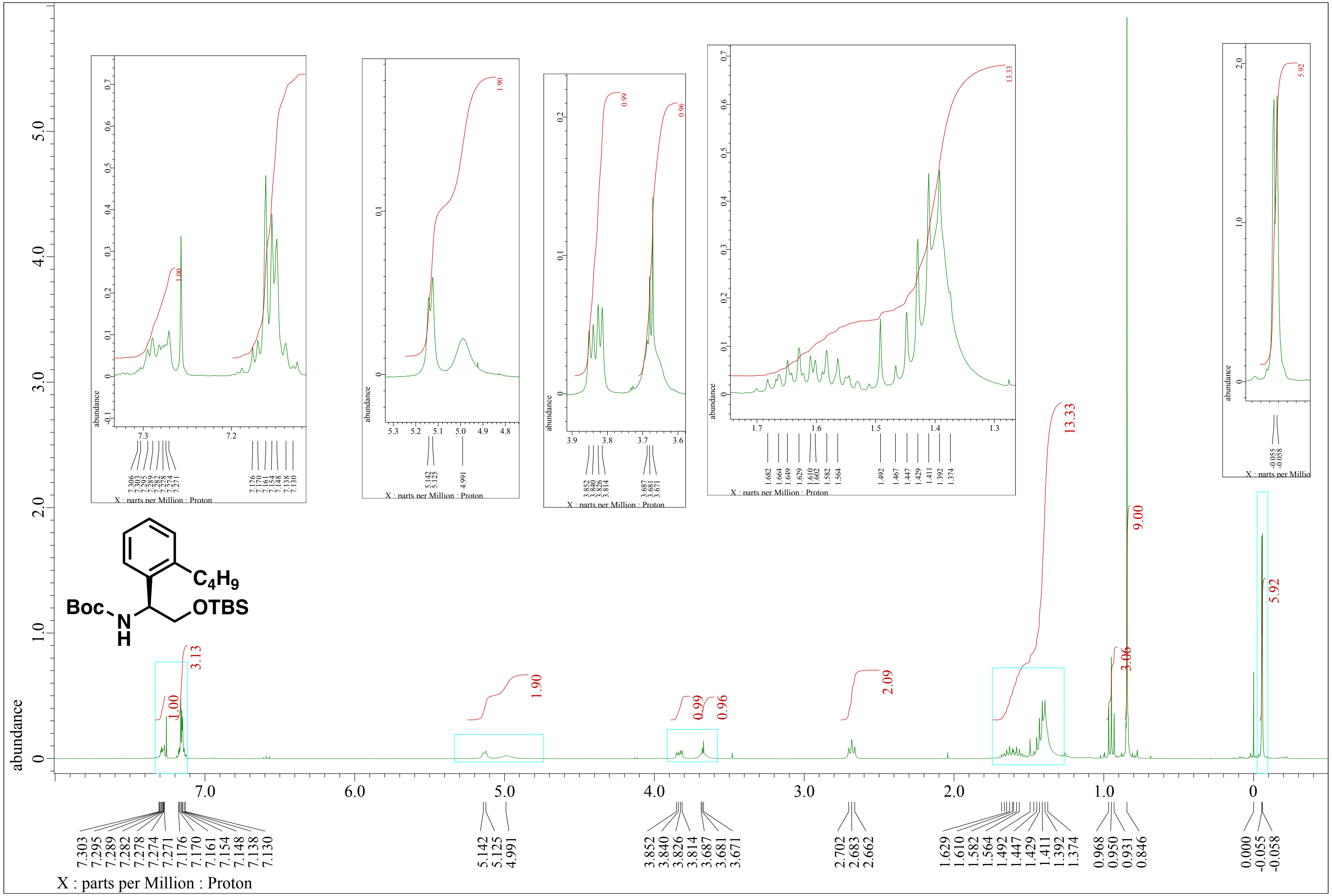

^{13}C NMR, 101 MHz, $CDCl_3$

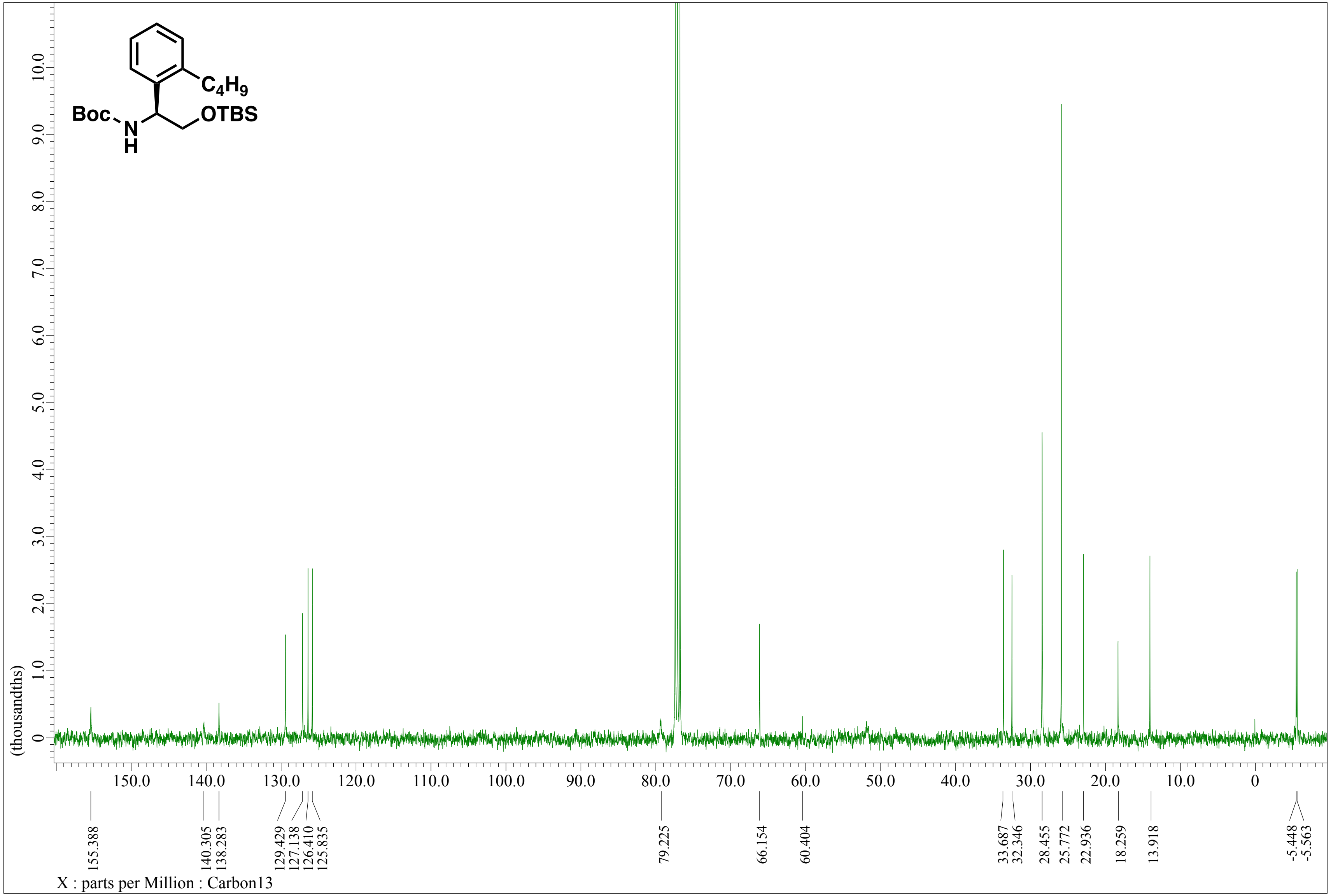

^{1}H NMR, 400 MHz, $CDCl_3$

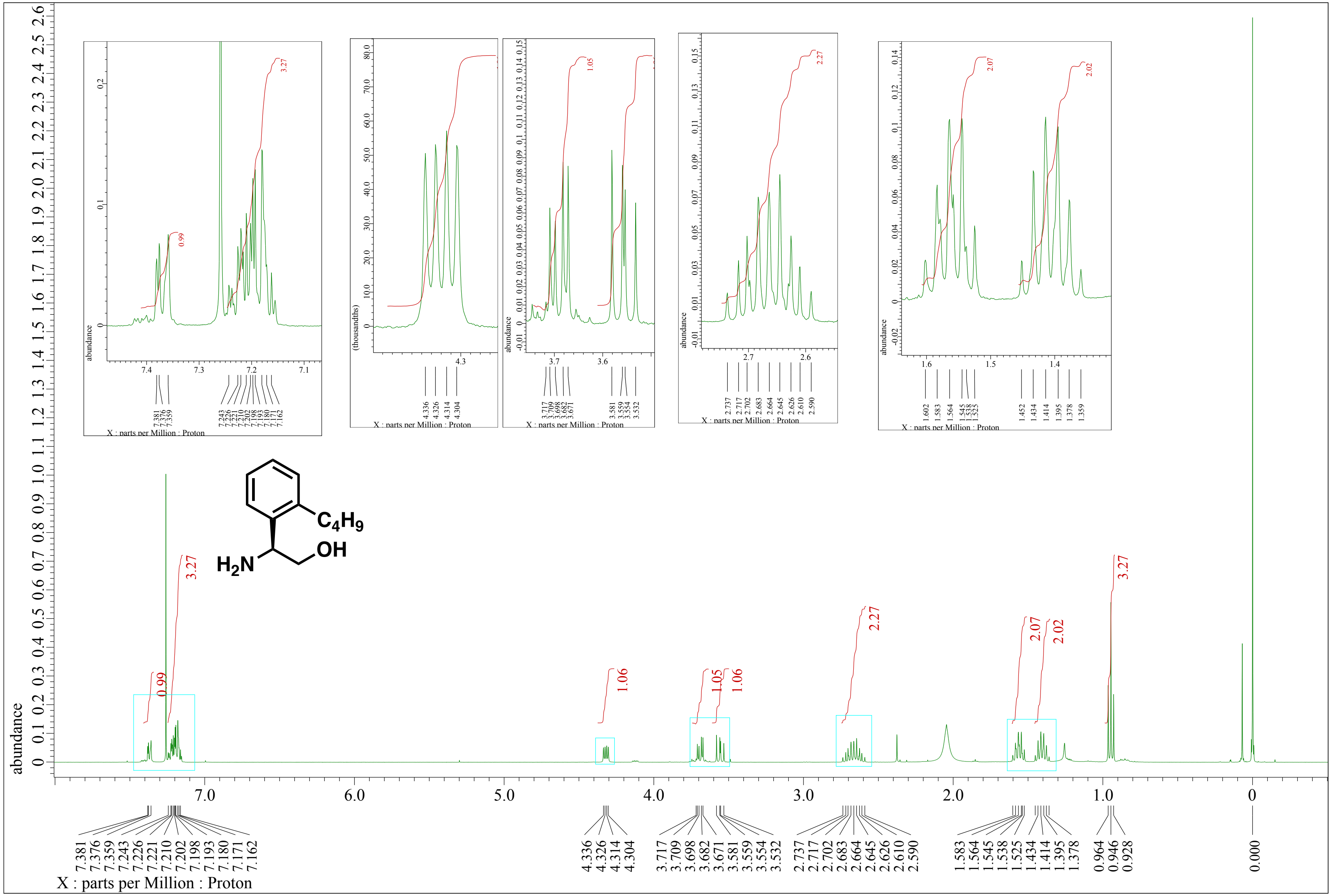

^{13}C NMR, 101 MHz, $CDCl_3$

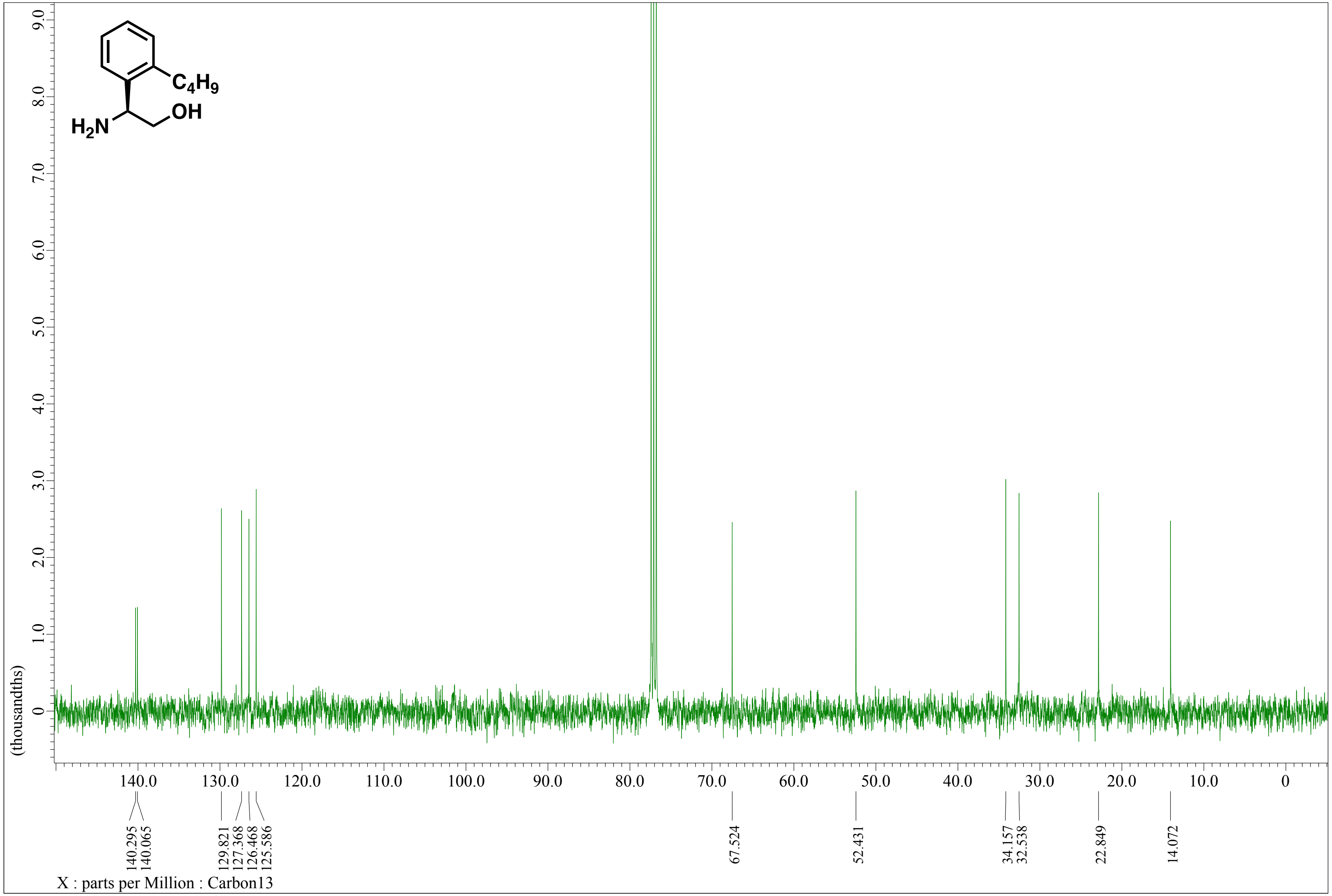

^{1}H NMR, 400 MHz, $CDCl_3$

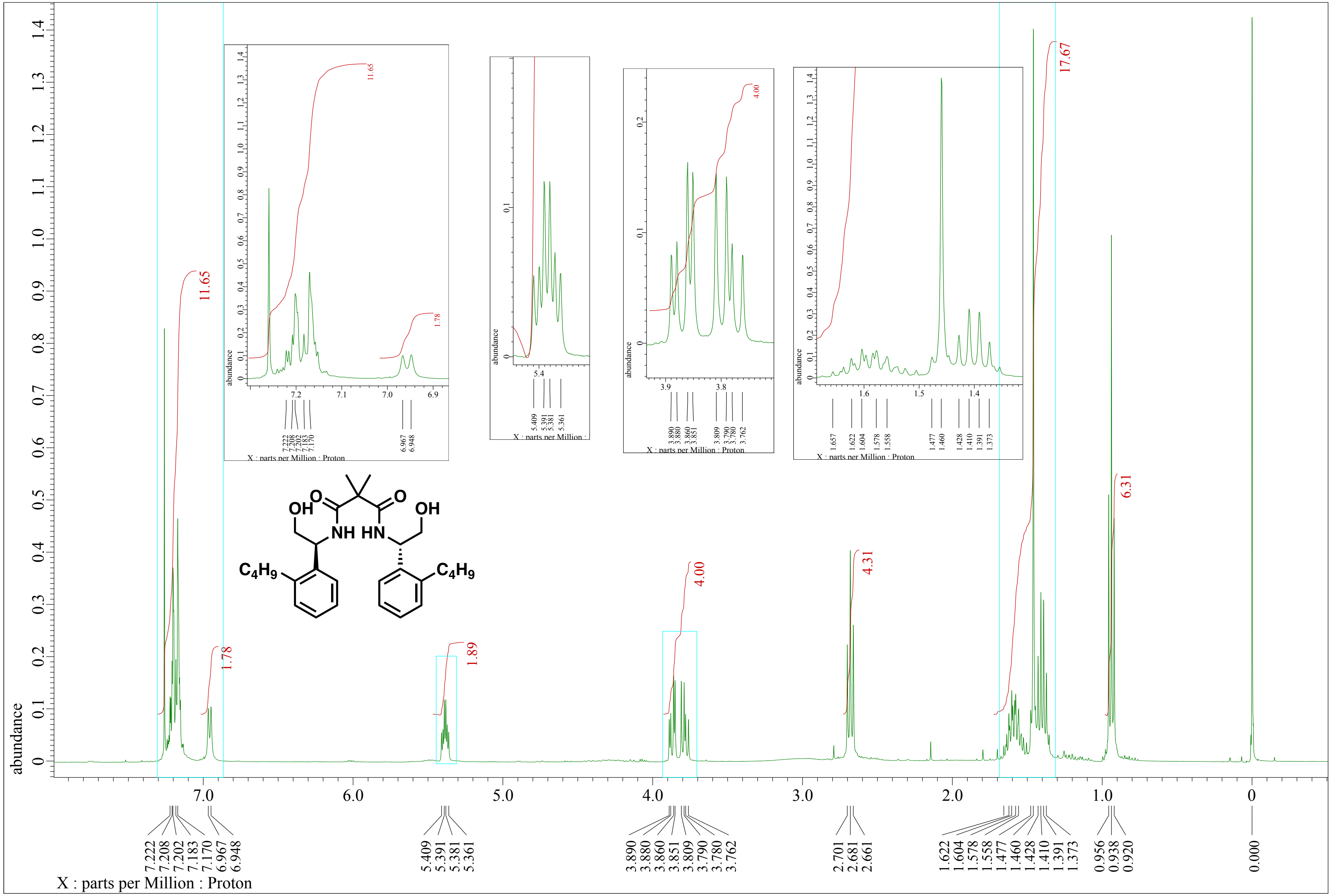

^{13}C NMR, 101 MHz, $CDCl_3$

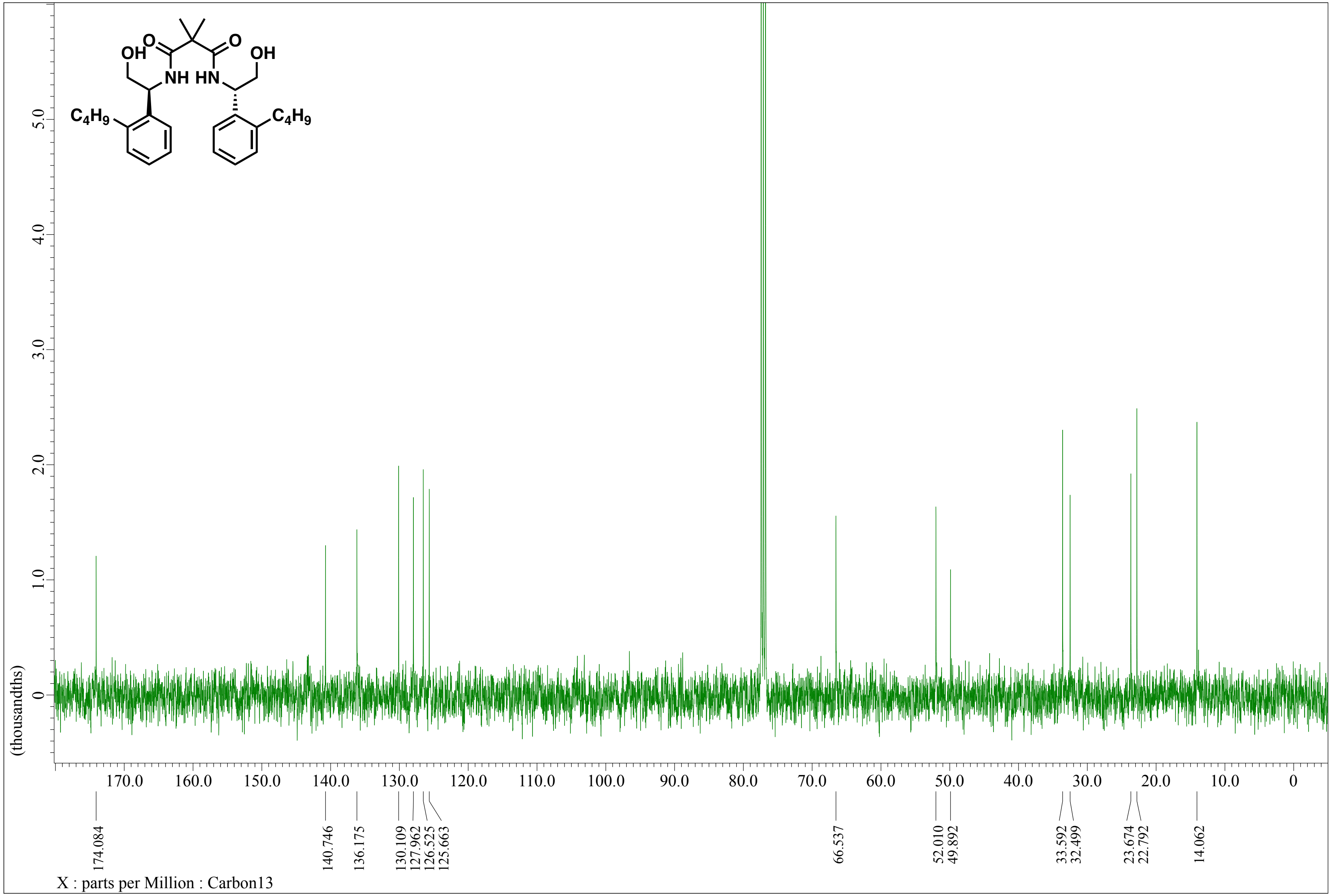

^{1}H NMR, 400 MHz, $CDCl_3$

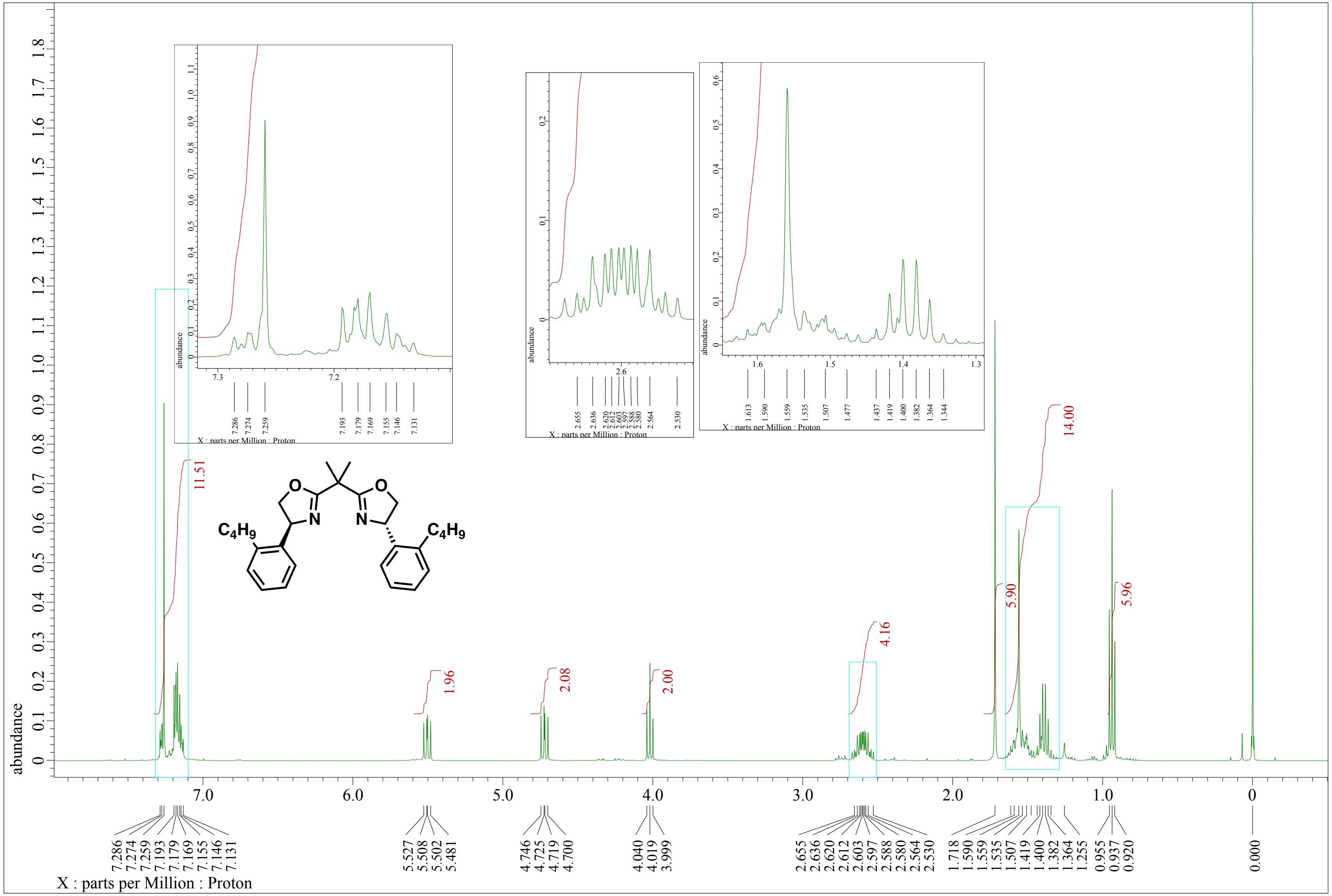

^{13}C NMR, 101 MHz, $CDCl_3$

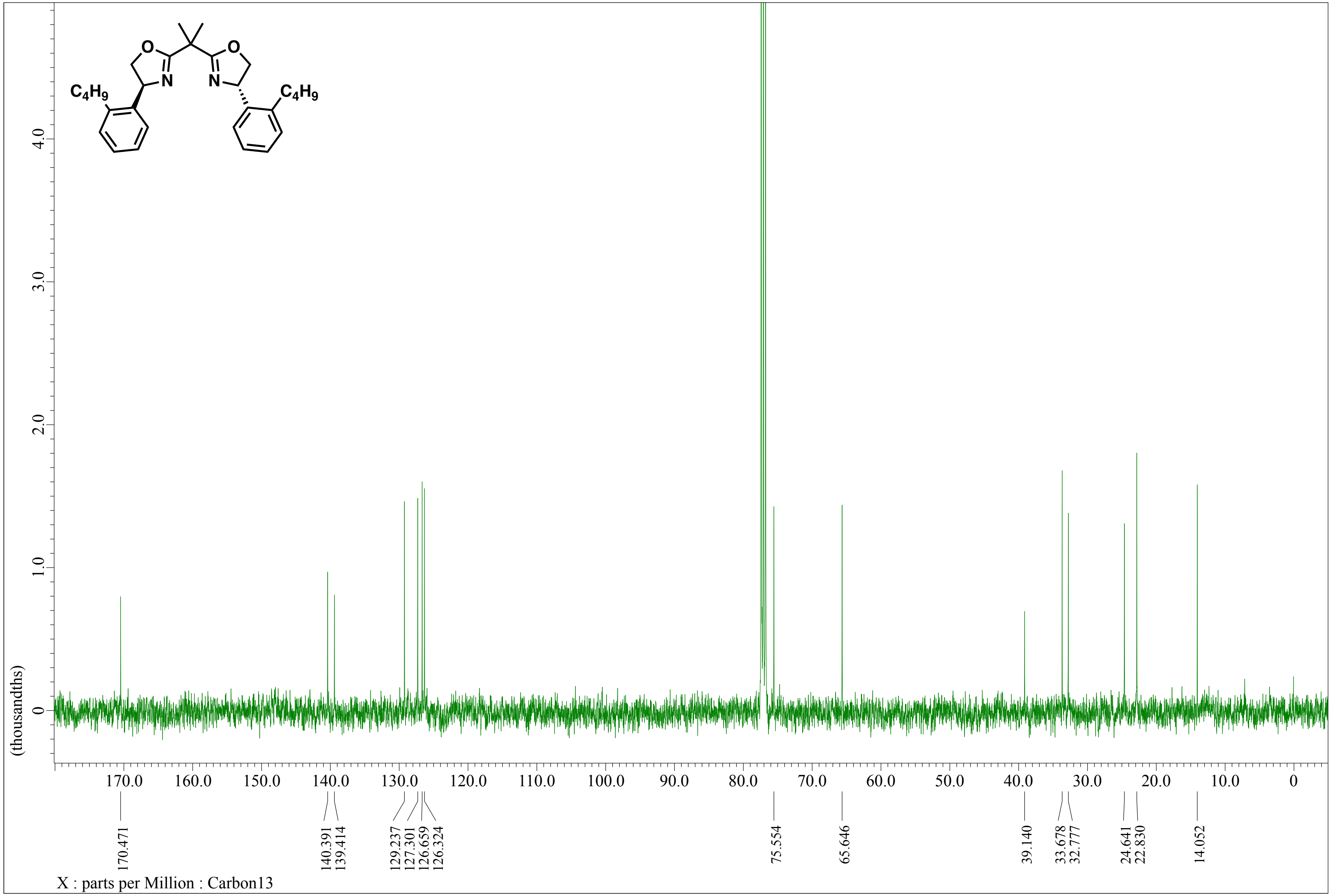

^{1}H NMR, 400 MHz, $CDCl_3$

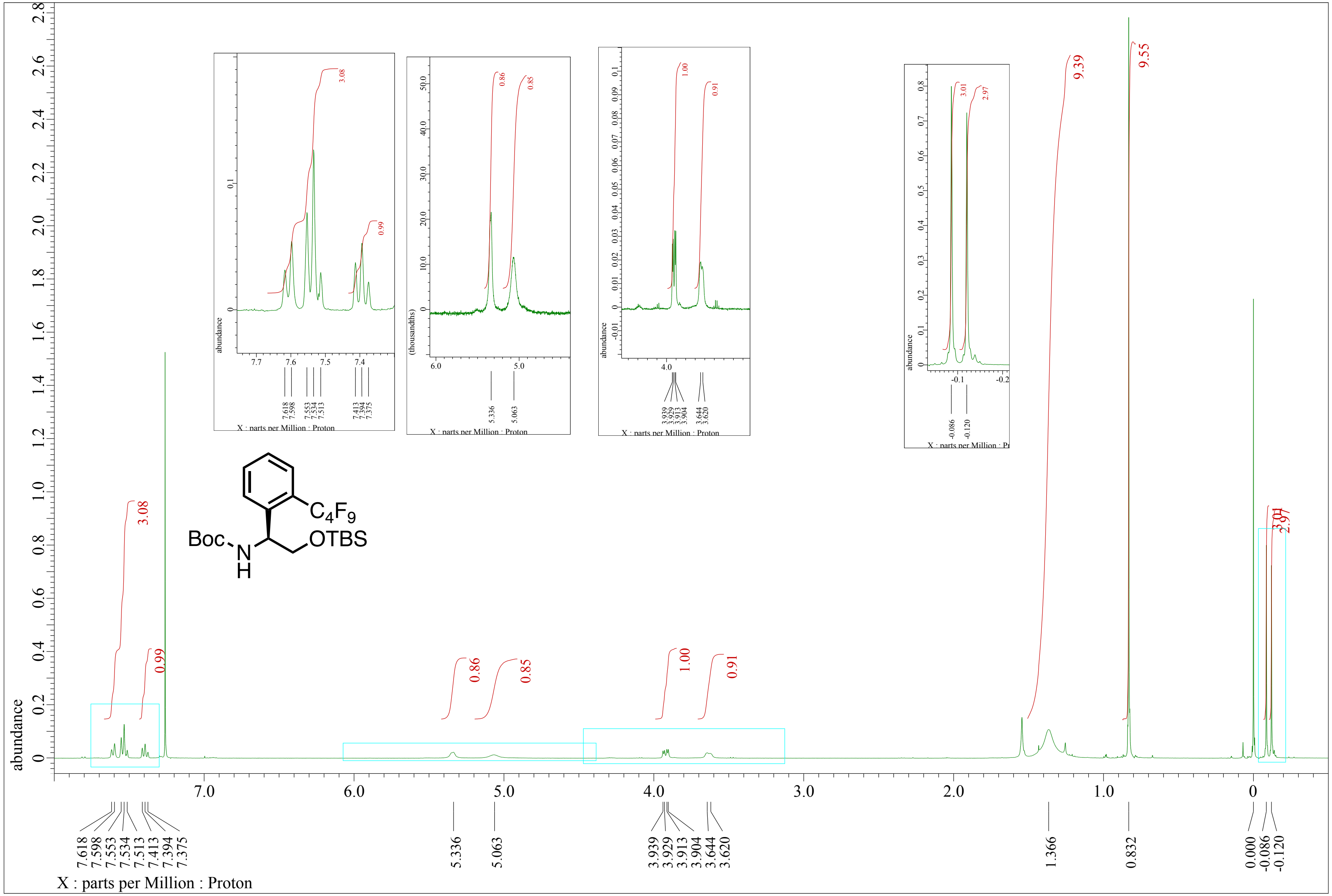

^{19}F NMR, 376 MHz, $CDCl_3$

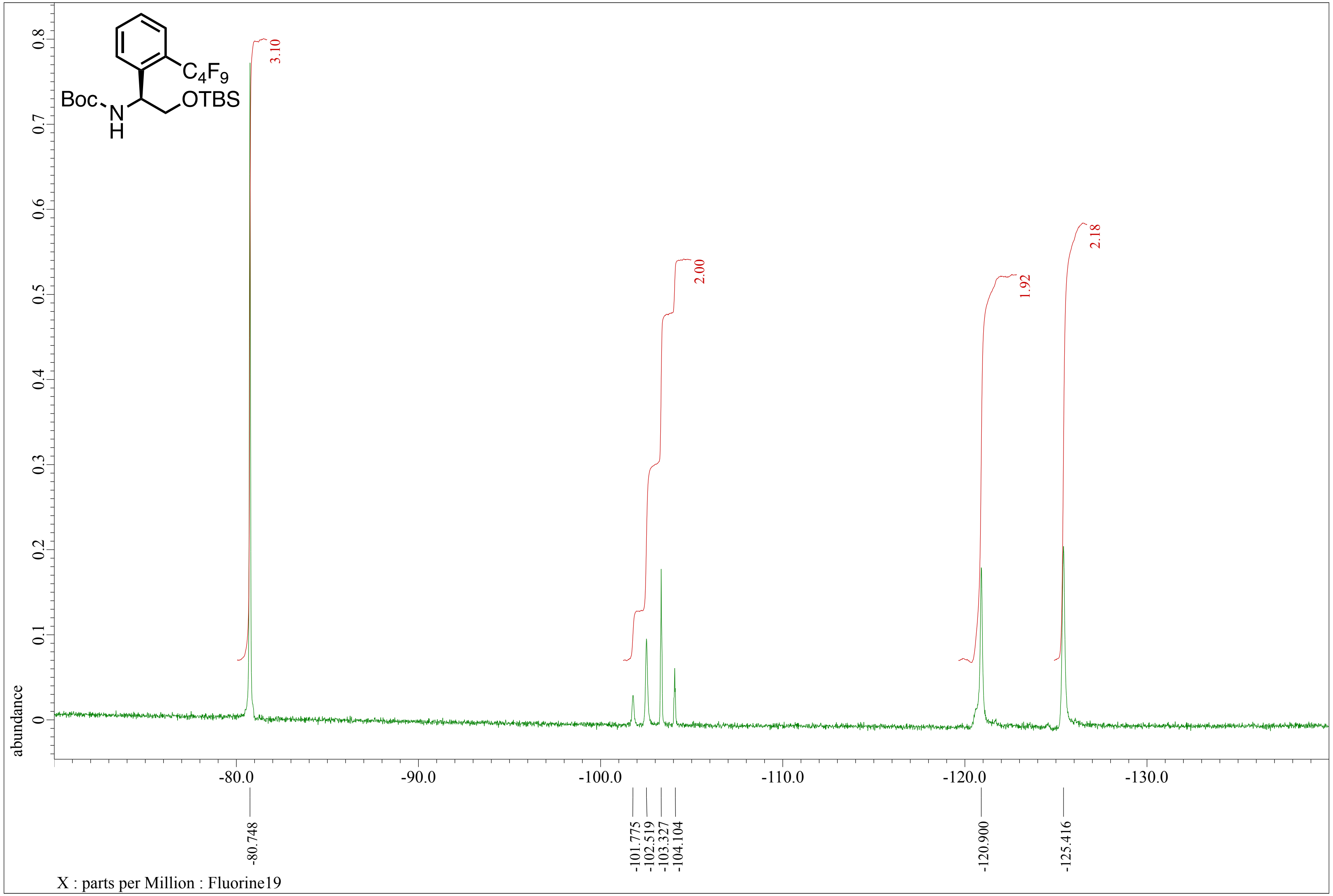

^{13}C NMR, 101 MHz, $CDCl_3$

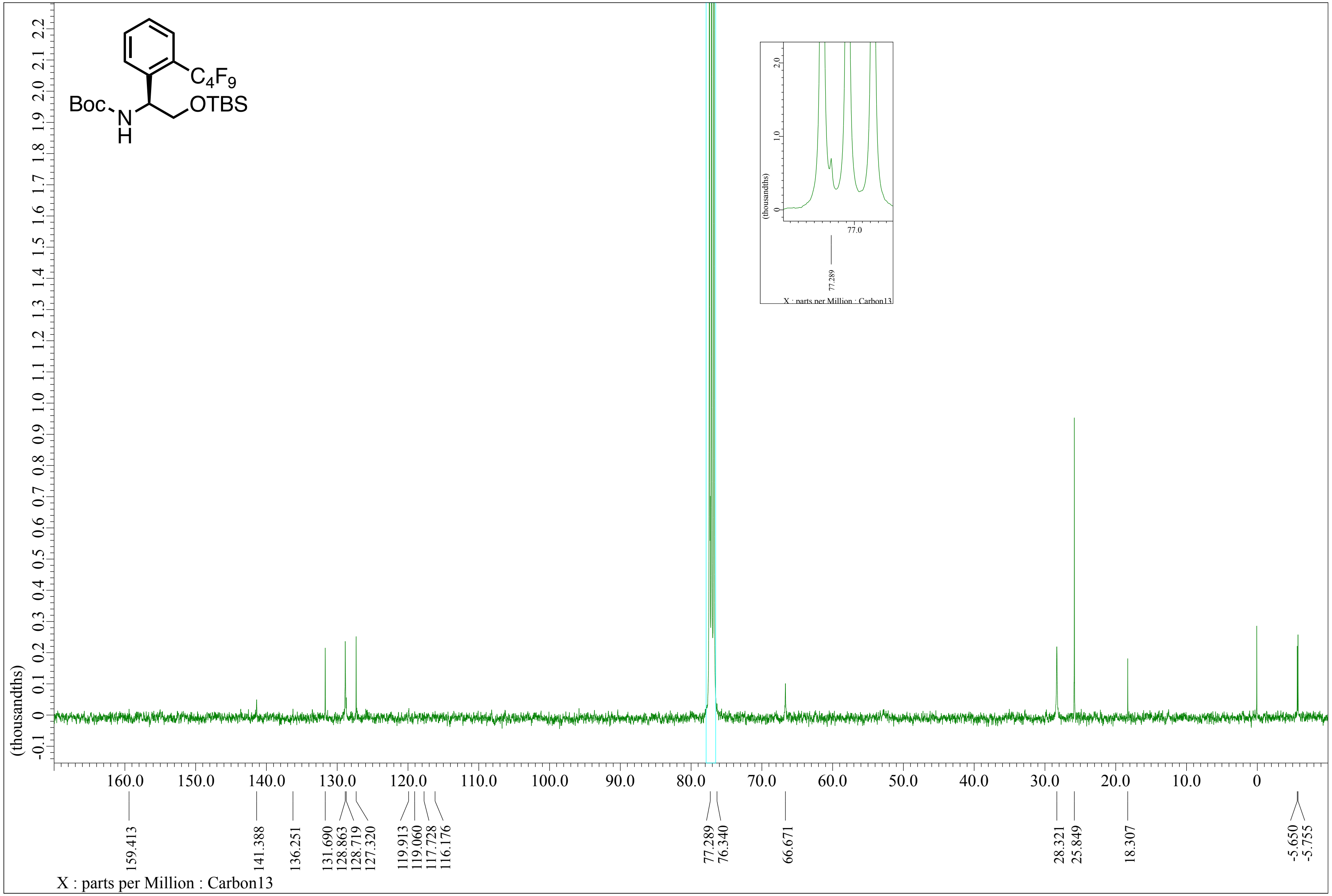

^{1}H NMR, 400 MHz, $CDCl_3$

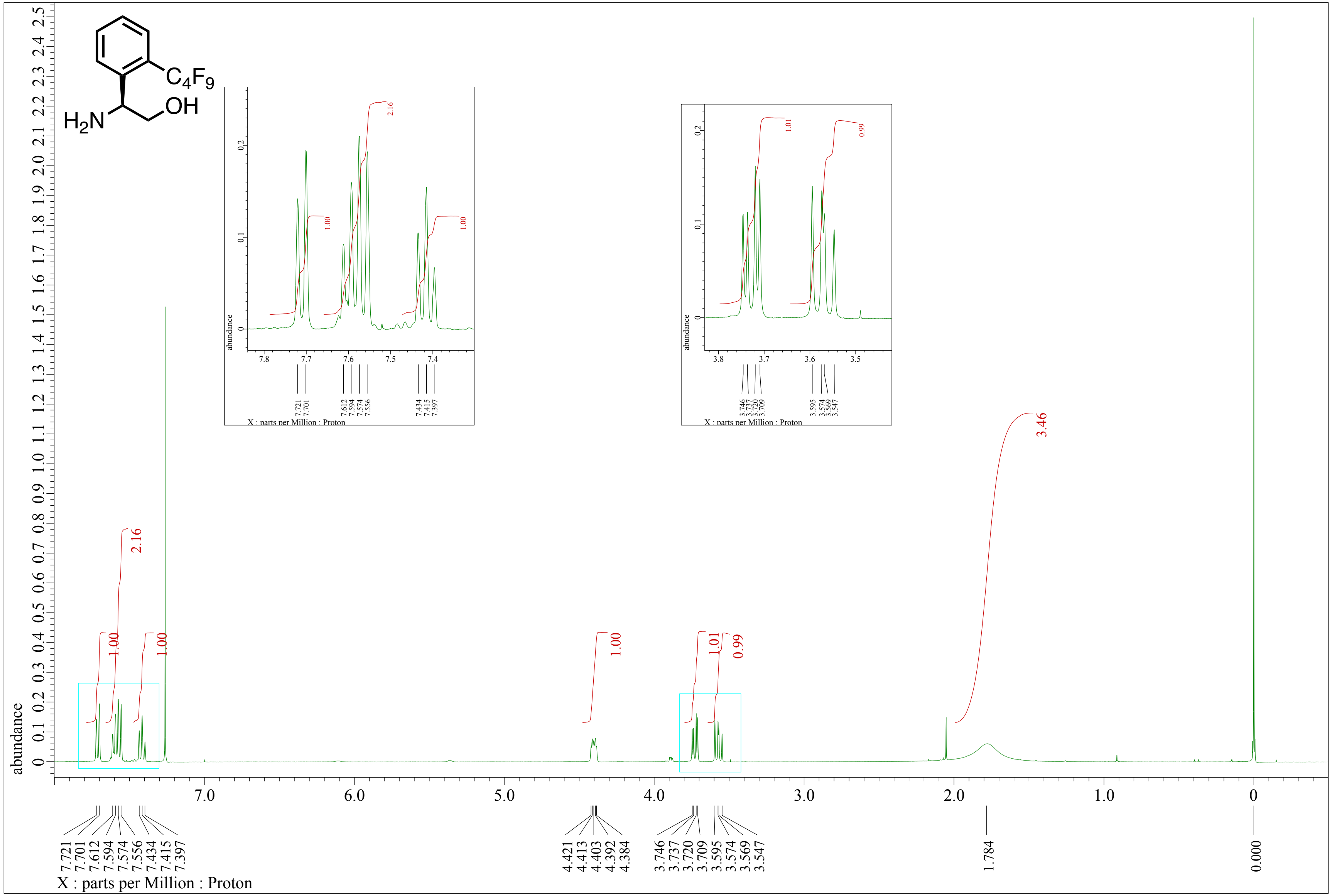

^{19}F NMR, 376 MHz, $CDCl_3$

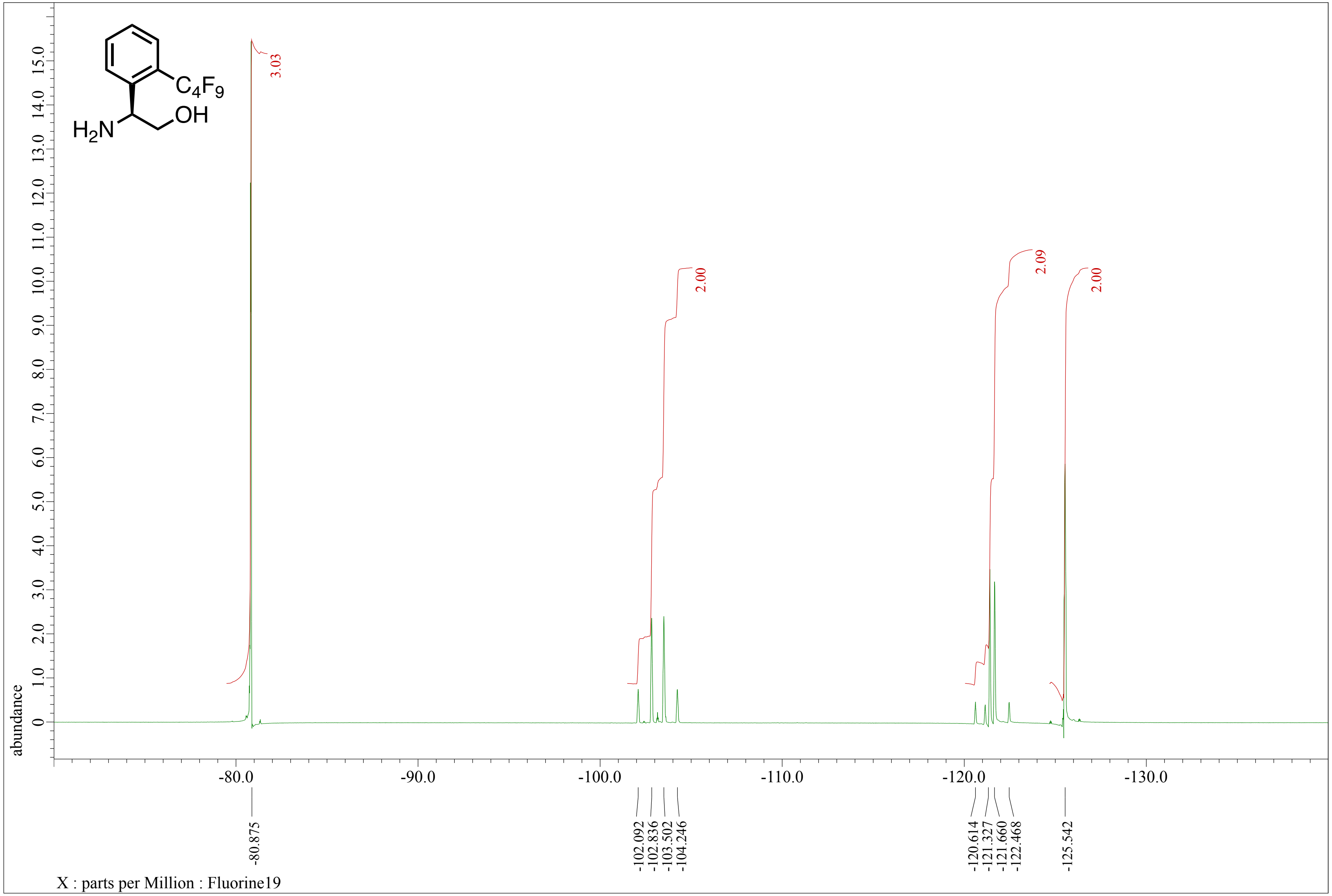

^{13}C NMR, 101 MHz, $CDCl_3$

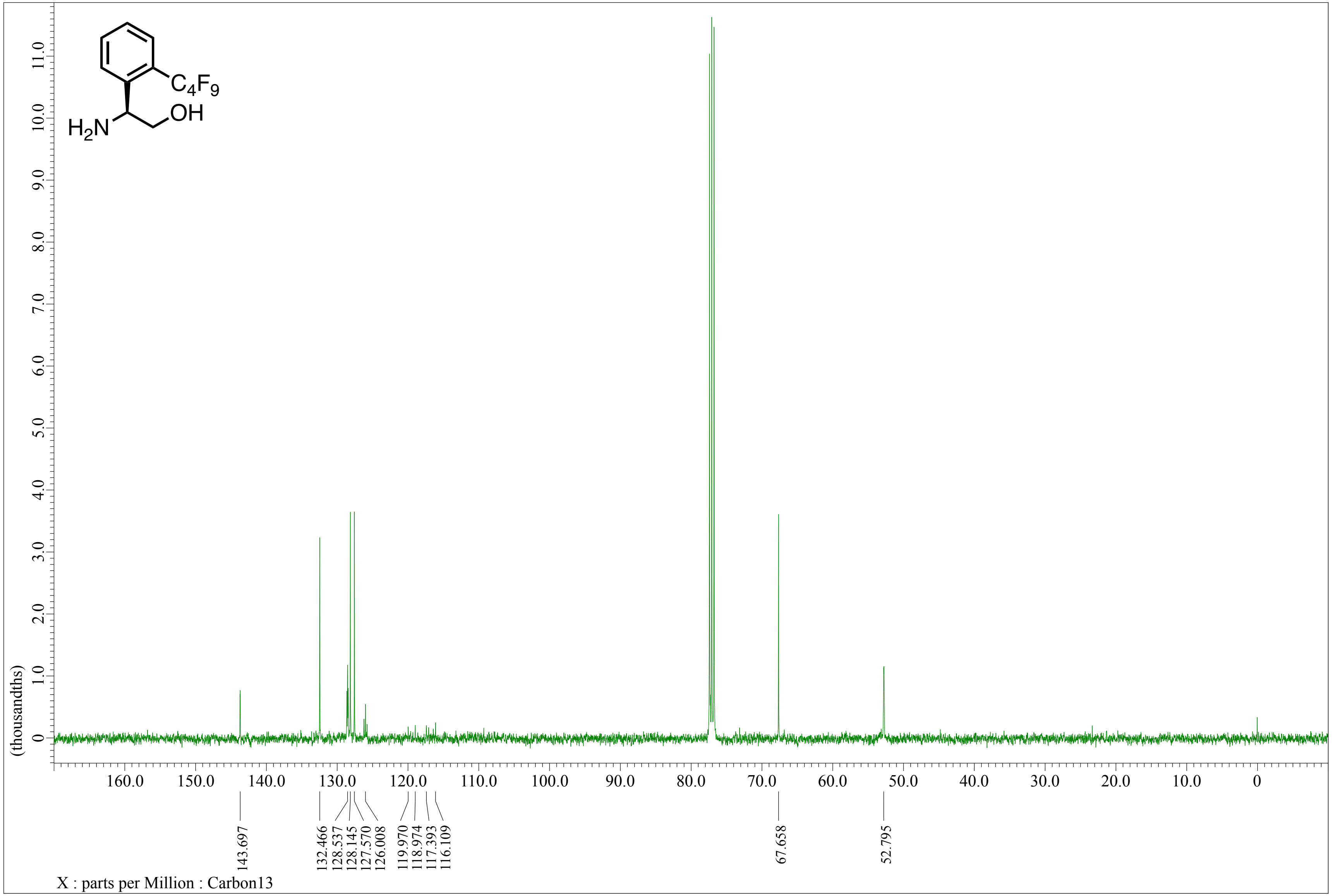

^{1}H NMR, 400 MHz, $CDCl_3$

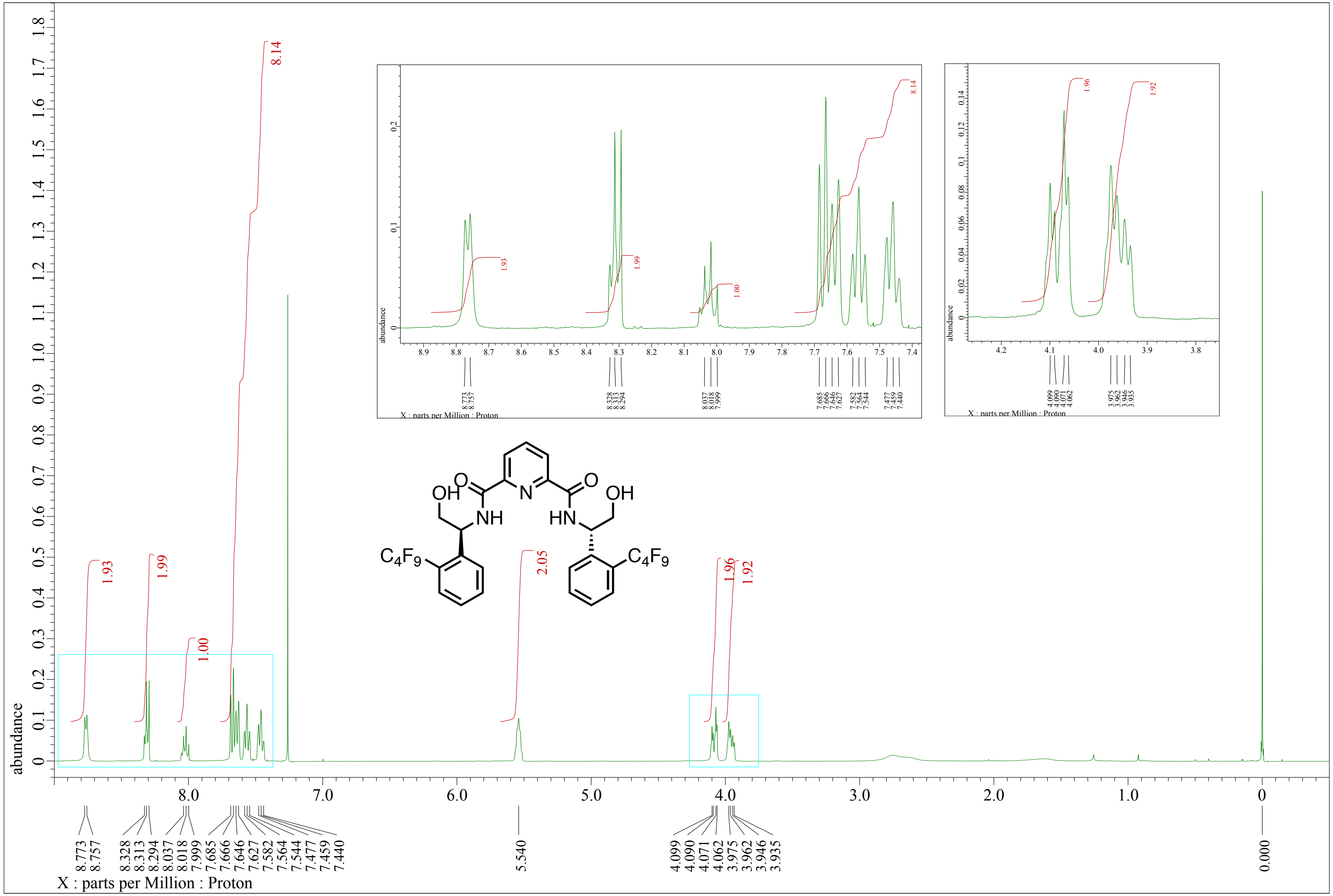

^{19}F NMR, 376 MHz, $CDCl_3$

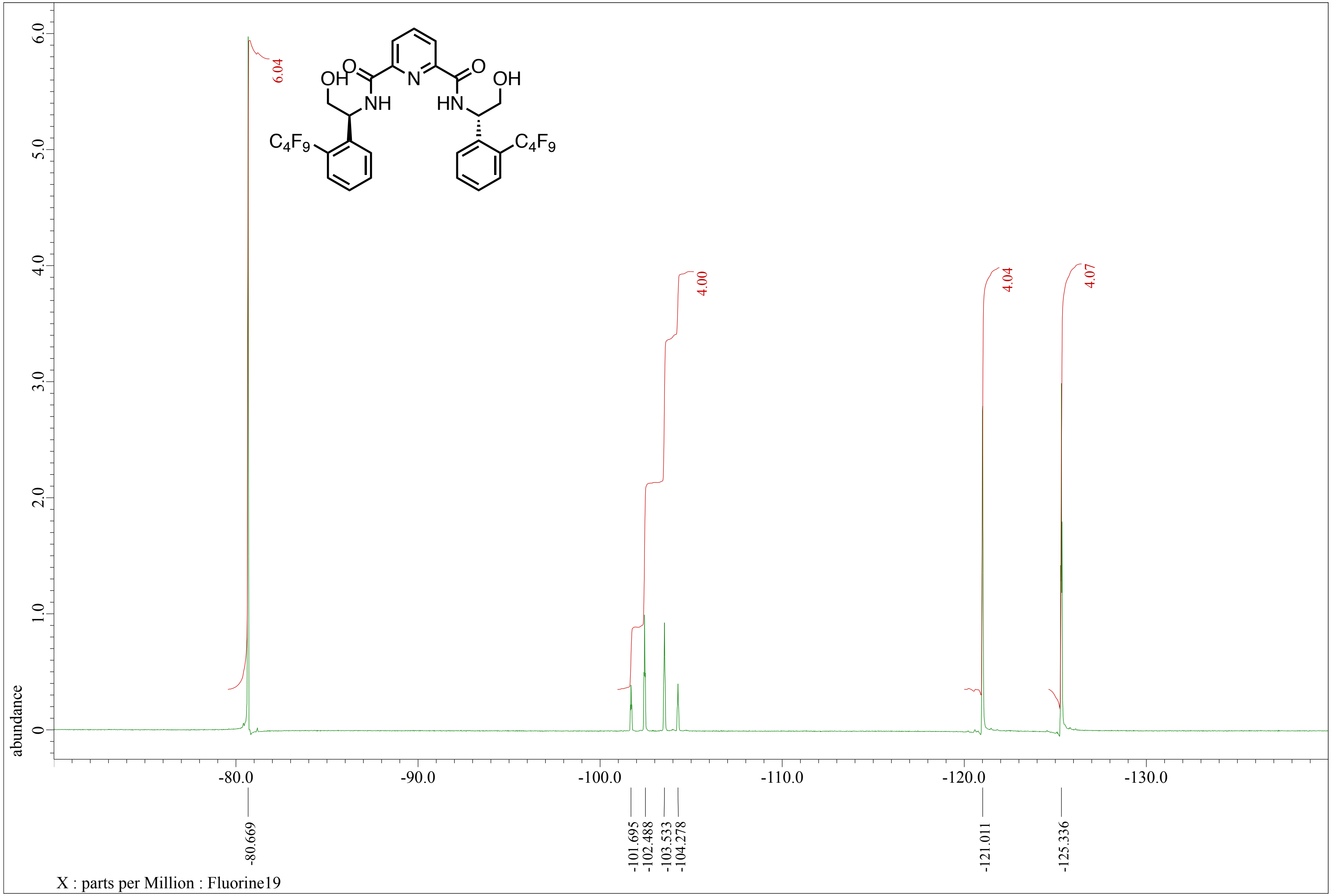

^{13}C NMR, 101 MHz, $CDCl_3$

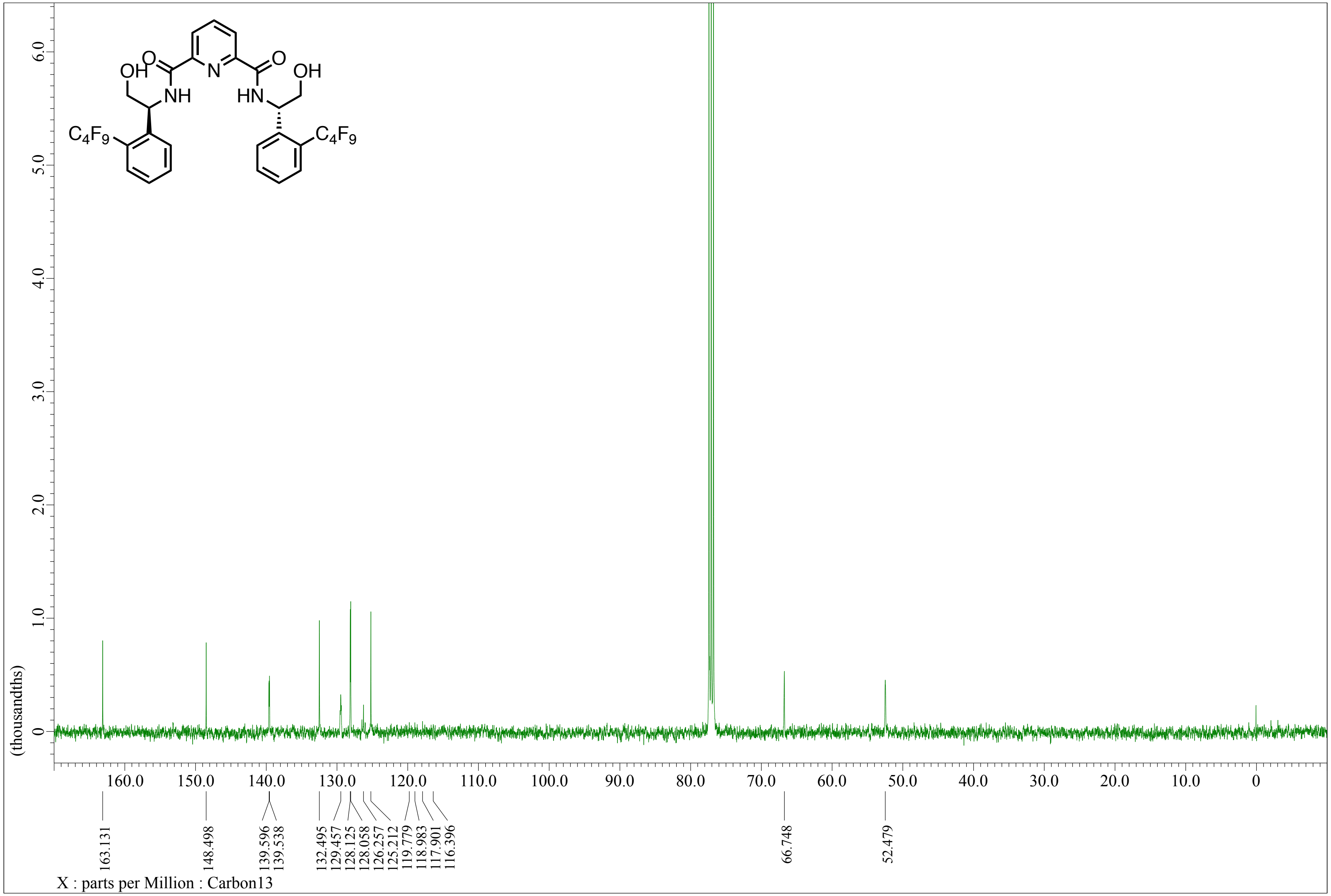

^{1}H NMR, 400 MHz, $CDCl_3$

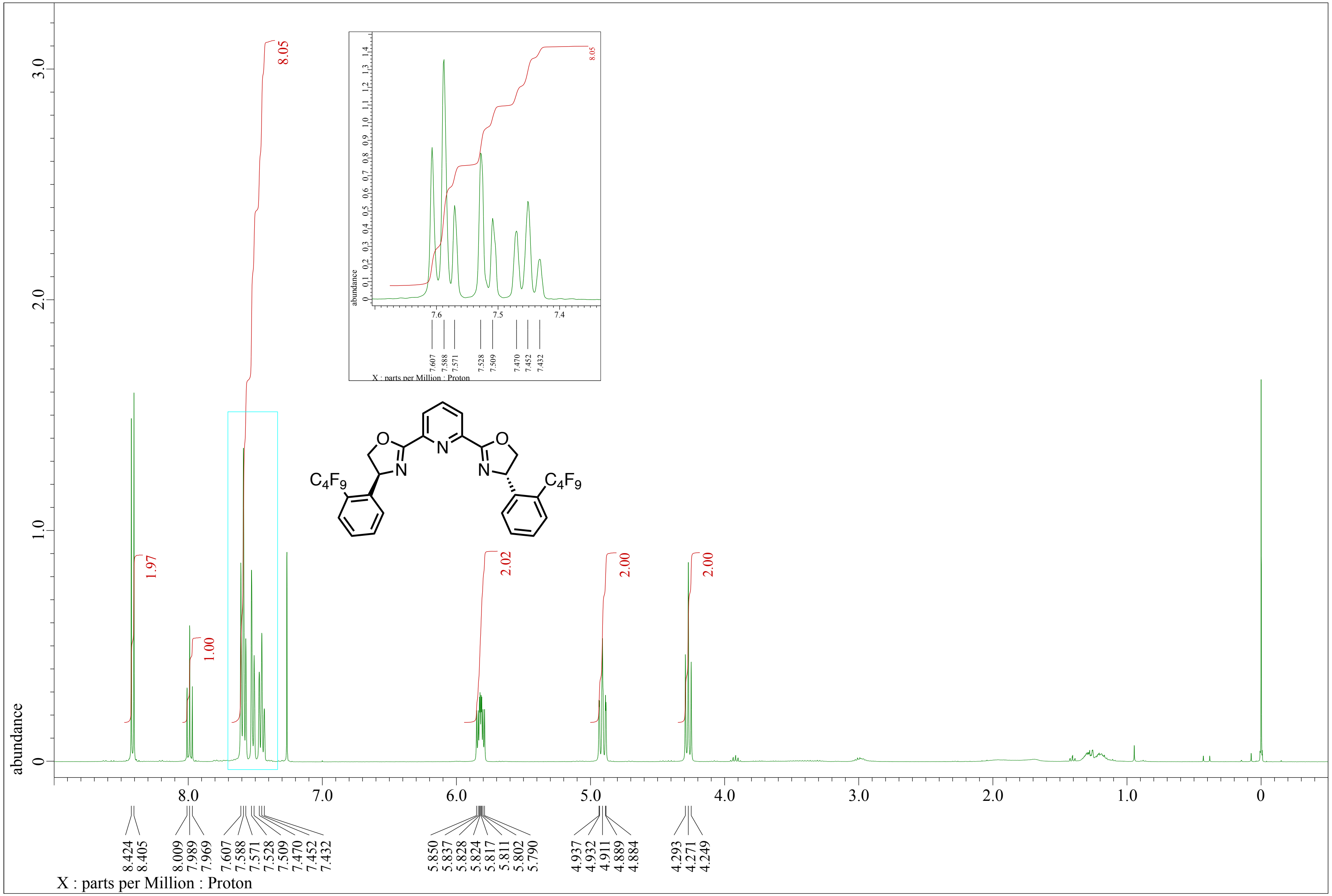

^{19}F NMR, 376 MHz, $CDCl_3$

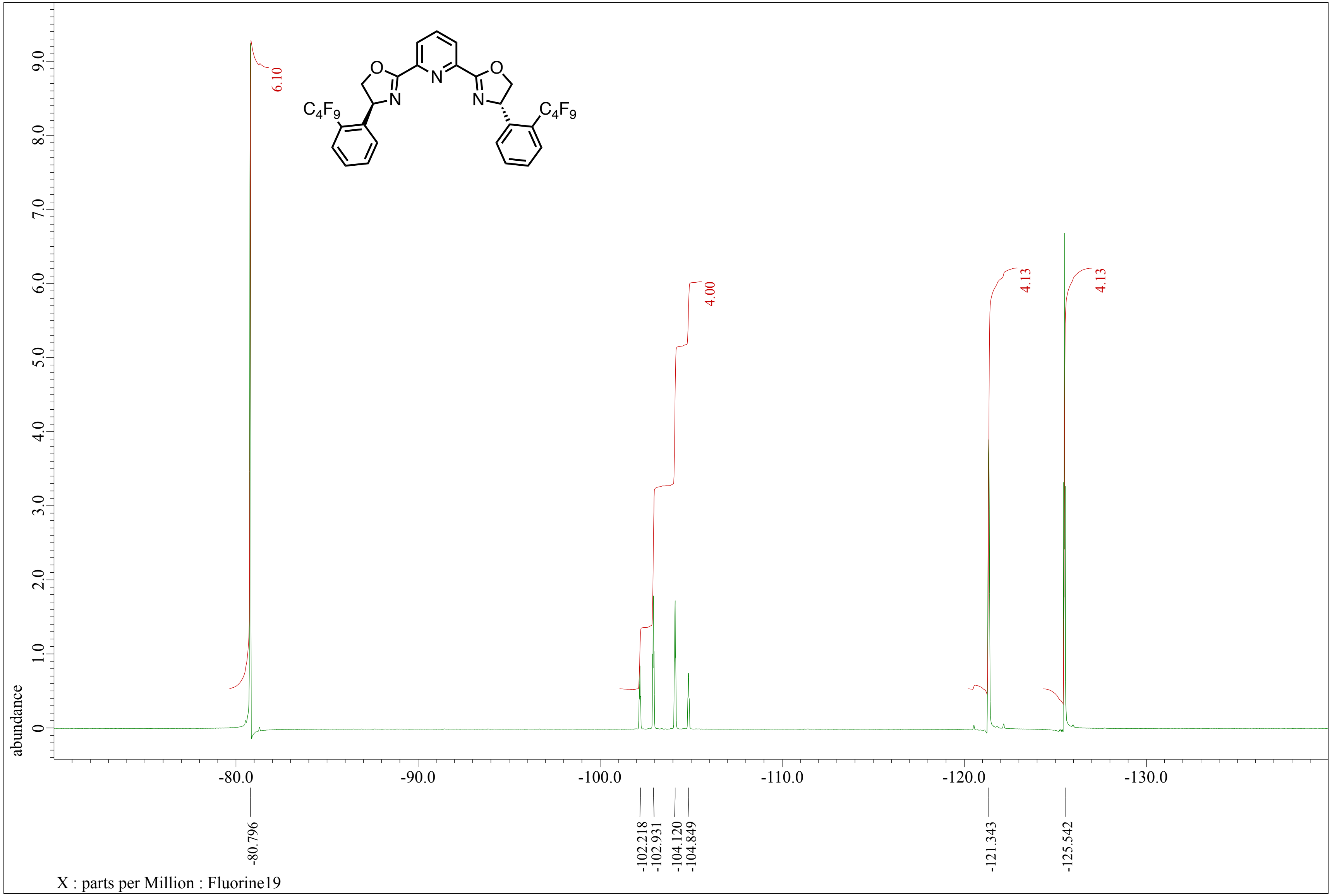

^{13}C NMR, 101 MHz, $CDCl_3$

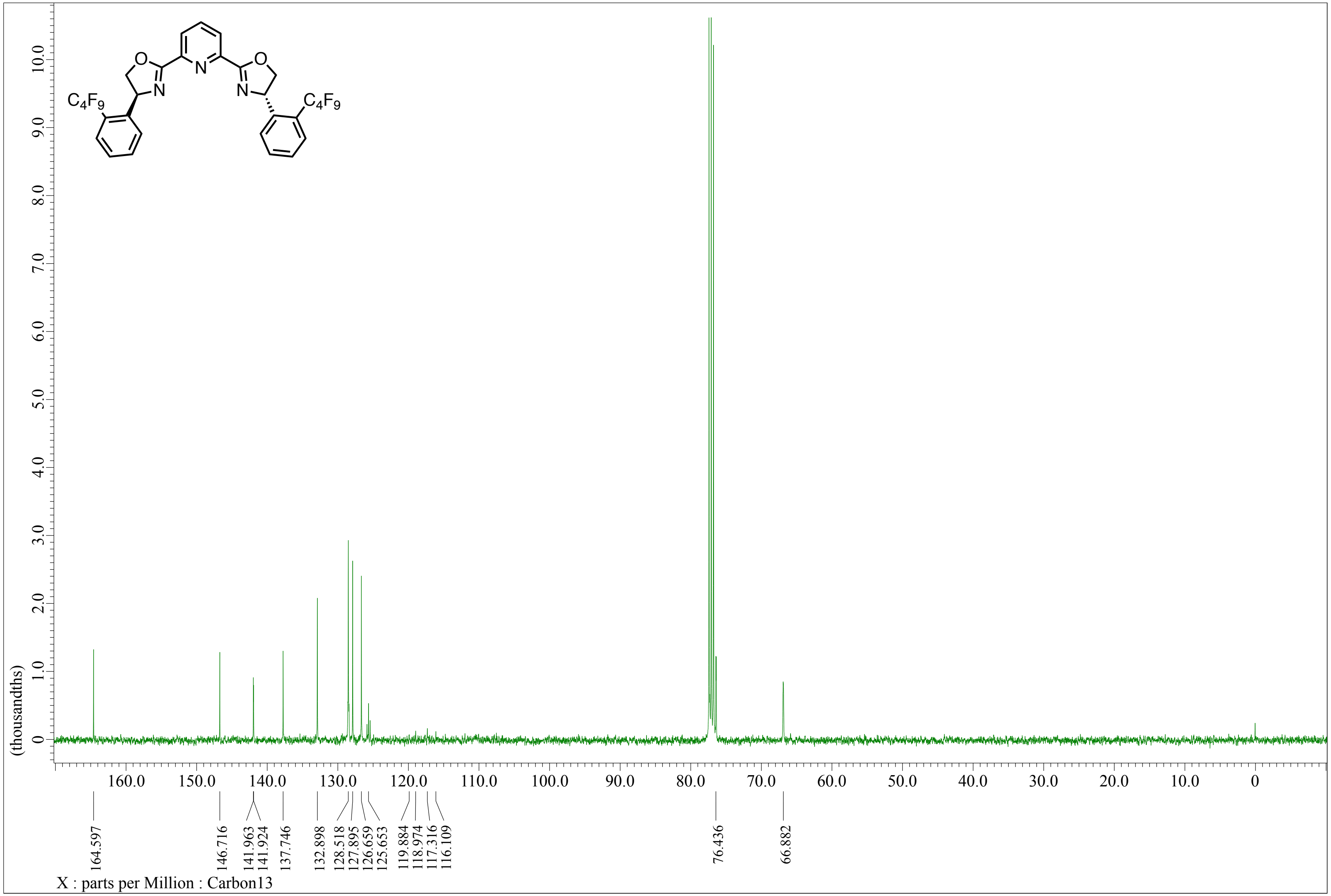

^{1}H NMR, 400 MHz, $CDCl_3$

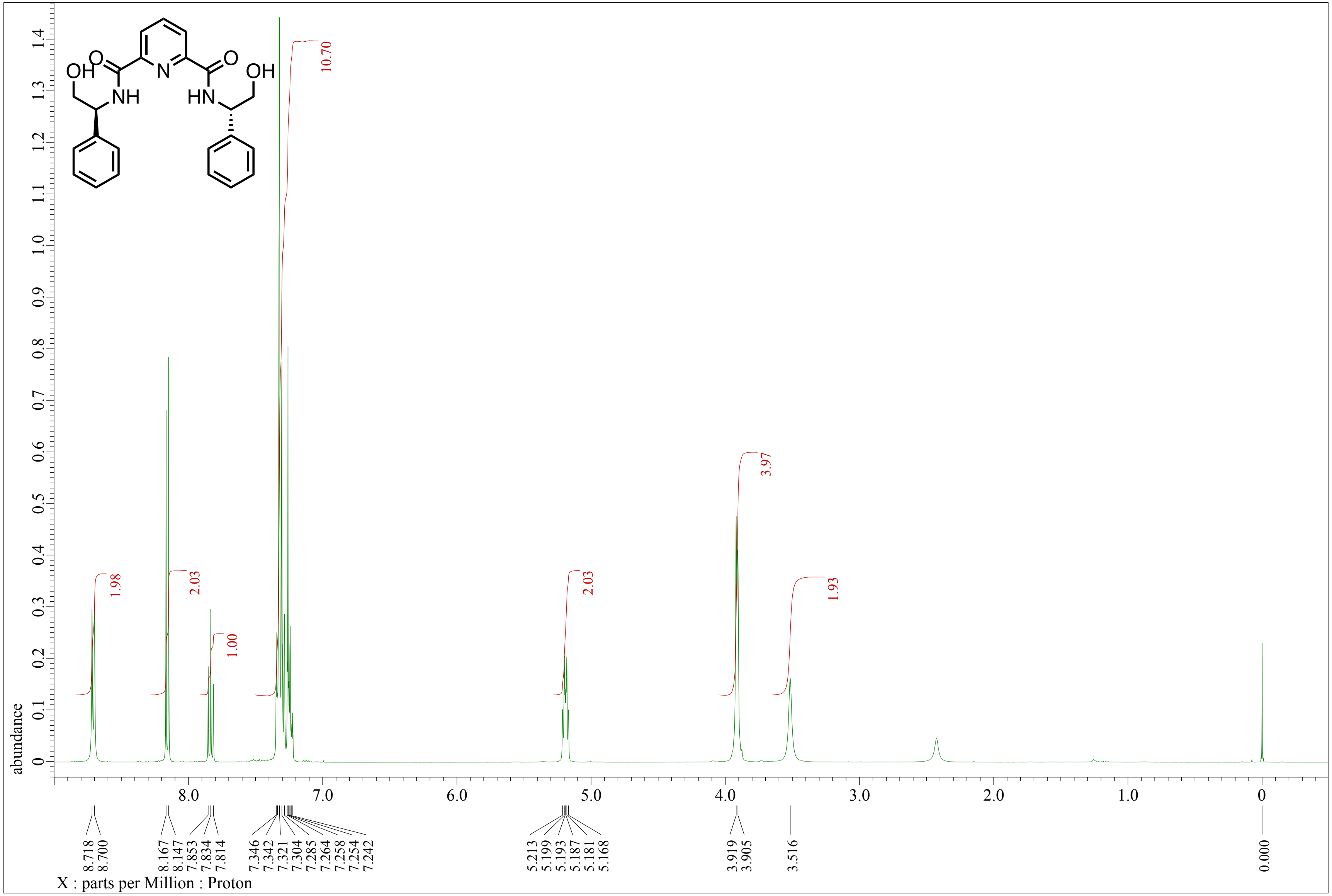

^{1}H NMR, 400 MHz, $CDCl_3$

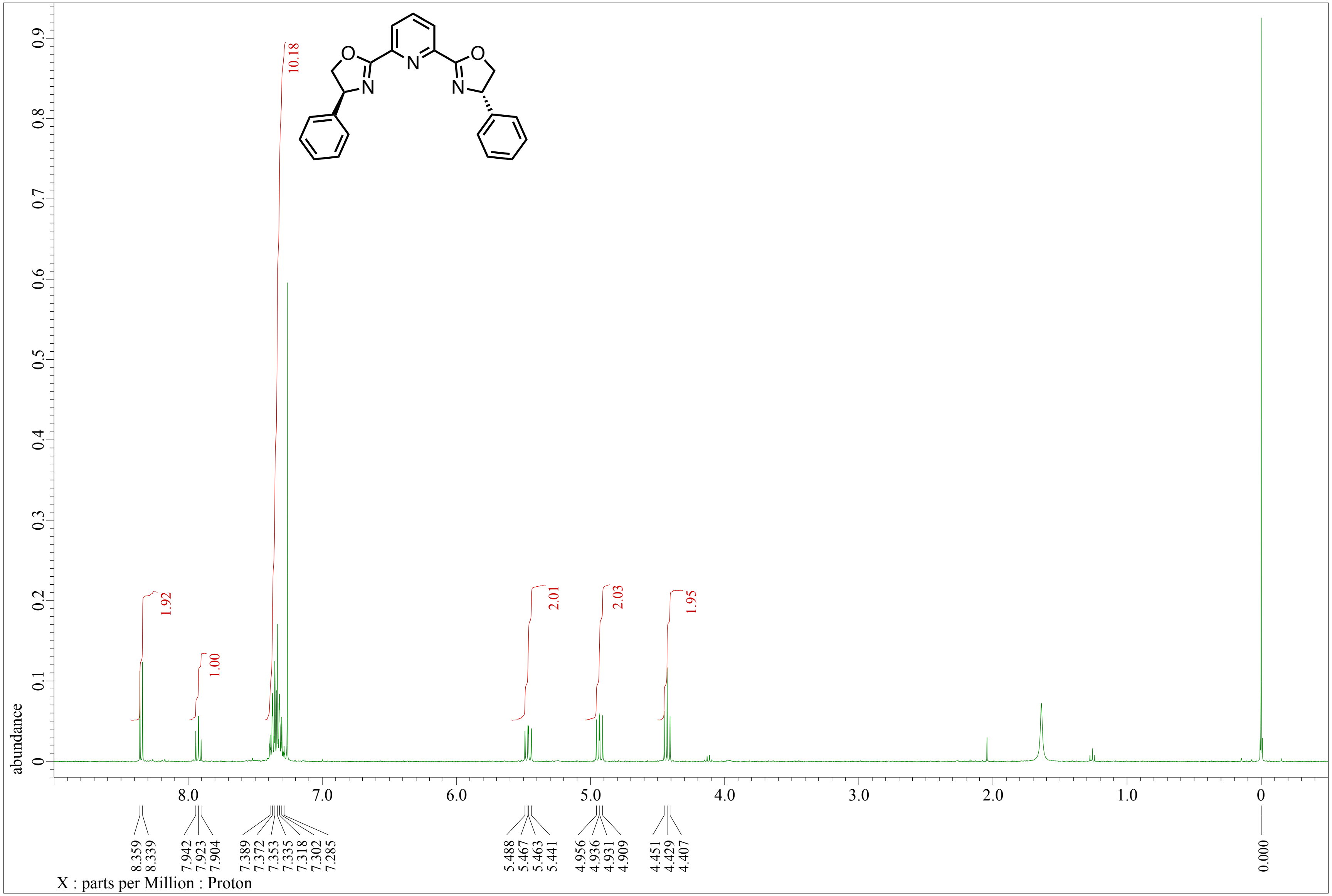

Supplement: Supplementary file 1 [file molecules-28-07632-s001.zip › molecules-2673967-supplementary.pdf]
